# SCP4 dephosphorylates mitotic histone H3 to maintain chromosome stability

**DOI:** 10.1038/s44319-026-00833-1

**Published:** 2026-06-19

**Authors:** Yupiao Zheng, Zhengmao Zhang, Qi Wang, Jin Cao, Yang Li, Tingbo Liang, Xin-Hua Feng, Xia Lin

**Affiliations:** 1 Department of Hepatobiliary and Pancreatic Surgery and Zhejiang Provincial Key Laboratory of Pancreatic Disease, The First Affiliated Hospital, Zhejiang University School of Medicine, Hangzhou, China; 2 Department of Pathology and Laboratory Medicine, Weill Cornell Medical College, New York, NY USA; 3 MOE Key Laboratory of Biosystems Homeostasis & Protection and Innovation Center for Cell Signaling Network, Life Sciences Institute, Zhejiang University, Hangzhou, China

**Keywords:** Cell Cycle, Development, DNA Replication, Recombination & Repair

## Abstract

Mitosis is tightly regulated at multiple levels to ensure chromosome stability. The transient phosphorylation of histone H3 at Threonine 3 (H3T3) during cell division is critical for proper chromosome condensation and the accurate segregation of sister chromatids. While Haspin has been identified as the kinase responsible for H3T3 phosphorylation during mitosis, the phosphatases that counteract this modification to maintain balanced phosphorylation levels remain under investigation. In this study, we systematically screened phosphatases encoded in the human genome and identified the nuclear phosphatase SCP4 as an H3T3 phosphatase. SCP4 modulates H3T3 phosphorylation levels and influences the chromosomal recruitment of chromosomal passenger complex (CPC) during mitosis. Aberrant SCP4 expression leads to defective chromosome separation during metaphase and chromosome lagging in anaphase, resulting in aneuploidy. Notably, in SCP4 knockout mice, zygotes exhibit mitotic defects during the first cleavage at the two-cell stage, highlighting SCP4’s essential role in ensuring faithful cell division. In summary, we identify SCP4 as a novel phosphatase regulating H3T3 phosphorylation and chromosome dynamics during mitosis, providing new insights into mechanisms safeguarding genomic stability.

## Synopsis

We identify SCP4, a chromatin-associated phosphatase, as a novel and critical regulator of mitotic fidelity and genomic stability. This finding expands our understanding of the molecular mechanisms by which cells prevent mitotic errors that contribute to tumorigenesis and developmental abnormalities.

- SCP4 is a specific mitotic phosphatase for the dephosphorylation of histone H3 threonine 3 (H3T3).
- SCP4 controls the precise recruitment of the chromosomal passenger complex (CPC) to mitotic chromosomes.
- Dysregulated SCP4 activity leads to chromosome missegregation and aneuploidy.
- *Scp4* knockout causes catastrophic mitotic defects during zygotic cleavage at the two-cell stage.

## Abstract (web summary)

We identify SCP4, a chromatin-associated phosphatase, as a novel and critical regulator of mitotic fidelity and genomic stability. This finding expands our understanding of the molecular mechanisms by which cells prevent mitotic errors that contribute to tumorigenesis and developmental abnormalities.

## Graphical abstract

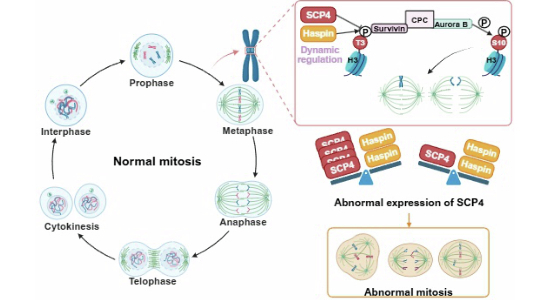

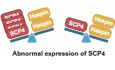

## Introduction

Faithful cell division is essential for normal organismal development. The regulation of mitosis and tissue growth involves complex mechanisms, among which post-translational modifications of histone H3, including acetylation, methylation, ubiquitination and phosphorylation, play crucial roles in modulating chromatin structure and chromosome dynamics (Andres et al, 2020; Hake et al, 2006; Jenuwein and Allis, 2001; Taylor and Young, 2021; Tolsma and Hansen, 2019; Wang and Higgins, 2013). During mitosis, phosphorylation at H3 N-terminal tail at sites such as threonine 3 (Thr3), serine 10 (Ser10), threonine 11 (Thr11), and serine 28 (Ser28) increases transiently (Goto et al, 1999; Polioudaki et al, 2004; Preuss et al, 2003; Prigent and Dimitrov, 2003; Zhou et al, 2008). These modifications are critical for proper chromosome condensation and sister chromatid segregation (Dai et al, 2005; de la Barre et al, 2000; Goto et al, 2002; Gurley et al, 1978; Nowak and Corces, 2004; Wei et al, 1998). Disrupted H3 phosphorylation can lead to aberrant chromosome behavior, including lagging chromosomes, chromosome bridges, and aneuploidy (Dai et al, 2005; Giet and Glover, 2001; Goto et al, 1999; Goto et al, 2002; Gurley et al, 1978; Nowak and Corces, 2004; Ota et al, 2002; Panigrahi and Pati, 2009).

Notably, phosphorylation at Thr3 (H3T3) by the kinase Haspin is essential for recruiting the chromosomal passenger complex (CPC) to chromosomes, thereby activating the mitotic kinase Aurora B (Hadders et al, 2020; Higgins, 2010; Kelly et al, 2010; Kestav et al, 2017; Wang et al, 2010; Wang et al, 2011; Yamagishi et al, 2010). Aurora B, in turn, phosphorylates H3 at Ser10, initiating spindle assembly and preventing premature nuclear reformation (Higgins, 2010; Kelly et al, 2010; Wang et al, 2010; Yamagishi et al, 2010). Thus, H3T3 phosphorylation plays a direct role in ensuring mitotic fidelity. Importantly, H3T3 phosphorylation is tightly regulated, occurring only during the early mitotic phase. It localizes along chromosome arms at prometaphase and intensifies at centromeric chromatin during metaphase compared to its weaker existence on chromosome arms, and it decreases dramatically and rapidly afterward (Dai et al, 2005; Polioudaki et al, 2004; Zhou et al, 2006). The transient nature of H3T3 phosphorylation is critical, as evidenced by observations showing that when Haspin is overactive, chromosomes remain condensed with elevated levels of H3 Ser10 and Thr3 phosphorylation, disrupting mitotic exit to interphase (Higgins, 2010; Kelly et al, 2010; Wang et al, 2010).

Since Haspin activity is not regulated by the cell cycle (Dai and Higgins, 2005; Higgins, 2010; Kelly et al, 2010), the balance between kinase and phosphatase activities likely governs H3T3 phosphorylation dynamics. Previous studies using phosphatase inhibitors have implicated PP1 and PP2A in regulating Ser10 phosphorylation (Hunt, 2013; Murnion et al, 2001). Additionally, Repo-Man/PP1γ has been shown to dephosphorylate Ser10 and Thr3 during late mitosis, particularly contributing to the decrease in Ser10 and Thr3 phosphorylation in telophase (Qian et al, 2013; Qian et al, 2011; Vagnarelli et al, 2006; Vagnarelli et al, 2011; Wurzenberger et al, 2012). However, whether additional phosphatases fine-tune H3T3 phosphorylation at distinct mitotic stages remains to be explored.

Here, we identify the nuclear phosphatase SCP4 as a novel H3T3 phosphatase through a systematic phosphatase library screening. SCP4 belongs to the small C-terminal domain serine/threonine phosphatase family (Wrighton et al, 2006). The initial member of this family, FCP1, is known to dephosphorylate the C-terminal domain of RNA polymerase II, influencing RNA transcription (Qian et al, 2007). Other family members, including SCP1, SCP2, SCP3, and SCP4 have since been recognized for their diverse roles in various cellular processes (Cao et al, 2018; Li et al, 2024; Liu et al, 2017; Polyanskaya et al, 2022; Wang et al, 2024; Xiao et al, 2022; Zhao et al, 2018; Zhao et al, 2014). Our findings demonstrate that SCP4 regulates H3T3 phosphorylation during mitosis, and its dysregulation causes chromosome missegregation and aneuploidy.

## Results and discussion

### SCP4 is a H3T3 phosphatase

**Figure 1:**
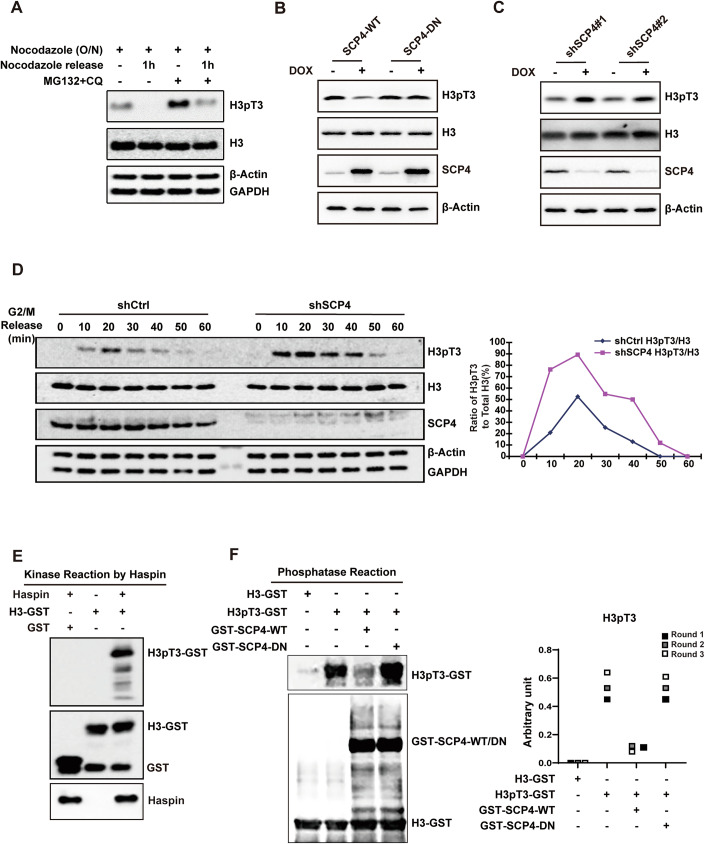
SCP4 dephosphorylates H3 at Thr3 site. (**A**) Thr3 site of H3 was phosphorylated (H3pT3) in HeLa cells synchronized at prometaphase by nocodazole treatment overnight. This phosphorylation declined rapidly 1 h post-prometaphase, which cannot be reversed by MG132 and chloroquine (CQ) treatment. β-Actin and GAPDH: internal controls for WB. (**B**) Dox-induced stable expression of wild-type SCP4 (SCP4-WT), but not the catalytically inactive mutant SCP4 (SCP4-DN) decreased H3pT3 level in HeLa cells. β-Actin: internal control for WB. (**C**) Dox-induced knockdown of SCP4 by shSCP4 increased H3pT3 level in HeLa cells. β-Actin: internal control for WB. (**D**) HeLa cells were synchronized at the G2/M phase by RO-3306 treatment and then released to resume cell cycle progression in regular culture medium. The decline of H3pT3 level during cell cycle progression was delayed in SCP4 knockdown (shSCP4) cells compared to control (shCtrl) cells. Relative signal density ratios of H3pT3 to H3 were quantified using ImageJ (Version 1.54p) and are presented as arbitrary units. Experiments were performed three times independently (*n* = 3), and results from the first experiment are presented as representative data. All raw data were provided in the Source Data File. β-Actin and GAPDH: internal controls for WB. (**E**) Purified GST fusion protein of H3 (H3- GST) was phosphorylated at Thr3 by Haspin (His tag) in an in vitro kinase assay, and H3pT3 level was examined by WB. (**F**) H3pT3 was dephosphorylated by purified GST fusion protein of SCP4 (GST-SCP4-WT), but not by the catalytically inactive mutant SCP4 (GST-SCP4-DN), in an in vitro phosphatase assay. Relative signal density ratios of H3pT3-GST to H3-GST were quantified using ImageJ (Version 1.54p) and are presented as arbitrary units. Experiments were performed three times independently (*n* = 3). Results from the first experiment are shown as representative data, with all individual data points presented. All raw data are provided in the Source Data File. Source data are available online for this figure.

H3T3 phosphorylation (H3pT3) is transient during mitosis. To examine its dynamics, we synchronized HeLa cells in prometaphase using nocodazole and monitored H3pT3 levels after release into normal medium. H3pT3 levels decreased sharply within one hour (Fig. 1A). To maintain high Cyclin B/CDK1 activity and arrest cells in metaphase, we treated nocodazole-released cells with the proteasomal inhibitor MG132 and the lysosomal inhibitor chloroquine (CQ), which prevent Cyclin B degradation. Previous studies (Vagnarelli et al, 2006; Vagnarelli et al, 2011; Wurzenberger et al, 2012) have shown that high Cyclin B/CDK1 activity inhibits Repo-Man/PP1γ. However, MG132 and CQ treatment did not prevent or slow the decline in H3pT3 levels (Fig. 1A), indicating that phosphatase(s) other than Repo-Man/PP1γ dephosphorylate H3pT3 even in the presence of high Cyclin B/CDK1 activity. Furthermore, the reduction in H3pT3 was not due to protein degradation, as inhibitors of proteasomal (MG132) and lysosomal (CQ) pathways failed to block the decline (Fig. 1A), supporting a primary role for phosphatase activity in regulating H3pT3 during mitosis.

**Figure EV1:**
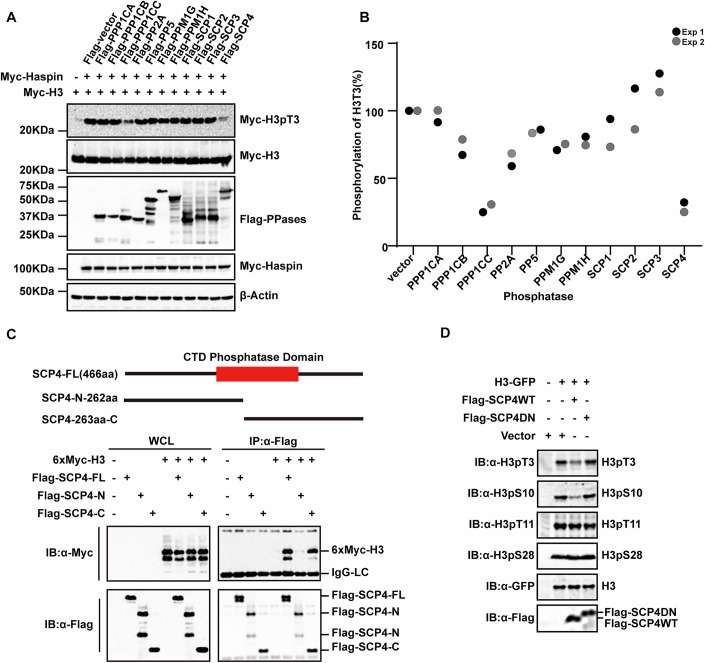
SCP4 reduced the phosphorylation level of H3 at the Thr3 site. (**A**) A representative panel of serine/threonine phosphatase library was screened to identify phosphatase(s) that reduce H3T3 phosphorylation, as assessed using phospho-T3- specific antibody. (**B**) Quantification of the ability of serine/threonine phosphatase family members to reduce H3T3 phosphorylation levels. The relative density ratio of H3pT3 to Myc-H3 levels was quantified using ImageJ (Version 1.54p). H3pT3 dephosphorylation results were obtained from two independent phosphatase screen assays (*n* = 2) and are presented as individual data points. (**C**) The C-terminal domain of SCP4 binds to H3. (**D**) SCP4 reduced the phosphorylation levels of T3 and S10, but not T11 and S28, in H3 in HEK293T cells. Source data are available online for this figure.

To identify phosphatases responsible for H3T3 dephosphorylation, we screened a phosphatase library containing 45 serine/threonine and dual-specificity phosphatases encoded in the human genome, which was constructed in our laboratory (Lin et al, 2006). Each phosphatase was co-transfected with H3 into HEK293T cells, and H3pT3 levels were assessed using H3pT3-specific antibody by western blotting. Representative results from the phosphatase family screen, including Protein Phosphatase 1, Protein Phosphatase 2, nuclear magnesium-dependent protein phosphatases, and C-terminal domain phosphatase family members, are shown in Fig. EV1A,B. Candidate phosphatases were further validated by overexpression and knockdown experiments. Among these, the nuclear phosphatase SCP4 significantly modulated H3pT3 levels. As shown in Fig. 1B, doxycycline (Dox)-inducible expression of wild-type SCP4 (SCP4-WT) in HeLa cells reduced H3pT3 levels, whereas a phosphatase-dead mutant (SCP4-DN, with a D295N substitution in the catalytic loop) had no effect, highlighting the essential role of phosphatase activity in SCP4 function. Conversely, Dox-induced shRNA-mediated SCP4 knockdown (shSCP4) elevated H3pT3 levels (Fig. 1C). We further analyzed the effect of aberrant SCP4 expression on H3pT3 dynamics during mitosis. A representative western blot analysis showed that, compared to control HeLa cells (shCtrl), shSCP4 cells released from G2/M arrest (by overnight treatment with RO-3306) exhibited delayed H3pT3 dephosphorylation, particularly in early metaphase (Fig. 1D). Notably, this phenotype distinguishes SCP4 from Repo-Man/PP1γ, which is associated with chromosome and regulates H3pT3 dephosphorylation late in mitosis (Qian et al, 2013; Qian et al, 2011; Vagnarelli et al, 2006; Vagnarelli et al, 2011; Wurzenberger et al, 2012). To rule out the possibility that SCP4 affects H3T3 phosphorylation indirectly through other phosphatase(s), we performed an in vitro phosphatase assay. H3 was purified as a GST fusion protein (H3-GST) and subsequently phosphorylated by active Haspin (Sigma-Aldrich) to produce H3pT3 (Fig. 1E). We found that purified GST-SCP4-WT could directly dephosphorylate H3pT3, whereas the SCP4-DN mutant lacked this ability (Fig. 1F). These results demonstrate that SCP4 is a bona fide H3T3 phosphatase acting during early mitosis.

**Figure 2:**
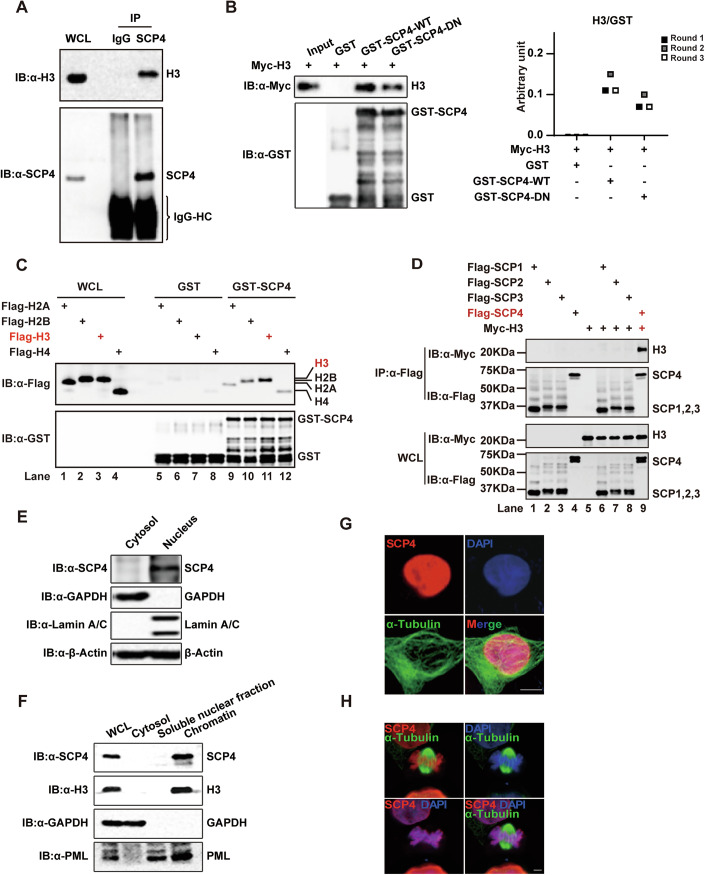
SCP4 is a chromatin-associated protein and interacts with H3 directly. (**A**) Complex formation between endogenous H3 and SCP4. HEK293T cell lysates were subjected to IP with anti-SCP4 antibody or rabbit IgG as a negative control. The IP products were examined by WB to detect the presence of H3. WCL: whole cell lysate. IgG-HC: heavy chain of IgG. (**B**) SCP4 binds to H3 directly in vitro. Purified GST only, GST-SCP4-WT, or GST-SCP4-DN proteins absorbed to glutathione sepharose beads were incubated with in vitro translated Myc-tagged H3. The pulled-down products were analyzed by WB to determine H3 and SCP4 association. Input: in vitro translated Myc-tagged H3. Comparison of binding affinity to H3 between GST-SCP4-WT and GST-SCP4-DN was performed using ImageJ (Version 1.54p). The relative density ratios of Myc-H3 to GST-SCP4-WT and GST-SCP4-DN levels were quantified and presented as arbitrary units. Assays were repeated three times independently (*n* = 3). Results from the first experiment are shown as representative data, with all individual data points presented. All raw data are provided in the Source Data File. (**C**) SCP4 has a higher binding affinity for H3 than for other histones. Purified GST only or GST-SCP4 fusion proteins were incubated separately with in vitro-translated, Flag-tagged histones (H2A, H2B, H3, and H4). SCP4-bound histones were analyzed by WB. WCL: whole cell lysates. (**D**) H3 binds to SCP4, but not other SCP family members. Flag-tagged SCP family members (SCP1, SCP2, SCP3, and SCP4) were co-transfected with Myc-tagged H3 individually in HEK293T cells. Cell lysates were subjected to Co-IP using anti-Flag-conjugated beads. H3 was only detected in the SCP4 IP products by WB. WCL: whole cell lysate. (**E**) Cytosol and nucleus fractionation assay of HEK293T cells indicated that SCP4 is a nuclear protein. GAPDH: marker for cytosol fraction; Lamin A/C: marker for nucleus fraction. β-Actin: internal control for WB. (**F**) HEK293T cell lysates were separated into cytosol, soluble nuclear fraction, and chromatin fraction. The subcellular location of SCP4 was determined by WB, indicating the chromatin localization. GAPDH: marker for cytosol fraction; PML (promyelocytic leukemia protein): marker for soluble nuclear and chromatin fractions. H3: marker for chromatin-associated protein. WCL whole cell lysate. (**G**) SCP4 is a nuclear protein as determined by IF in HeLa cells. α-tubulin: marker of the cytoskeleton. Scale bars: 5 μm. (**H**) IF staining of mitotic HeLa cells showed that SCP4 was localized on the chromosome. Scale bars: 5 μm. Source data are available online for this figure.

**Figure EV2:**
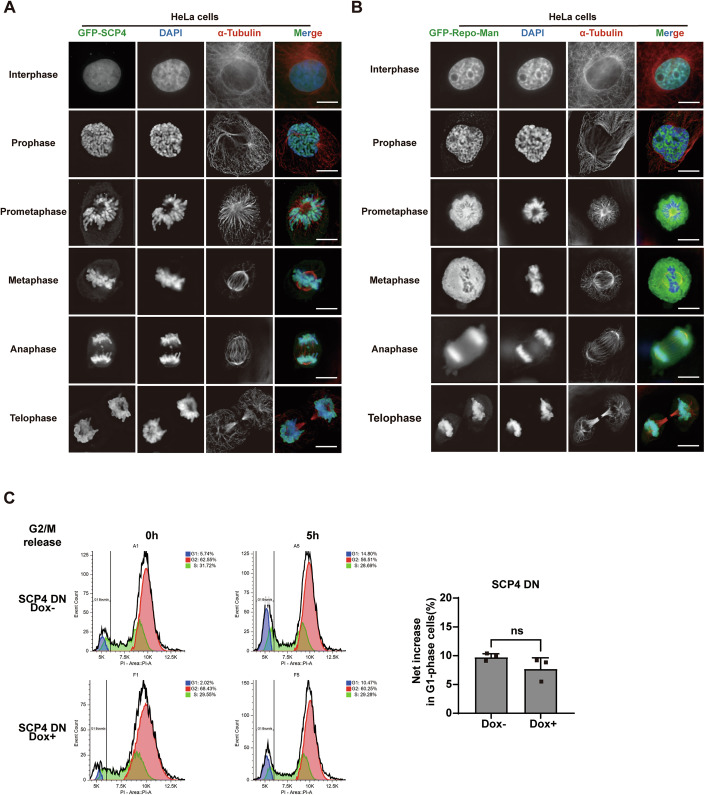
Distinct chromosomal localization patterns of SCP4 and Repo-Man during mitosis. (**A**,** B**) The chromosomal localization of GFP-SCP4 (**A**) and GFP-Repo-Man (**B**) in interphase, prophase, prometaphase, metaphase, anaphase, and telophase was determined by IF staining of HeLa cells. SCP4 remains associated with chromosomes throughout mitosis, while Repo-Man localizes to the nucleus in interphase, disperses from prophase to metaphase, and recruits to chromosomes at the onset of anaphase. Scale bars: 10 μm. (**C**) FACS analysis of cell cycle distribution showed that SCP4-DN expression had no significant effect on the progression from G2/M into G1 phase in HeLa cells after release from nocodazole-induced synchronization. The FACS data were analyzed using the Floreada.io software (version 3/30/2025) and presented as the mean percentage ± SD. The net increase in the percentage of G1 phase cells was calculated from three independent rounds, and statistical analysis was performed by using an unpaired, two-tailed Student’s *t*-test. ns, not significant (*P* = 0.1592). Source data are available online for this figure.

Consistent with its role as an H3T3 phosphatase, co-immunoprecipitation (Co-IP) experiments in HEK293T cells revealed that SCP4 physically interacts with H3 (Fig. 2A). The GST pull-down assay, utilizing purified GST-SCP4-WT or GST-SCP4-DN and in vitro-translated Myc-tagged H3 confirmed a direct interaction between the H3 and SCP4; SCP4-DN exhibited weaker binding affinity, possibly due to the substitution of the negatively charged aspartate D in SCP4-WT with the uncharged asparagine N in SCP4-DN (Fig. 2B). Domain-mapping experiments demonstrated that the C-terminal domain of SCP4 (amino acid 263 to the C-terminal end) mediates the interaction with H3 (Fig. EV1C). Since multiple sites in H3 undergo phosphorylation during mitosis, we tested SCP4 activity towards these sites and found that SCP4 exhibits activity towards H3T3 and H3S10 sites but not H3T11 or H3S28 (Fig. EV1D). Furthermore, SCP4 showed preferential binding to H3 over other nucleosomal histones, such as H2A, H2B, and H4 (lane 11) in an GST pull-down assay (Fig. 2C), and H3 displayed higher affinity for SCP4 than for other SCP family members (SCP1, SCP2, and SCP3) (lane 9) in a Co-IP experiment (Fig. 2D), underscoring the specificity of the SCP4-H3 interaction. Supporting its role in regulating H3T3 phosphorylation, subcellular fractionation and chromatin precipitation experiments in HEK293T cells confirmed that SCP4 is a chromatin-associated nuclear protein (Fig. 2E,F). Immunofluorescence (IF) staining of HeLa cells further demonstrated that SCP4 localizes to the nucleus during interphase (Fig. 2G) and associates with chromosomes during mitosis (Fig. 2H). These findings collectively establish SCP4 as a chromatin-associated phosphatase that directly binds and dephosphorylates H3pT3. Since Repo-Man has previously been reported to function as an H3T3 phosphatase, we compared the mitotic chromosomal localization of SCP4 and Repo-Man by IF staining and observed distinct localization patterns between the two proteins in HeLa cells. SCP4 remains associated with chromosomes throughout mitosis (Fig. EV2A). In contrast, Repo-Man localizes to the nucleus during interphase, disperses from prophase to metaphase, and then abruptly recruits to chromosomes at the onset of anaphase (Fig. EV2B). The dynamic localization of Repo-Man is coordinated with Cyclin B/CDK1 activity, which phosphorylates Repo-Man at multiple sites to regulate its chromosomal targeting (Qian et al, 2011; Vagnarelli et al, 2006; Vagnarelli et al, 2011; Wurzenberger et al, 2012).

### SCP4 modulates chromosomal recruitment of CPC during cell division

**Figure 3:**
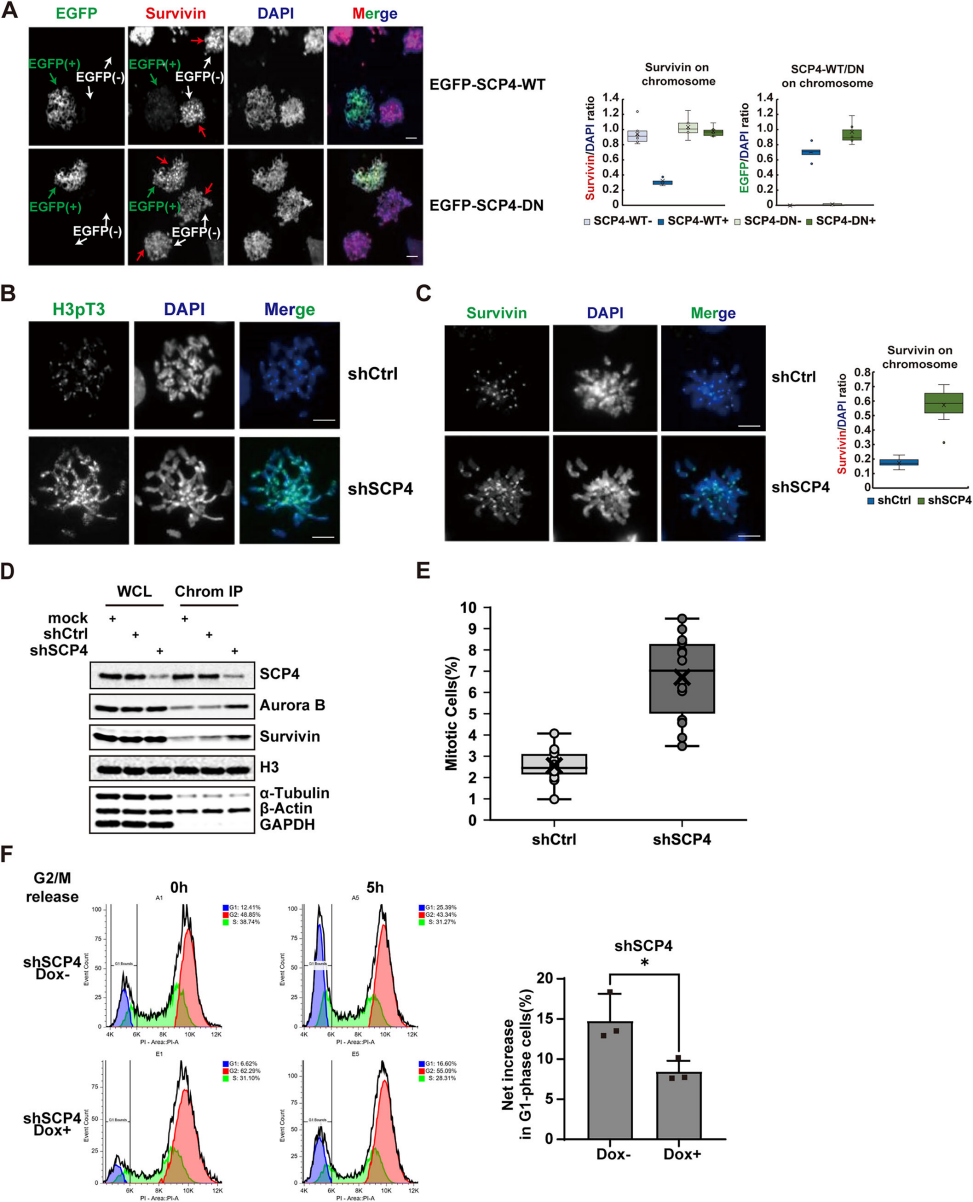
Aberrant expression of SCP4 disturbs the chromosomal recruitment of CPC. (**A**) Overexpression of SCP4 reduced Survivin recruitment to mitotic chromosomes. Expression of EGFP-tagged SCP4-WT (EGFP-SCP4-WT), but not the catalytically inactive EGFP-SCP4-DN mutant (green arrow), attenuated the chromatin localization of Survivin in mitotic HeLa cells (red arrow), compared with EGFP-negative cells (white arrow). Cells were synchronized with nocodazole, cytospun onto microscope slides, and immunostained for EGFP and Survivin. Images from the first of three independent transfections (*n* = 3) are presented as representative data. Relative fluorescence intensity ratios of EGFP and Survivin normalized to DAPI in each group were quantified using ImageJ (Version 1.54p) and are presented as arbitrary units. The Min, Max, median, the first percentile (Q1/bottom box), the third percentile (Q3/top box), and whiskers for the box plots are provided in the accompanying table in the Source Data File. Scale bars: 5 μm. (**B**,** C**) Knockdown of SCP4 enhanced the chromosomal localization of H3pT3 (**B**) and Survivin (**C**) in mitotic HeLa cells. Cells were synchronized with nocodazole, cytospun onto microscope slides, and immunostained for H3pT3 and Survivin. Images from the first of three independent transfections (*n* = 3) are presented as representative data. Relative fluorescence intensity ratios of Survivin normalized to DAPI were quantified using ImageJ (Version 1.54p) and are presented as arbitrary units. The Min, Max, median, the first percentile (Q1/bottom box), the third percentile (Q3/top box), and whiskers for the box plots are provided in the accompanying table within the Source Data File. Statistical analysis was performed using an unpaired, two-tailed Student’s *t*-tests. *P* = 1.60 × 10⁻^9^. shCtrl: control cells; shSCP4: SCP4 knockdown cells. Scale bars: 5 μm. (**D**) Increased chromosomal binding of CPC subunits, Aurora B and Survivin in SCP4 knockdown (shSCP4) HeLa cells. WCL (whole cell lysate) and its chromatin fraction were obtained, and chromatin-binding proteins were eluted with increasing concentration of salt. Mock: cells transfected with transfection reagents only; shCtrl: control cells; shSCP4: SCP4 knockdown cells. a-Tubulin, β-Actin, and GAPDH: internal controls for WB. (**E**) Increased percentage of mitotic cells in SCP4 knockdown (shSCP4) HCT116 cells as determined by mitotic index assay. Samples were collected from four independent transfections (*n* = 4), and fourteen pairs of measurements were performed. Data were presented as individual data points. The min, max, median, the first percentile (Q1/bottom box), the third percentile (Q3/top box), and whiskers for the box plots are provided in the accompanying table within the Source Data File. Statistical analysis was performed using an unpaired, two-tailed Student’s *t*-test. ***P = 1.00 × 10⁻⁶. (**F**) FACS analysis of cell cycle distribution showed that SCP4 knockdown delays progression from G2/M into G1 phase in HeLa cells after release from nocodazole-induced synchronization. The FACS data were analyzed and presented using the Floreada.io software (version 3/30/2025). Data were presented as the mean percentage ± SD from three independent experiments (*n* = 3). The net increase in the percentage of G1 phase cells was calculated from three independent rounds, and statistical analysis was performed by using an unpaired, two-tailed Student’s *t*-test. *P = 0.0400. Source data are available online for this figure.

During mitosis, CPC is recruited sequentially to chromosome arms and centromeres, a process critical for accurate chromatid segregation. This recruitment depends on H3T3 phosphorylation, which directly interacts with Survivin, a key CPC subunit (Higgins, 2010; Kelly et al, 2010; Wang et al, 2010; Yamagishi et al, 2010). Given that SCP4 dephosphorylates H3pT3, we hypothesized that aberrant SCP4 expression may affect CPC localization on chromosomes. Indeed, IF staining of HeLa cells transiently transfected with EGFP-SCP4-WT or -DN constructs revealed that EGFP-SCP4-WT expression reduced chromosomal recruitment of Survivin compared to EGFP-negative cells in prometaphase, whereas the EGFP-SCP4-DN mutant had no such effect (Fig. 3A, indicated by red arrows). Conversely, SCP4 knockdown increased H3pT3 levels on prometaphase chromosomes (Fig. 3B) and enhanced Survivin’s chromosomal localization (Fig. 3C). To further validate these findings, we performed chromatin-coated bead binding assays in HeLa cells, which demonstrated that SCP4 knockdown enhanced chromatin association of CPC subunits (Aurora B and Survivin) (Fig. 3D). Consistent with this, mitotic index analysis revealed that the proportion of mitotic phase cells increased significantly from 2.5% in control cells to 6.7% in SCP4 knockdown HCT116 cells (*P* = 1 × 10⁻^6^), indicating that SCP4 knockdown prolonged the mitotic phase (Fig. 3E). Furthermore, FACS analysis of cell cycle distribution showed that the net increase in the G1 population was reduced significantly from 14.7% in control cells to 8.5% in SCP4 knockdown cells (*P* = 0.0400), indicating delayed progression from G2/M into G1 in HeLa cells (Fig. 3F), whereas SCP4-DN had no significant effect on cell cycle progression (*P* = 0.1592) (Fig. EV2C).

### SCP4 influences chromosome behavior during metaphase and anaphase

**Figure 4:**
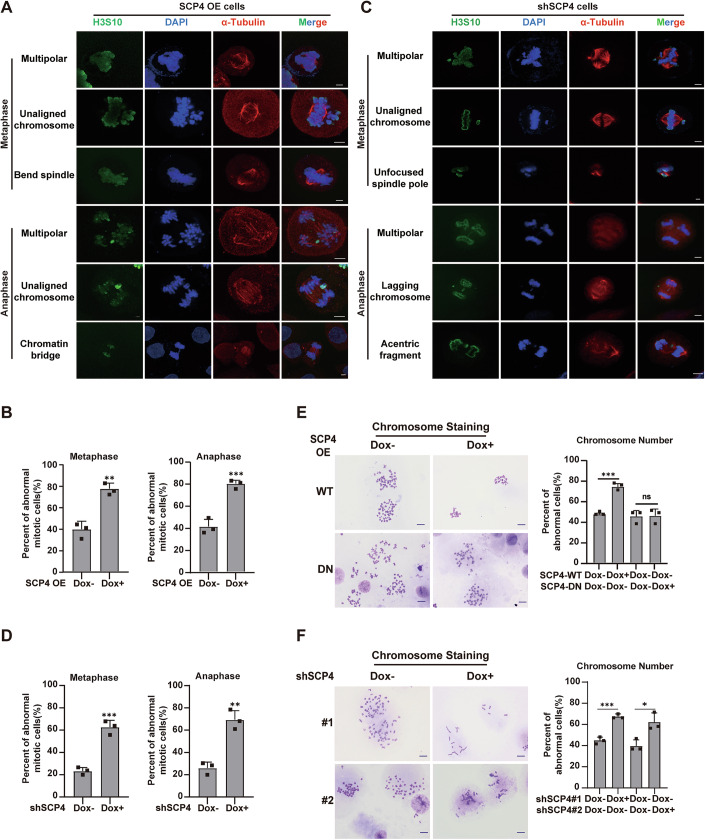
Aberrant expression of SCP4 leads to abnormal chromosome behavior during cell division and increased chromosomal instability. (**A**–**D**) SCP4 overexpression (SCP4 OE) (**A**,** B**) or knockdown (shSCP4) (**C**,** D**) increased the percentage of HeLa cells exhibiting mitotic defects during both metaphase and anaphase. Cells were stained with anti-phospho-histone H3 (Ser10) (P-H3S10) antibody to visualize chromosomes and anti-α-tubulin antibody to visualize the mitotic spindle. Mitotic defects observed in metaphase include multipolar spindles, unaligned chromosomes, bent spindles, and unfocused spindle poles, while anaphase defects include multipolar spindles, unaligned chromosomes, chromatin bridge, lagging chromosomes, and acentric fragment. Fifty cells per condition were analyzed, and the percentage of defective spindles in total mitotic cells was calculated. For bar graphs, data are presented as the mean percentage ± SD from three independent experiments (*n* = 3). Statistical significance was determined using an unpaired, two-tailed Student’s *t*-test. **P < 0.01; ***P < 0.001. For SCP4 overexpression: *P* = 0.0019 (metaphase) and *P* = 0.0007 (anaphase). For SCP4 knockdown: *P* = 0.0006 (metaphase) and *P* = 0.0015 (anaphase). Scale bars: 5 μm. (**E**,** F**) Mitotic chromosome spreads from control and Dox-induced SCP4-WT- or SCP4-DN-overexpressing (SCP4 OE) (**E**) or control and Dox-induced SCP4 knockdown (shSCP4) (**F**) HCT116 cells were prepared, stained with Giemsa solution, and the chromosome numbers counted in 50 mitotic cells per condition. Diploid cells: chromosome number *n* = 45; aneuploid cells: chromosome number *n* ≠ 45. For bar graphs, data are presented as the mean percentage ± SD from three independent experiments (*n* = 3). Statistical significance was determined using an unpaired, two-tailed Student’s *t*-test. *P < 0.05; ***P < 0.001; ns not significant. For SCP4 overexpression: *P* = 0.0001 (WT) and *P* = 0.9001 (DN). For SCP4 knockdown: *P* = 0.0005 (sh#1) and *P* = 0.0199 (sh#2). Scale bars: 10 μm. Source data are available online for this figure.

We next examined the effect of aberrant SCP4 expression on mitotic chromosome behavior in HeLa cells. Using H3pS10 (chromosome marker) and α-tubulin (spindle marker) staining, we found that SCP4 overexpression significantly increased mitotic abnormalities during both metaphase (including multipolar spindles, unaligned chromosomes, and bent spindles) and anaphase (including multipolar spindles, unaligned chromosomes, and chromatin bridge) (Fig. 4A). Quantification showed that the percentage of cells with mitotic defects increased from 38.0 ± 7.2% in control cells to 74.7 ± 5.0% in SCP4-overexpressing cells during metaphase, and from 40.7 ± 6.4% to 79.3 ± 3.1% during anaphase (Fig. 4B). Similarly, SCP4 knockdown also elevated the frequency of metaphase defects (including multipolar spindles, unaligned chromosomes, and unfocused spindle poles) and anaphase abnormalities (including multipolar spindles, lagging chromosomes, and acentric fragments) (Fig. 4C). Quantification revealed that the percentage of mitotically abnormal cells increased from 23.3 ± 3.1% in control cells to 62.7 ± 6.1% in SCP4 knockdown cells in metaphase, and from 26.0 ± 5.3% to 69.3 ± 8.1% in anaphase (Fig. 4D). These findings demonstrate that precise SCP4 expression is essential for faithful chromosome segregation. A summary of mitotic chromosome abnormalities is provided in Table EV1A.

**Figure EV3:**
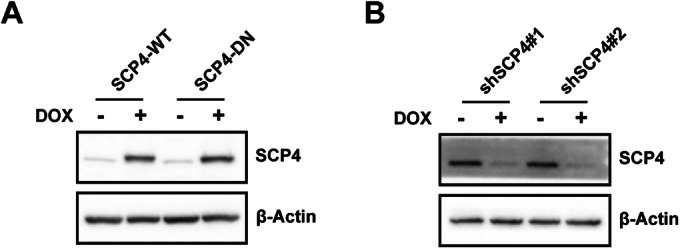
Generation of HCT116 and HeLa stable cell lines with SCP4 overexpression or knockdown, as well as H3T3 mutant. (**A**,** B**) Dox-inducible SCP4 overexpression (**A**) or knockdown by shSCP4 (**B**) was confirmed by WB. β-Actin: internal control for WB. Source data are available online for this figure.

Consistent with these mitotic defects, SCP4 dysregulation induces aneuploidy. For this analysis, we used the HCT116 colorectal cancer cell line, which typically maintains a relatively stable karyotype with 45 chromosomes, to generate stable cell lines with Dox-inducible SCP4 overexpression or knockdown (Lee et al, 2016; Mohr and Illmer, 2005; Thompson and Compton, 2008) (Fig. EV3A,B). SCP4-WT overexpression reduced diploid cells (45 chromosomes) from 53.3 ± 1.2% to 26.7 ± 3.1%, with 73.3 ± 3.1% cells becoming aneuploid (*n* ≠ 45) (Fig. 4E). The phosphatase-dead mutant SCP4-DN had no effect (44.7 ± 5.8% aneuploid in controls vs. 45.3 ± 6.4% aneuploid in SCP4-DN cells), confirming that SCP4’s catalytic activity is required for its role in mitotic regulation. Two independent shSCP4 constructs similarly increased aneuploid cells (shSCP4#1: 67.3 ± 2.3%; shSCP4#2: 62.0 ± 8.7%) compared to controls (44.7 ± 3.1 and 39.3 ± 5.8%; Fig. 4F). A summary of chromosome aneuploidy was listed in Table EV1B.

### SCP4 regulates chromosome dynamics and the first mitotic division via H3T3 phosphorylation

**Figure EV4:**
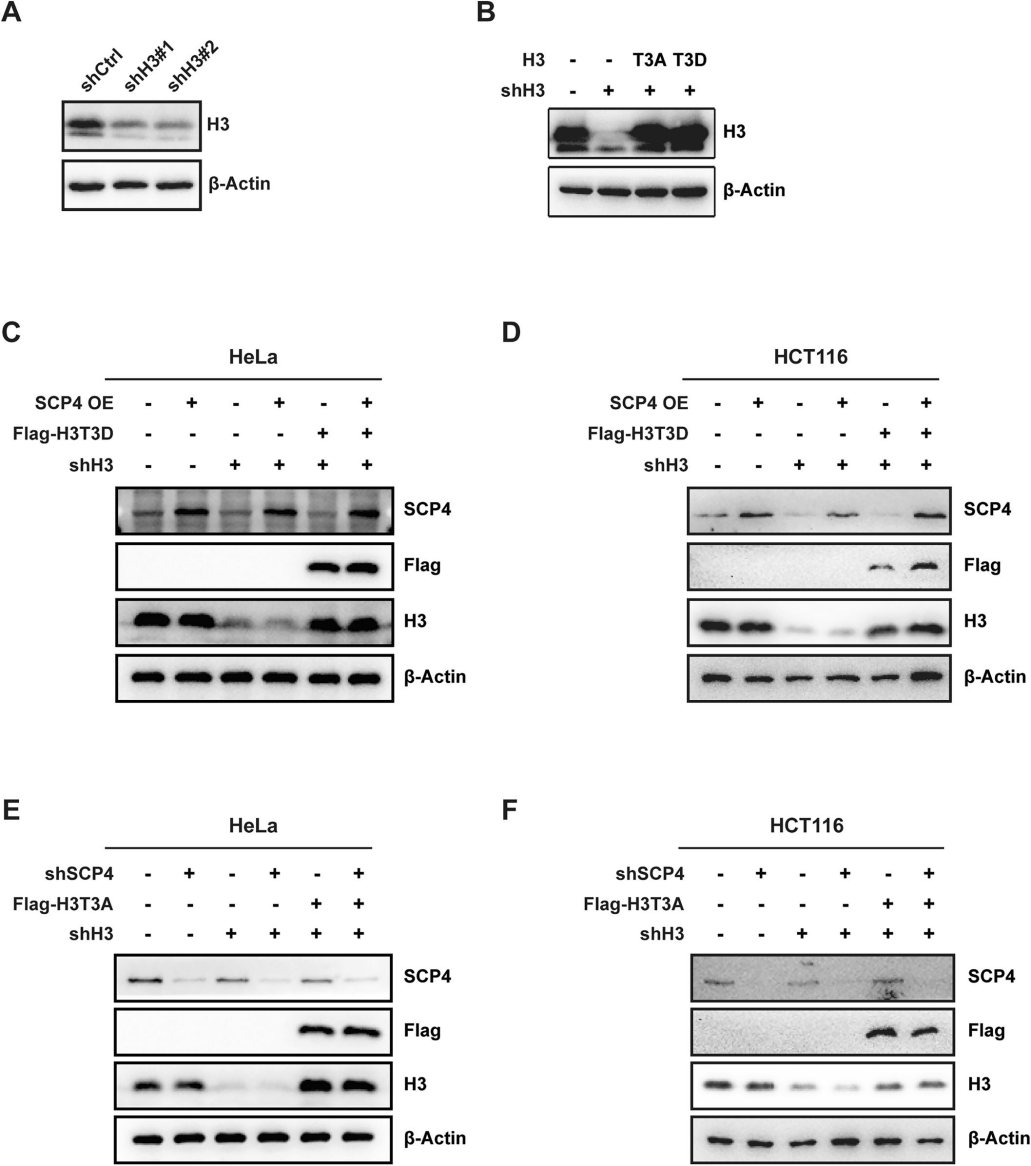
Stable HeLa and HCT116 cell lines were generated to express either a phospho-mimetic H3 mutant (H3T3D or Thr3Asp) together with SCP4 or a non-phosphorylatable H3 mutant (H3T3A or Thr3Ala) together with shSCP4, while endogenous H3 was simultaneously knocked down using shH3. (**A**) The knockdown efficiency of shH3#1 and shH3#2 was confirmed by WB in HeLa cells. (**B**) Expression of shH3-resistant H3T3D and H3T3A mutants was confirmed by WB in HeLa cells. (**C**,** D**) SCP4-overexpressing cells were established in H3T3D-expressing HeLa (**C**) and HCT116 cells (**D**). (**E**,** F**) SCP4-knockdown cells were established in H3T3A-expressing HeLa (**E**) and HCT116 cells (**F**). β-Actin: internal control for WB. Source data are available online for this figure.

**Figure 5:**
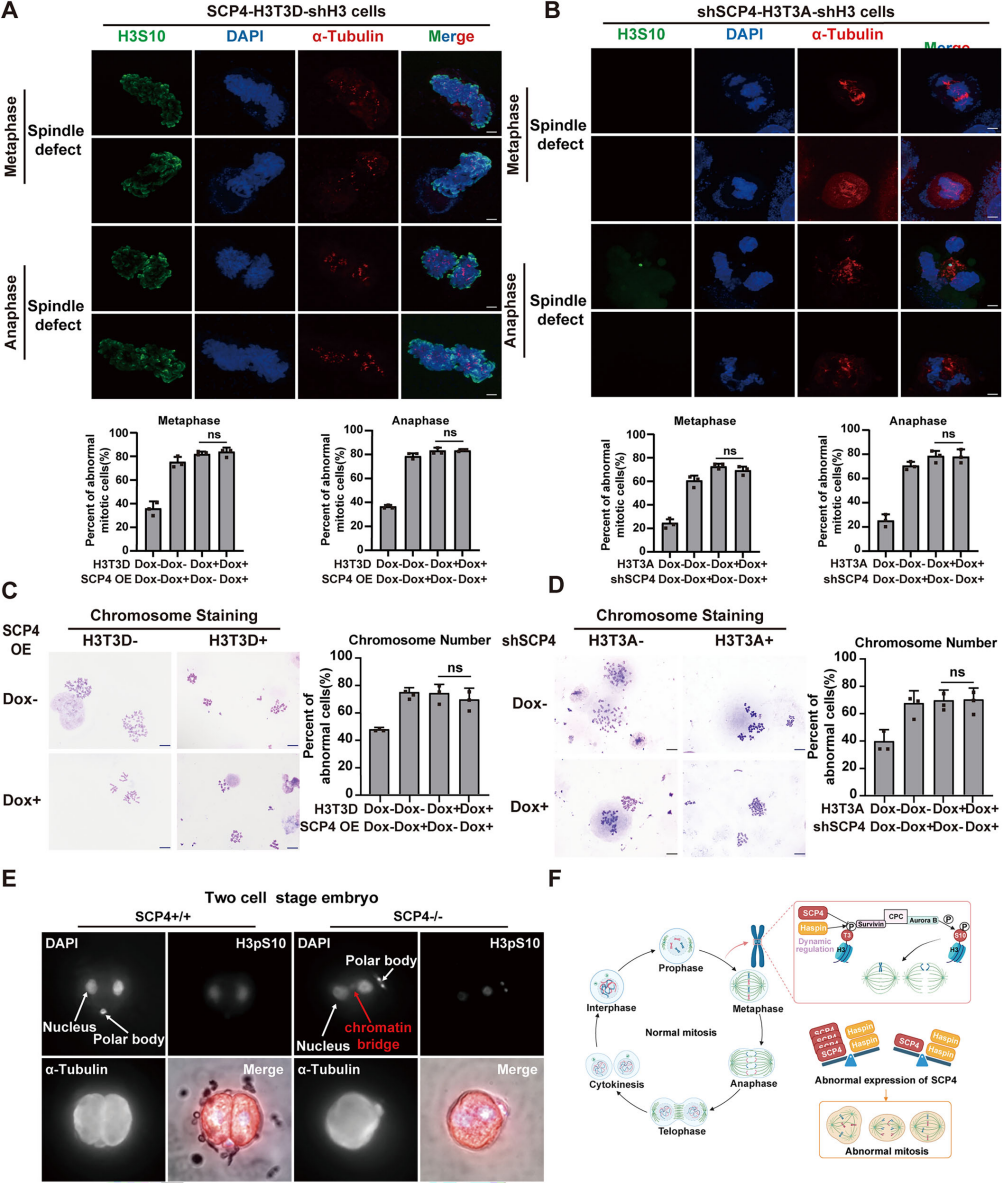
Aberrant H3T3 phosphorylation leads to mitotic defects and increased chromosomal instability. (**A**,** B**) Expression of the phospho-mimetic H3T3D (**A**) or non-phosphorylatable H3T3A (**B**) mutant induced mitotic defects during metaphase and anaphase in HeLa cells, which were not exacerbated by SCP4 overexpression (SCP4 OE) or knockdown (shSCP4). The percentages of mitotic cells with metaphase or anaphase defects were quantified and presented as a bar graph. Data were presented as the mean percentage ± SD from three independent experiments (*n* = 3). Statistical significance was determined using an unpaired, two-tailed Student’s *t*-test. ns, not significant. For H3T3D: *P* = 0.4353 (metaphase) and *P* > 0.9999 (anaphase). For H3T3A: *P* = 0.2062 (metaphase) and *P* = 0.8820 (anaphase). Scale bars: 5 μm. (**C**,** D**) Expression of H3T3D or H3T3A induced aneuploidy in HCT116 cells, which was not exacerbated by SCP4 overexpression (SCP4 OE) (**C**) or knockdown (shSCP4) (**D**). Chromosome numbers in 50 cells were analyzed, and the percentages of diploid cells (*n* = 45 chromosomes) and aneuploid cells (*n* ≠ 45 chromosomes) were presented as a bar graph. Data were presented as the mean percentage ± SD from three independent experiments (*n* = 3). Statistical significance was determined using an unpaired, two-tailed Student’s *t*-test. ns not significant (H3T3D: *P* = 0.4670; H3T3A: *P* = 0.9202). Scale bars: 10 μm. (**E**) Mitotic abnormalities during the first embryonic cleavage in Scp4 homozygous deletion mice. Representative IF images of two-cell stage zygotes from Scp4 homozygous deletion mice, showing abnormalities during the first mitotic division. Chromatin is visualized by P-H3S10 staining. Zygotes were stained with DAPI and α-tubulin. White arrows indicate the nucleus and polar body, while the red arrow indicates a chromatin bridge. Images were acquired at 40× magnification. (**F**) Working model: SCP4 dephosphorylates histone H3 at T3 to ensure accurate chromosome dynamics during mitosis. Dysregulation of SCP4 activity results in mitotic abnormalities. Source data are available online for this figure.

To verify that SCP4 exerts its effects via H3T3 phosphorylation, we generated stable HeLa and HCT116 cell lines expressing either a phospho-mimetic (H3T3D, Thr3 to Asp) or non-phosphorylatable (H3T3A, Thr3 to Ala) H3 mutant. Both mutants were designed to be refractory to aberrant SCP4 expression, while endogenous histone H3 was concurrently knocked down using H3-specific shRNAs (Fig. EV4A–F). Both mutants induced severe mitotic defects, and importantly, neither SCP4 overexpression nor knockdown exacerbated these phenotypes. Specifically, in phospho-mimetic H3T3D-expressing HeLa cells, 82.0 ± 2.0% of metaphase cells and 83.3 ± 2.3% of anaphase cells exhibited spindle and chromosome segregation defects, compared to 36.0 ± 6.0% (metaphase) and 36.7 ± 1.2% (anaphase) in controls (Fig. 5A). While SCP4 overexpression increased defect rates in control cells (75.3 ± 4.2% metaphase, 78.7 ± 2.3% anaphase), it had no additional effect on H3T3D cells (84.0 ± 3.5% metaphase, 83.3 ± 1.2% anaphase), consistent with the inability of SCP4 to dephosphorylate this phospho-mimetic mutant (Fig. 5A). Similarly, non-phosphorylatable H3T3A-expressing HeLa cells displayed high baseline defect rates (72.7 ± 2.3% metaphase, 78.7 ± 4.2% anaphase vs. 24.7 ± 3.1% and 25.3 ± 5.0% in controls). SCP4 knockdown increased abnormalities in controls (60.7 ± 4.2% metaphase, 70.7 ± 3.1% anaphase) but not in H3T3A cells (69.3 ± 3.1% metaphase, 78.0 ± 6.0% anaphase) (Fig. 5B), confirming that SCP4 cannot modulate phosphorylation of the non-phosphorylatable mutant. A summary of mitotic chromosome abnormalities is provided in Table EV1A.

Consistent with these mitotic defects, aneuploidy analysis in HCT116 cells reinforced these findings. Both H3T3D (72.7 ± 6.1% aneuploidy) and H3T3A (68.0 ± 7.2%) cells exhibited elevated aneuploidy (*n* ≠ 45), but neither SCP4 overexpression (Fig. 5C, H3T3D: 68.0 ± 8.0%) nor knockdown (Fig. 5D, H3T3A: 68.7 ± 8.1%) further increased these rates. In contrast, control cells showed significant aneuploidy upon SCP4 perturbation (Fig. 5C, overexpression: 46.7 ± 1.2% to 73.3 ± 3.1%; Fig. 5D knockdown: 38.7 ± 8.1% to 66.0 ± 8.7%). A summary of chromosome aneuploidy was listed in Table EV1B.

**Figure EV5:**
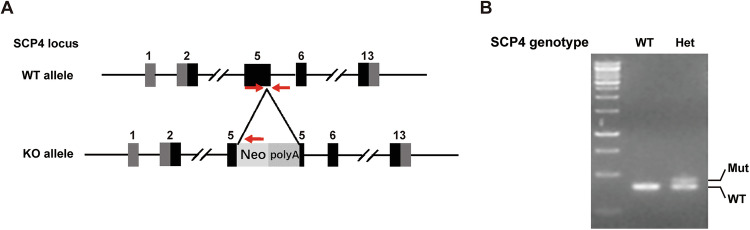
Generation of Scp4 gene knockout mice. (**A**) Diagram illustrating the strategy used to generate Scp4 knockout mice. Red arrows indicate the position of primers used for genotyping. (**B**) Genotyping of Scp4 knockout mice by PCR. Het heterozygous mouse, Mut Scp4 mutant allele.

Moreover, to assess the physiological role of SCP4 in ensuring accurate cell division in vivo, we generated *Scp4* knockout mice (Fig. EV5A,B). Homozygous deletion caused embryonic lethality. DAPI and α-tubulin staining of zygotes from a heterozygous mouse cross revealed mitotic abnormalities (chromatin bridges) in 3 out of 11 zygotes during the first cleavage at the two-cell stage (Fig. 5E), whereas no abnormalities were observed in control crosses. Although zygote genotyping was not performed, the frequency of defects aligns with expected Mendelian ratios, strongly implicating that SCP4 loss causes these division errors. These findings demonstrate that SCP4 ensures mitotic fidelity primarily by regulating H3T3 phosphorylation, playing essential roles in chromosome segregation and early embryonic development.

In this study, we identified the nuclear phosphatase SCP4 as an H3 Thr3 phosphatase that regulates H3T3 phosphorylation. SCP4 modulates the dephosphorylation of H3T3 during mitosis and controls the chromosomal recruitment of the CPC. Functional analyses demonstrate that SCP4 is essential for faithful cell division, as its dysregulation results in severe mitotic abnormalities, including aberrant spindle formation, chromosome misalignment in metaphase, lagging chromosomes, multipolar spindles in anaphase, and aneuploidy (see working model, Fig. 5F). Future investigations are warranted to elucidate the upstream regulatory mechanisms governing SCP4 activity during mitosis. Additionally, it will be important to determine whether SCP4 functionally cooperates with, or counteracts, Haspin kinase and other phosphatases to maintain balanced H3T3 phosphorylation levels.

## Methods

**Table:** Reagents and tools table

Reagent/resource	Reference or source	Identifier or catalog number	
**Cell lines**			
HCT116 (*H. sapiens*)	ATCC	CCL-247EMT	
HEK-293 cells (*H. sapiens*)	ATCC	CRL-1573	
HeLa (*H. sapiens*)	ATCC	CCL-2	
**Antibodies**	**Source**	**Catalog number**	**Dilution ratio**
Anti-Aurora B	Cell Signaling Technology	28711	1:1000
Anti-CTDSPL2 (SCP4)	Cell Signaling Technology	6932	1:500
Anti-Flag-Tag	Sigma-Aldrich.	F1804	1:500
Anti-GAPDH	Abclonal (CHN).	AC033	1:100000
Anti-GFP	Cell Signaling Technology	2555	1:1000
Anti-GST-Tag	Cell Signaling Technology	2625	1:500
Anti-His-Tag	Cell Signaling Technology	2365	1:1000
Anti-Histone H3	Cell Signaling Technology	4499	1:3000
Anti-Lamin A/C	Abclonal (CHN).	A19524	1:50000
Anti-Myc-Tag	Cell Signaling Technology	2276	1:1000
Anti-phospho-Histone H3 (Ser10)	Cell Signaling Technology	53348	1:500
Anti-phospho-Histone H3 (Ser28)	Cell Signaling Technology	9713	1:1000
Anti-phospho-Histone H3 (Thr3)	Cell Signaling Technology	13576	1:1000
Anti-phospho-Histone H3 (Thr11)	Cell Signaling Technology	9764	1:1000
Anti-PML	Cell Signaling Technology	33156	1:1000
Anti-Survivin	Cell Signaling Technology	2808	1:1000
Anti-α-Tubulin	Abclonal (CHN).	AC012	1:10000
Anti-β-Actin	Abclonal (CHN).	AC026	1:100000
**Oligonucleotide sequence**			
H3T3A	atggcccgaGCCaagcagact		
H3T3D	atggcccgaGACaagcagact		
SCP4 KO genotyping primer 1 (forward)	5’-GCAGTTCAAGTGAGGCCATC-3’		
SCP4 KO genotyping primer 2 (reverse, WT)	5’-GGGGAGTGTATTGGGGACTT-3’		
SCP4 KO genotyping primer 3 (reverse, Mut)	5’-CCTCGTCCTGCAGTTCATTC-3’		
shH3# 1	GCTGTTTGAGGACACCAACCTGTGT		
shH3# 2	TGCTAATCCGCAAGTTGCCCTTCCA		
shSCP4# 1	GGAAACCTGCTCTTCCGTT		
shSCP4# 2	GCCTTTGCATATCAGCTTTC		
wild-type H3T3	atggcccgaACCaagcagact		
**Chemicals, enzymes and other reagents**	**Source**	**Catalog Number**	
Alexa Fluor 488	Invitrogen	A-11094	
BamHI-HF	NEB	R3136	
biotin-dATP	Invitrogen	19524016	
biotin-dUTP	Invitrogen	R0081	
Blasticidin	Sigma-Aldrich	203350	
BSA	Beyotime (CHN)	ST023	
Cell Cycle Detection Kit	Vazyme (CHN)	AC101	
Chloroquine	Sigma-Aldrich	ST9H9BC1508D	
DAPI	Biosharp (CHN)	BL105A	
DMEM	Hyclone	SH30243.01	
DNA Polymerase I, Large (Klenow) Fragment	NEB	M0210	
Doxycycline	Sigma-Aldrich	1225984	
EDTA	Solarbio (CHN)	E1170	
EGTA	Solarbio (CHN)	E8053	
FBS	Vazyme (CHN)	F101-01	
Giemsa solution	HK-bio	HK 2025-1	
Glutathione Sepharose 4B beads	Thermo Scientific Pierce	16100	
Haspin, active (His tag)	Sigma-Aldrich	14-744-M	
HEPES	Beyotime (CHN)	C0217	
Hygromycin	Sigma-Aldrich	10843555001	
IgG Isotype Control	Cell Signaling Technology	3900	
KCl	Macklin (CHN)	P816348	
LipoGene 2000 Plus	UElandy (CHN)	L7003 BSA	
MG132	Sigma-Aldrich	M8699	
MgCl_2_	DAWEN (CHN)	DW269	
NaCl	Macklin (CHN)	s805275	
Nocodozole	Sigma-Aldrich	SML1665	
NotI-HF	NEB	R3189	
PFA	Beyotime (CHN)	P0099	
Phosphatase Inhibitor Cocktail I	MedChemExpress	HY-K0021	
ProLong Gold mounting medium containing DAPI	Invitrogen	P36931	
Protease Inhibitor Cocktail	MedChemExpress	HY-K0010	
Protein A/G Magnetic Beads	Vazyme (CHN)	PB101-01	
Puromycin	Sigma-Aldrich	P9620	
RO-3306	Sigma-Aldrich	SML0569	
Streptavidin M280 Dynabeads	Invitrogen	11205D	
Sucrose	Biosharp (CHN)	BS085	
Taxol	Sigma-Aldrich	T7402	
Thio-dCTP	APExBIO	B8104	
Thio-dGTP	MedChemExpress	178036-66-3	
Thymidine	Beyotime (CHN)	ST1704	
TnT® Quick Coupled Transcription/Translation System	Promega	L1170	
Tris-HCl	Solarbio (CHN)	T1140	
**Software**			
GraphPad Prism 8	https://www.graphpad.com		
**Other**			
laser scanning confocal microscope	Zeiss (GER)	LSM900	
Upright microscope	Olympus	BX53	

### Plasmids and shRNA constructs

The cDNAs encoding SCP4-WT and a catalytically inactive SCP4 mutant (SCP4-DN, Asp295Asn substitution) were cloned into the mammalian expression vectors pXF6F and pEGFP-XF6F (derivatives of pRK5, Genentech), as well as into the lentiviral vector pWPI-Tet-on-puromycin (Addgene). The cDNAs encoding SCP1, SCP2, and SCP3 were also cloned into the mammalian expression vector pXF6F.

The cDNA encoding shSCP4 was cloned into the mammalian expression vector pLKO.1-puromycin and the lentiviral vector pLKO.1-Tet-on-puromycin (Addgene). The shSCP4 target sequences were #1: GGAAACCTGCTCTTCCGTT and #2: GCCTTTGCATATCAGCTTTC.

The cDNA encoding H3 was cloned into the mammalian expression vector pXF6M (derivative of pRK5, Genentech). Phospho-mimetic (H3T3D or Thr3Asp) and non-phosphorylatable (H3T3A or Thr3Ala) H3 mutants were generated using the Mut Express MultiS Fast Mutagenesis Kit (Vazyme, CHN) and cloned into the lentiviral vector pBobi-hygromycin (Addgene). The sequences of wild-type H3T3 and its mutants were as follows: wild-type H3T3, atggcccgaACCaagcagact; H3T3D, atggcccgaGACaagcagact; H3T3A, atggcccgaGCCaagcagact. Both mutants were engineered to be resistant to shH3 through synonymous mutations in the shH3 target sequence.

The cDNA encoding shH3 was cloned into the lentiviral vector pLKO.1-Blasticidin (Addgene). The shH3 target sequences were #1: GCTGTTTGAGGACACCAACCTGTGT and #2: TGCTAATCCGCAAGTTGCCCTTCCA.

### Cell culture, cell transfection, lentivirus production, and stable cell line generation

HEK293T cells, HeLa, and HCT116 cells were cultured in DMEM (Hyclone) supplemented with 10% FBS (Vazyme, CHN) and 1% Penicillin/Streptomycin. Cell transfection was performed using LipoGene 2000 Plus (UElandy, CHN) according to the manufacturer’s instructions. Lentivirus was produced by co-transfecting HEK293T cells with the packaging plasmid psPAX2, the envelope plasmid pMD2.G, and the lentiviral plasmid. Stable cells were generated through appropriate drug selection. Inducible expression of target genes or shRNA was under the control of doxycycline (Dox)-inducible promoter. Stable cell lines expressing H3T3D or H3T3A were established in H3-knockdown HeLa and HCT116 cells through hygromycin selection.

### Immunoprecipitation (IP), Western blot (WB), and immunofluorescence (IF) staining

IP and WB were performed as previously described (Zhao et al, 2014). For IF staining, cells were fixed in 4% paraformaldehyde for 10 min. After blocking with 4% BSA for 1 h, cells were incubated with the primary antibody for 1 h at room temperature, followed by incubation with the appropriate fluorescently labeled secondary antibodies. The cells were then mounted with ProLong Gold mounting medium containing DAPI (Invitrogen). Immunofluorescence images were captured using a laser scanning confocal microscope (LSM900, Zeiss, GER).

### GST-fusion protein production and in vitro binding assay

To determine the direct interaction between SCP4 and H3, SCP4 (WT or DN) was cloned into the pGEX vector (Millipore) for GST-fusion protein production. Purified GST-SCP4 protein, absorbed onto Glutathione Sepharose 4B beads, was incubated with in vitro-translated Myc-tagged H3 (TnT® Quick Coupled Transcription/Translation System, Promega) at 4 °C for 2 h in binding buffer (0.1% Nonidet P-40, 120 mM NaCl, 50 mM Tris-HCl, pH 7.5, and 2 mM EDTA). H3 association with SCP4 was assessed by WB. To compare the association of SCP4 with histone family members (H2A, H2B, H3, and H4), purified GST-SCP4 protein-beads were subjected to an in vitro binding assay with in vitro-translated Flag-tagged histone proteins as described above. Histone association with SCP4 was assessed by WB.

### Subcellular fractionation

Cell pellets were suspended in buffer A (250 mM sucrose, 20 mM HEPES, pH 7.4, 10 mM KCl, 1.5 mM MgCl_2_, and 1 mM EDTA) and incubated for 10 min. The nuclear pellet was collected by centrifugation at 1200 × *g* for 5 min, with the supernatant representing the cytoplasmic fraction. The nuclear pellet was washed twice with buffer A and then resuspended in buffer B (20 mM HEPES, pH 7.9, 1.5 mM MgCl2, 0.2 mM EDTA, and 0.5 M NaCl) at 4 °C for 30 min, followed by a high-speed centrifugation at 10,000 × *g* for 15 min. The supernatant was used as the soluble nuclear fraction, while the pellet contained the chromatin fraction.

### Cell synchronization, chromosome staining, and chromosome number counting

To obtain metaphase cell lysates, cells were treated with Nocodazole (50 ng/ml) for 16–20 h, collected, and lysed in RIPA buffer. To stop cells at G2/M phase of the cell cycle, cells were treated with RO-3306 (5 μM) for 16–20 h. To enrich cells at metaphase or anaphase for mitotic chromosome staining, cells were synchronized by culturing in medium containing thymidine (4 μM) for 24 h, followed by culture in regular medium for 9 h. Then, cells were treated with nocodazole (40 ng/ml) for 4 h. The cells were released from nocodazole arrest for 30–40 min (metaphase) and 120–130 min (anaphase) before sample collection. Mitotic chromosomes were cytospun onto Superfrost Plus microscope slides at 120 × *g* for 5 min and immunostained with the indicated antibodies. For chromosome number counting, cells were treated with nocodazole (100 ng/ml) for 16–20 h, collected, and centrifuged at 500 × *g* for 5 min. The cell pellet was resuspended in prewarmed 75 mM KCl at 37 °C for 15 min and fixed with methanol/acetic acid (3:1) at 4 °C for 30 min, followed by centrifugation at 500 × *g* for 5 min to collect the pellet. The pellet was resuspended in methanol/acetic acid (3:1) and dropped onto a cold slide for chromosome spreading. The slide was stained with Giemsa solution (HK-Bio, HK) and examined under a microscope. Data were collected from 50 cells for each condition.

### Chromatin-coated bead binding assay

Chromatin-coated bead binding assays were used to identify chromatin-binding proteins. DNA-coated beads were first prepared by generating a 3–5 kb DNA fragment via NotI and BamHI digestion of the pBluescript SK (+) vector. The overhangs of this fragment were filled in with the Klenow fragment of DNA polymerase I in the presence of biotin-dATP, biotin-dUTP, Thio-dCTP, and Thio-dGTP. The biotin-labeled linear DNA was then bound to streptavidin M280 Dynabeads (Invitrogen) at a concentration of 1.8 μg DNA/μl Dynabeads slurry, washed three times with binding solution (3% Polyvinyl Alcohol, 50 mM Tris-HCl, pH 8.0, 2 mM 0.5 M EDTA, and 1 M NaCl), and stored at 4 °C as DNA-coated beads. Next, mitotic spindles were isolated. Briefly, cells were synchronized at metaphase with 5 μg/ml Taxol and harvested by centrifugation at 300 × *g* for 1 min. The cells were resuspended in lysis buffer (100 mM PIPES, pH 6.9, 1 mM MgSO_4_, 2 mM EGTA, 0.5% NP-40, 20 mM β-glycerol phosphate, with phosphatase and proteinase inhibitors, and Taxol) and incubated for 15 min at 37 °C. Mitotic spindles were collected by centrifugation at 700 × *g* for 2 min and resuspended in lysis buffer. The mitotic spindles were then washed with low-ionic-strength buffer (1 mM PIPES, pH 6.9, with inhibitors) and resuspended in the same buffer. To identify chromatin-binding proteins, the DNA-coated beads were washed with low-ionic strength buffer (1 mM Pipes, pH 6.9) and added to the mitotic spindle preparation. The mitotic spindle/DNA-beads mixture was incubated at RT for 2 h, and the precipitated beads were washed three times with washing buffer (10 mM HEPES, pH 7.7, 50 mM NaCl, 1.5 mM MgCl_2,_ 0.5 mM EGTA, 50 mM Sucrose, 0.1 mM DTT, and 0.05% NP-40). Chromatin-binding proteins were eluted from the beads by incubating in washing buffer with a NaCl concentration gradient, nutating at room temperature for 10 min. Histones were eluted using 2 M NaCl. All eluted proteins were examined by WB.

### Mitotic index assay

HCT116 cells expressing shCtrl or shSCP4 were seeded on coverslips and cultured in regular medium for 24 h. Cells were then fixed in 4% paraformaldehyde for 10 min, permeabilized with PBS-T (PBS with 0.2% Tween-20), and subjected to IF staining using an anti-H3pS10 antibody followed by an Alexa Fluor 488-conjugated anti-rabbit secondary antibody. After nuclear counterstaining with DAPI, cells were examined under a Leica microscope. Fluorescent images were quantified, and the mitotic index was calculated as the percentage of H3pS10-positive (mitotic) among all DAPI-positive cells. Statistical analysis was performed using an unpaired, two-tailed Student’s *t-*test.

### FACS analysis

To investigate cell cycle progression from G2/M into G1 phase, we employed a sequential thymidine-nocodazole block in HeLa cells. Cells were first arrested at the G1/S boundary by treatment with 2 mM thymidine for 18 h. After thorough washing with PBS, cells were released into fresh medium for 9 h, followed by incubation with 100 ng/mL nocodazole for 4 h to arrest them in G2/M phase. Cells were then either collected immediately (0 h time point) or washed, replated in fresh medium, and harvested after 5 h (5 h time point). For FACS analysis, cells were fixed in 70% ice-cold ethanol, stained with Propidium Iodide (PI) and RNase A, and analyzed for DNA content by flow cytometry. The FACS data were analyzed and presented using the Floreada.io software (version 3/30/2025).

### In vitro kinase and phosphatase assay

Purified C-terminal tagged GST-H3 fusion protein was phosphorylated in vitro by active Haspin protein in kinase reaction buffer (20 mM Hepes, pH 7.5, 5 mM MgCl_2_, 1 mM DTT, and 0.1 mM ATP) at 30 °C for 30 min. Successful phosphorylation of H3 at Thr3 (H3pT3) was confirmed by WB using an anti-phospho-H3 (Thr3) antibody. Subsequently, H3pT3 was subjected to an in vitro phosphatase assay with purified GST-SCP4 fusion protein as the phosphatase in phosphatase reaction buffer (40 mM Tris-HCl, pH 8.0, 20 mM KCl, 30 mM MgCl_2_, and 2 mM dithiothreitol) at 37 °C for 1 h. Dephosphorylation of H3pT3 was analyzed by WB.

### Husbandry care for mice

Mice were housed in groups of 2–4 per cage under specific pathogen-free (SPF) conditions in a temperature-controlled room maintained at 22 ± 2 °C with a relative humidity of 50–60% and a 12:12 h light/dark cycle. They had ad libitum access to standard rodent chow and filtered tap water. All animals were acclimatized to the facility for at least 1 week prior to the start of experiments. Cage changes and general husbandry procedures were performed weekly. All procedures were conducted in accordance with institutional animal care guidelines and approved by the local ethics committee.

### Generation of *Scp4* knockout mice and two-cell-stage embryo staining

*Scp4* knockout mice were generated by inserting a neomycin resistance gene containing a stop codon into exon 5 of the *Scp4* genomic DNA. Genotyping was performed by PCR using the following primers:

Primer 1 (forward): 5-GCAGTTCAAGTGAGGCCATC-3

Primer 2 (reverse): 5-GGGGAGTGTATTGGGGACTT-3; for WT allele

Primer 3 (reverse): 5-CCTCGTCCTGCAGTTCATTC-3; for mutant allele

To examine the effect of *Scp4* knockout on early embryo development, *Scp4* heterozygous mice were mated with either *Scp4* heterozygous or wild-type mice. Zygotes at the two-cell stage were harvested, fixed in 4% paraformaldehyde for 30 min, permeabilized with 0.1% Triton X-100 for 5 min, and stained with DAPI and anti-α-tubulin to visualize the first cleavage of the zygote under a fluorescence microscope.

### Blinding

While group assignment of mice was based on genotype, all subsequent phenotypic assessments and data analyses were performed by investigators blinded to the mice’s genetic status. Group codes were revealed only after all data had been acquired and analyzed.

### Animal study approval

The animal studies were approved by the Animal Experimental Ethical Inspection of the First Affiliated Hospital, Zhejiang University School of Medicine, under institutional approval number 1744.

### Statistical analysis

Data were presented as indicated in the figure legends. For quantitative data (e.g., bar graphs), results are shown as means ± standard deviation (S.D.) from three independent experiments (*n* = 3). Comparisons between two groups were performed using an unpaired, two-tailed Student’s *t-*test (*P < 0.05; **P < 0.01; ***P < 0.001; ns not significant). For categorical data (e.g., tables), results from three independent experiments were pooled and analyzed using the Chi-square test, with *P* < 0.05 considered significant (ns not significant). All statistical analyses were conducted using GraphPad Prism software.

## Supplementary information

Table EV1

Peer Review File

Source data Fig. 1

Source data Fig. 2

Source data Fig. 3

Source data Fig. 4

Source data Fig. 5

Figure EV1 Source Data

Figure EV2 Source Data

Figure EV3 Source Data

Figure EV4 Source Data

Expanded View Figures

## Data Availability

We have not deposited any data in public databases. All raw data for the figures are provided in the Source Data File. The source data of this paper are collected in the following database record: biostudies:S-SCDT-10_1038-S44319-026-00833-1.
