# Supplementary figures and images for: SCP4 dephosphorylates mitotic histone H3 to maintain chromosome stability (part 1 of 2)
Source: EMBO Rep. 2026 Jun 19;27(14):3944–63. doi: 10.1038/s44319-026-00833-1 (PMC13400628; doi:10.1038/s44319-026-00833-1)

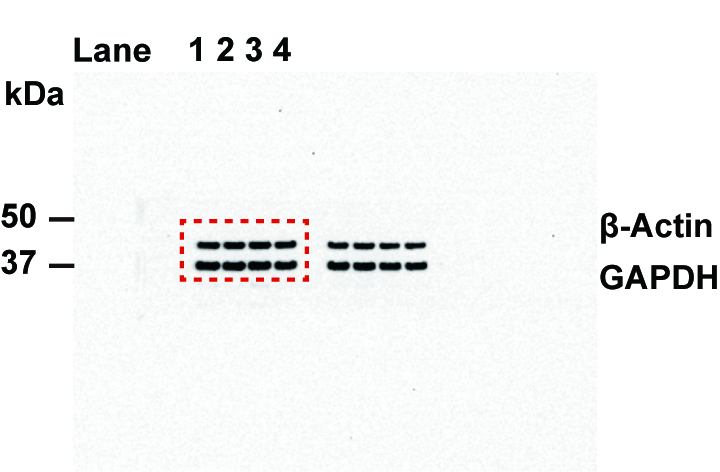

Supplement: Supplementary file 3 — Source data Fig. 1 [file 44319_2026_833_MOESM3_ESM.zip › 1A/Actin GAPDH/Origin 1A Actin GAPDH.tif]

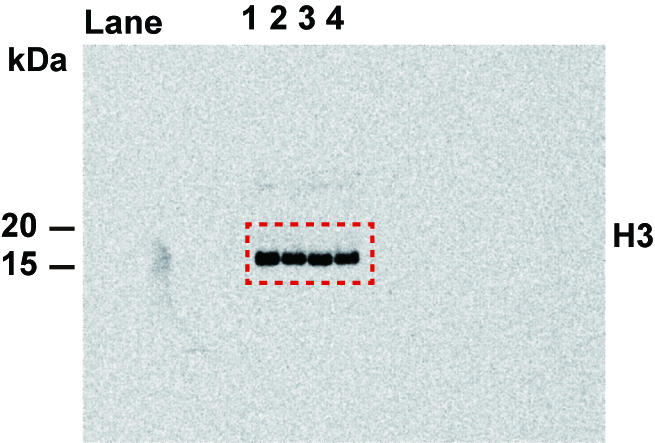

Supplement: Supplementary file 3 — Source data Fig. 1 [file 44319_2026_833_MOESM3_ESM.zip › 1A/H3/Origin 1A H3.tif]

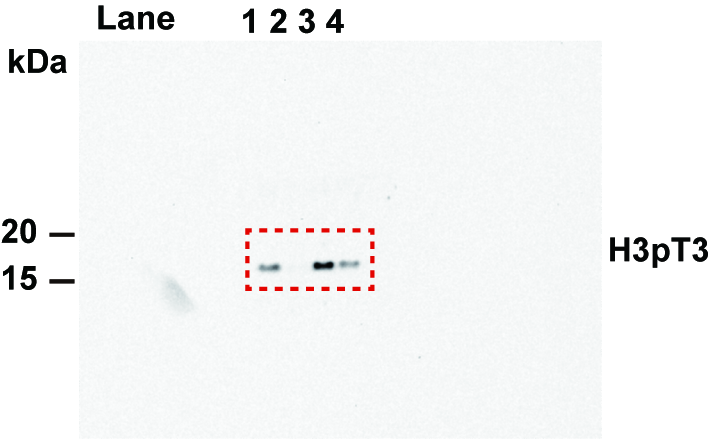

Supplement: Supplementary file 3 — Source data Fig. 1 [file 44319_2026_833_MOESM3_ESM.zip › 1A/H3pT3/Origin 1A H3pT3.tif]

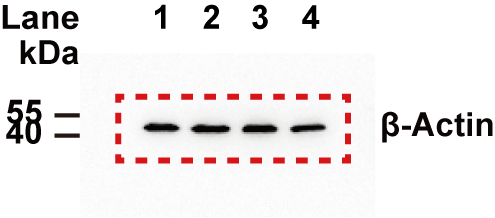

Supplement: Supplementary file 3 — Source data Fig. 1 [file 44319_2026_833_MOESM3_ESM.zip › 1B/Actin/Origin 1B Actin.tif]

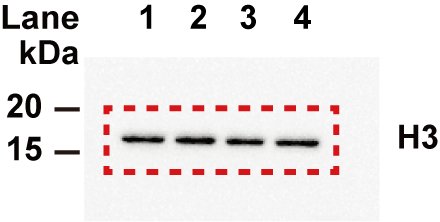

Supplement: Supplementary file 3 — Source data Fig. 1 [file 44319_2026_833_MOESM3_ESM.zip › 1B/H3/Origin 1B H3.tif]

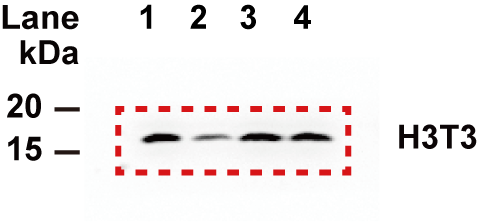

Supplement: Supplementary file 3 — Source data Fig. 1 [file 44319_2026_833_MOESM3_ESM.zip › 1B/H3pT3/Origin 1B H3pT3.tif]

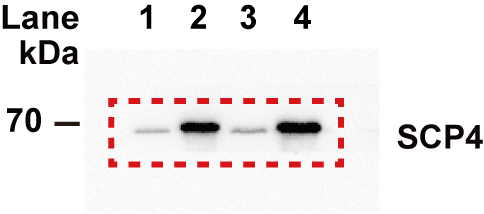

Supplement: Supplementary file 3 — Source data Fig. 1 [file 44319_2026_833_MOESM3_ESM.zip › 1B/SCP4/Origin 1B SCP4.tif]

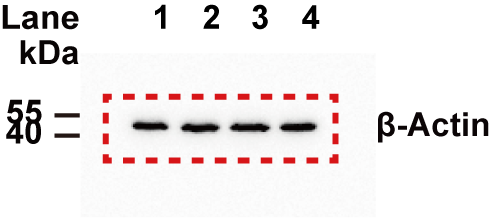

Supplement: Supplementary file 3 — Source data Fig. 1 [file 44319_2026_833_MOESM3_ESM.zip › 1C/Actin/Origin 1C Actin.tif]

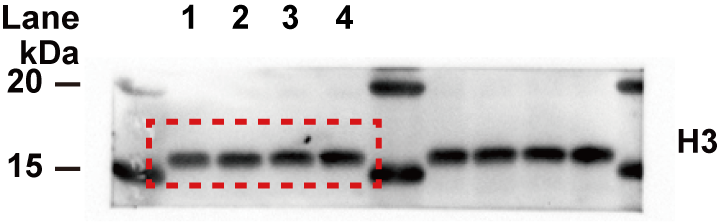

Supplement: Supplementary file 3 — Source data Fig. 1 [file 44319_2026_833_MOESM3_ESM.zip › 1C/H3/Origin 1C H3.tif]

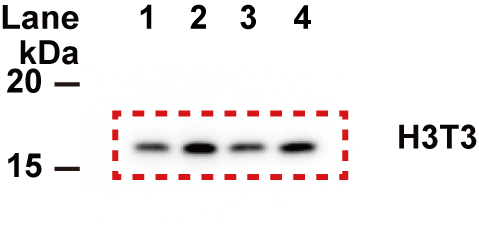

Supplement: Supplementary file 3 — Source data Fig. 1 [file 44319_2026_833_MOESM3_ESM.zip › 1C/H3pT3/Origin 1C H3pT3.tif]

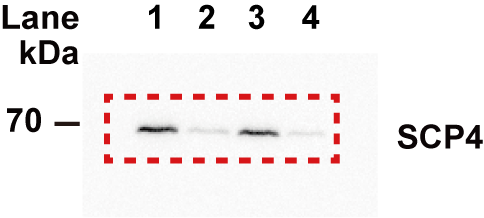

Supplement: Supplementary file 3 — Source data Fig. 1 [file 44319_2026_833_MOESM3_ESM.zip › 1C/SCP4/Origin 1C SCP4.tif]

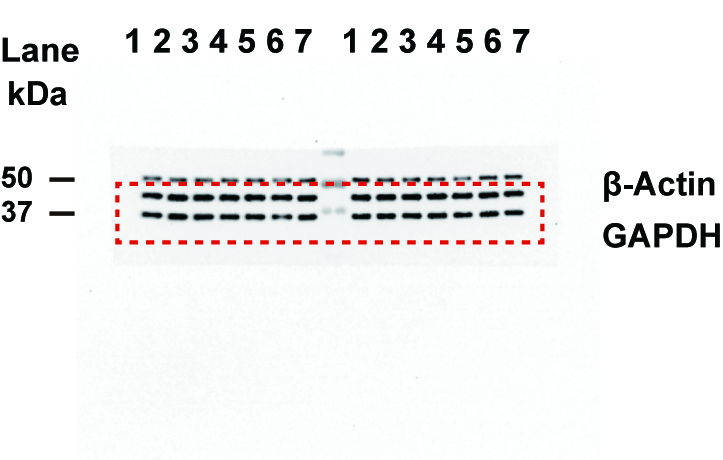

Supplement: Supplementary file 3 — Source data Fig. 1 [file 44319_2026_833_MOESM3_ESM.zip › 1D/Actin GAPDH/Origin 1D Actin GAPDH.tif]

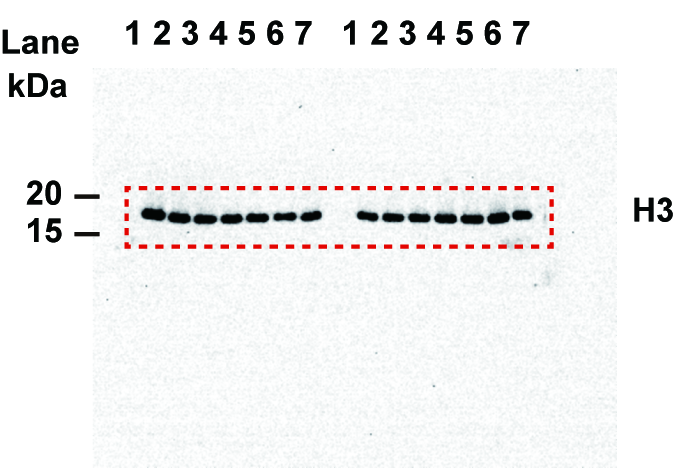

Supplement: Supplementary file 3 — Source data Fig. 1 [file 44319_2026_833_MOESM3_ESM.zip › 1D/H3/Origin 1D H3.tif]

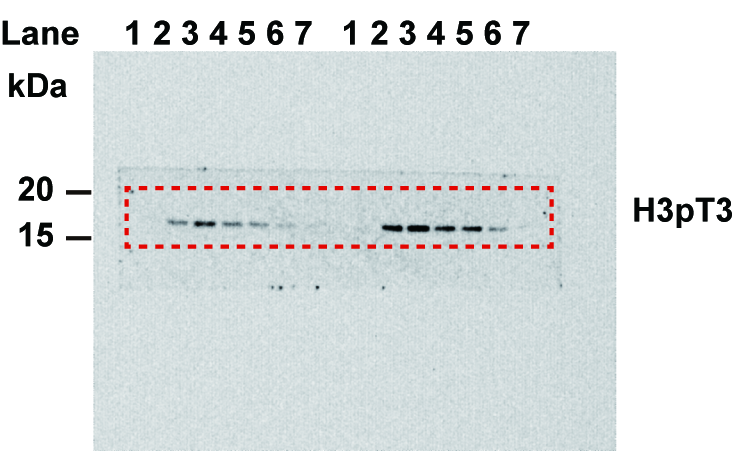

Supplement: Supplementary file 3 — Source data Fig. 1 [file 44319_2026_833_MOESM3_ESM.zip › 1D/H3pT3/Origin 1D H3pT3.tif]

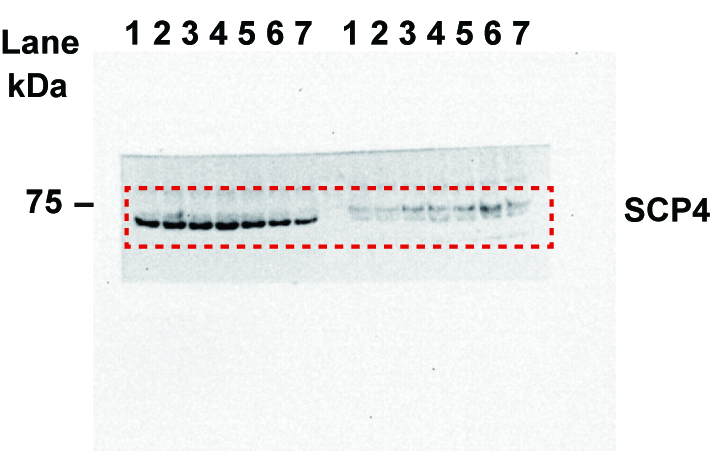

Supplement: Supplementary file 3 — Source data Fig. 1 [file 44319_2026_833_MOESM3_ESM.zip › 1D/SCP4/Origin 1D SCP4.tif]

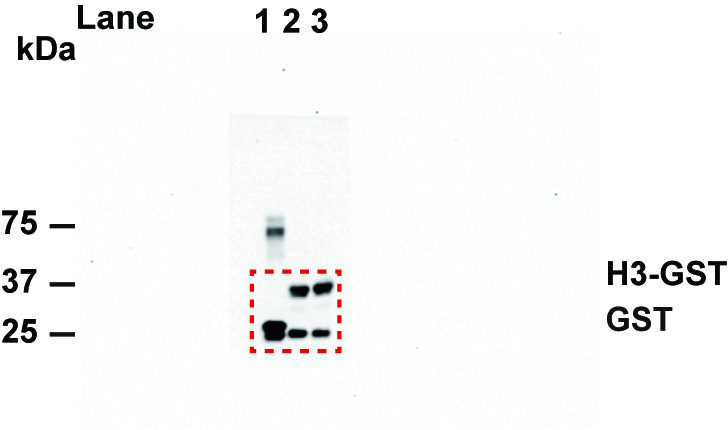

Supplement: Supplementary file 3 — Source data Fig. 1 [file 44319_2026_833_MOESM3_ESM.zip › 1E/H3-GST GST/Origin 1E GST H3-GST.tif]

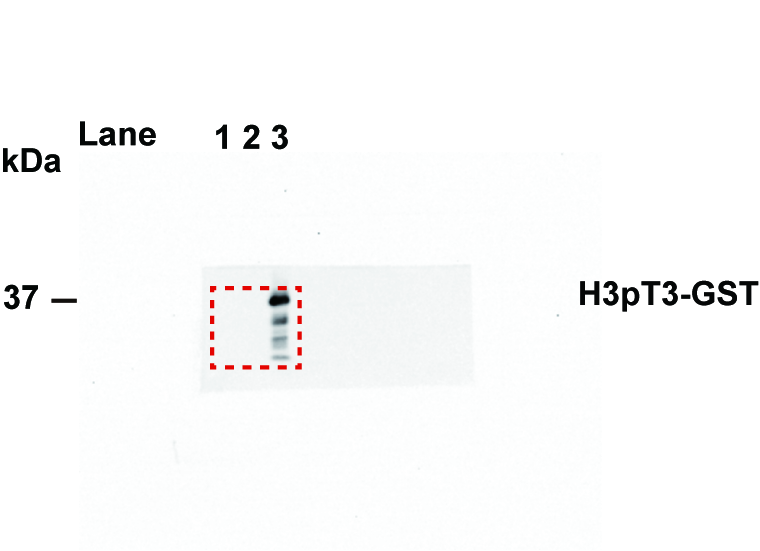

Supplement: Supplementary file 3 — Source data Fig. 1 [file 44319_2026_833_MOESM3_ESM.zip › 1E/H3pT3-GST/Origin 1E GST H3pT3-GST.tif]

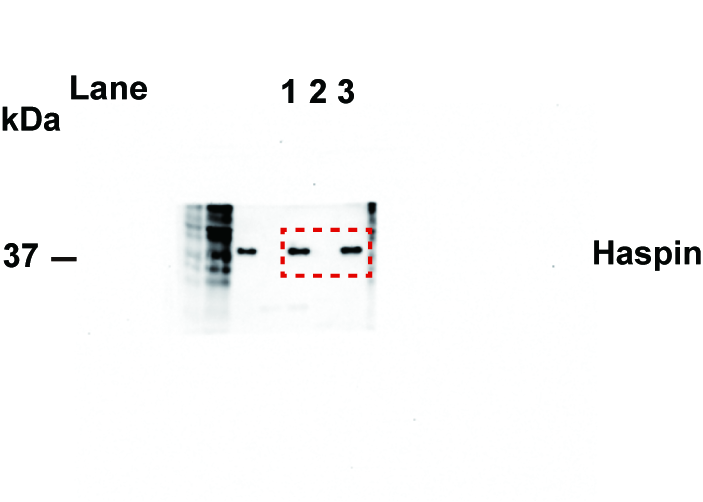

Supplement: Supplementary file 3 — Source data Fig. 1 [file 44319_2026_833_MOESM3_ESM.zip › 1E/Haspin/Origin 1E Haspin.tif]

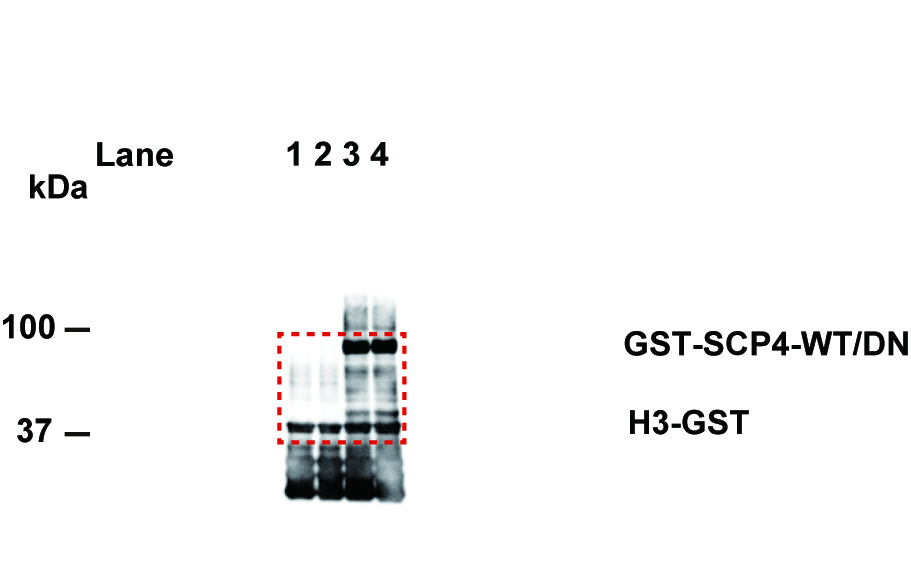

Supplement: Supplementary file 3 — Source data Fig. 1 [file 44319_2026_833_MOESM3_ESM.zip › 1F/GST-SCP4WT_DN H3-GST/Origin 1F H3-GST GST-SCP4-WT_DN.tif]

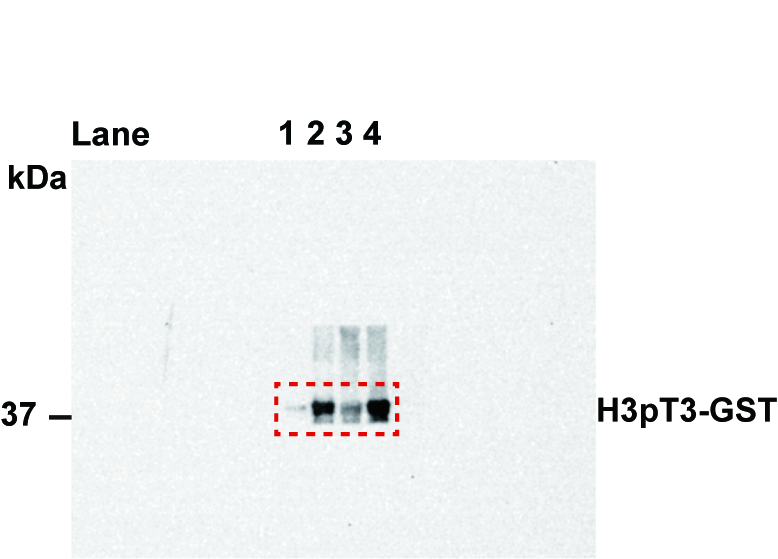

Supplement: Supplementary file 3 — Source data Fig. 1 [file 44319_2026_833_MOESM3_ESM.zip › 1F/H3pT3-GST/Origin 1F GST H3pT3-GST.tif]

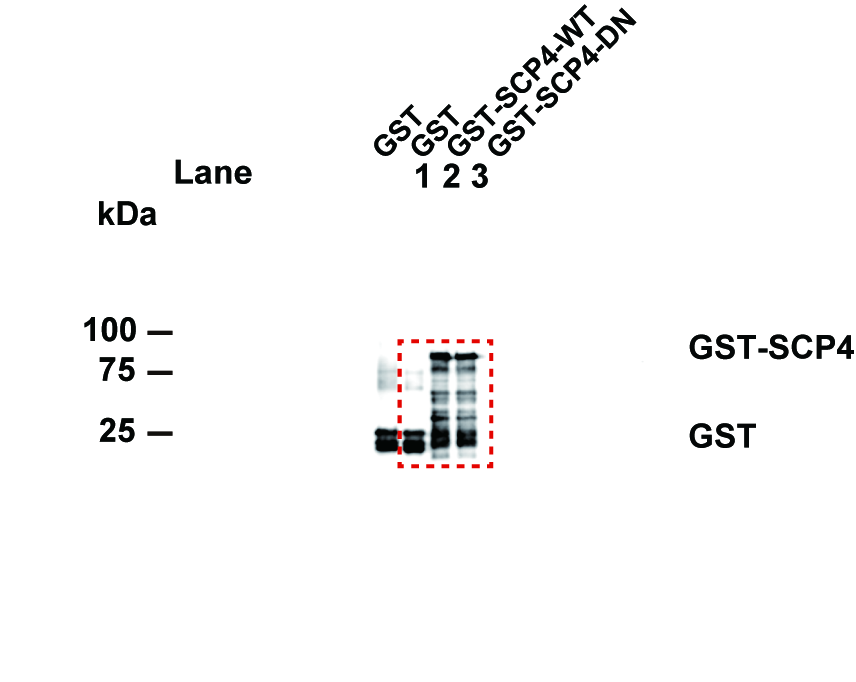

Supplement: Supplementary file 4 — Source data Fig. 2 [file 44319_2026_833_MOESM4_ESM.zip › 2B/GST GST-SCP4/Origin 2B GST GST-SCP4.tif]

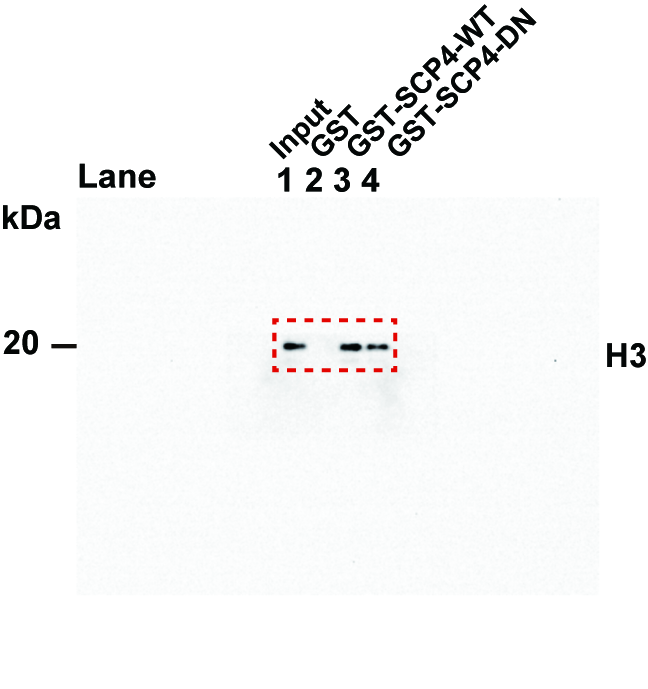

Supplement: Supplementary file 4 — Source data Fig. 2 [file 44319_2026_833_MOESM4_ESM.zip › 2B/H3/Origin 2B H3.tif]

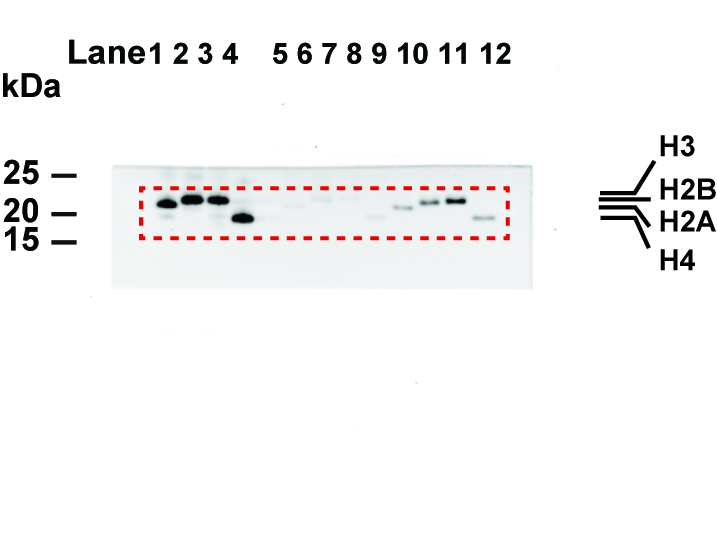

Supplement: Supplementary file 4 — Source data Fig. 2 [file 44319_2026_833_MOESM4_ESM.zip › 2C/IB_α-FLAG/Origin 2C IB_α-FLAG.tif]

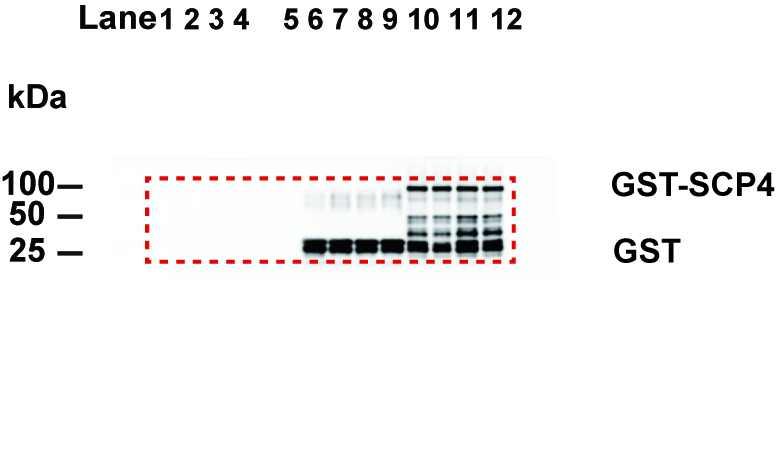

Supplement: Supplementary file 4 — Source data Fig. 2 [file 44319_2026_833_MOESM4_ESM.zip › 2C/IB_α-GST/Origin 2C IB_α-GST.tif]

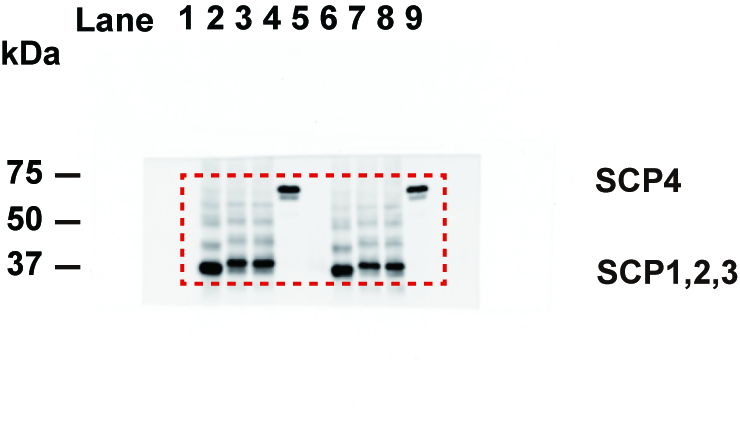

Supplement: Supplementary file 4 — Source data Fig. 2 [file 44319_2026_833_MOESM4_ESM.zip › 2D/IP_α-FLAG IB_α-FLAG/Origin 2D IP_α-FLAG IB_α-FLAG.tif]

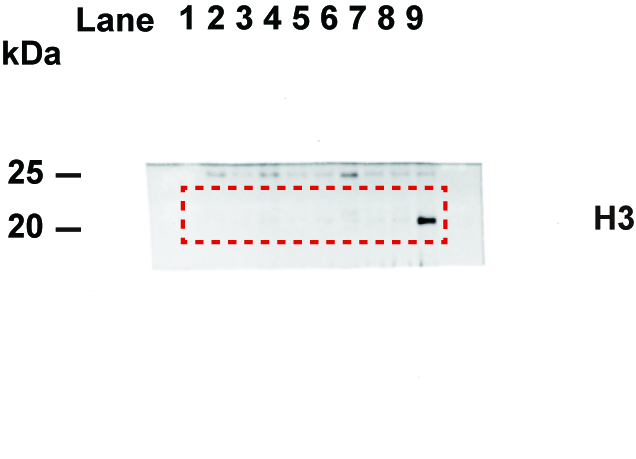

Supplement: Supplementary file 4 — Source data Fig. 2 [file 44319_2026_833_MOESM4_ESM.zip › 2D/IP_α-FLAG IB_α-Myc/Origin 2D IP_α-FLAG IB_α-Myc.tif]

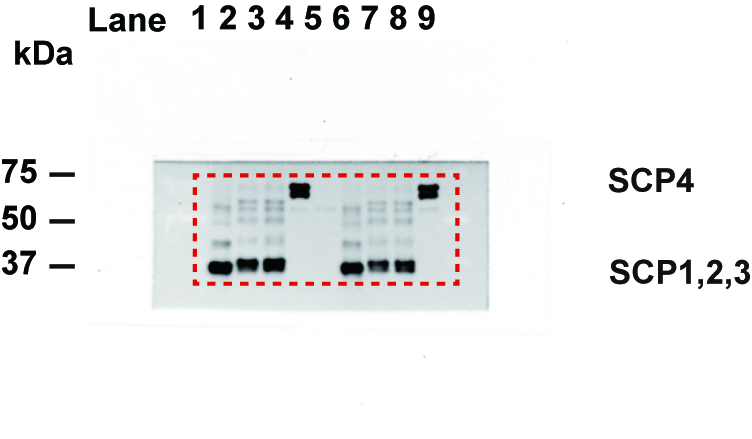

Supplement: Supplementary file 4 — Source data Fig. 2 [file 44319_2026_833_MOESM4_ESM.zip › 2D/WCL IB_α-FLAG/Origin 2D WCL IB_α-FLAG.tif]

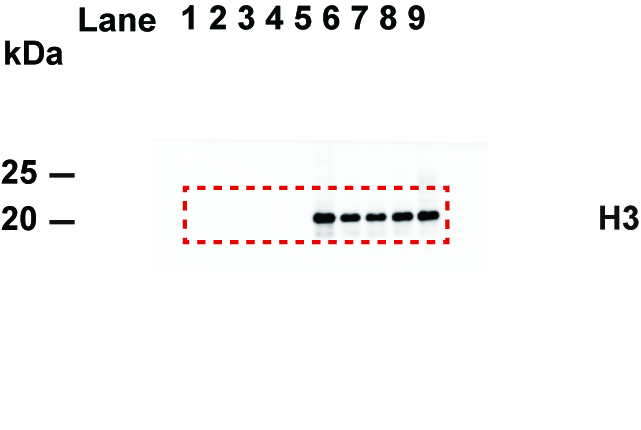

Supplement: Supplementary file 4 — Source data Fig. 2 [file 44319_2026_833_MOESM4_ESM.zip › 2D/WCL IB_α-Myc/Origin 2D WCL IB_α-Myc.tif]

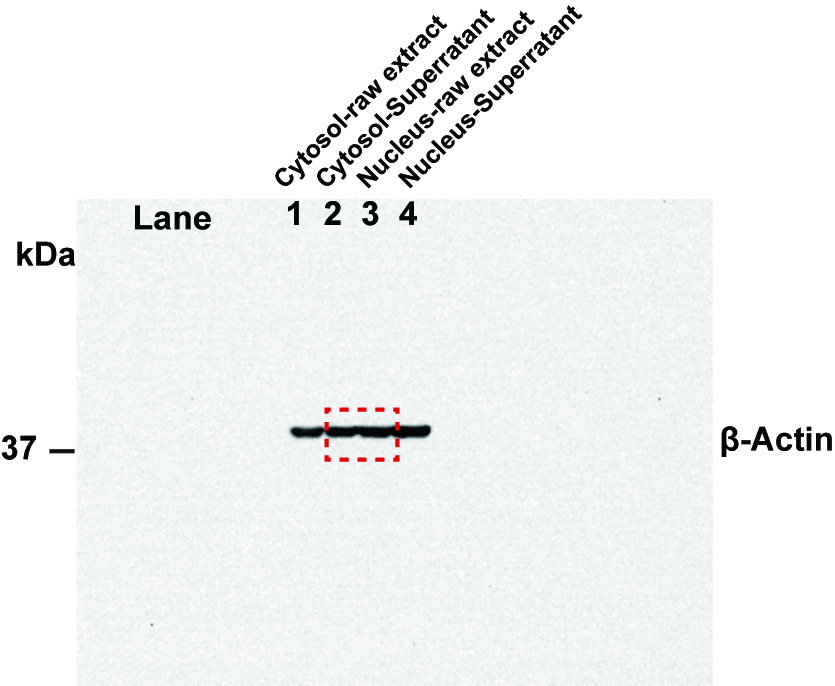

Supplement: Supplementary file 4 — Source data Fig. 2 [file 44319_2026_833_MOESM4_ESM.zip › 2E/Actin/Origin 2E Actin.tif]

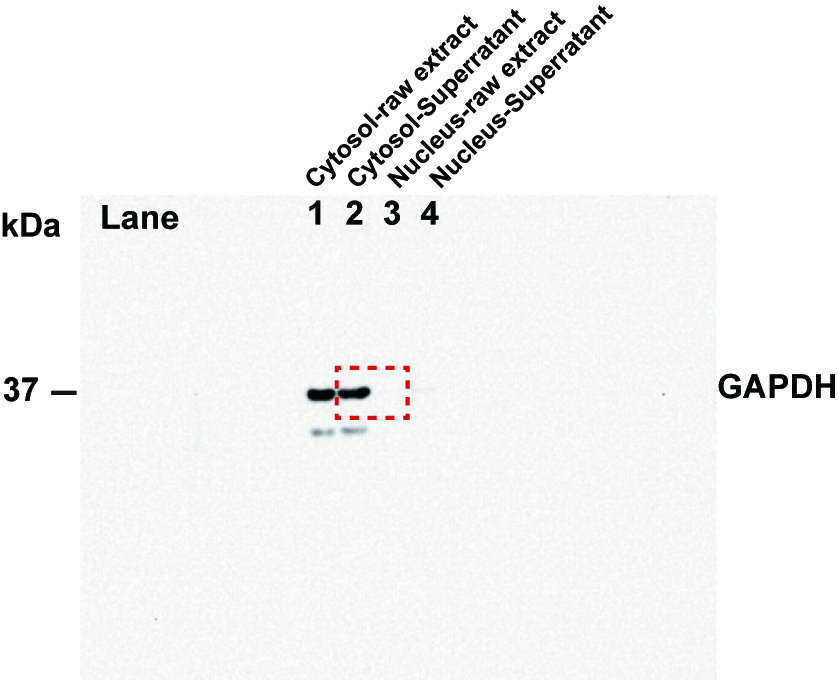

Supplement: Supplementary file 4 — Source data Fig. 2 [file 44319_2026_833_MOESM4_ESM.zip › 2E/GAPDH/Origin 2E GAPDH.tif]

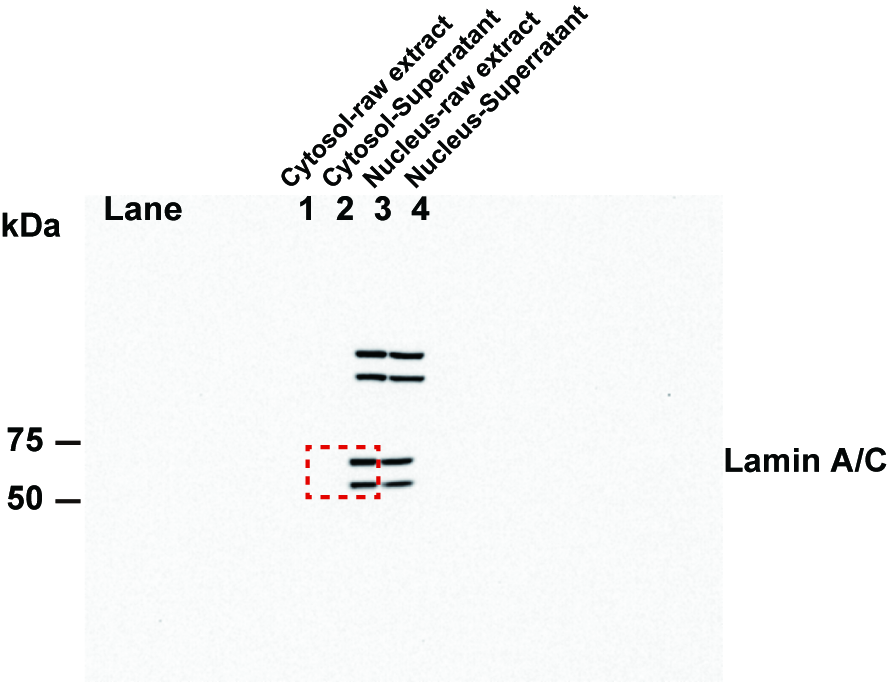

Supplement: Supplementary file 4 — Source data Fig. 2 [file 44319_2026_833_MOESM4_ESM.zip › 2E/Lamin A_C/Origin 2E LaminA_C.tif]

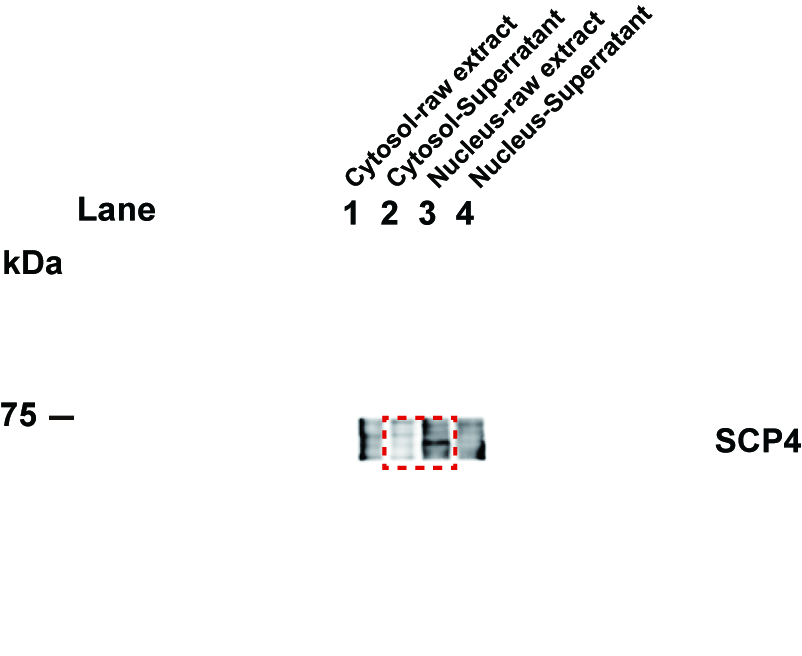

Supplement: Supplementary file 4 — Source data Fig. 2 [file 44319_2026_833_MOESM4_ESM.zip › 2E/SCP4/Origin 2E SCP4.tif]

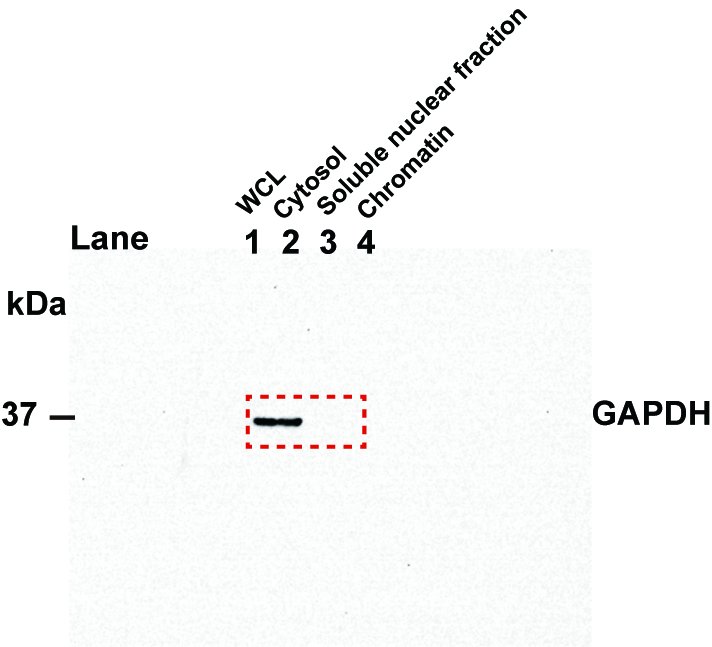

Supplement: Supplementary file 4 — Source data Fig. 2 [file 44319_2026_833_MOESM4_ESM.zip › 2F/GAPDH/Origin 2F GAPDH.tif]

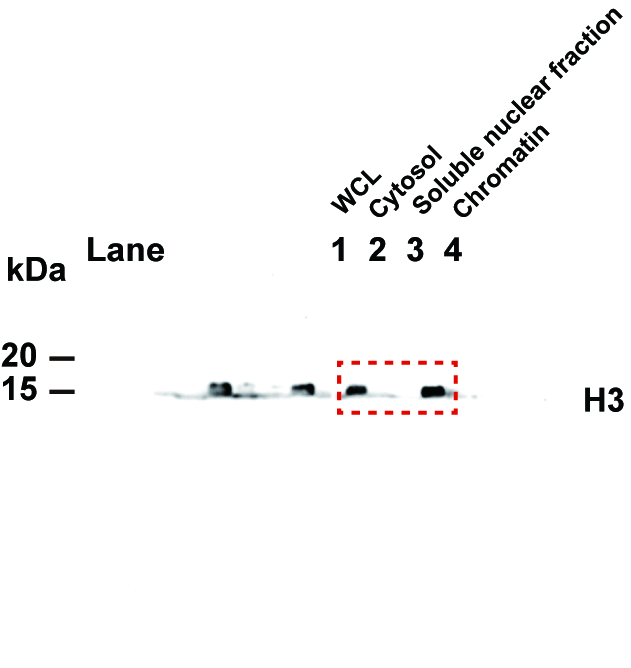

Supplement: Supplementary file 4 — Source data Fig. 2 [file 44319_2026_833_MOESM4_ESM.zip › 2F/H3/Origin 2F H3.tif]

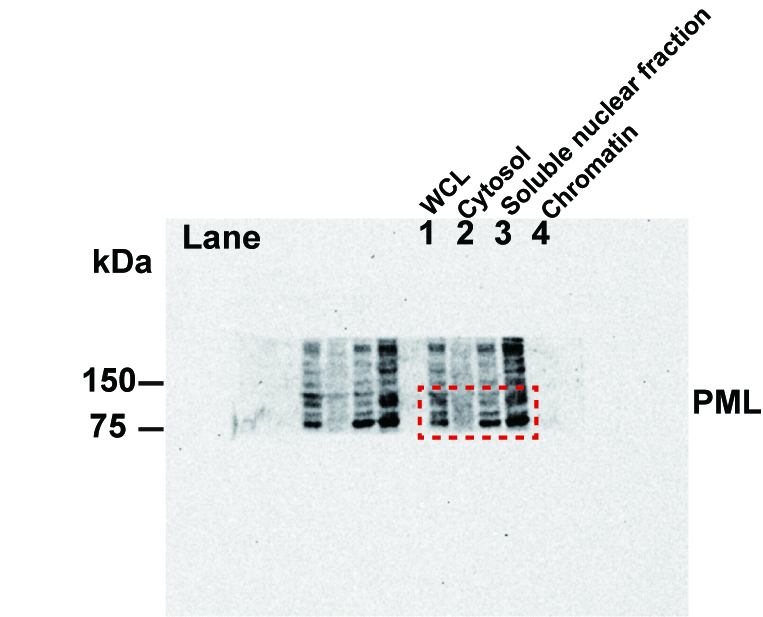

Supplement: Supplementary file 4 — Source data Fig. 2 [file 44319_2026_833_MOESM4_ESM.zip › 2F/PML/Origin 2F PML.tif]

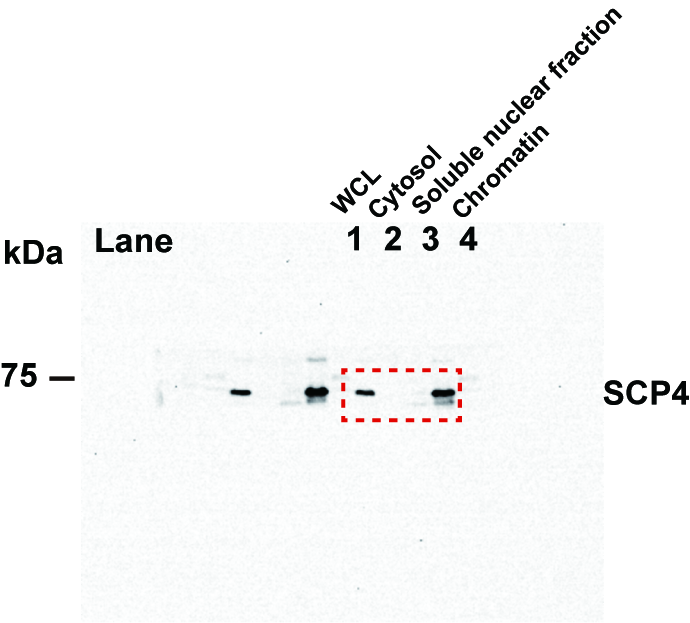

Supplement: Supplementary file 4 — Source data Fig. 2 [file 44319_2026_833_MOESM4_ESM.zip › 2F/SCP4/Origin 2F SCP4.tif]

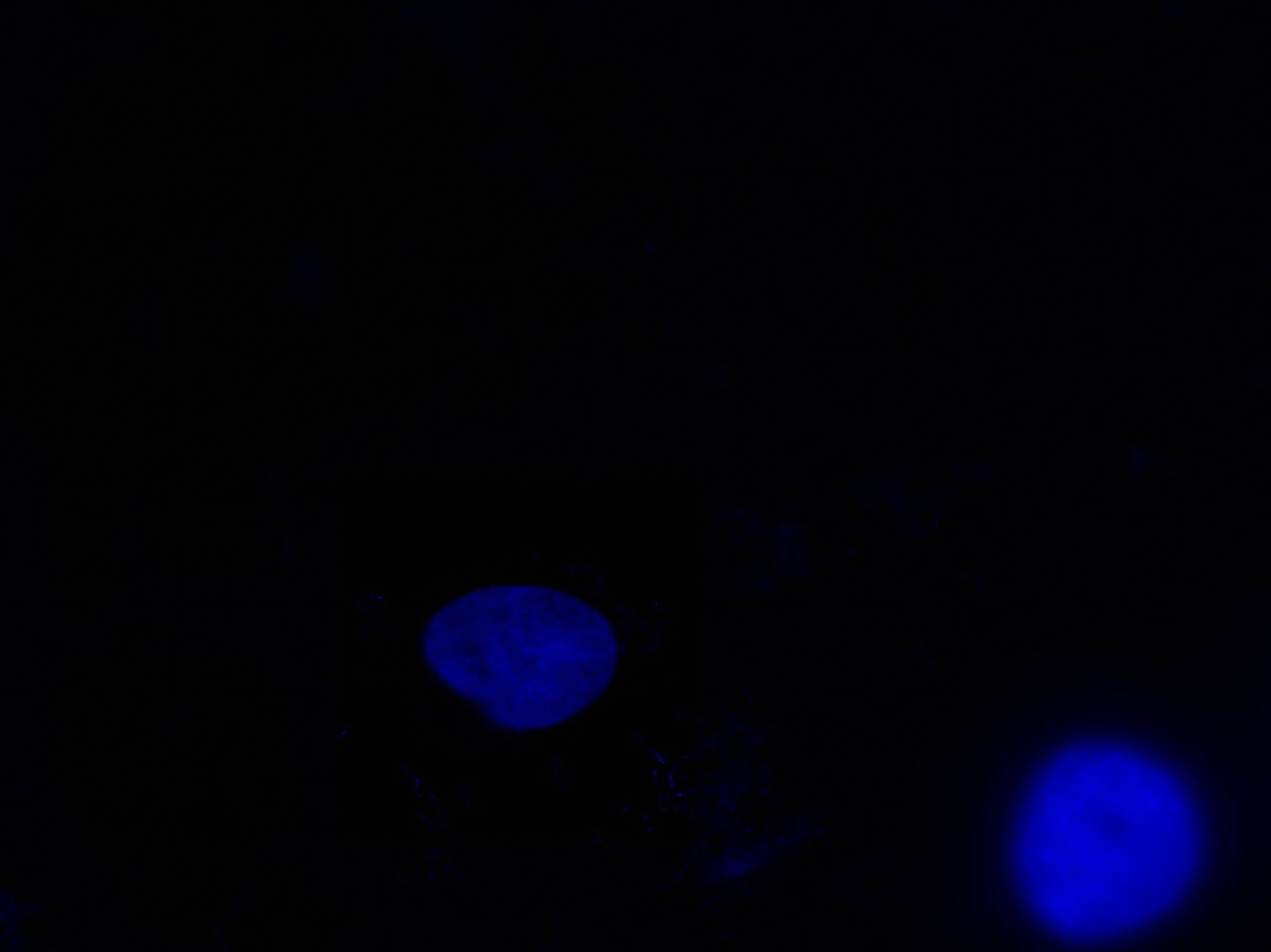

Supplement: Supplementary file 4 — Source data Fig. 2 [file 44319_2026_833_MOESM4_ESM.zip › 2G/SCP4(R)_Tubulin(G)_005_dec_z3(c1).tif]

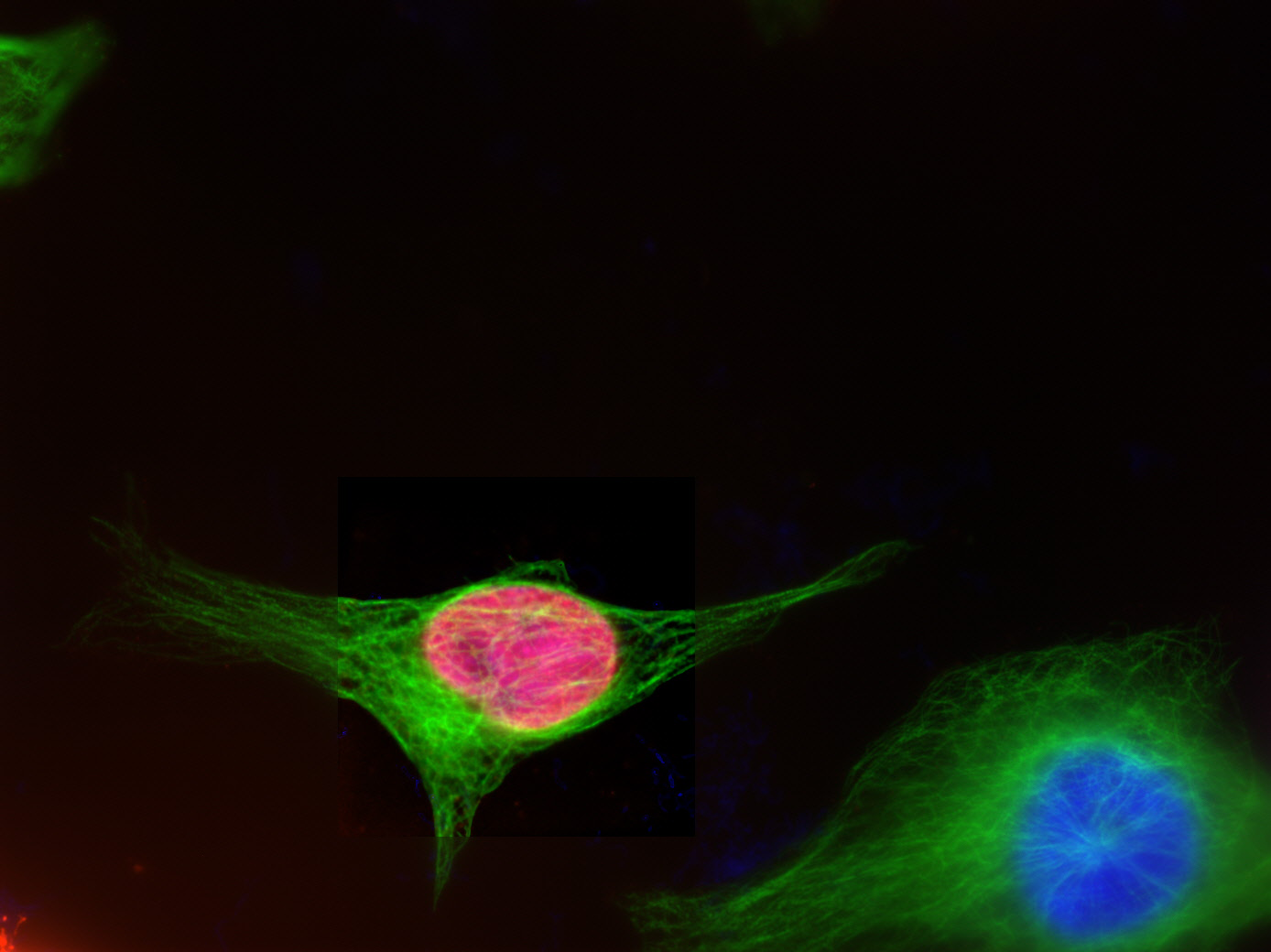

Supplement: Supplementary file 4 — Source data Fig. 2 [file 44319_2026_833_MOESM4_ESM.zip › 2G/SCP4(R)_Tubulin(G)_005_dec_z3(c1+c2+c3).tif]

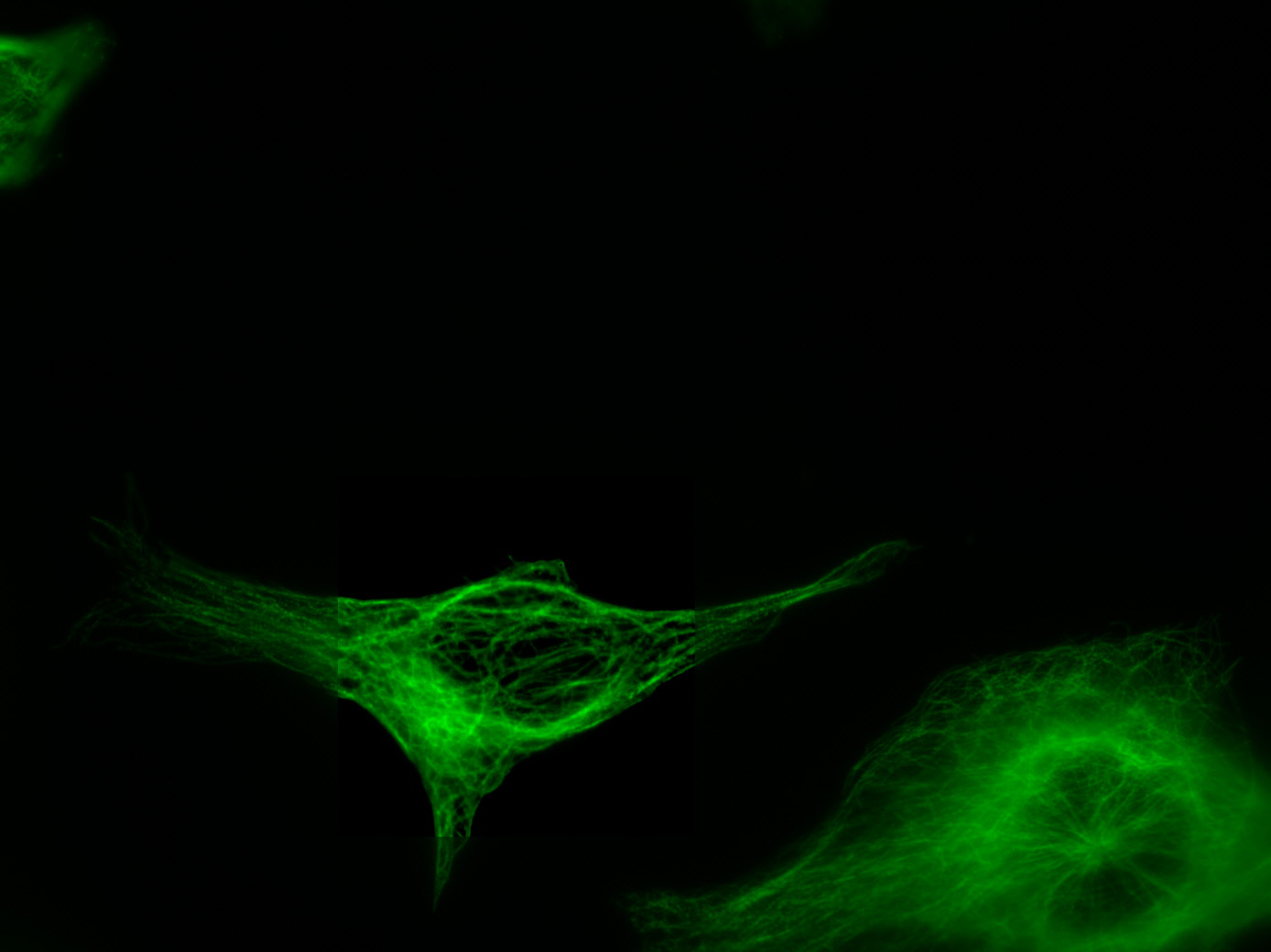

Supplement: Supplementary file 4 — Source data Fig. 2 [file 44319_2026_833_MOESM4_ESM.zip › 2G/SCP4(R)_Tubulin(G)_005_dec_z3(c2).tif]

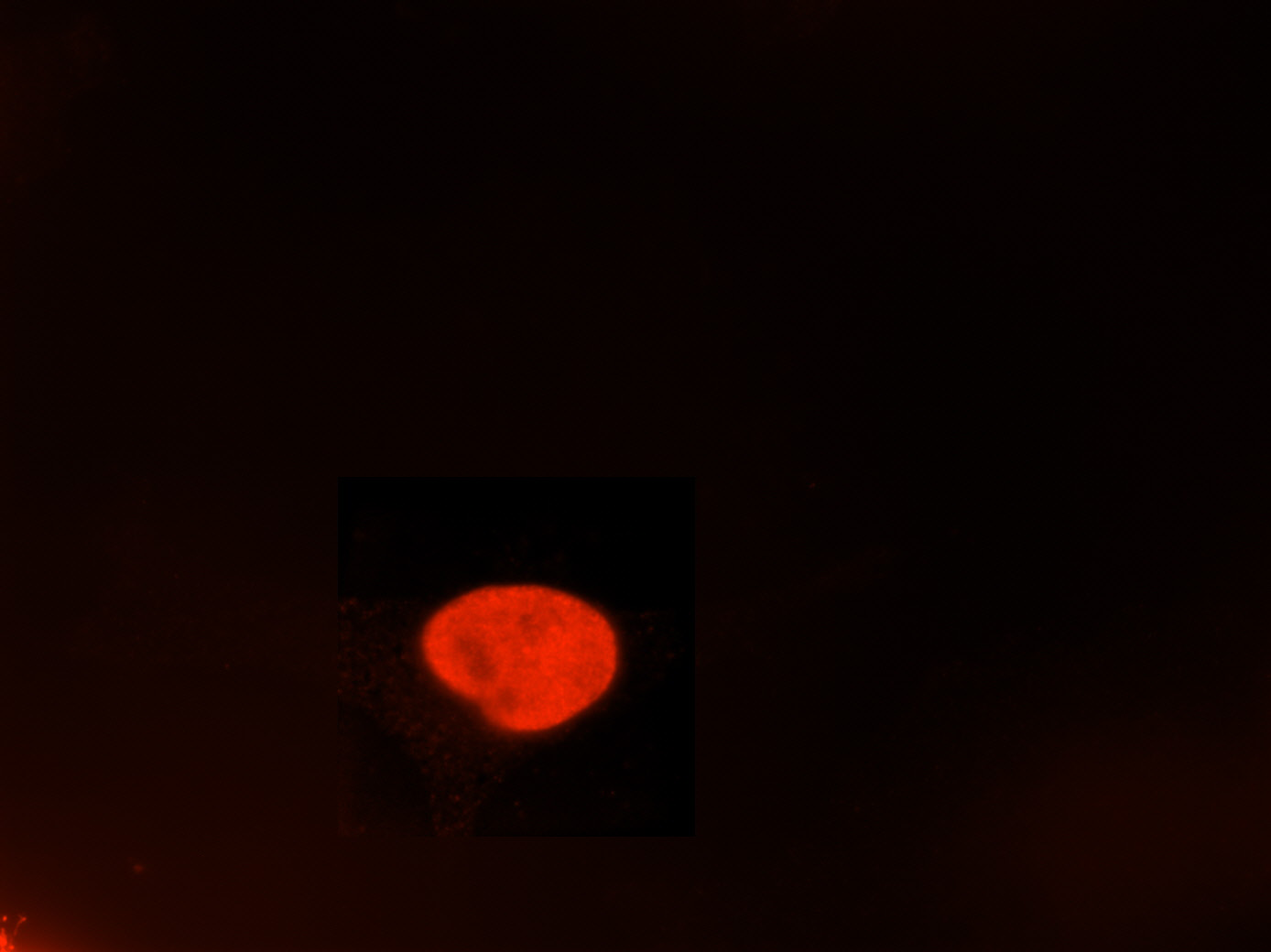

Supplement: Supplementary file 4 — Source data Fig. 2 [file 44319_2026_833_MOESM4_ESM.zip › 2G/SCP4(R)_Tubulin(G)_005_dec_z3(c3).tif]

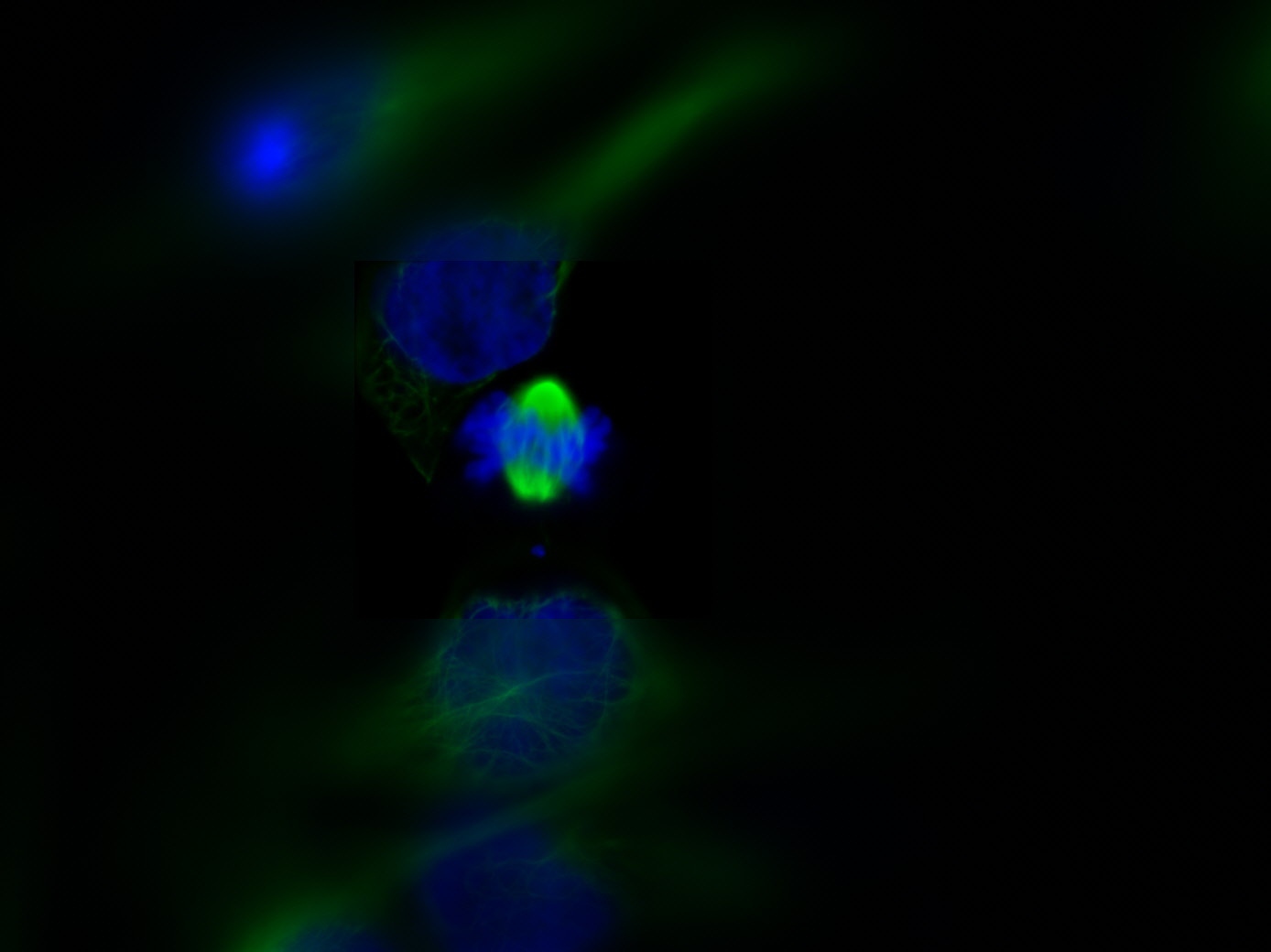

Supplement: Supplementary file 4 — Source data Fig. 2 [file 44319_2026_833_MOESM4_ESM.zip › 2H/SCP4(R)_Tubulin(G)_007_dec_z10(c1+c2).tif]

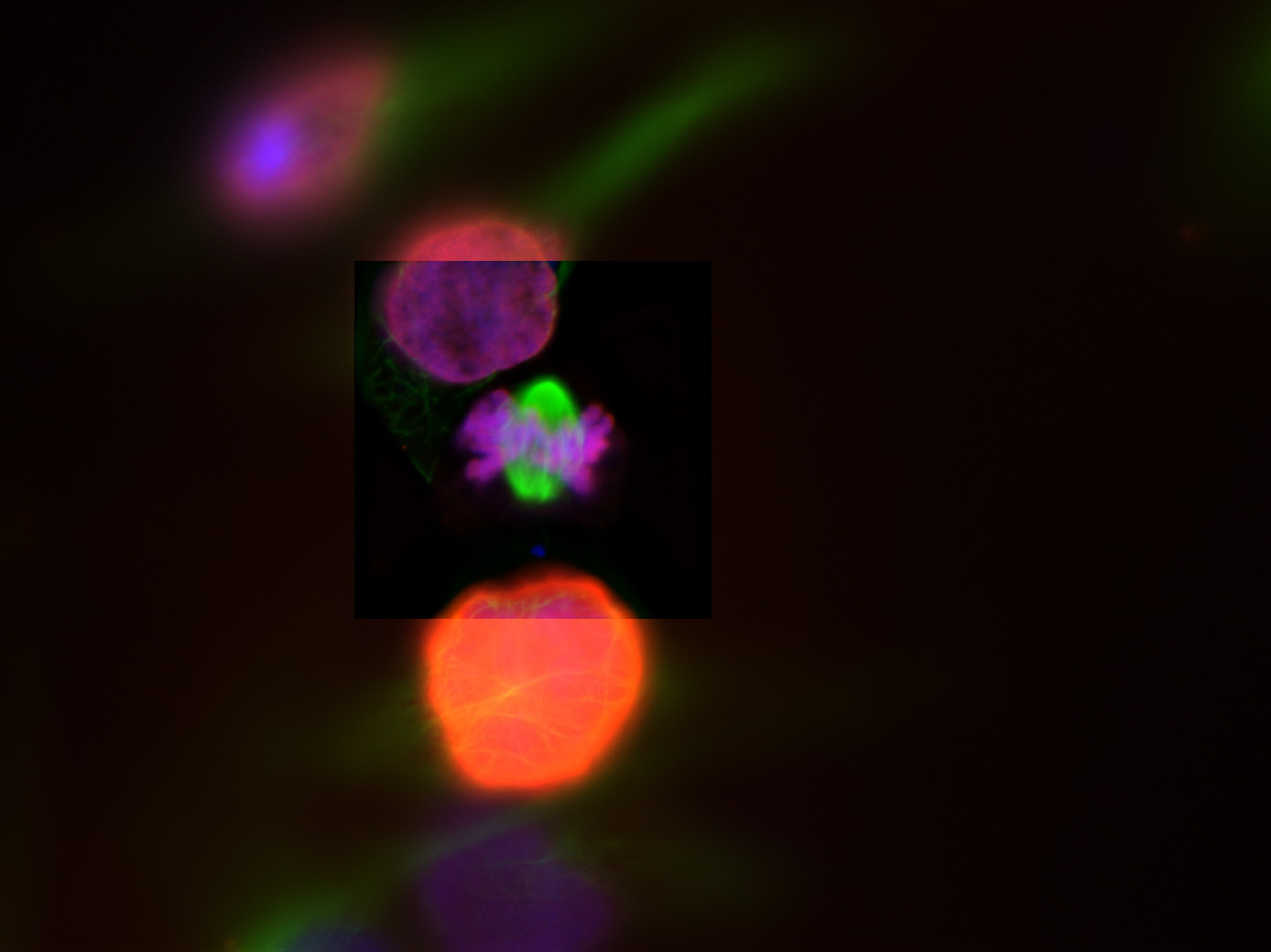

Supplement: Supplementary file 4 — Source data Fig. 2 [file 44319_2026_833_MOESM4_ESM.zip › 2H/SCP4(R)_Tubulin(G)_007_dec_z10(c1+c2+c3).tif]

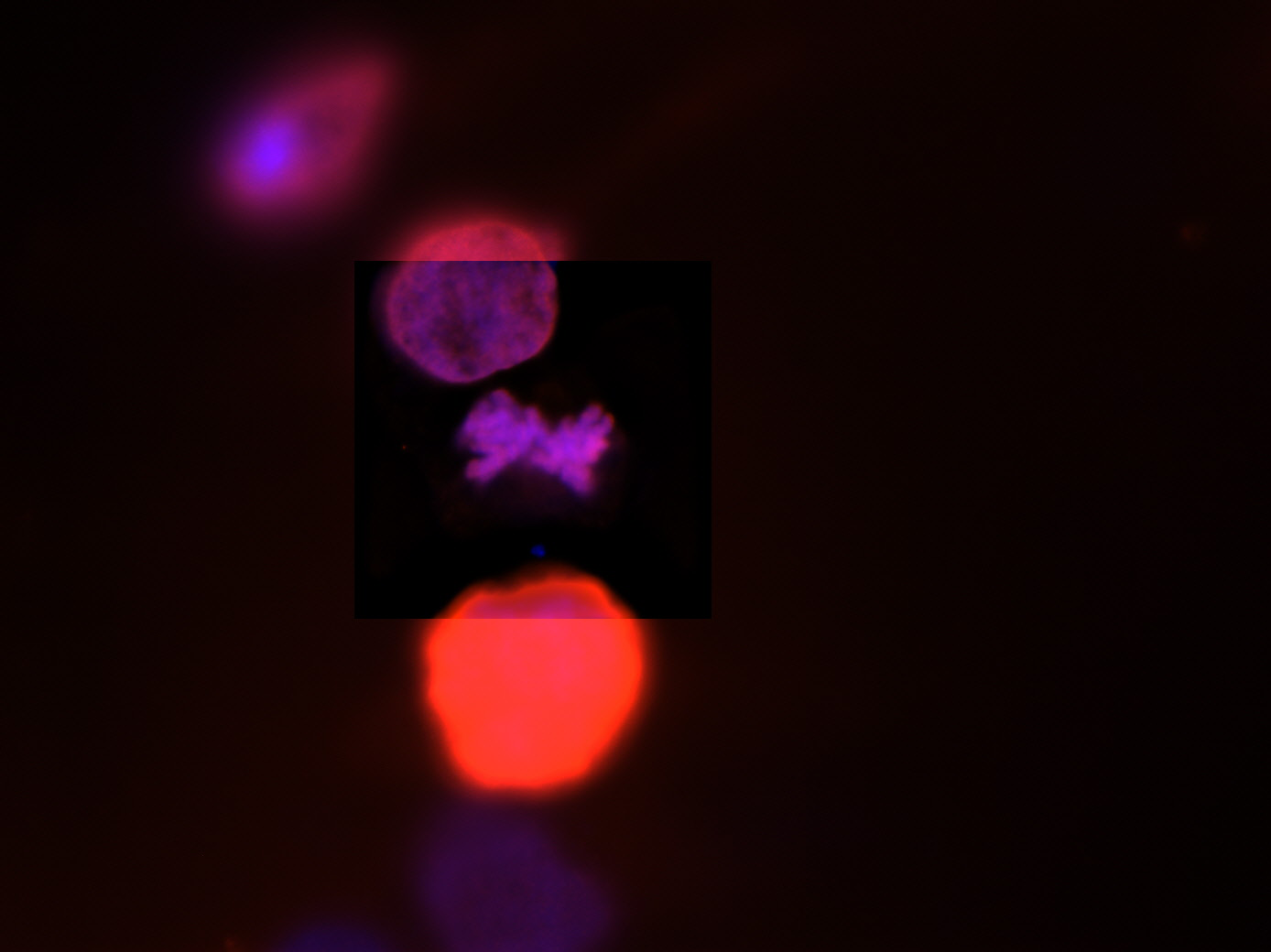

Supplement: Supplementary file 4 — Source data Fig. 2 [file 44319_2026_833_MOESM4_ESM.zip › 2H/SCP4(R)_Tubulin(G)_007_dec_z10(c1+c3).tif]

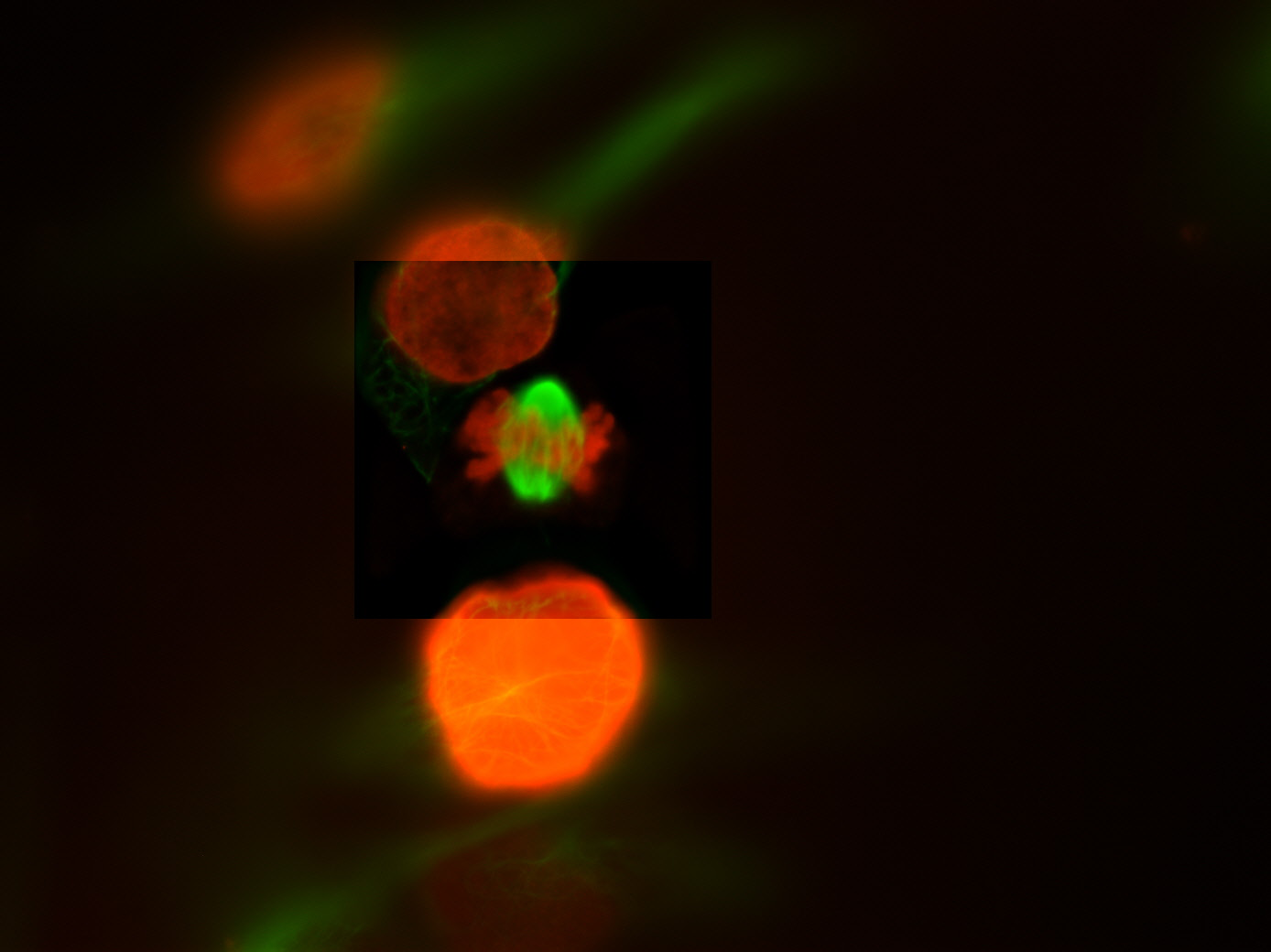

Supplement: Supplementary file 4 — Source data Fig. 2 [file 44319_2026_833_MOESM4_ESM.zip › 2H/SCP4(R)_Tubulin(G)_007_dec_z10(c2+c3).tif]

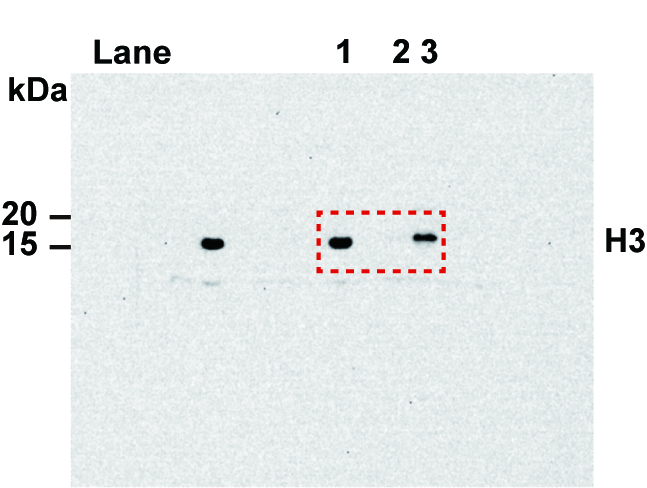

Supplement: Supplementary file 4 — Source data Fig. 2 [file 44319_2026_833_MOESM4_ESM.zip › 2A/H3/Origin 2A H3.tif]

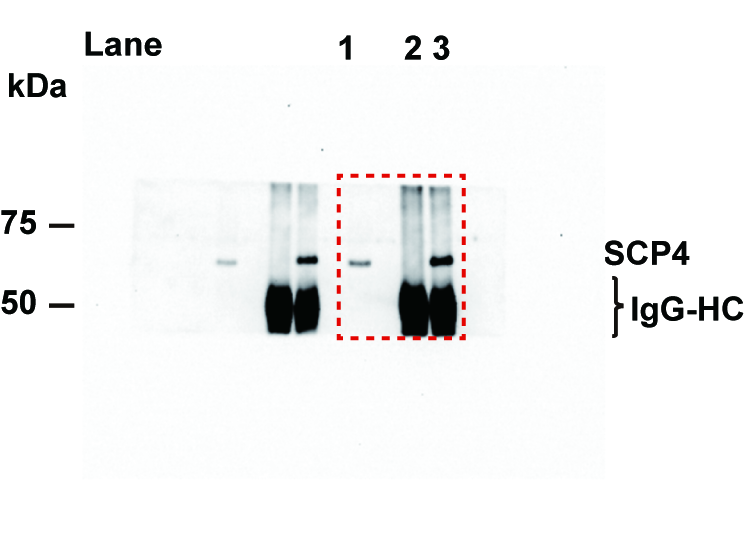

Supplement: Supplementary file 4 — Source data Fig. 2 [file 44319_2026_833_MOESM4_ESM.zip › 2A/SCP4 lgG-HC/Origin 2A SCP4 IgG-HC.tif]

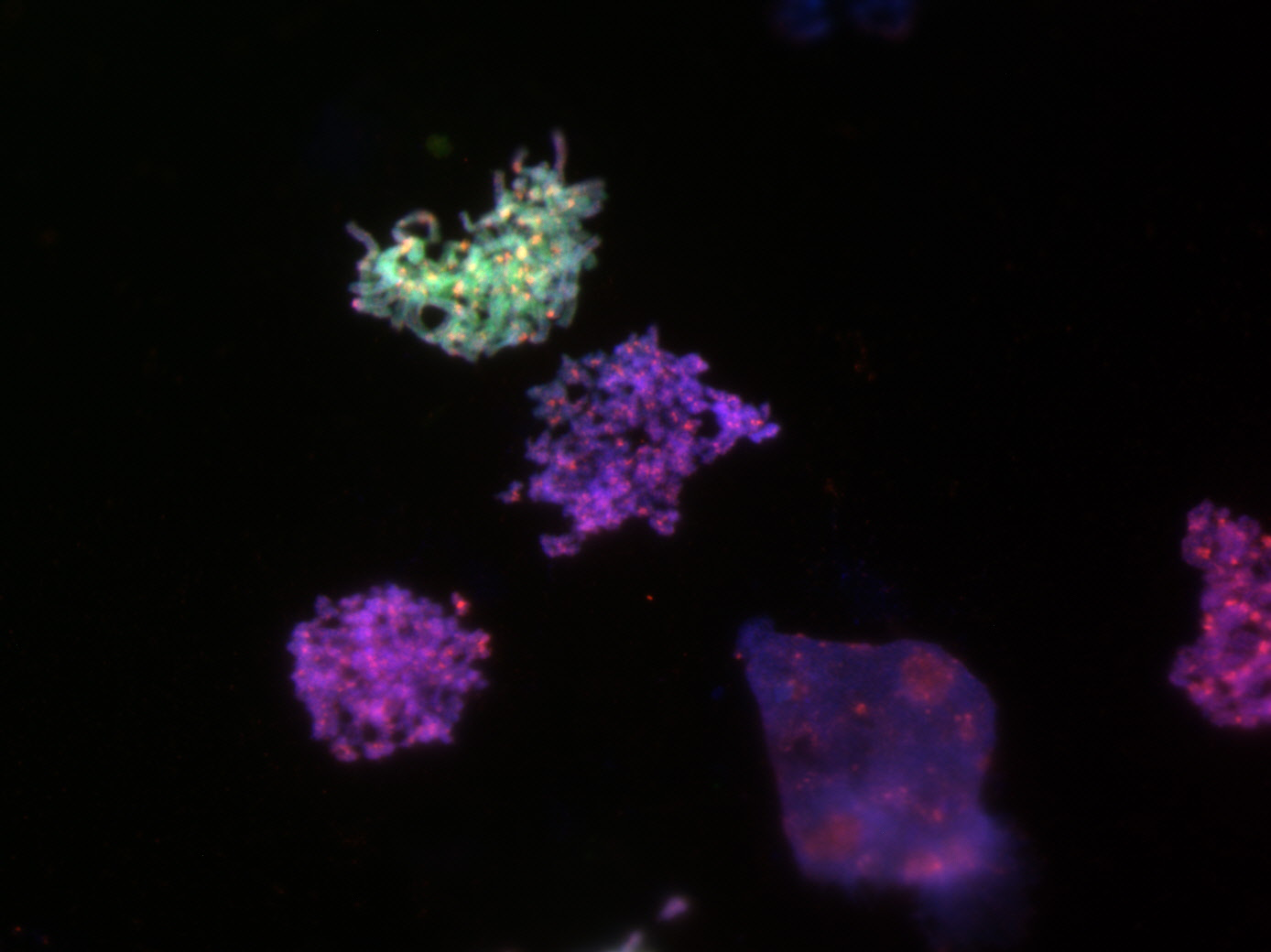

Supplement: Supplementary file 5 — Source data Fig. 3 [file 44319_2026_833_MOESM5_ESM.zip › 3A/EGFP-SMP4dn_Survivin_Chrom_0813_007_(DAPI+FiTC+Texasred).tif]

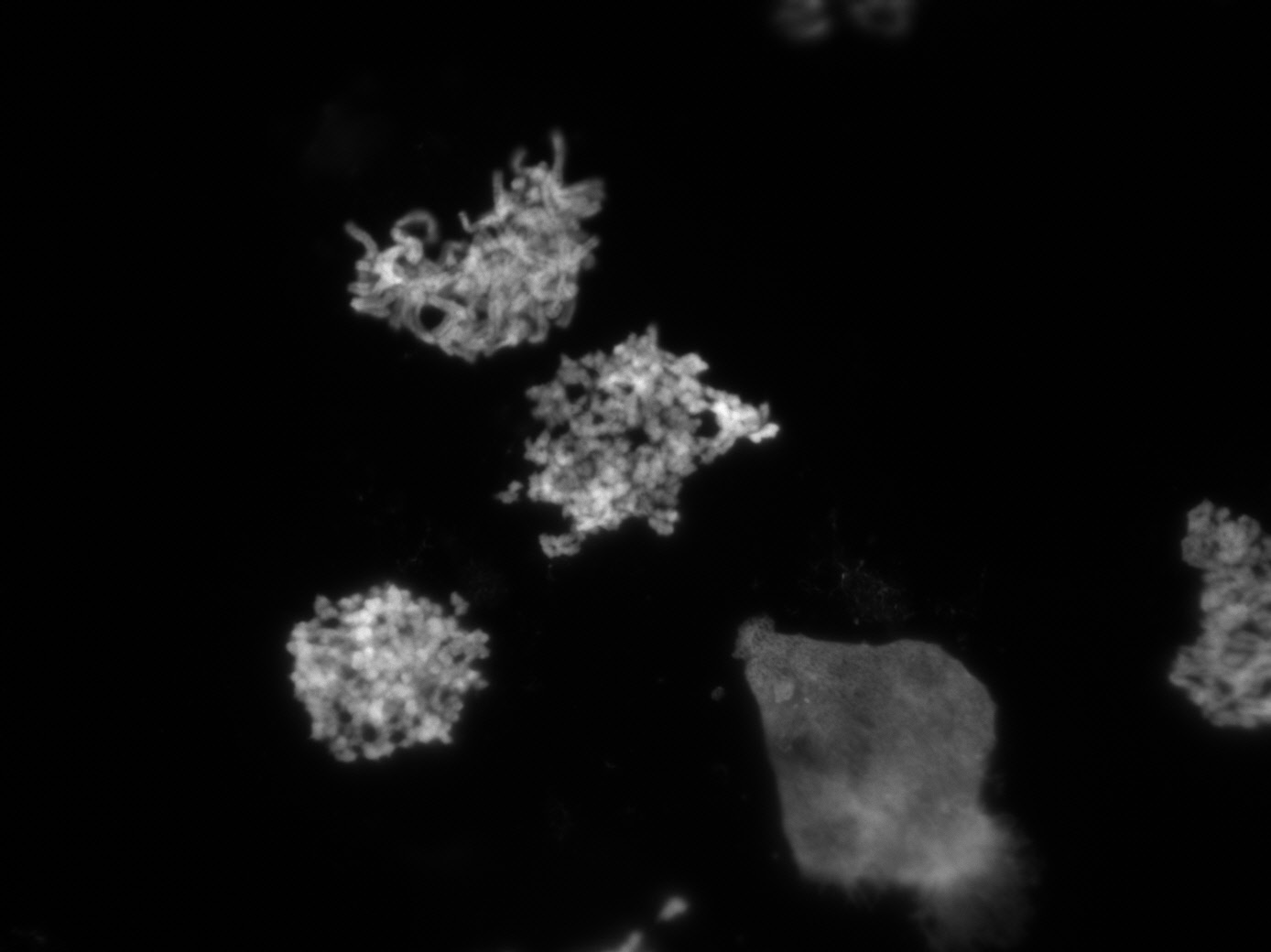

Supplement: Supplementary file 5 — Source data Fig. 3 [file 44319_2026_833_MOESM5_ESM.zip › 3A/EGFP-SMP4dn_Survivin_Chrom_0813_007_DAPI.tif]

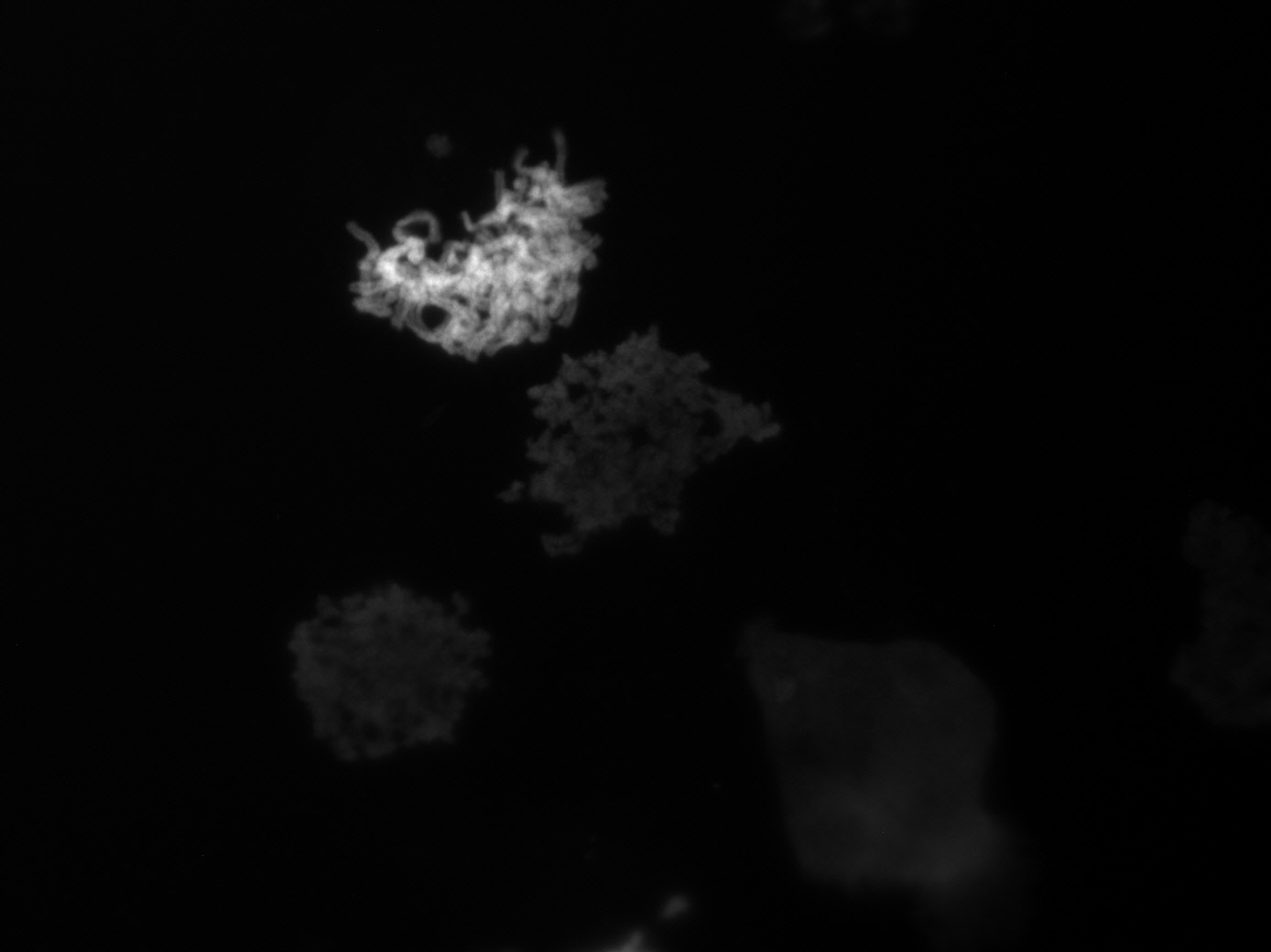

Supplement: Supplementary file 5 — Source data Fig. 3 [file 44319_2026_833_MOESM5_ESM.zip › 3A/EGFP-SMP4dn_Survivin_Chrom_0813_007_FiTC.tif]

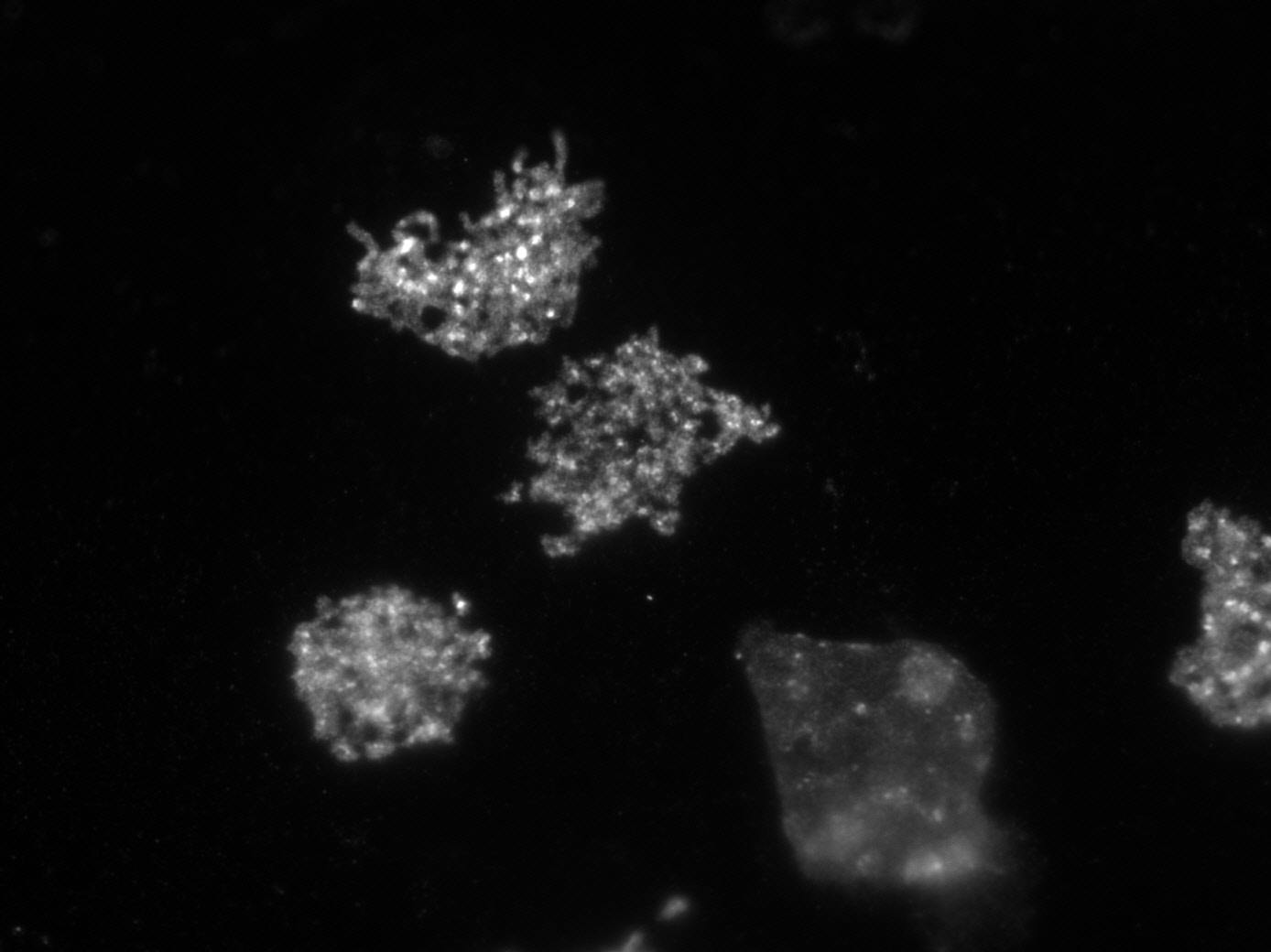

Supplement: Supplementary file 5 — Source data Fig. 3 [file 44319_2026_833_MOESM5_ESM.zip › 3A/EGFP-SMP4dn_Survivin_Chrom_0813_007_Texasred.tif]

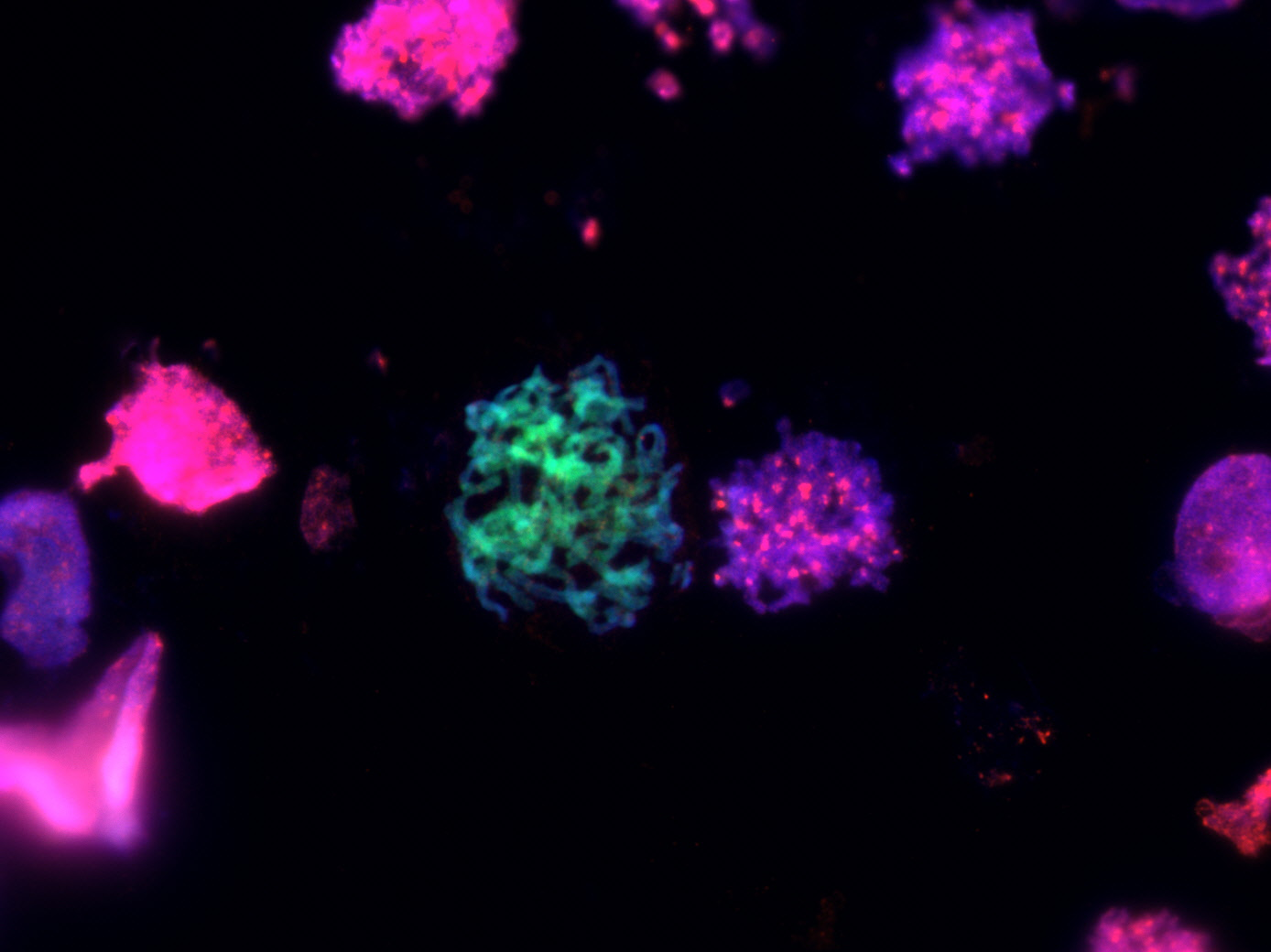

Supplement: Supplementary file 5 — Source data Fig. 3 [file 44319_2026_833_MOESM5_ESM.zip › 3A/EGFP-SMP4_Survivin_Chrom_0813_006_(DAPI+FiTC+Texasred).tif]

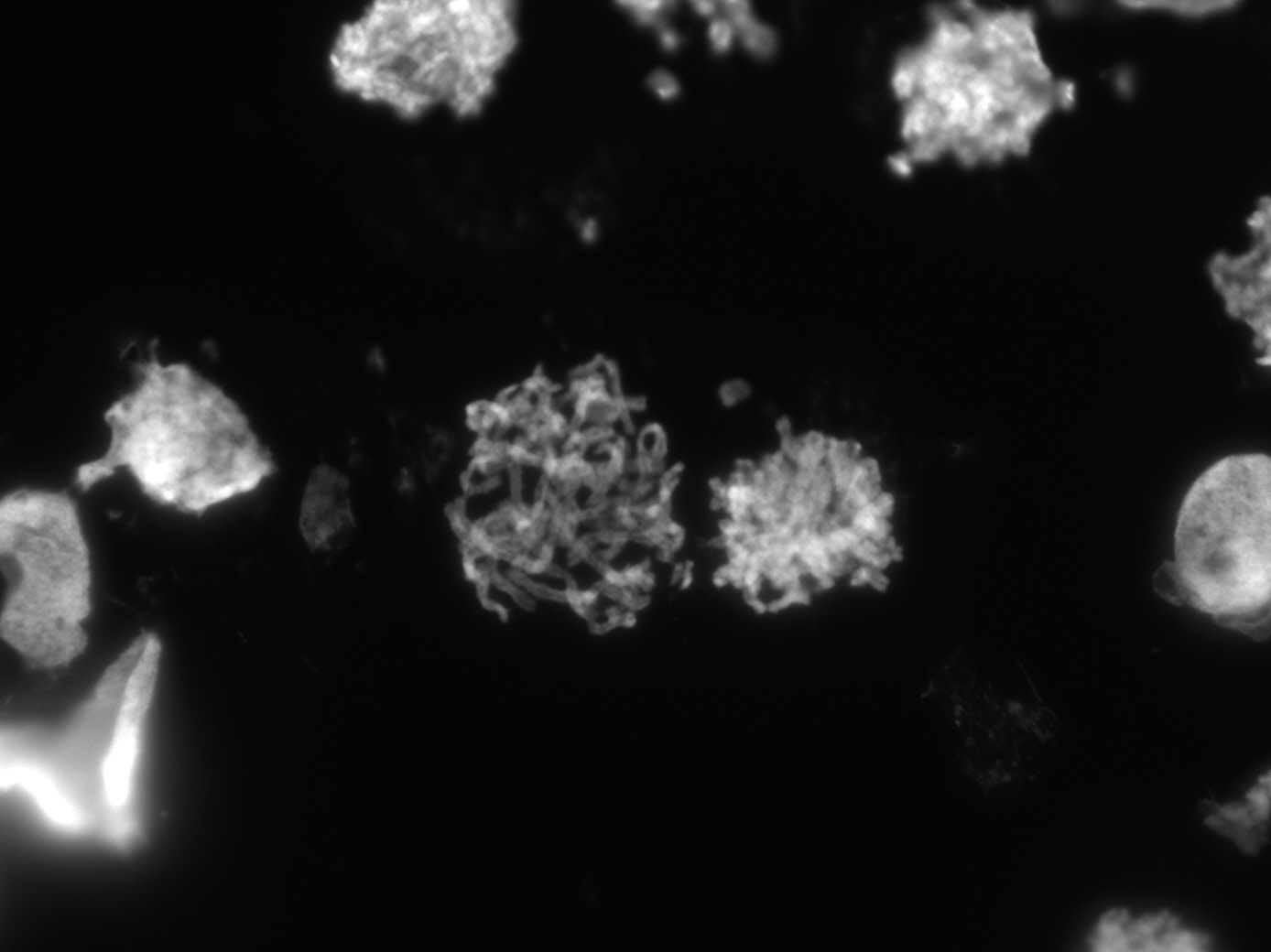

Supplement: Supplementary file 5 — Source data Fig. 3 [file 44319_2026_833_MOESM5_ESM.zip › 3A/EGFP-SMP4_Survivin_Chrom_0813_006_DAPI.tif]

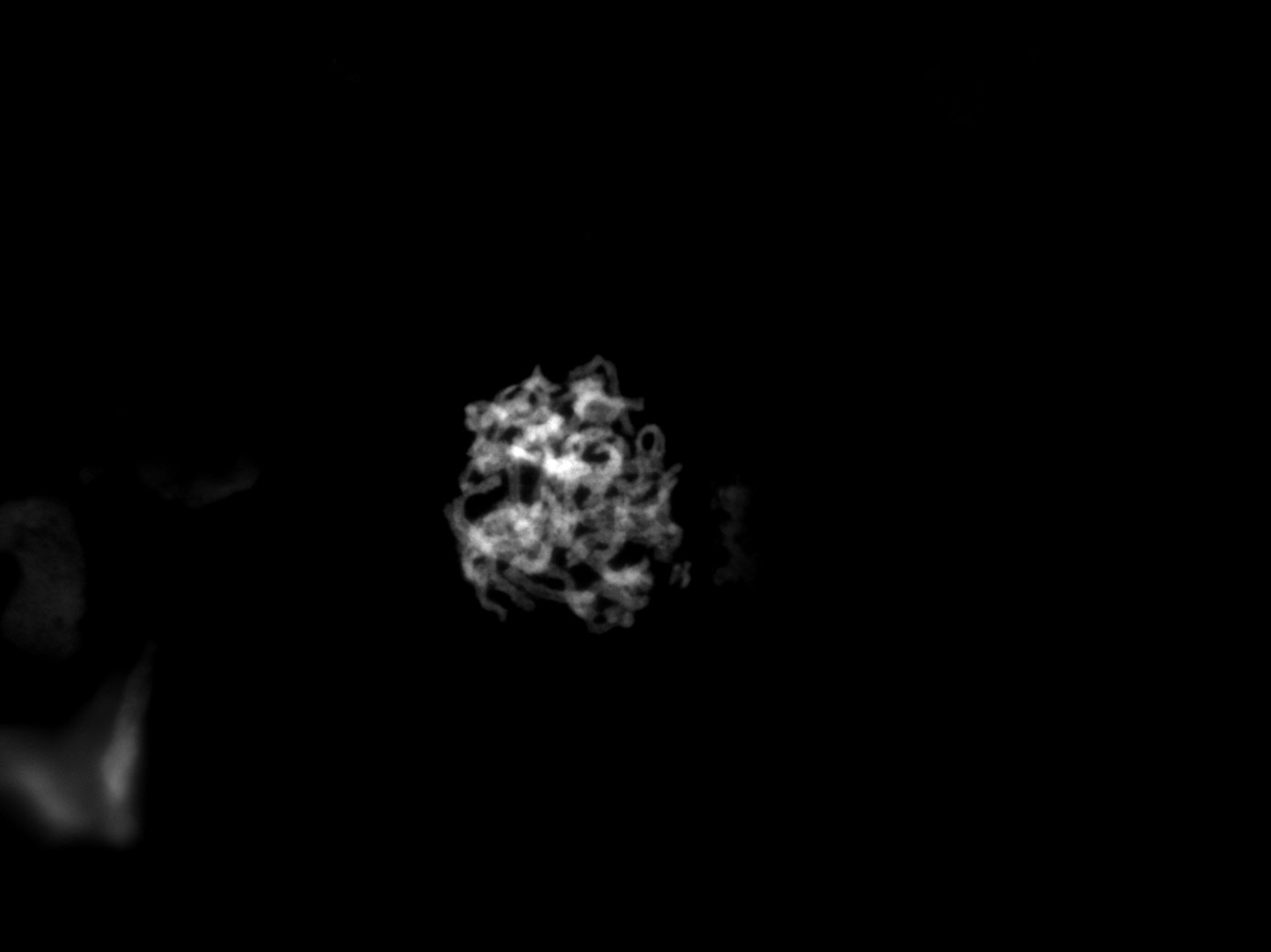

Supplement: Supplementary file 5 — Source data Fig. 3 [file 44319_2026_833_MOESM5_ESM.zip › 3A/EGFP-SMP4_Survivin_Chrom_0813_006_FiTC.tif]

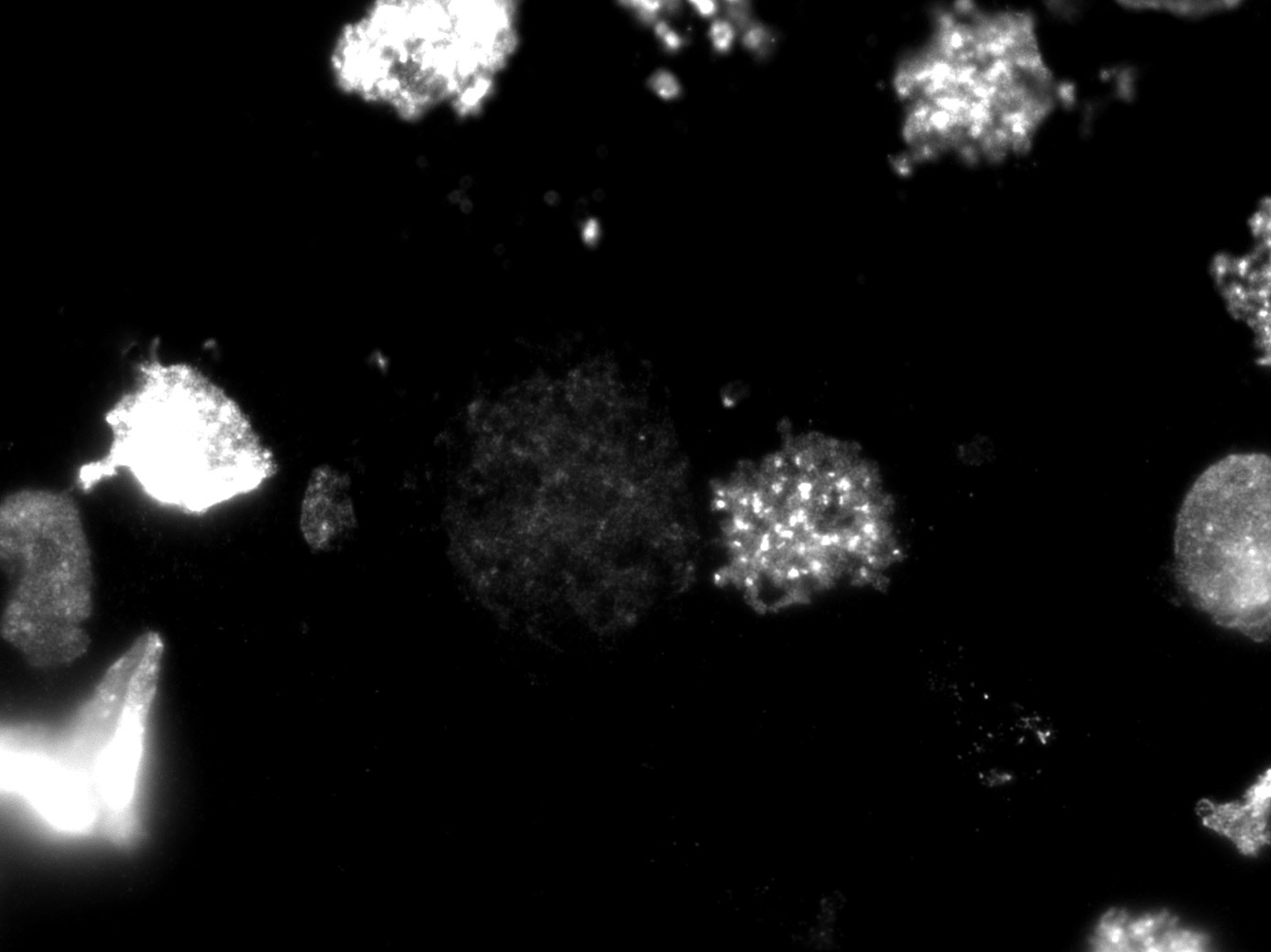

Supplement: Supplementary file 5 — Source data Fig. 3 [file 44319_2026_833_MOESM5_ESM.zip › 3A/EGFP-SMP4_Survivin_Chrom_0813_006_Texasred.tif]

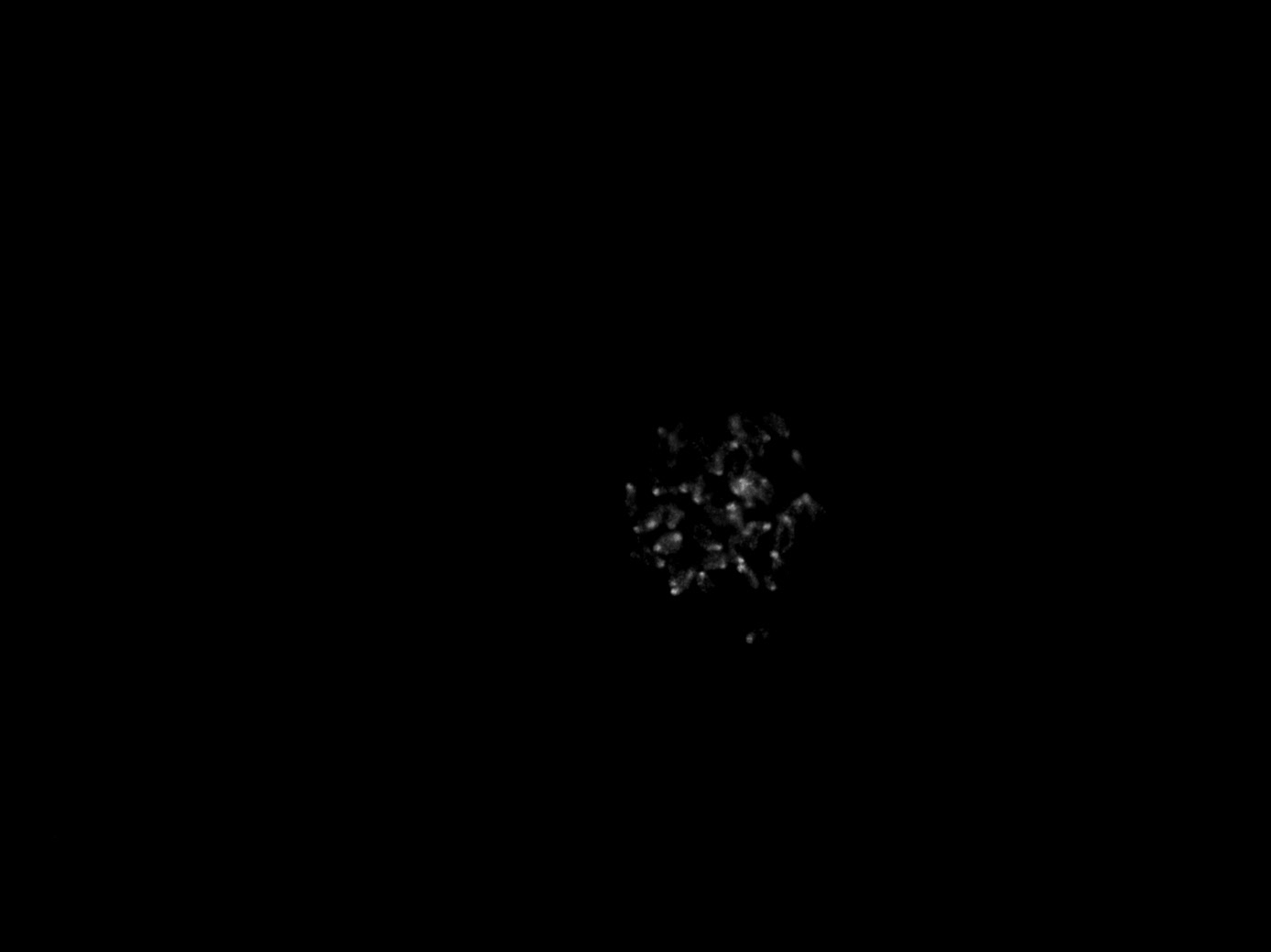

Supplement: Supplementary file 5 — Source data Fig. 3 [file 44319_2026_833_MOESM5_ESM.zip › 3B/B324CF~1.tif]

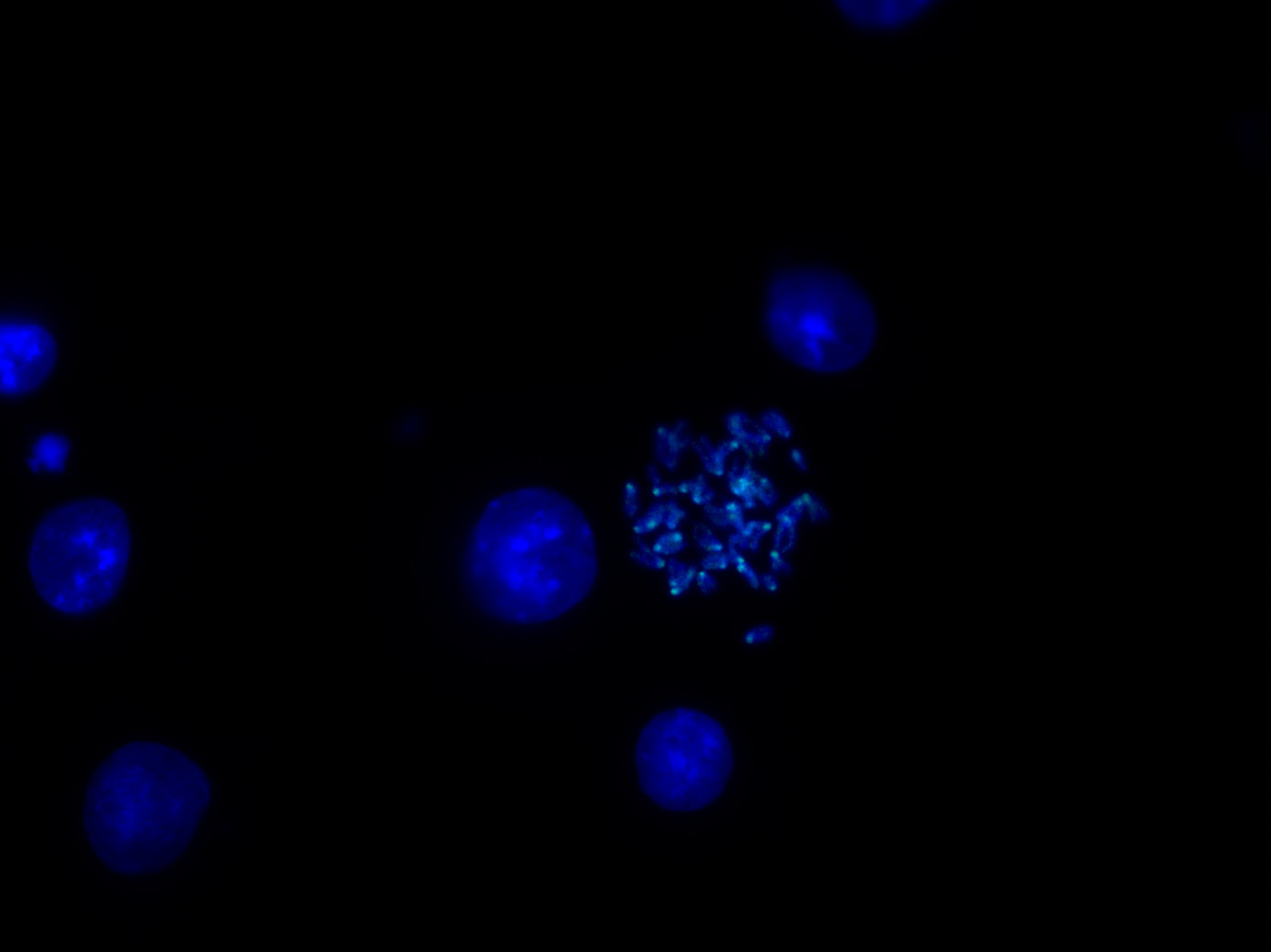

Supplement: Supplementary file 5 — Source data Fig. 3 [file 44319_2026_833_MOESM5_ESM.zip › 3B/B3___C~2.tif]

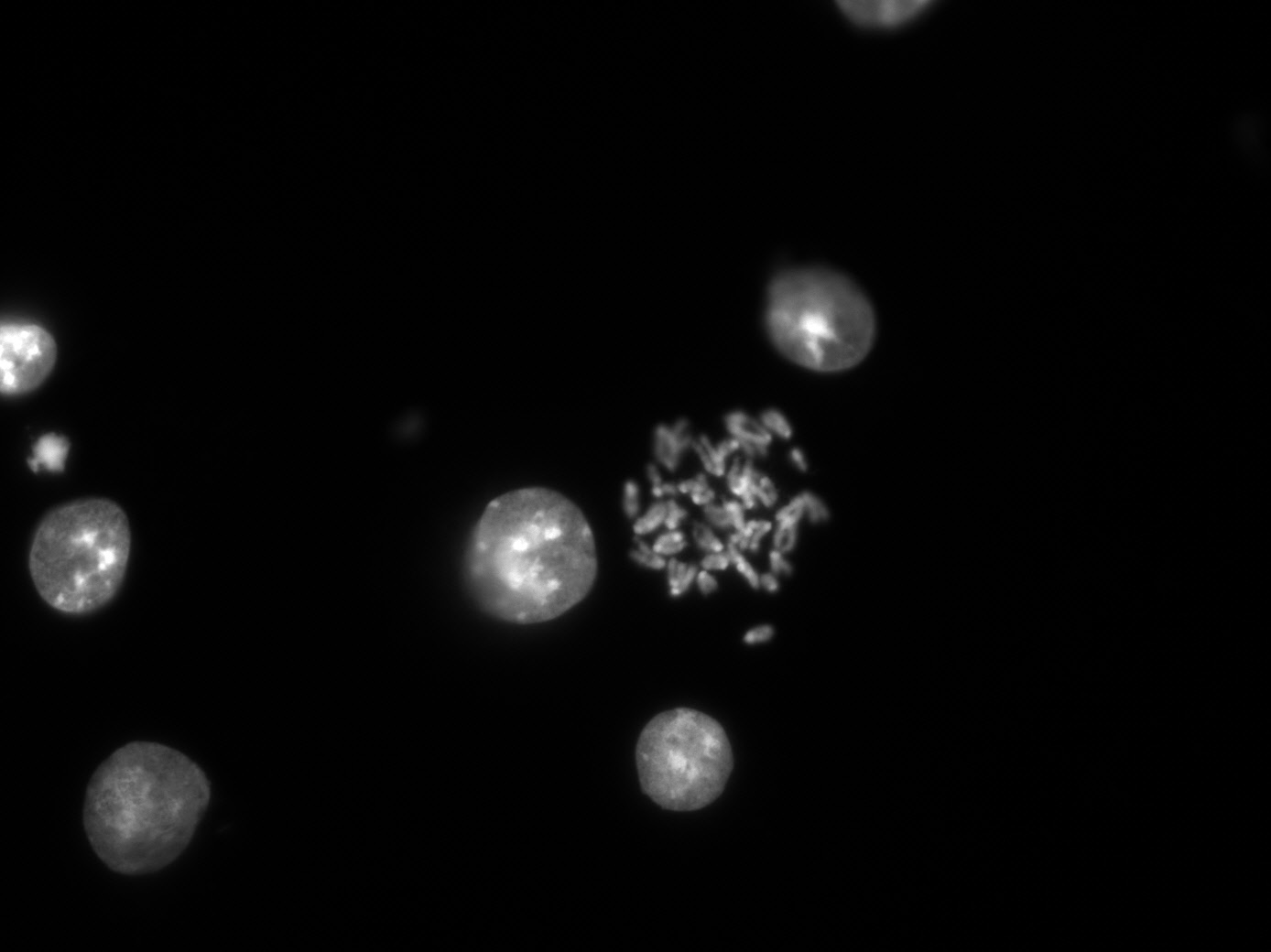

Supplement: Supplementary file 5 — Source data Fig. 3 [file 44319_2026_833_MOESM5_ESM.zip › 3B/B3___C~4.tif]

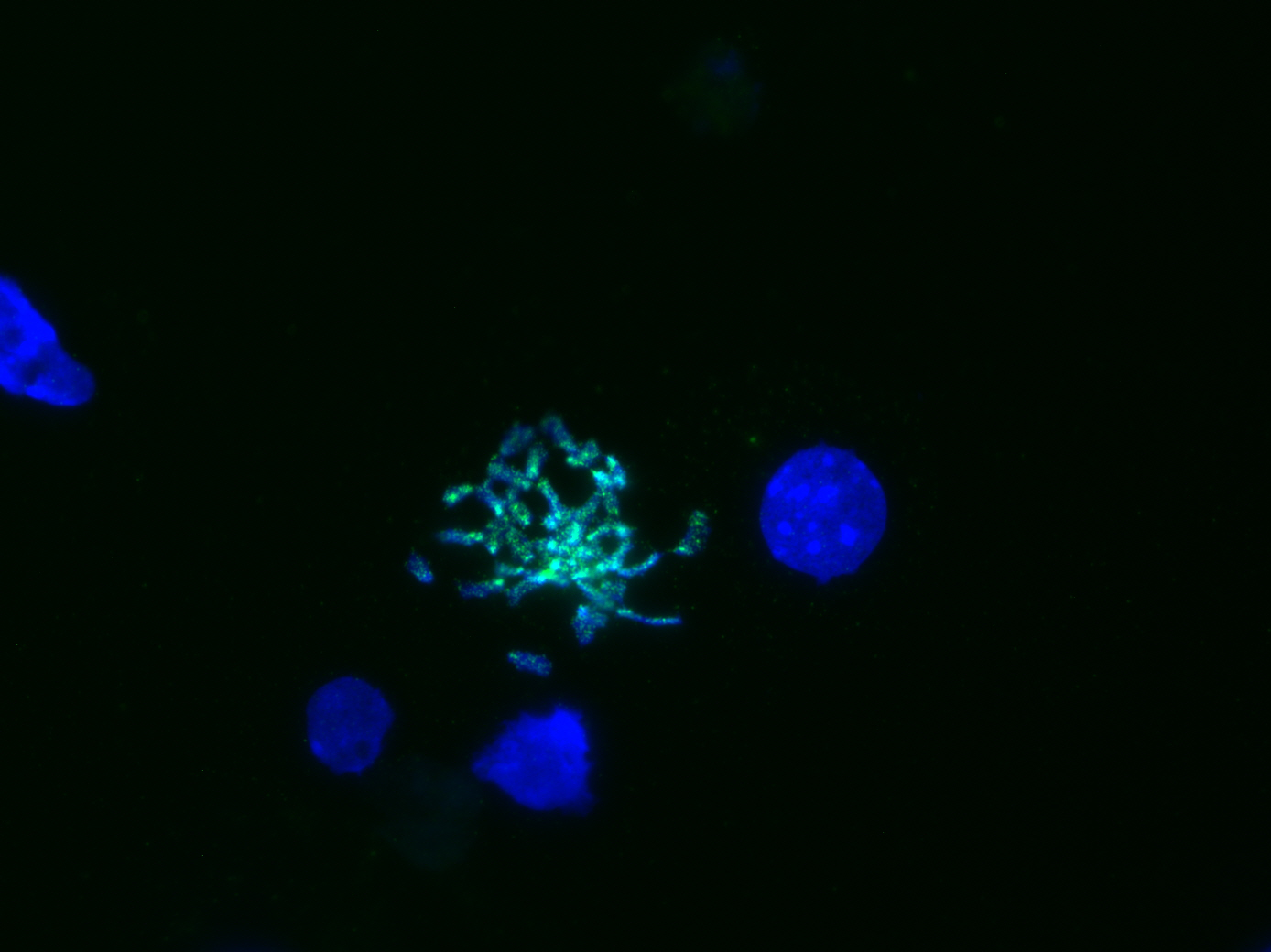

Supplement: Supplementary file 5 — Source data Fig. 3 [file 44319_2026_833_MOESM5_ESM.zip › 3B/MEF_B3~1.tif]

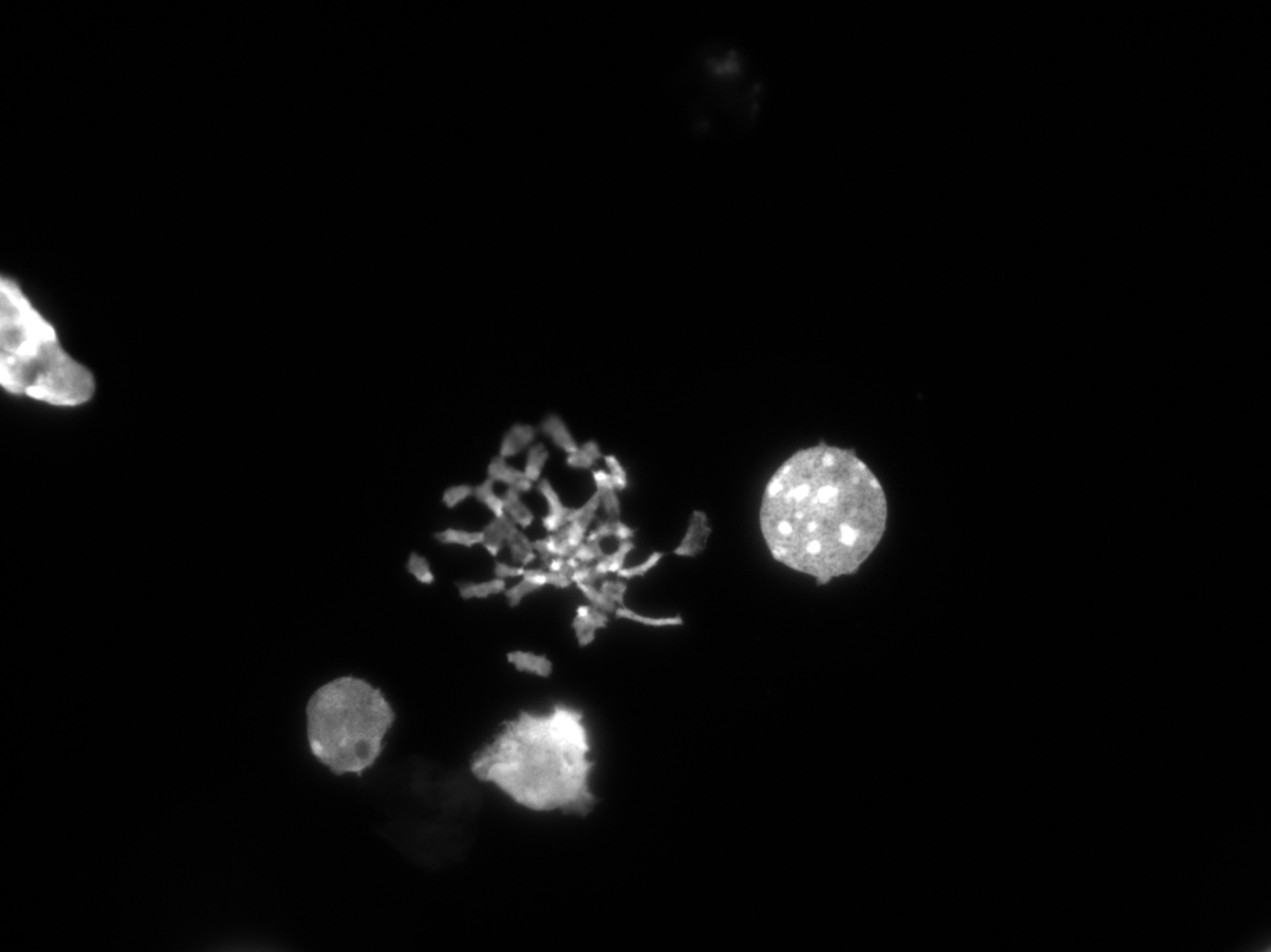

Supplement: Supplementary file 5 — Source data Fig. 3 [file 44319_2026_833_MOESM5_ESM.zip › 3B/MEF_B3~2.tif]

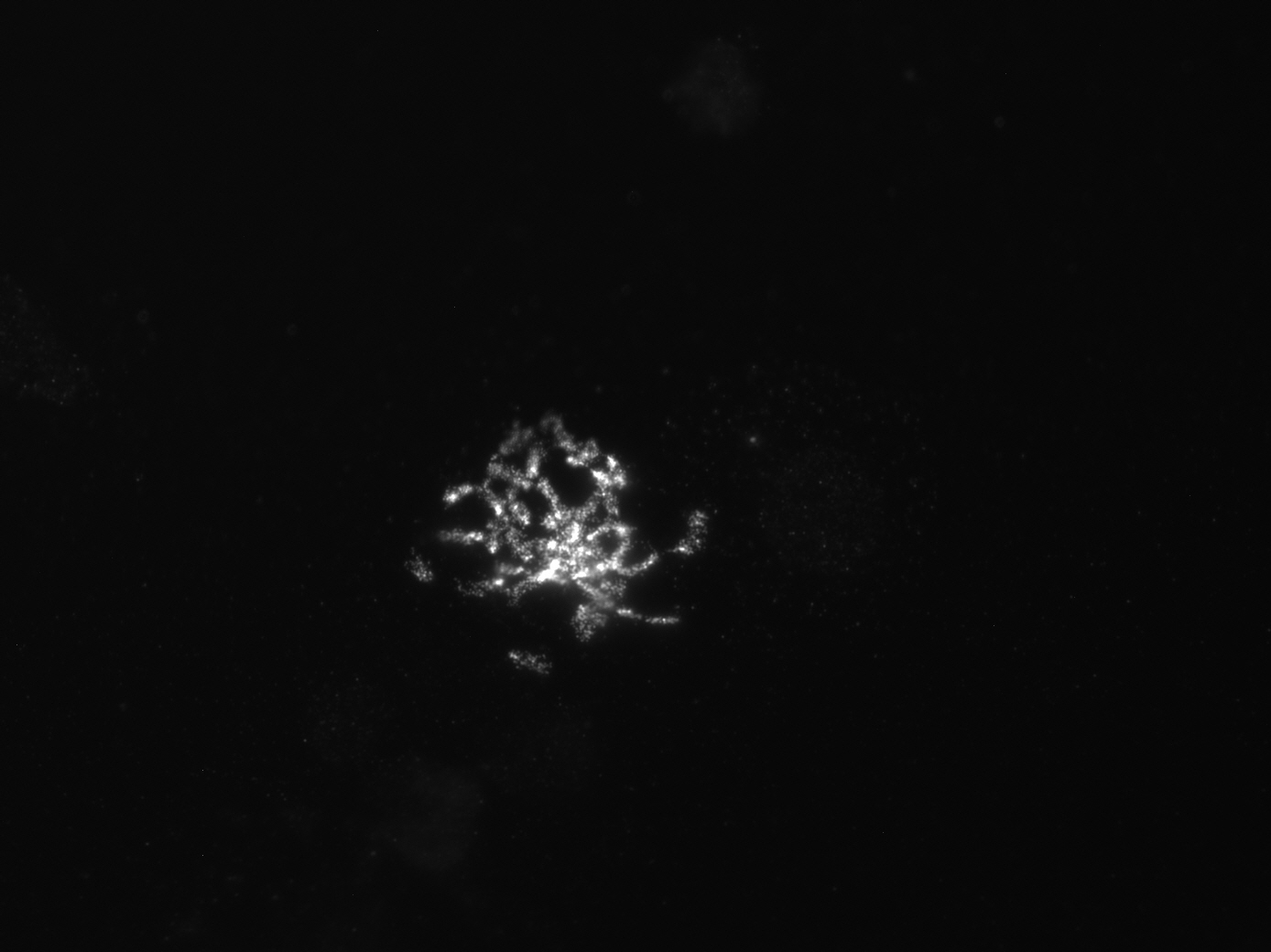

Supplement: Supplementary file 5 — Source data Fig. 3 [file 44319_2026_833_MOESM5_ESM.zip › 3B/MEF_B3~3.tif]

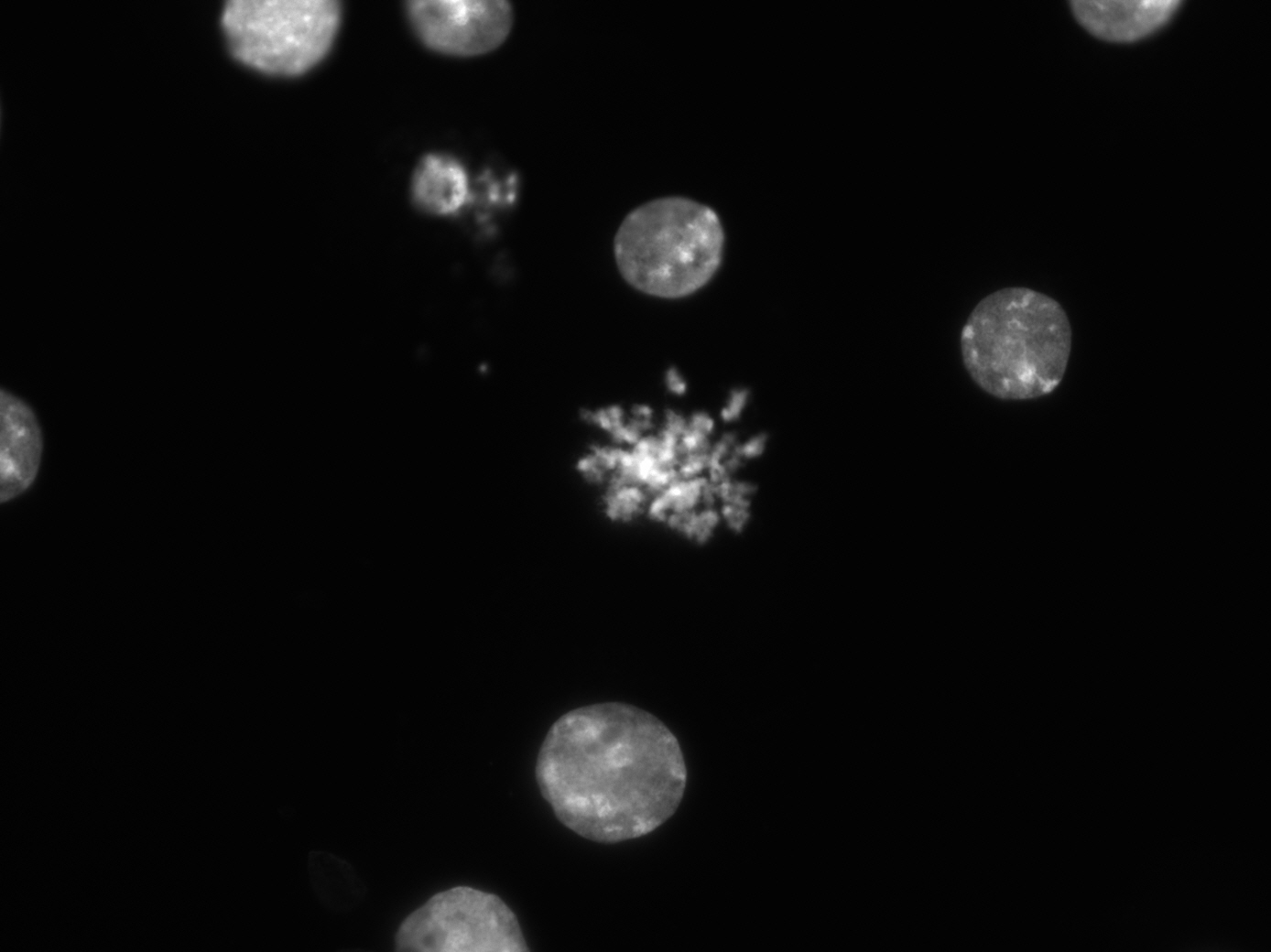

Supplement: Supplementary file 5 — Source data Fig. 3 [file 44319_2026_833_MOESM5_ESM.zip › 3C/MEF_B1++_Chrom_Sur_0807_001_z4DAPI.tif]

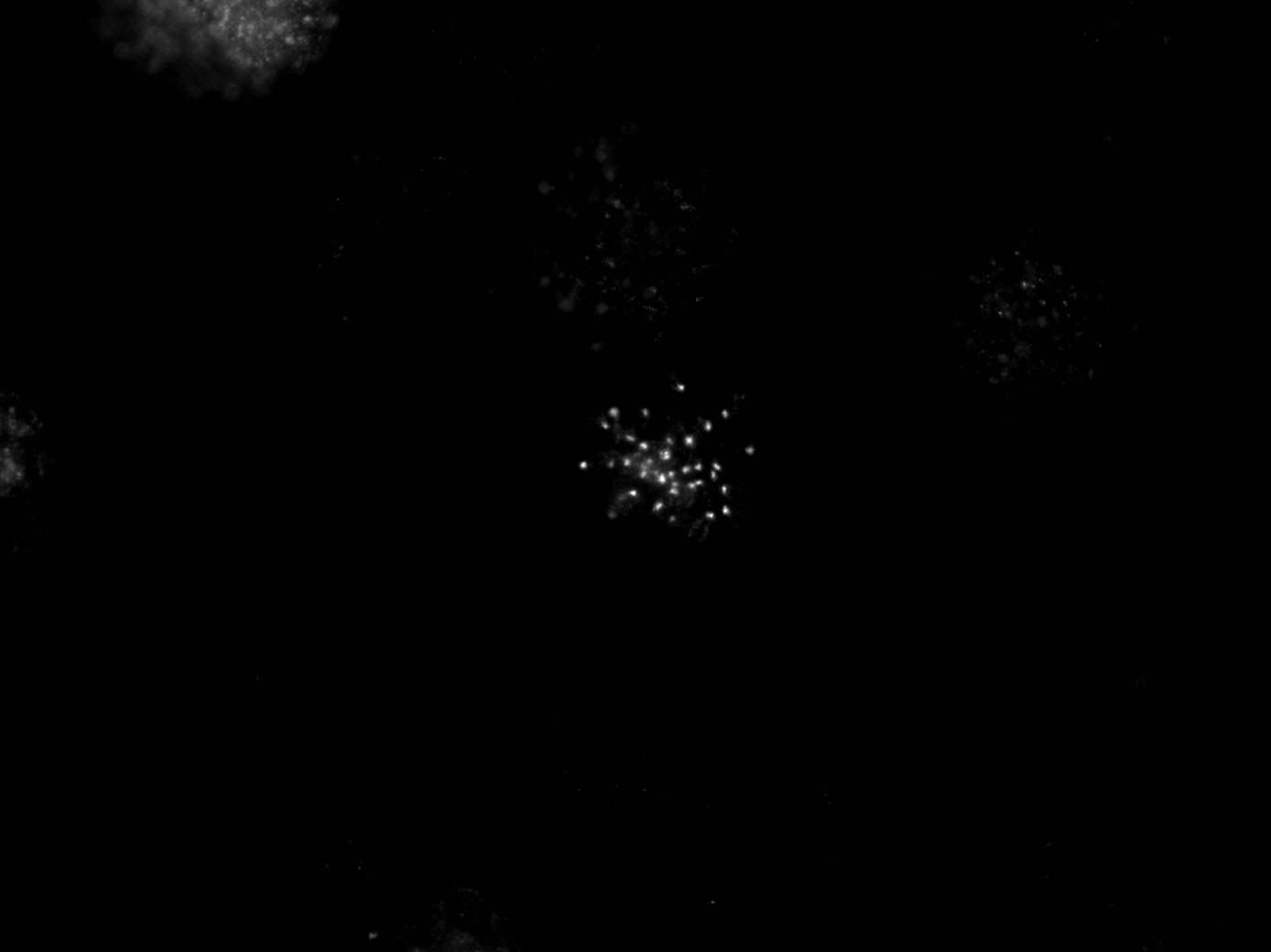

Supplement: Supplementary file 5 — Source data Fig. 3 [file 44319_2026_833_MOESM5_ESM.zip › 3C/MEF_B1++_Chrom_Sur_0807_001_z4FiTC.tif]

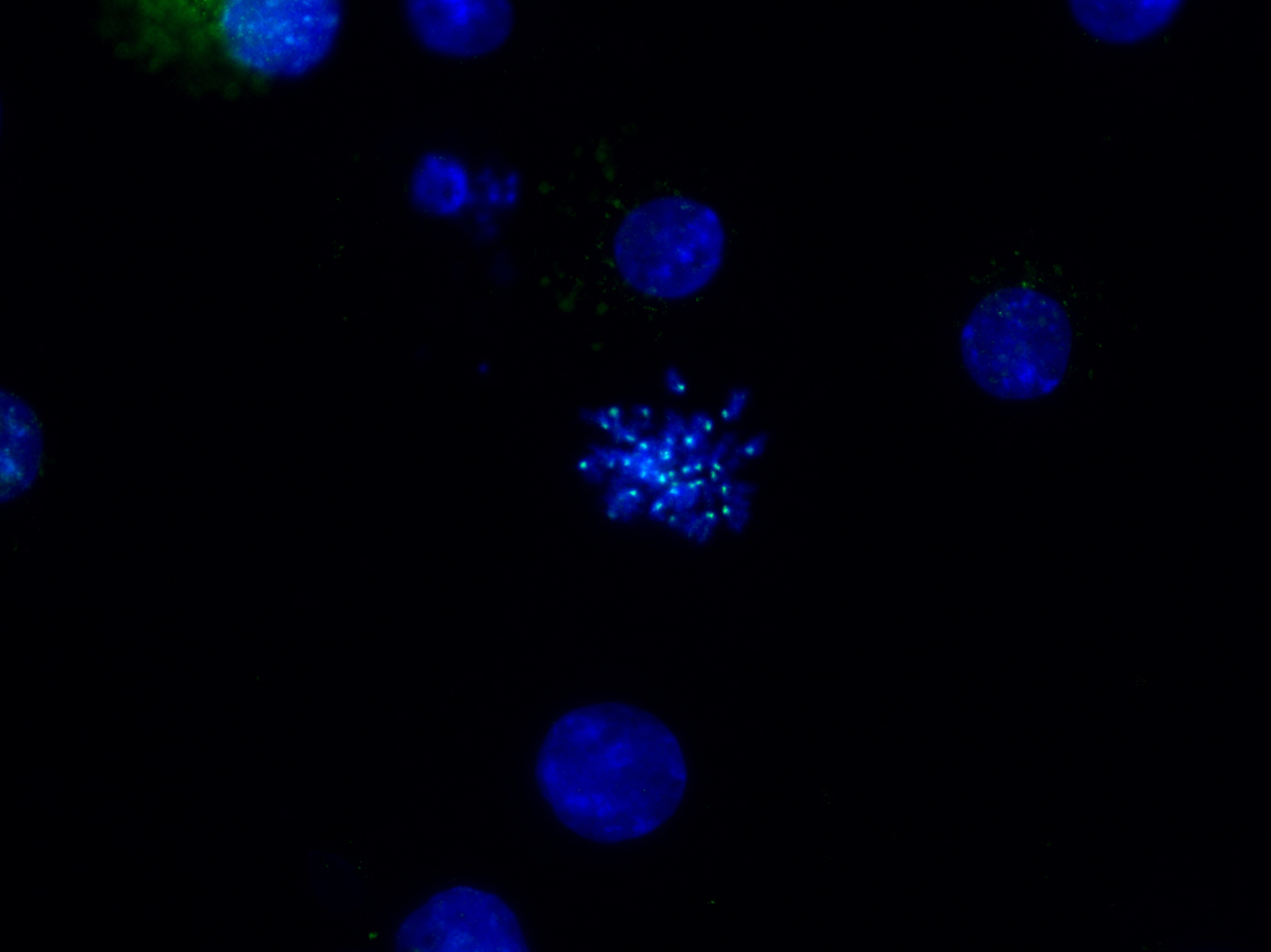

Supplement: Supplementary file 5 — Source data Fig. 3 [file 44319_2026_833_MOESM5_ESM.zip › 3C/MEF_B1++_Chrom_Sur_0807_001_z4Merg.tif]

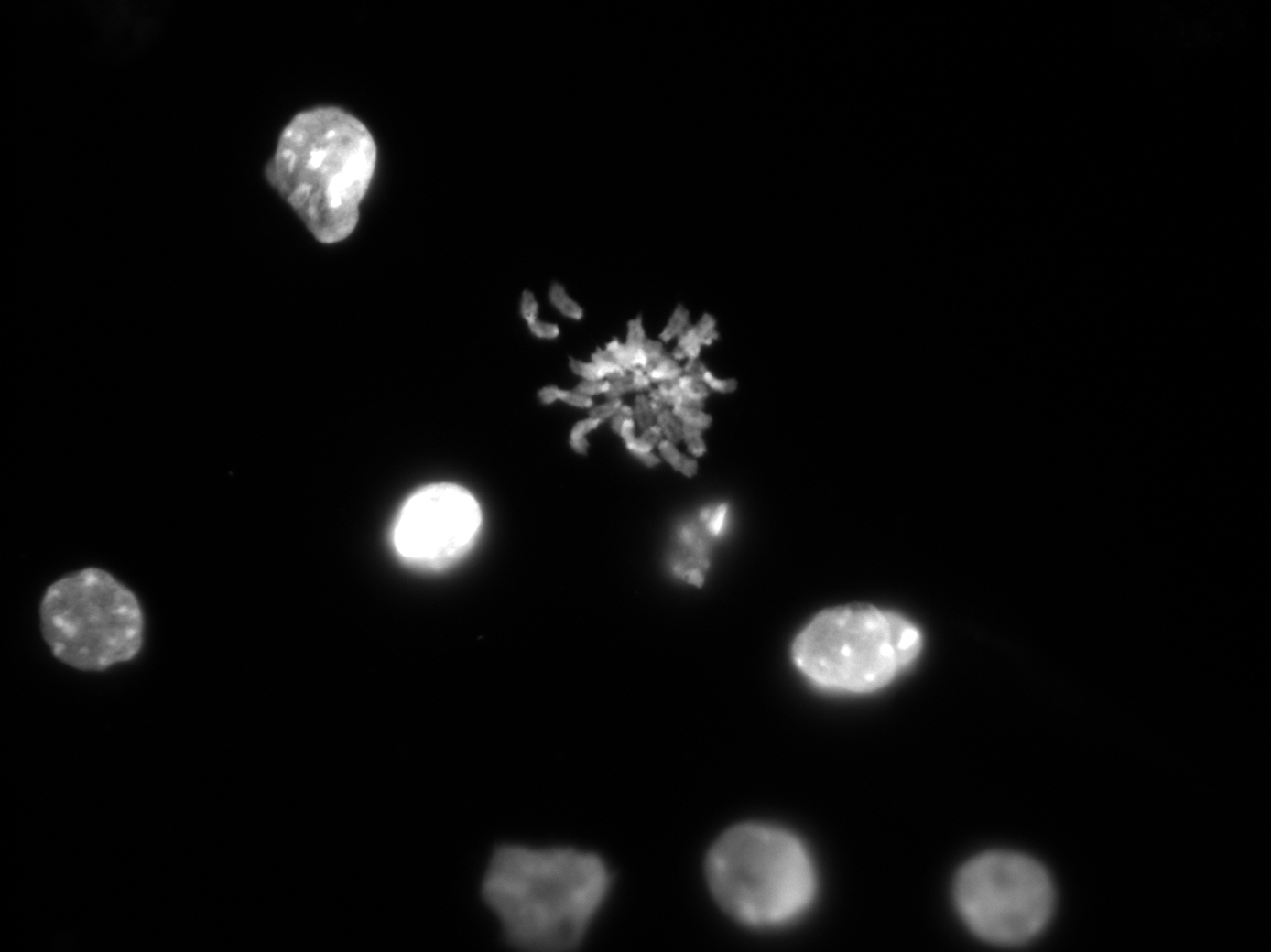

Supplement: Supplementary file 5 — Source data Fig. 3 [file 44319_2026_833_MOESM5_ESM.zip › 3C/MEF_B3--_Chrom_Sur_0813_018_DAPI.tif]

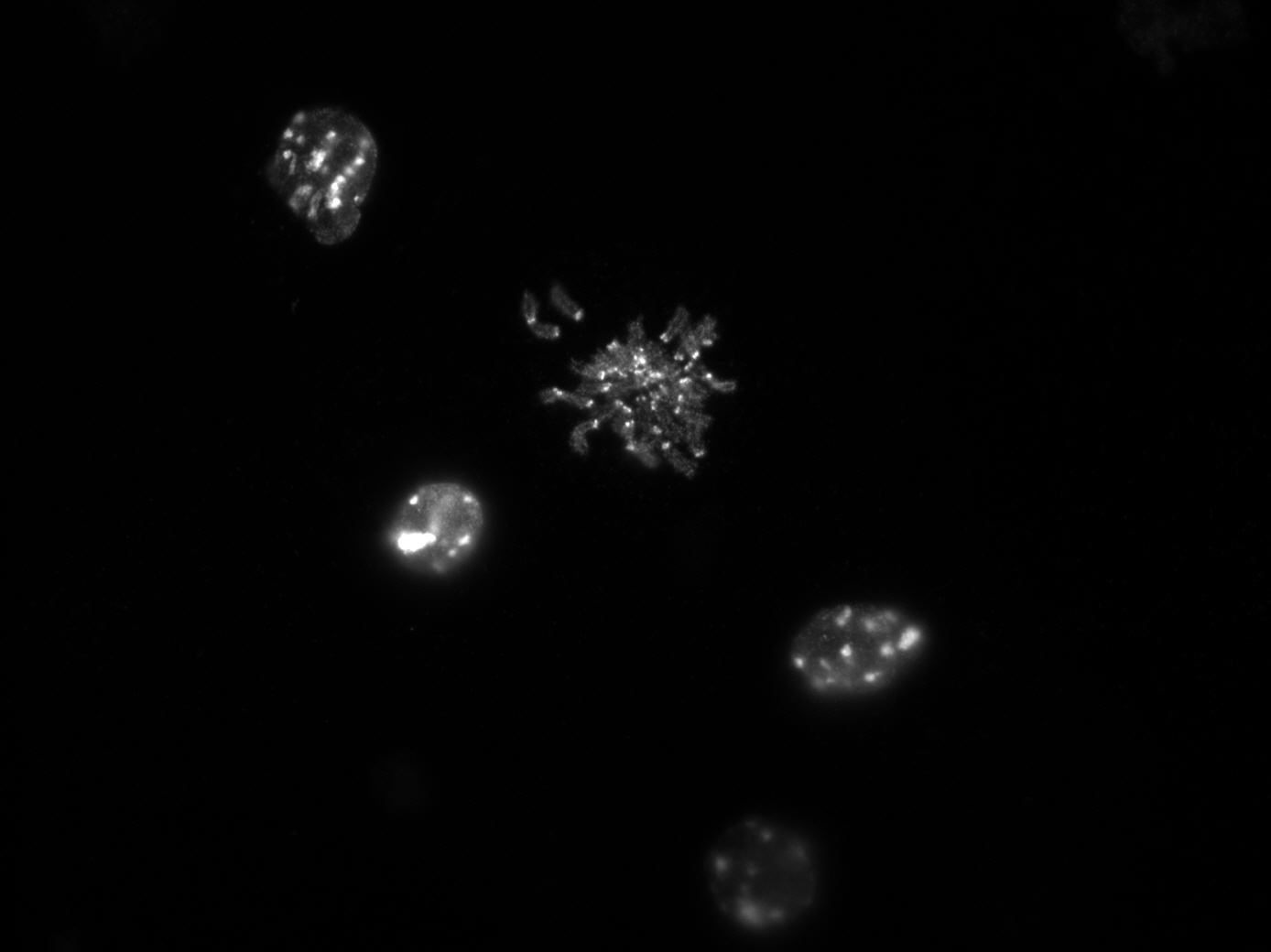

Supplement: Supplementary file 5 — Source data Fig. 3 [file 44319_2026_833_MOESM5_ESM.zip › 3C/MEF_B3--_Chrom_Sur_0813_018_FiTC.tif]

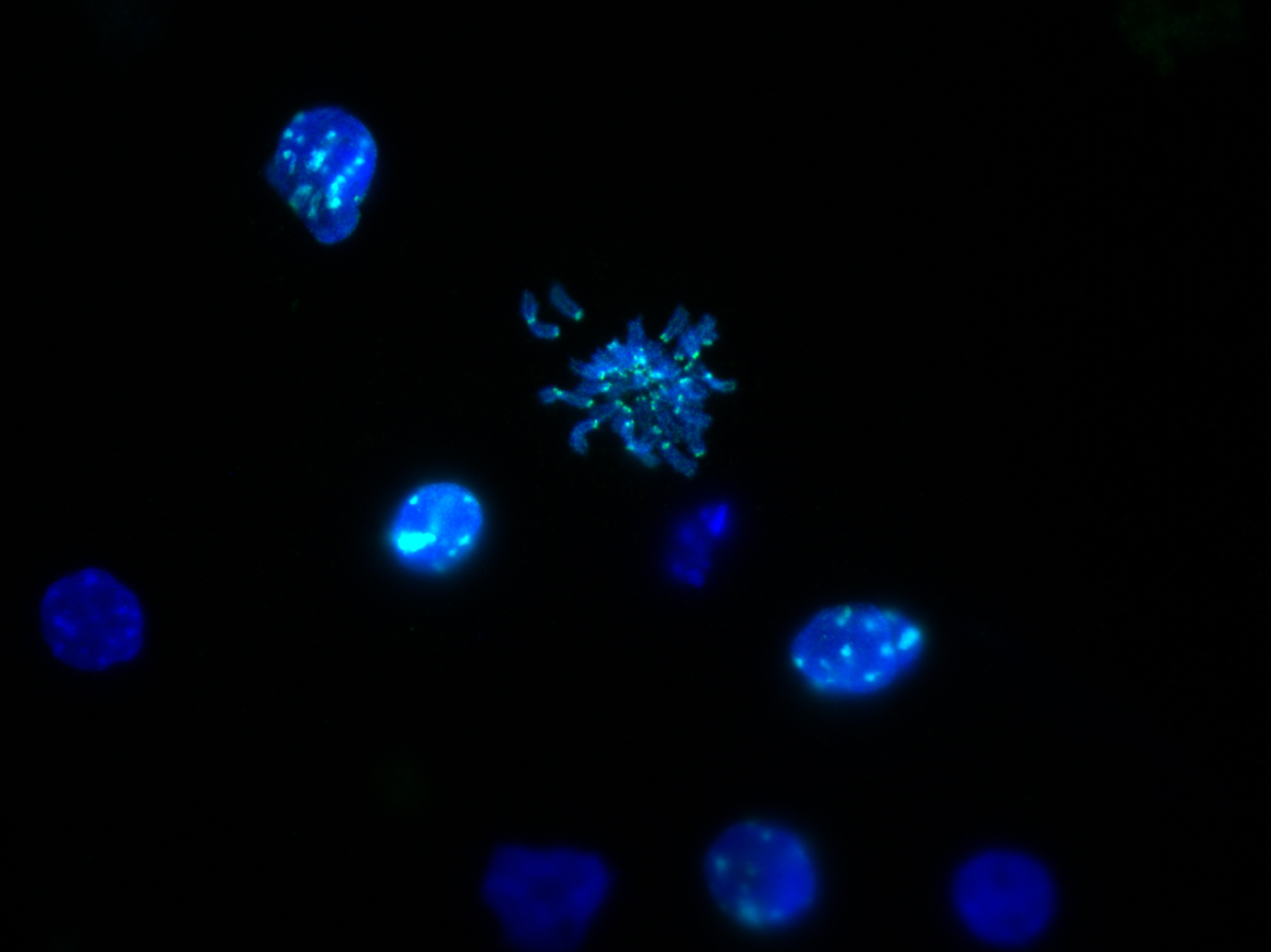

Supplement: Supplementary file 5 — Source data Fig. 3 [file 44319_2026_833_MOESM5_ESM.zip › 3C/MEF_B3--_Chrom_Sur_0813_018_Merg.tif]

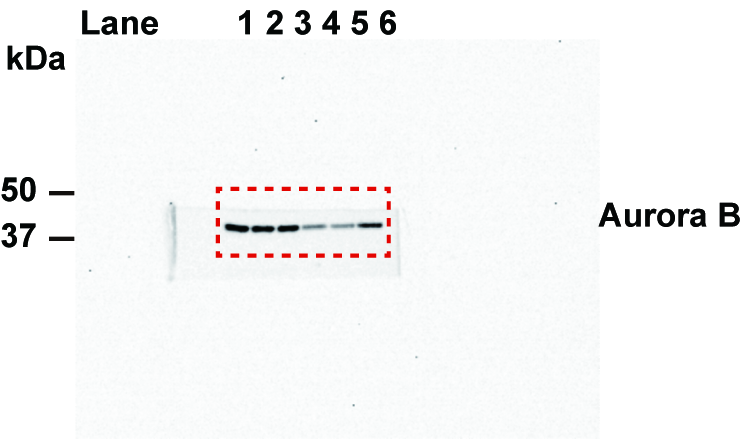

Supplement: Supplementary file 5 — Source data Fig. 3 [file 44319_2026_833_MOESM5_ESM.zip › 3D/Aurora B/Origin 3D Aurora B.tif]

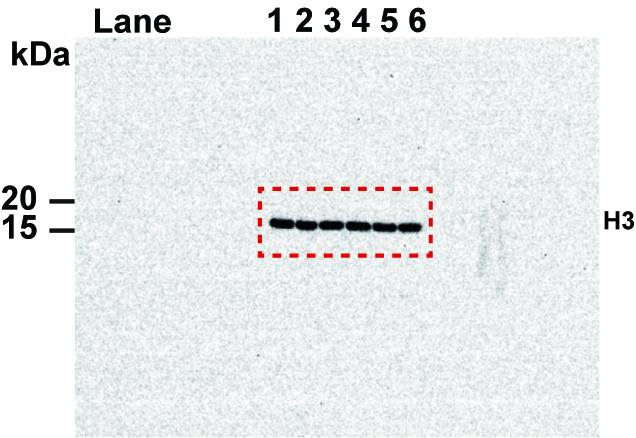

Supplement: Supplementary file 5 — Source data Fig. 3 [file 44319_2026_833_MOESM5_ESM.zip › 3D/H3/Origin 3D H3.tif]

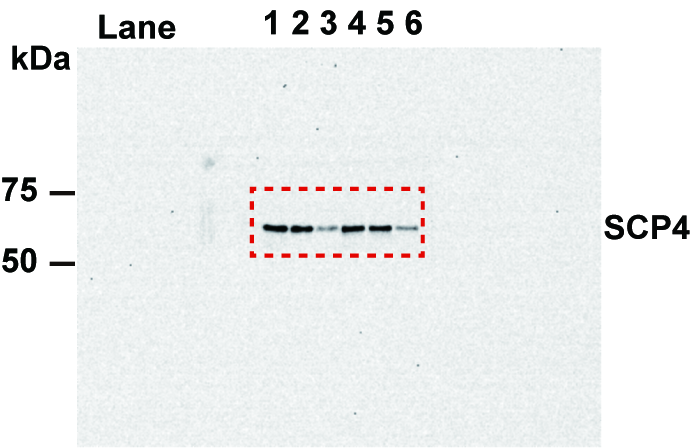

Supplement: Supplementary file 5 — Source data Fig. 3 [file 44319_2026_833_MOESM5_ESM.zip › 3D/SCP4/Origin 3D SCP4.tif]

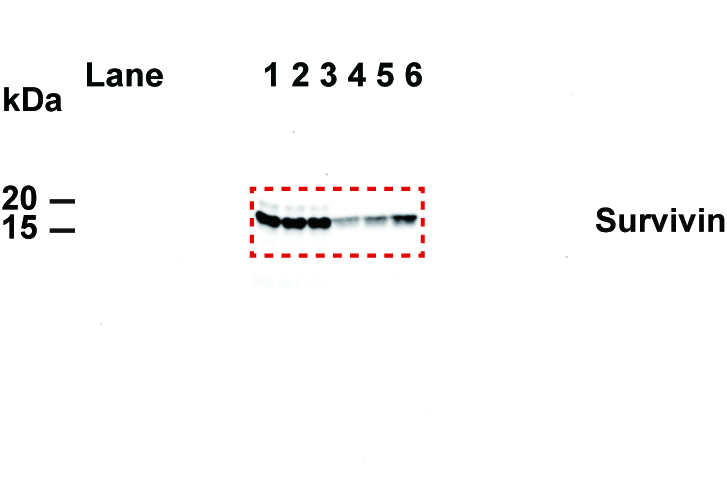

Supplement: Supplementary file 5 — Source data Fig. 3 [file 44319_2026_833_MOESM5_ESM.zip › 3D/Survivin/Origin 3D Survivin.tif]

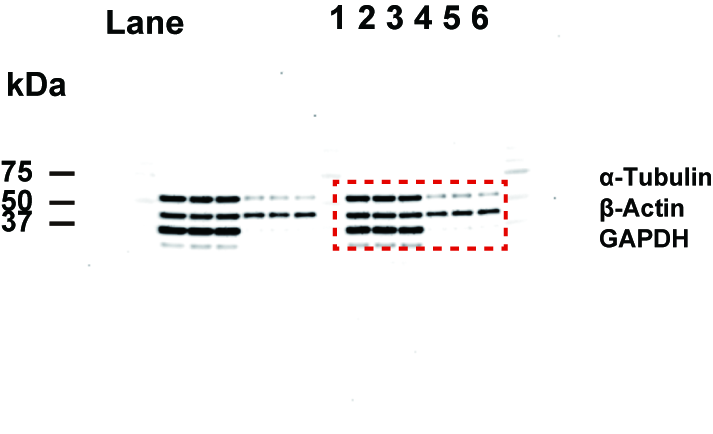

Supplement: Supplementary file 5 — Source data Fig. 3 [file 44319_2026_833_MOESM5_ESM.zip › 3D/Tubulin Actin GAPDH/Origin 3D Tubulin Actin GAPDH.tif]

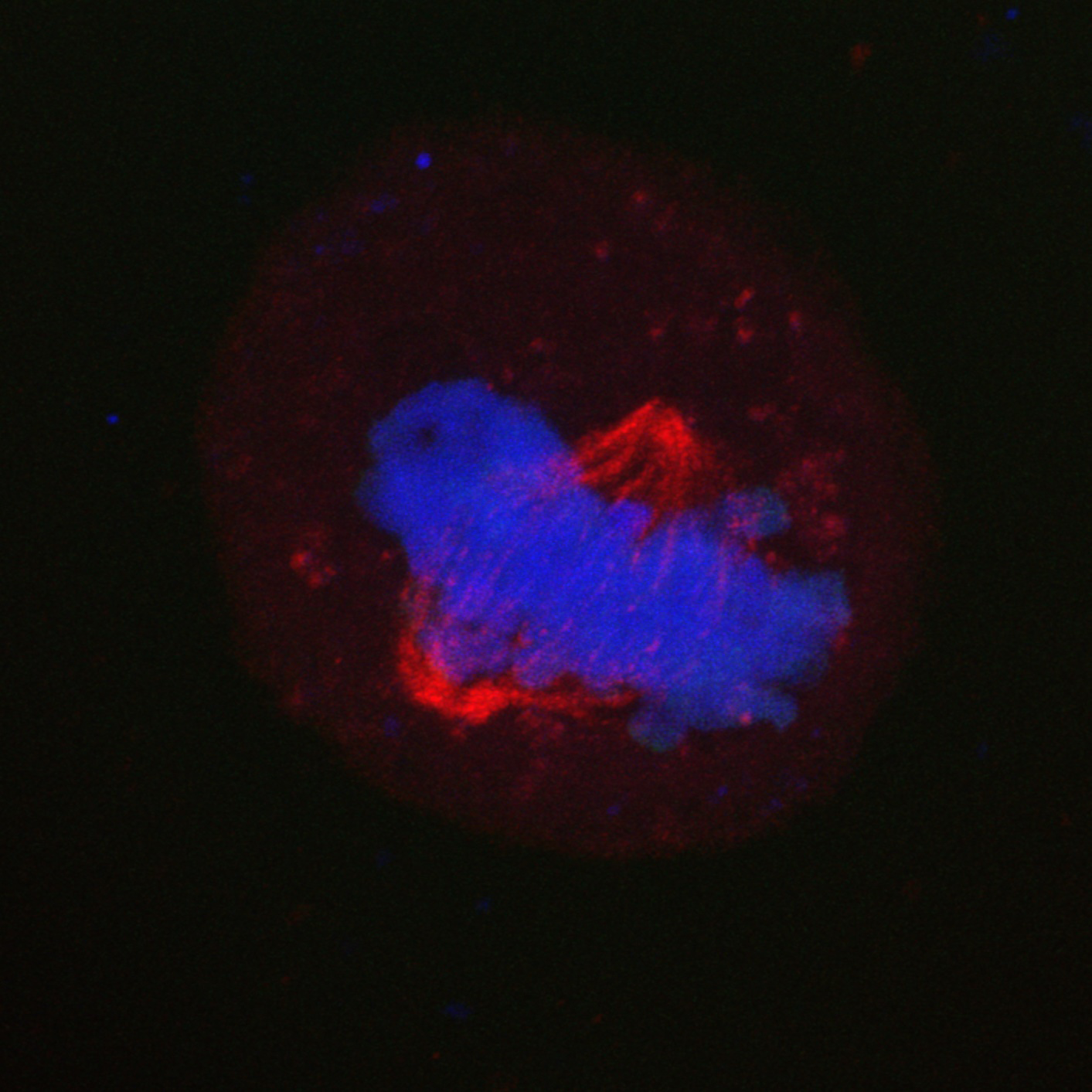

Supplement: Supplementary file 6 — Source data Fig. 4 [file 44319_2026_833_MOESM6_ESM.zip › 4A/SCP4 OE Metaphase-Bend spindle/Experiment-4089-Airyscan Processing-03-Orthogonal Projection-04_c1-3.tif]

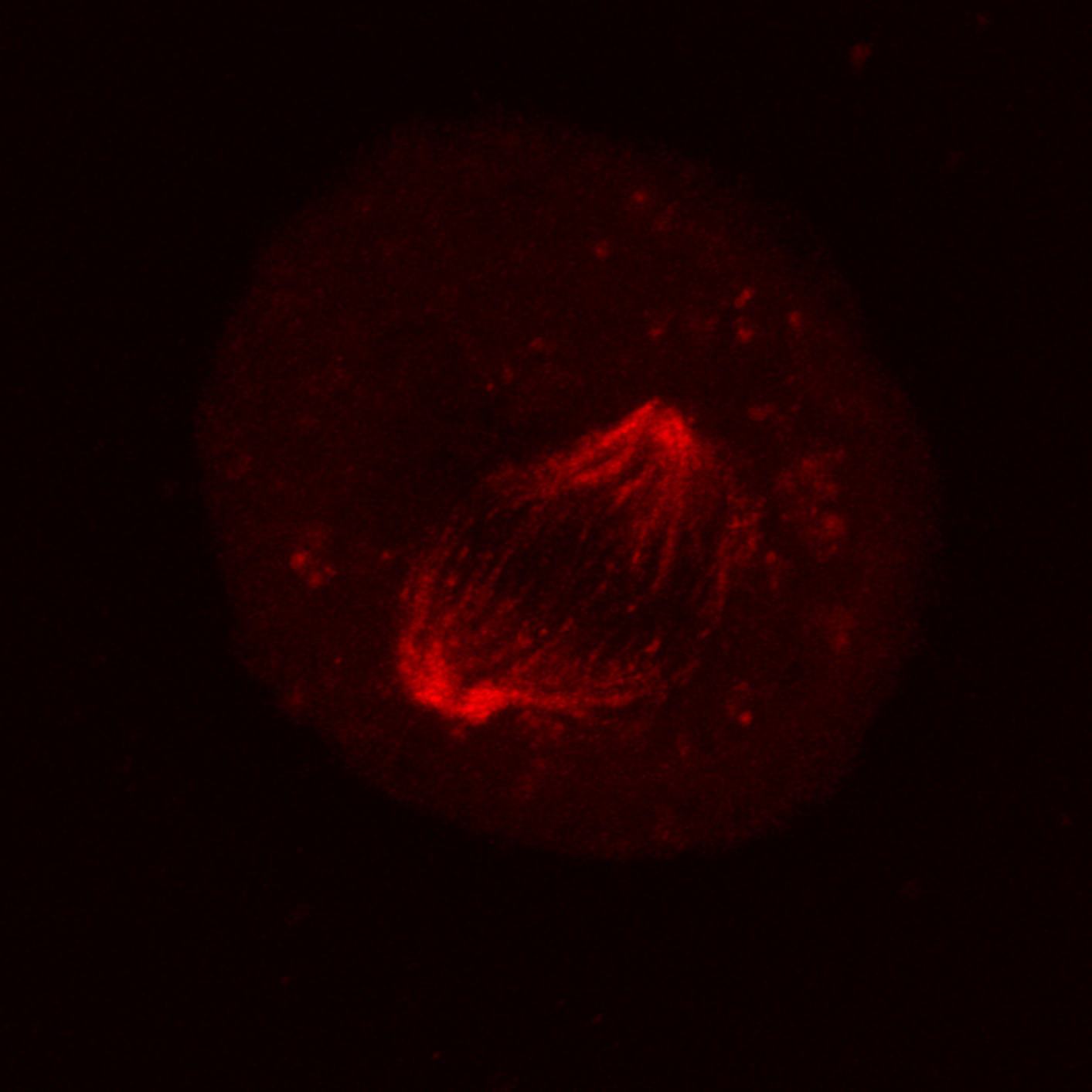

Supplement: Supplementary file 6 — Source data Fig. 4 [file 44319_2026_833_MOESM6_ESM.zip › 4A/SCP4 OE Metaphase-Bend spindle/Experiment-4089-Airyscan Processing-03-Orthogonal Projection-04_c1.tif]

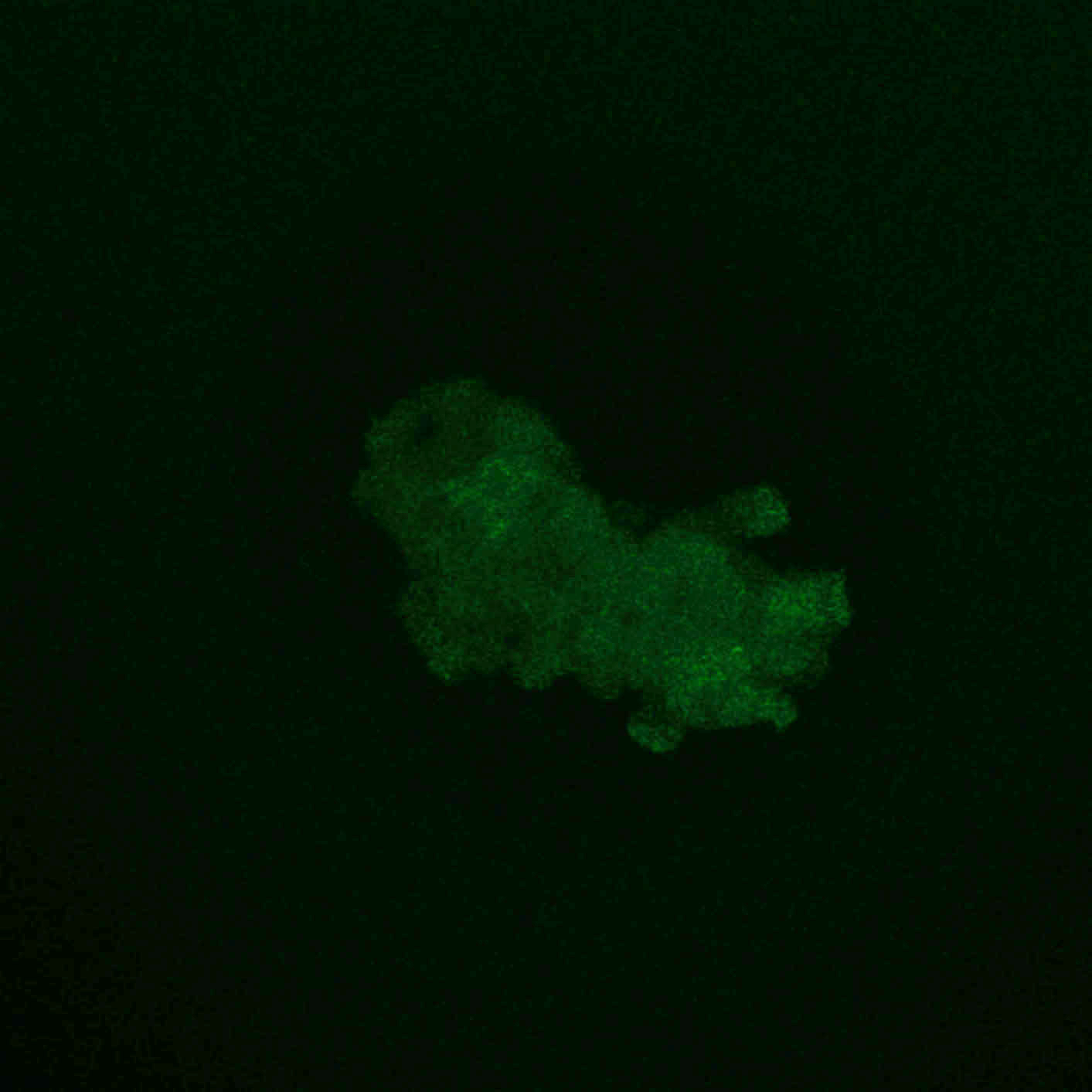

Supplement: Supplementary file 6 — Source data Fig. 4 [file 44319_2026_833_MOESM6_ESM.zip › 4A/SCP4 OE Metaphase-Bend spindle/Experiment-4089-Airyscan Processing-03-Orthogonal Projection-04_c2.tif]

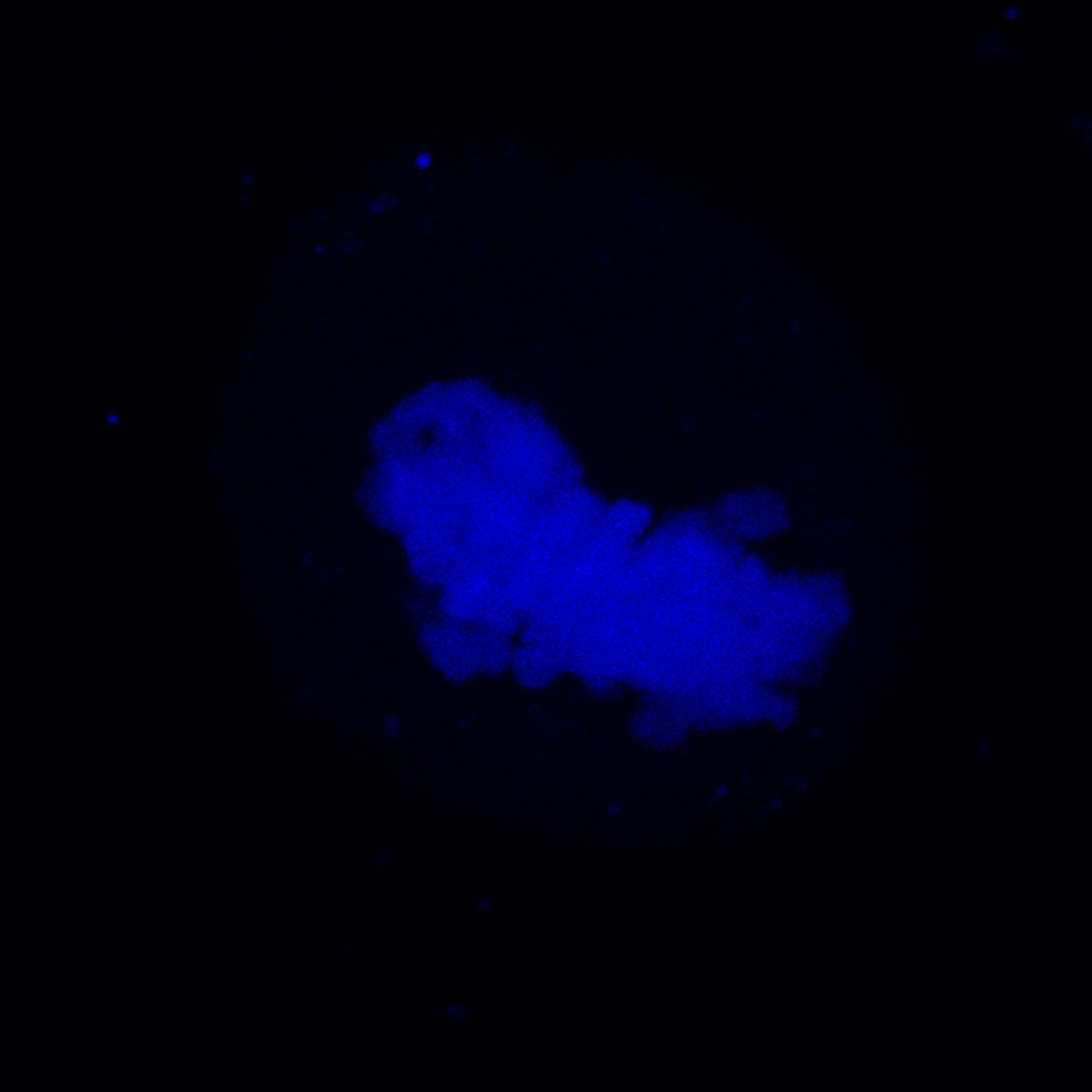

Supplement: Supplementary file 6 — Source data Fig. 4 [file 44319_2026_833_MOESM6_ESM.zip › 4A/SCP4 OE Metaphase-Bend spindle/Experiment-4089-Airyscan Processing-03-Orthogonal Projection-04_c3.tif]

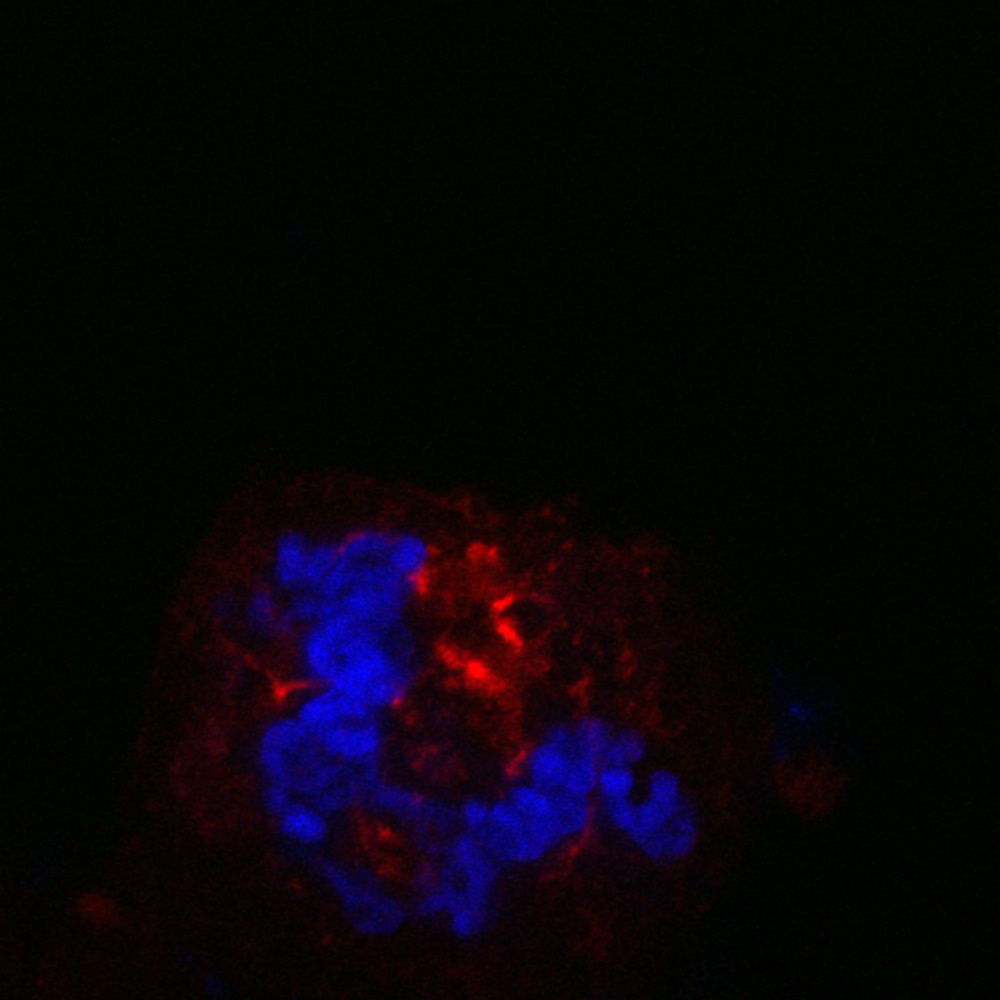

Supplement: Supplementary file 7 — Source data Fig. 5 [file 44319_2026_833_MOESM7_ESM.zip › 5B/shSCP4-H3T3A-shH3 Anaphase-2/Experiment-4576-Airyscan Processing-20-Orthogonal Projection-01/Experiment-4576-Airyscan Processing-20_z08c1-3.tif]

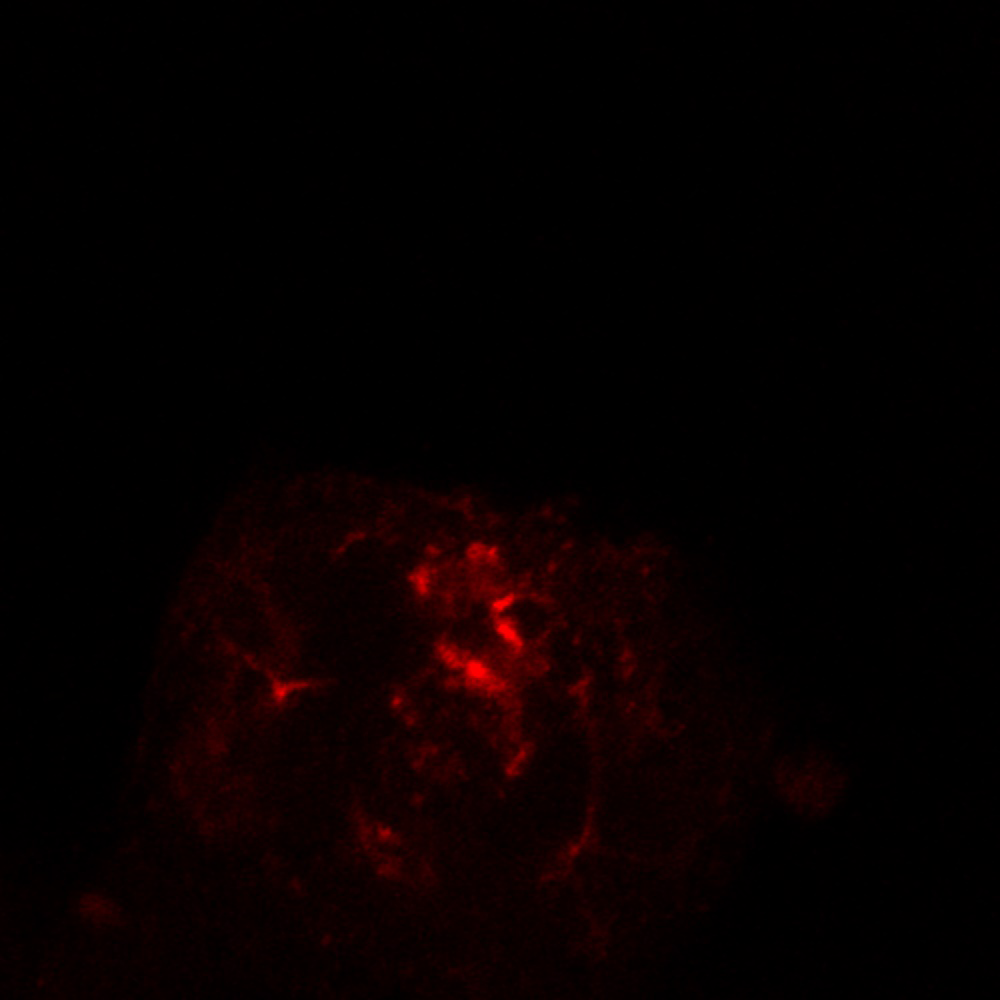

Supplement: Supplementary file 7 — Source data Fig. 5 [file 44319_2026_833_MOESM7_ESM.zip › 5B/shSCP4-H3T3A-shH3 Anaphase-2/Experiment-4576-Airyscan Processing-20-Orthogonal Projection-01/Experiment-4576-Airyscan Processing-20_z08c1.tif]

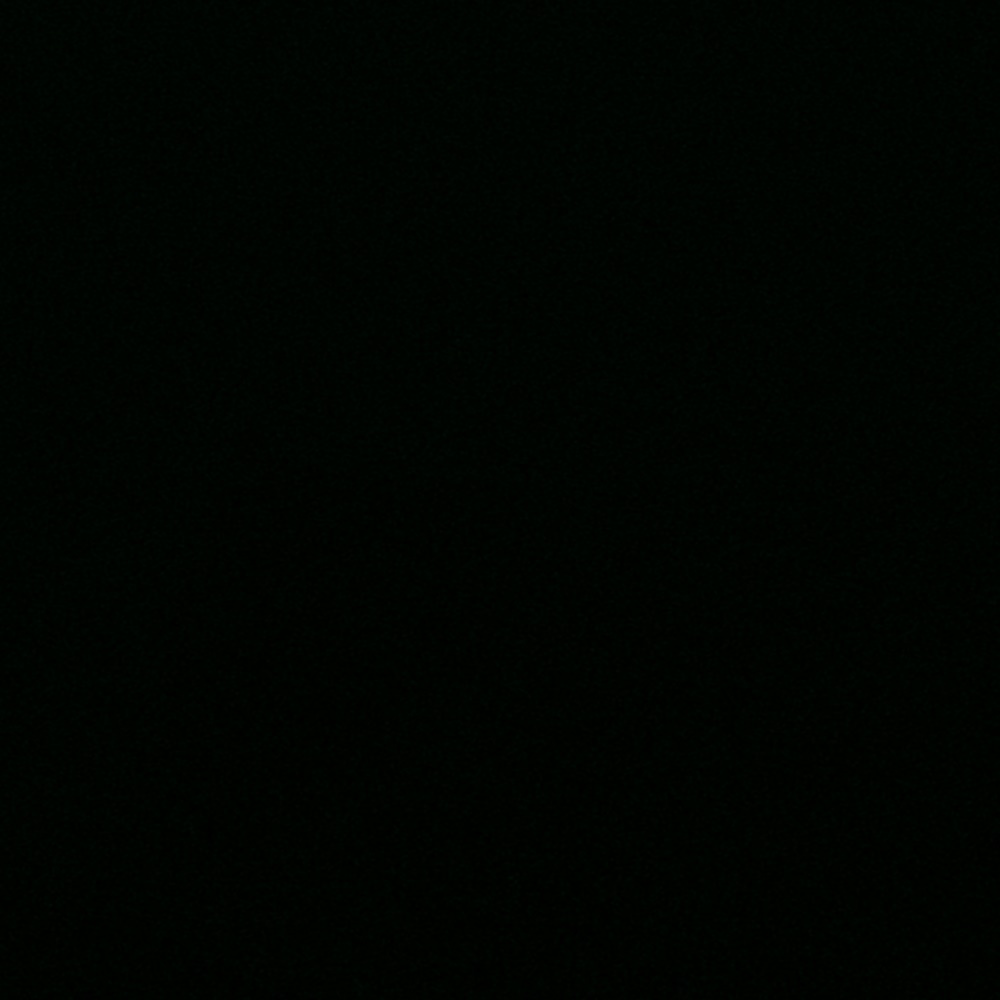

Supplement: Supplementary file 7 — Source data Fig. 5 [file 44319_2026_833_MOESM7_ESM.zip › 5B/shSCP4-H3T3A-shH3 Anaphase-2/Experiment-4576-Airyscan Processing-20-Orthogonal Projection-01/Experiment-4576-Airyscan Processing-20_z08c2.tif]

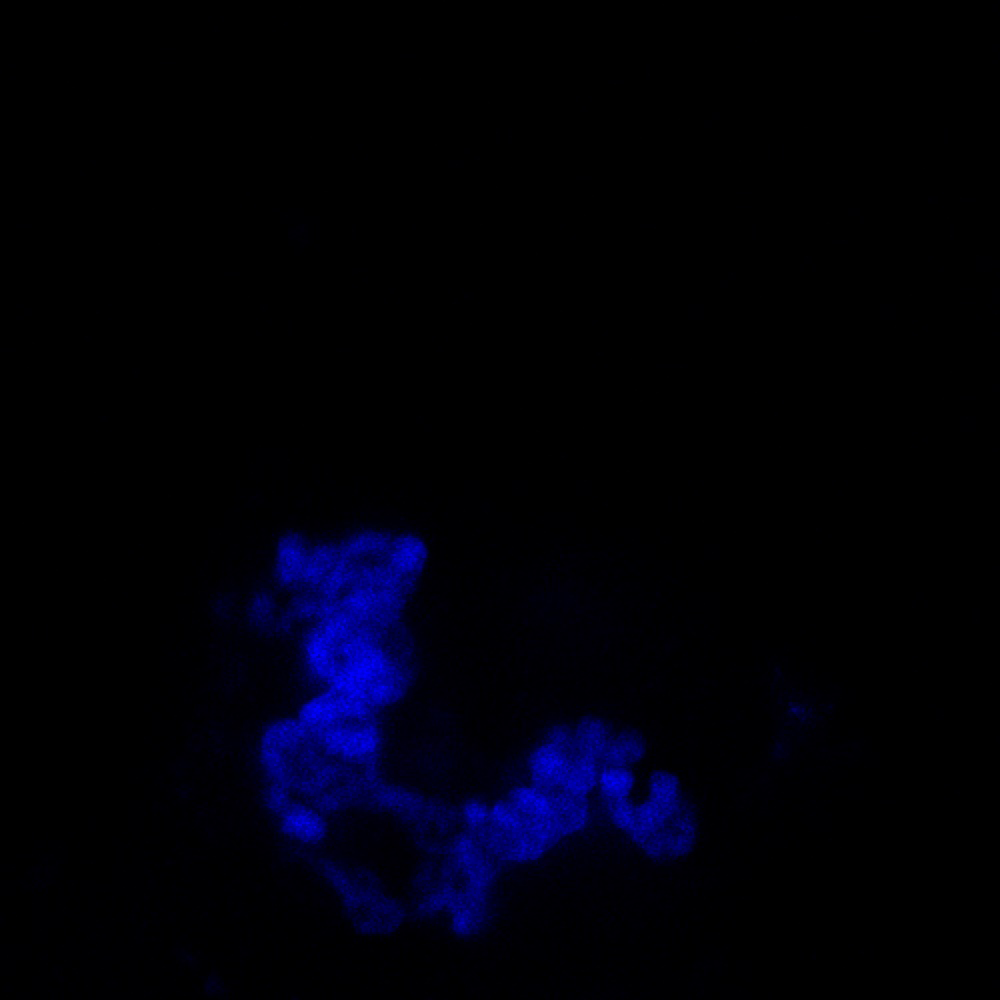

Supplement: Supplementary file 7 — Source data Fig. 5 [file 44319_2026_833_MOESM7_ESM.zip › 5B/shSCP4-H3T3A-shH3 Anaphase-2/Experiment-4576-Airyscan Processing-20-Orthogonal Projection-01/Experiment-4576-Airyscan Processing-20_z08c3.tif]

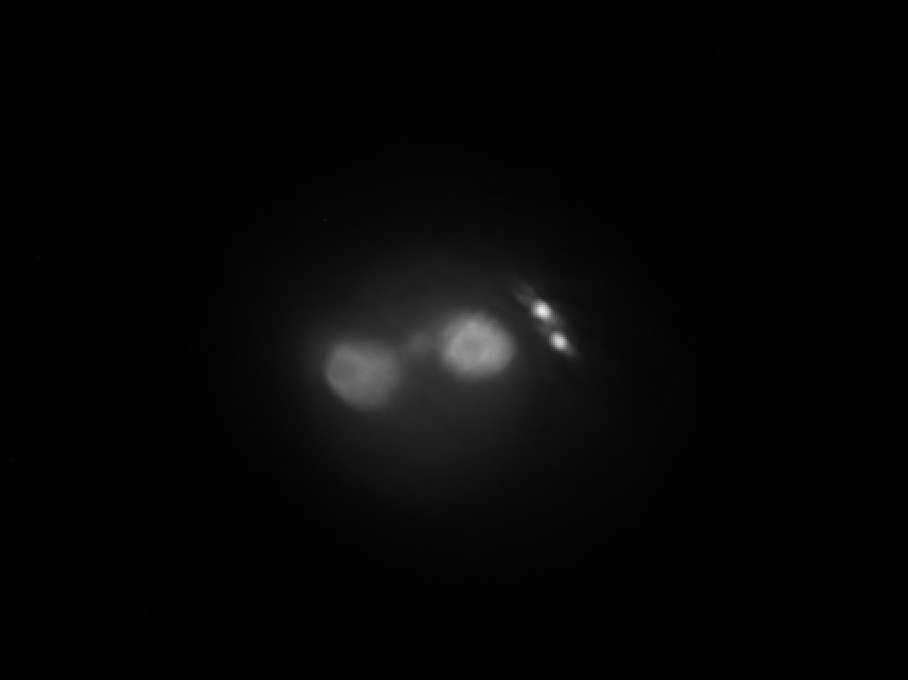

Supplement: Supplementary file 7 — Source data Fig. 5 [file 44319_2026_833_MOESM7_ESM.zip › 5E/KO-DAPI.tif]

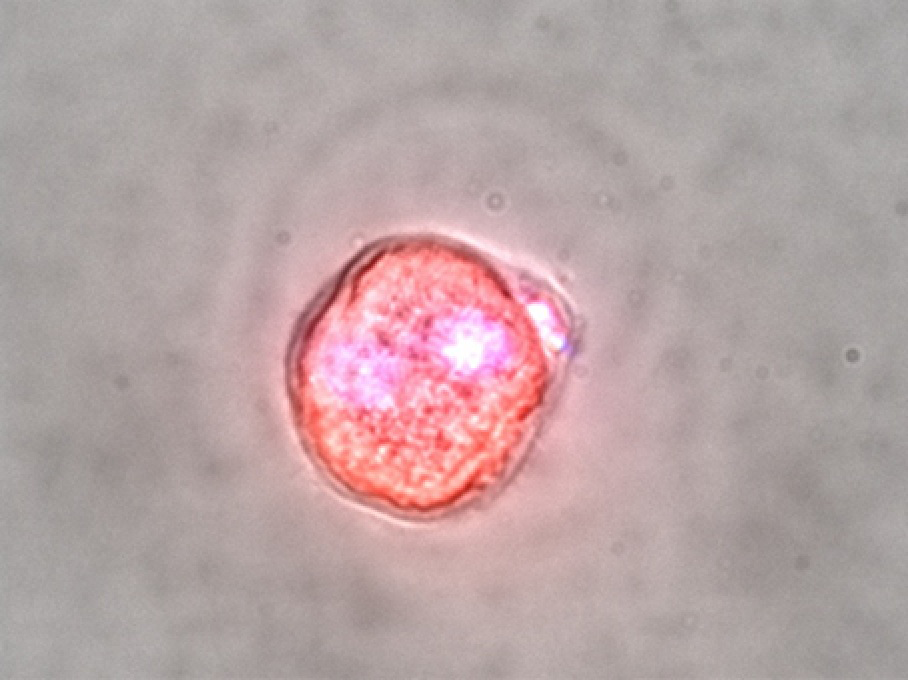

Supplement: Supplementary file 7 — Source data Fig. 5 [file 44319_2026_833_MOESM7_ESM.zip › 5E/KO-Merge.tif]

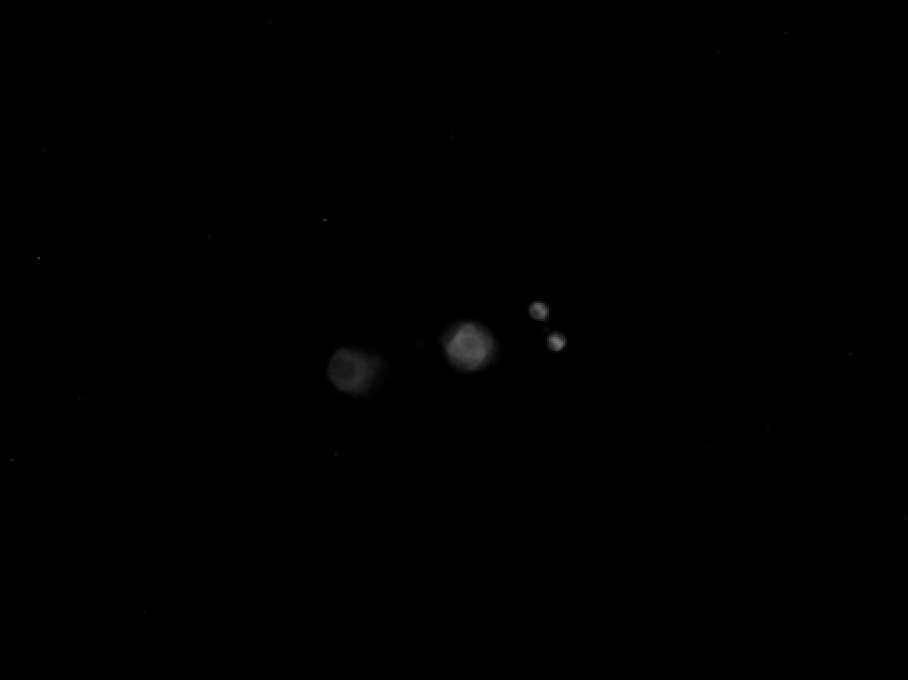

Supplement: Supplementary file 7 — Source data Fig. 5 [file 44319_2026_833_MOESM7_ESM.zip › 5E/KO-pS10.tif]

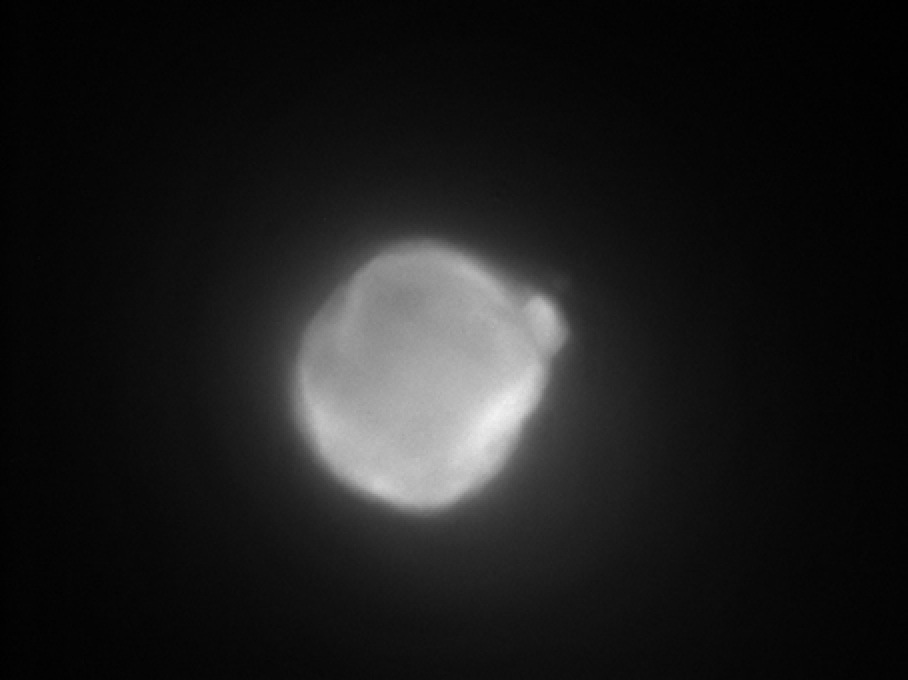

Supplement: Supplementary file 7 — Source data Fig. 5 [file 44319_2026_833_MOESM7_ESM.zip › 5E/KO-tubulin.tif]

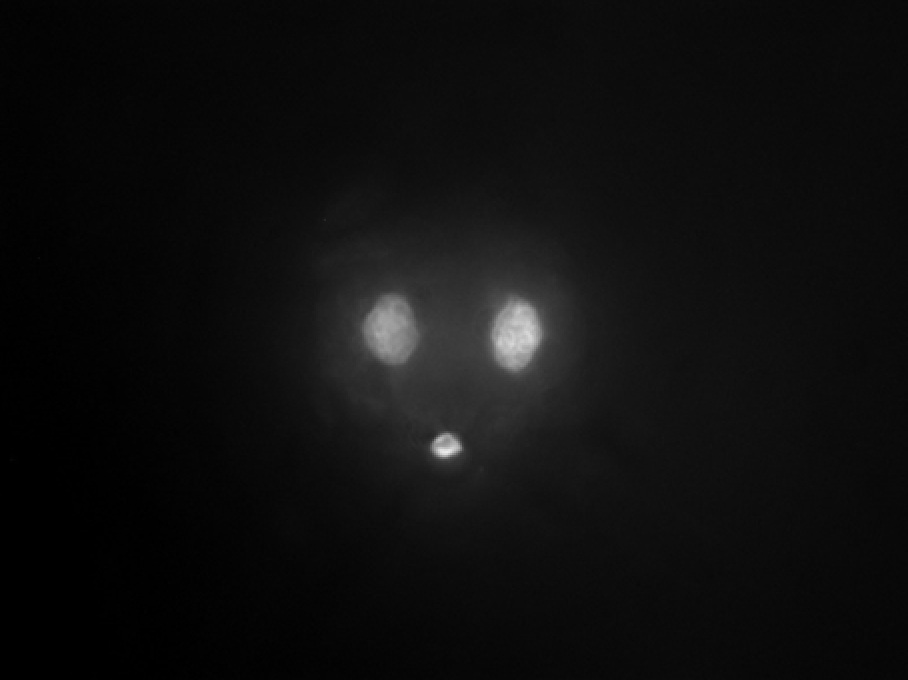

Supplement: Supplementary file 7 — Source data Fig. 5 [file 44319_2026_833_MOESM7_ESM.zip › 5E/WT-DAPI.tif]

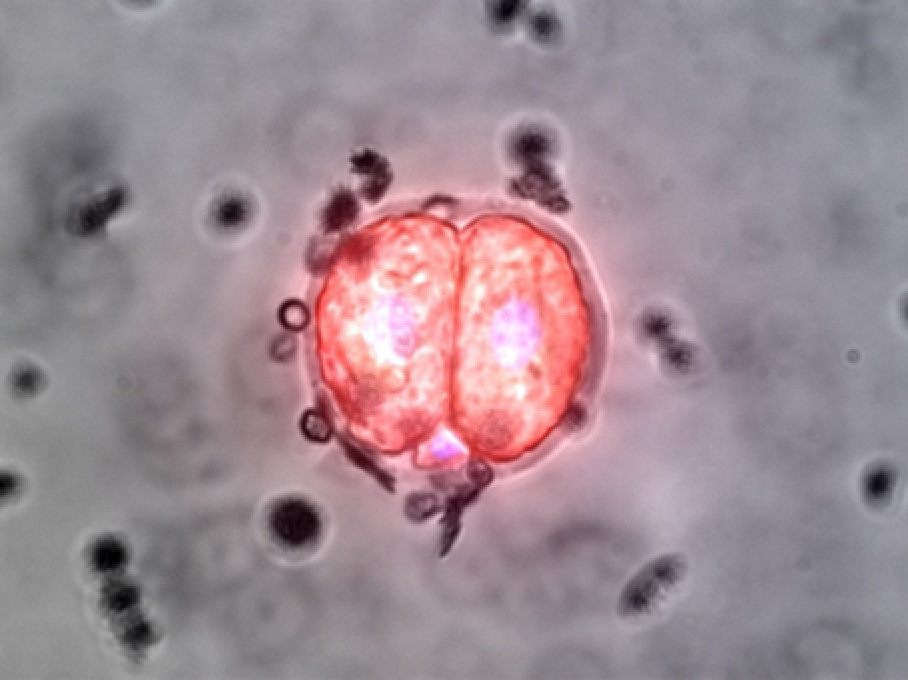

Supplement: Supplementary file 7 — Source data Fig. 5 [file 44319_2026_833_MOESM7_ESM.zip › 5E/WT-Merge.tif]

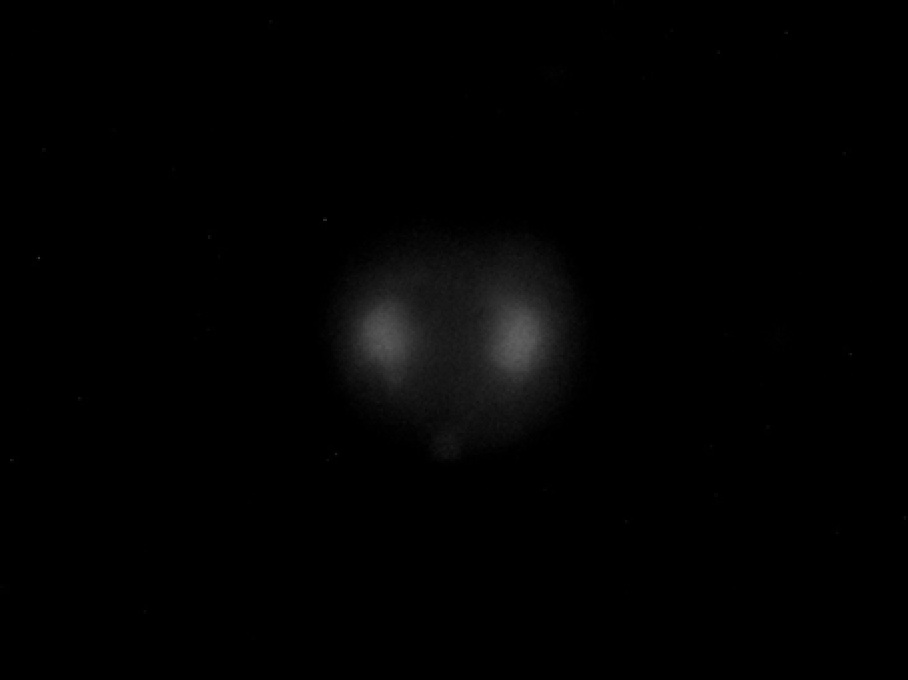

Supplement: Supplementary file 7 — Source data Fig. 5 [file 44319_2026_833_MOESM7_ESM.zip › 5E/WT-pS10.tif]

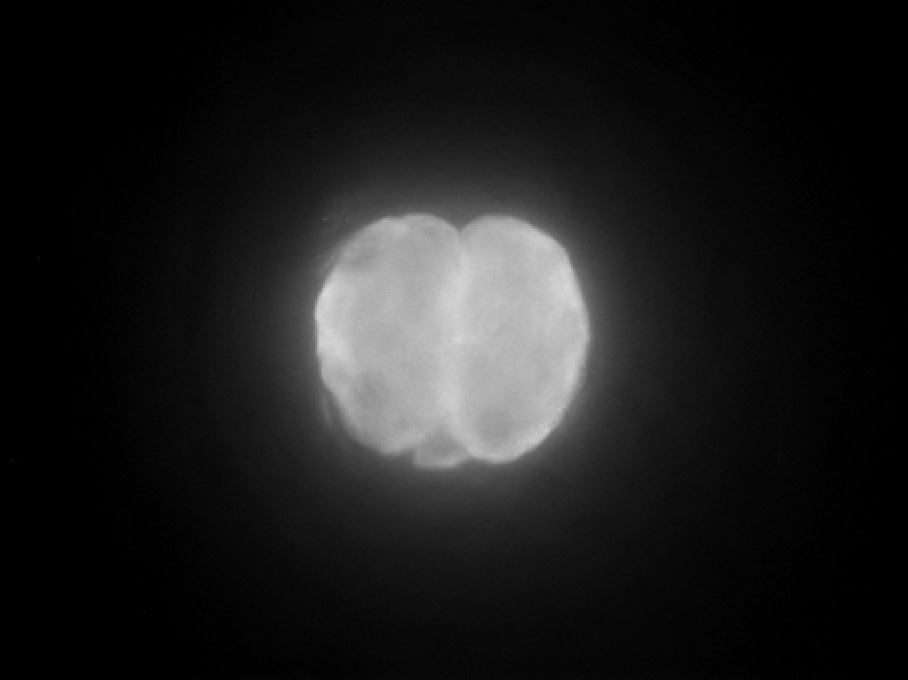

Supplement: Supplementary file 7 — Source data Fig. 5 [file 44319_2026_833_MOESM7_ESM.zip › 5E/WT-tubulin.tif]

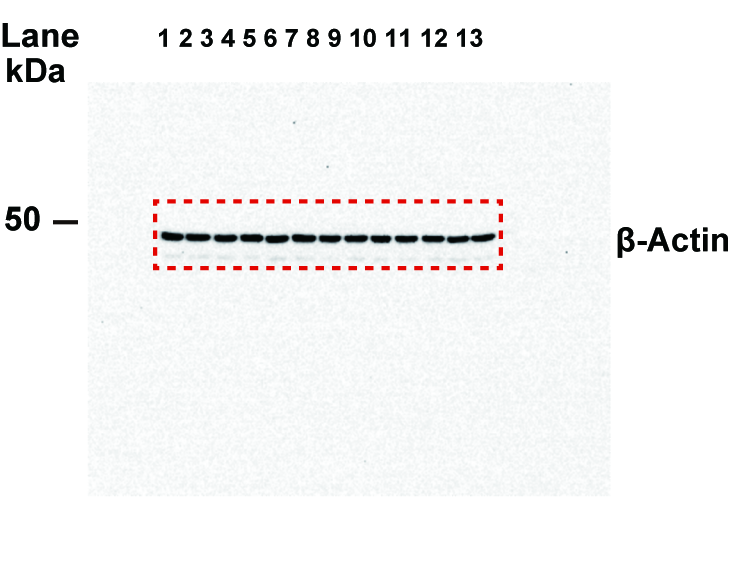

Supplement: Supplementary file 8 — Figure EV1 Source Data [file 44319_2026_833_MOESM8_ESM.zip › EV1A/Actin/Origin EV1A Actin.tif]

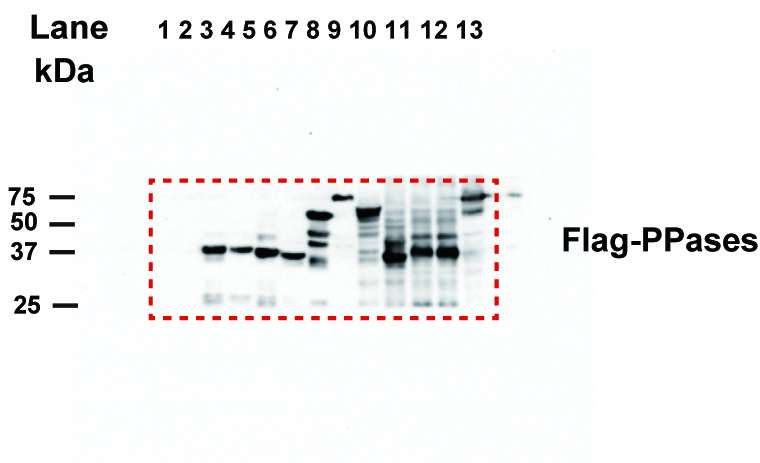

Supplement: Supplementary file 8 — Figure EV1 Source Data [file 44319_2026_833_MOESM8_ESM.zip › EV1A/Flag-PPases/Origin EV1A Flag-PPases.tif]

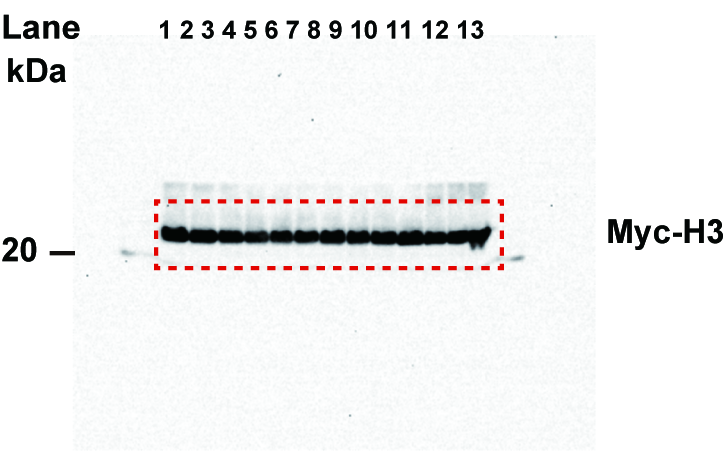

Supplement: Supplementary file 8 — Figure EV1 Source Data [file 44319_2026_833_MOESM8_ESM.zip › EV1A/Myc-H3/Origin EV1A Myc-H3.tif]

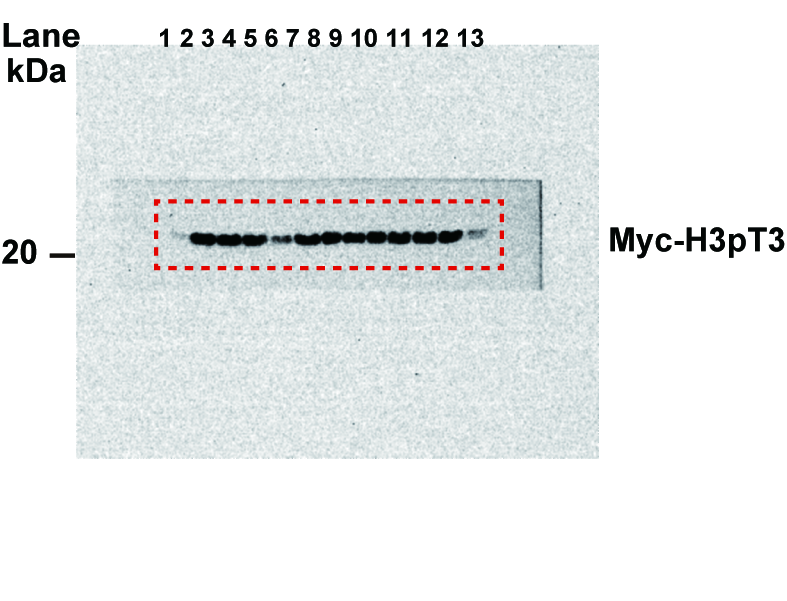

Supplement: Supplementary file 8 — Figure EV1 Source Data [file 44319_2026_833_MOESM8_ESM.zip › EV1A/Myc-H3pT3/Origin EV1A Myc-H3pT3.tif]

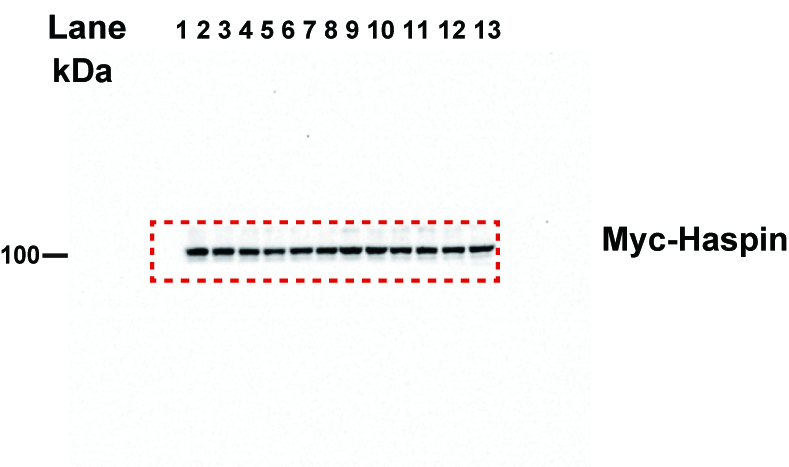

Supplement: Supplementary file 8 — Figure EV1 Source Data [file 44319_2026_833_MOESM8_ESM.zip › EV1A/Myc-Haspin/Origin EV1A Myc-Haspin.tif]

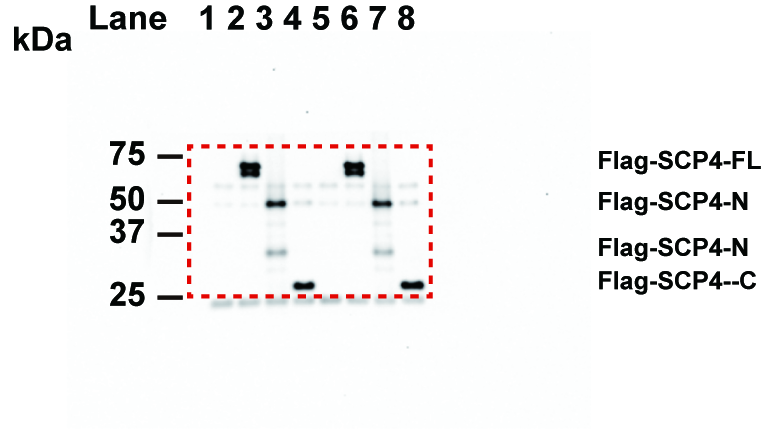

Supplement: Supplementary file 8 — Figure EV1 Source Data [file 44319_2026_833_MOESM8_ESM.zip › EV1C/IP_FLAG IB_FLAG/Origin EV1C IP_FLAG IB_FLAG(1).tif]

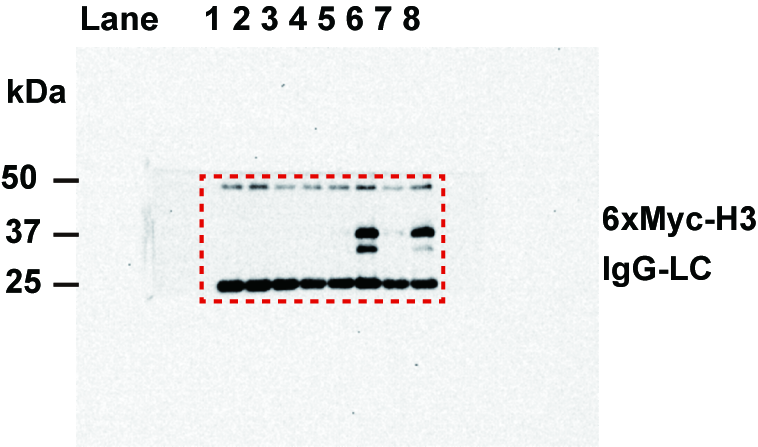

Supplement: Supplementary file 8 — Figure EV1 Source Data [file 44319_2026_833_MOESM8_ESM.zip › EV1C/IP_FLAG IB_Myc/Origin EV1C IP_FLAG IB_Myc.tif]

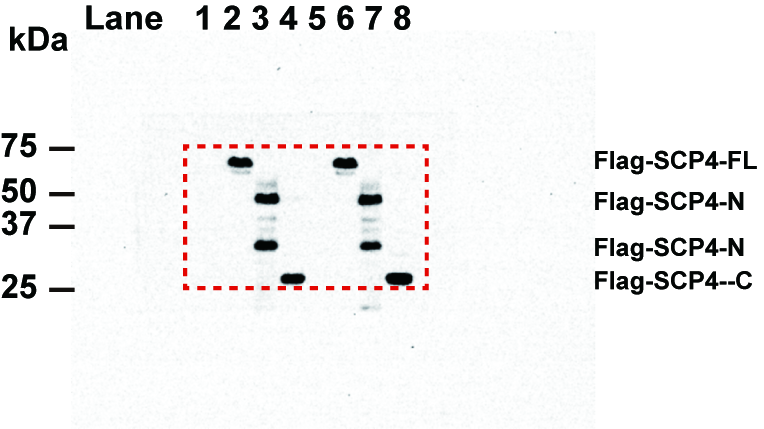

Supplement: Supplementary file 8 — Figure EV1 Source Data [file 44319_2026_833_MOESM8_ESM.zip › EV1C/WCL IB_α-Flag/Origin EV1C WCL IB_FLAG(1).tif]

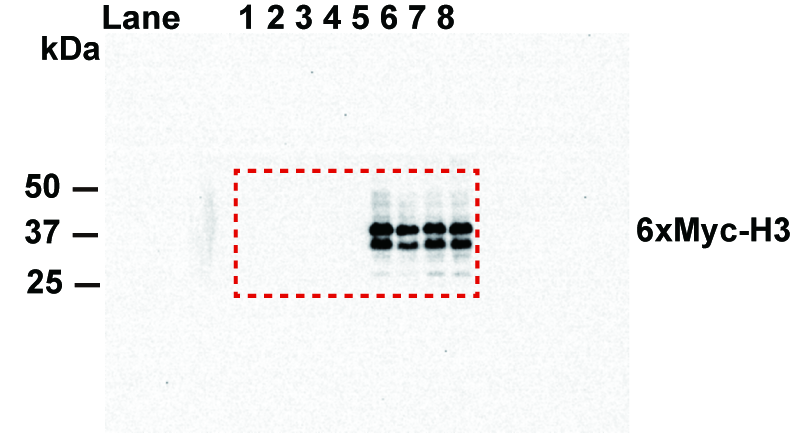

Supplement: Supplementary file 8 — Figure EV1 Source Data [file 44319_2026_833_MOESM8_ESM.zip › EV1C/WCL IB_α-Myc/Origin EV1C WCL IB_Myc.tif]

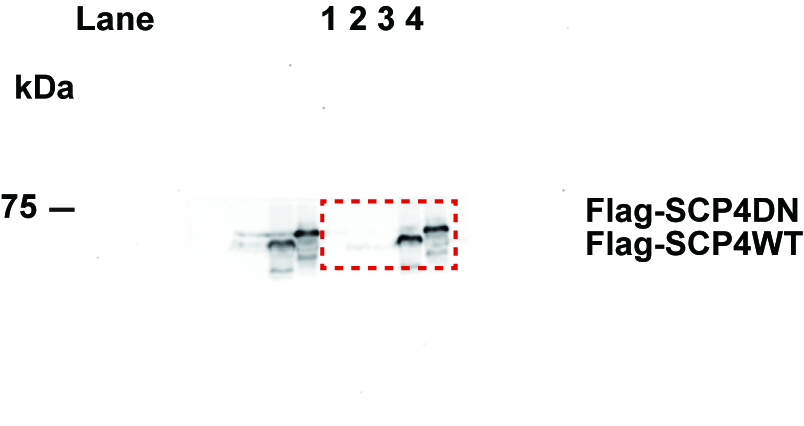

Supplement: Supplementary file 8 — Figure EV1 Source Data [file 44319_2026_833_MOESM8_ESM.zip › EV1D/FLAG SCP4WT DN/Origin EV1D FLAG-SCP4DN_WT.tif]

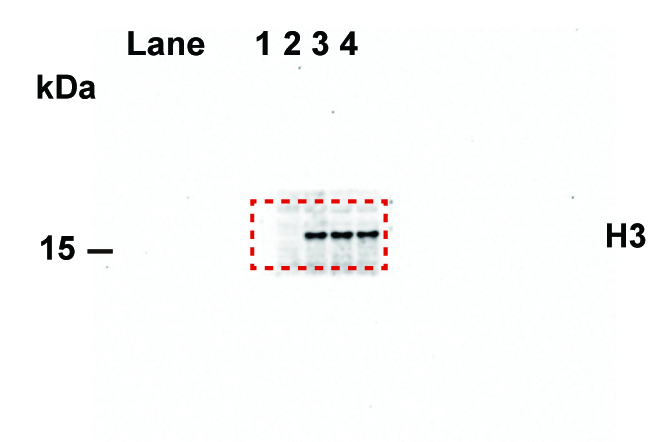

Supplement: Supplementary file 8 — Figure EV1 Source Data [file 44319_2026_833_MOESM8_ESM.zip › EV1D/H3/Origin EV1D H3.tif]

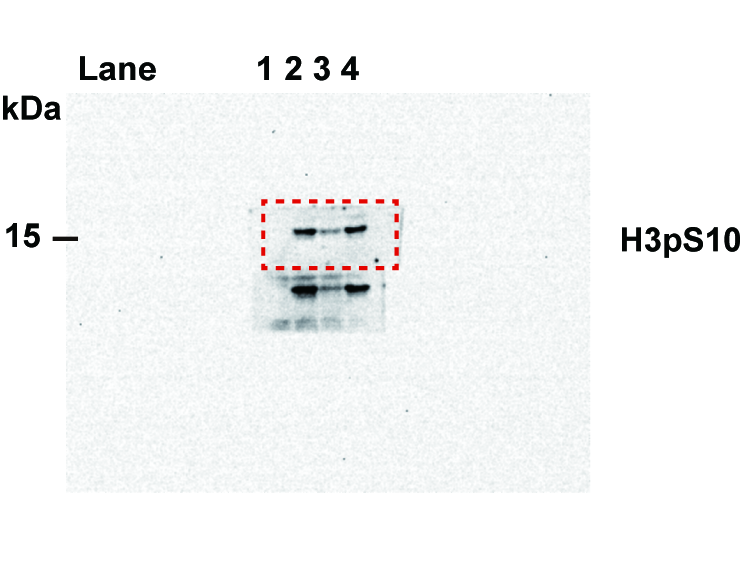

Supplement: Supplementary file 8 — Figure EV1 Source Data [file 44319_2026_833_MOESM8_ESM.zip › EV1D/H3pS10/Origin EV1D H3pS10.tif]

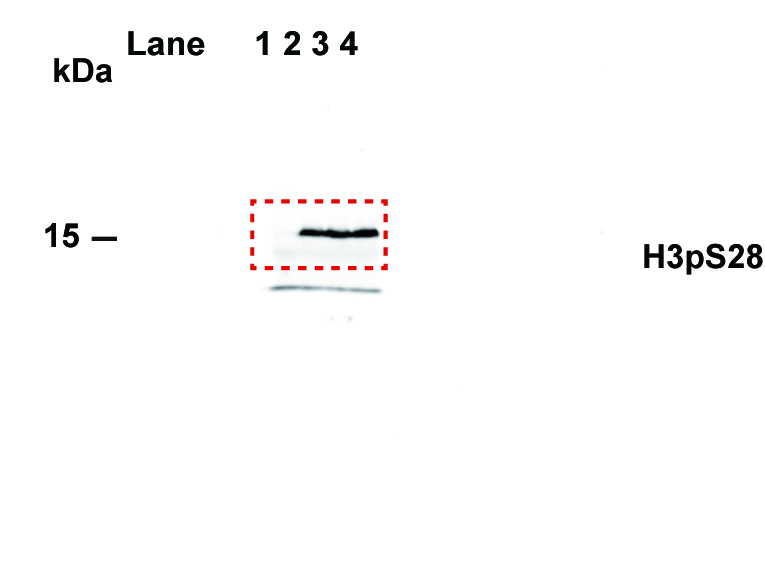

Supplement: Supplementary file 8 — Figure EV1 Source Data [file 44319_2026_833_MOESM8_ESM.zip › EV1D/H3pS28/Origin EV1D H3pS28.tif]
