# Supplementary figures and images for: SCP4 dephosphorylates mitotic histone H3 to maintain chromosome stability (part 2 of 2)
Source: EMBO Rep. 2026 Jun 19;27(14):3944–63. doi: 10.1038/s44319-026-00833-1 (PMC13400628; doi:10.1038/s44319-026-00833-1)

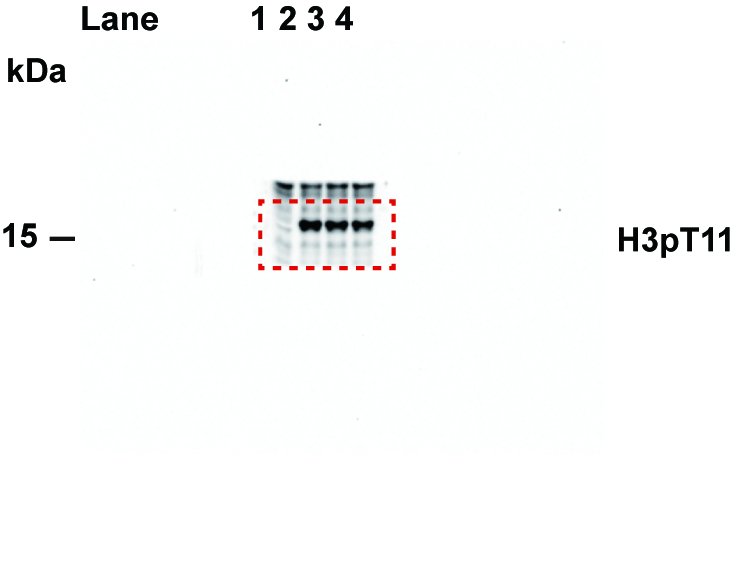

Supplement: Supplementary file 8 — Figure EV1 Source Data [file 44319_2026_833_MOESM8_ESM.zip › EV1D/H3pT11/Origin EV1D H3pT11.tif]

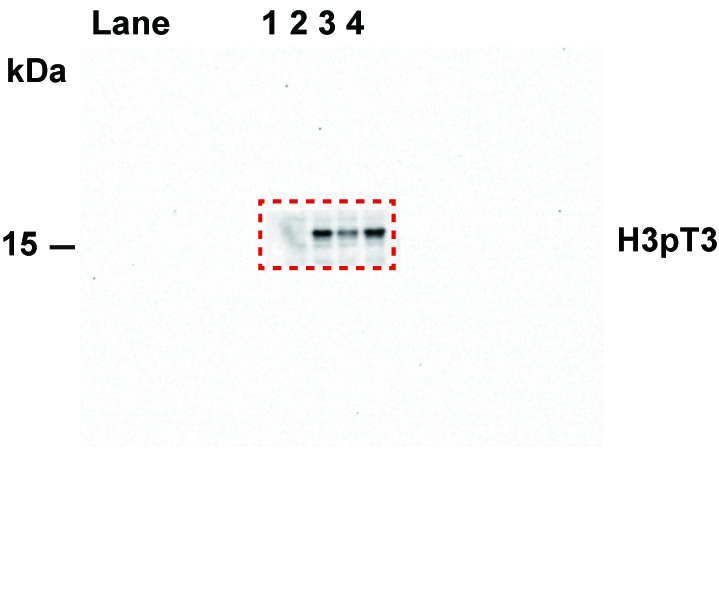

Supplement: Supplementary file 8 — Figure EV1 Source Data [file 44319_2026_833_MOESM8_ESM.zip › EV1D/H3pT3/Origin EV1D H3pT3.tif]

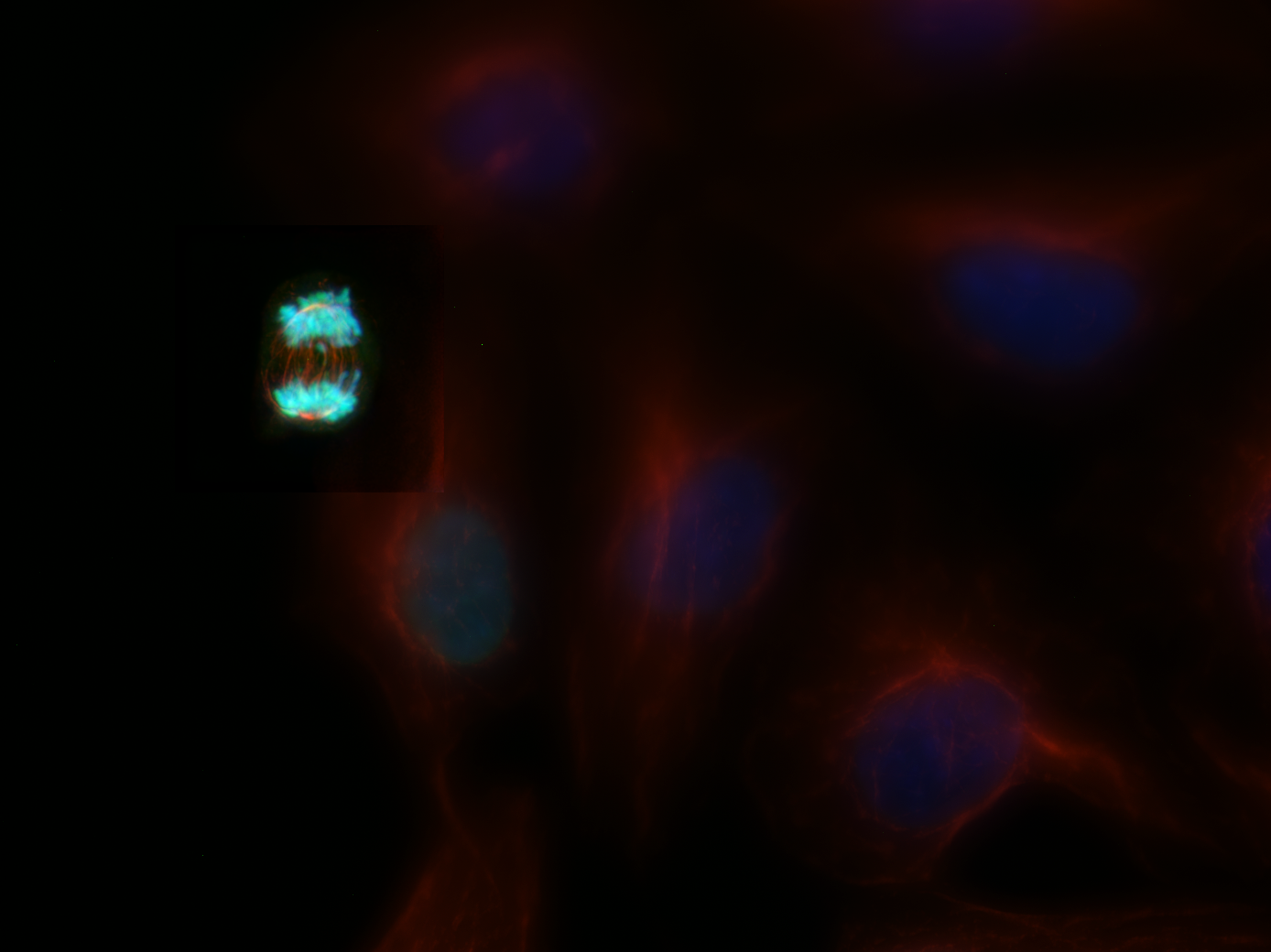

Supplement: Supplementary file 9 — Figure EV2 Source Data [file 44319_2026_833_MOESM9_ESM.zip › EV2A/EMBO_GFP-SCP4_Anaphase_06_dec-Orthogonal Projection-07/EMBO_GFP-SCP4_Anaphase_06_dec-Orthogonal Projection-07_c1-3.tif]

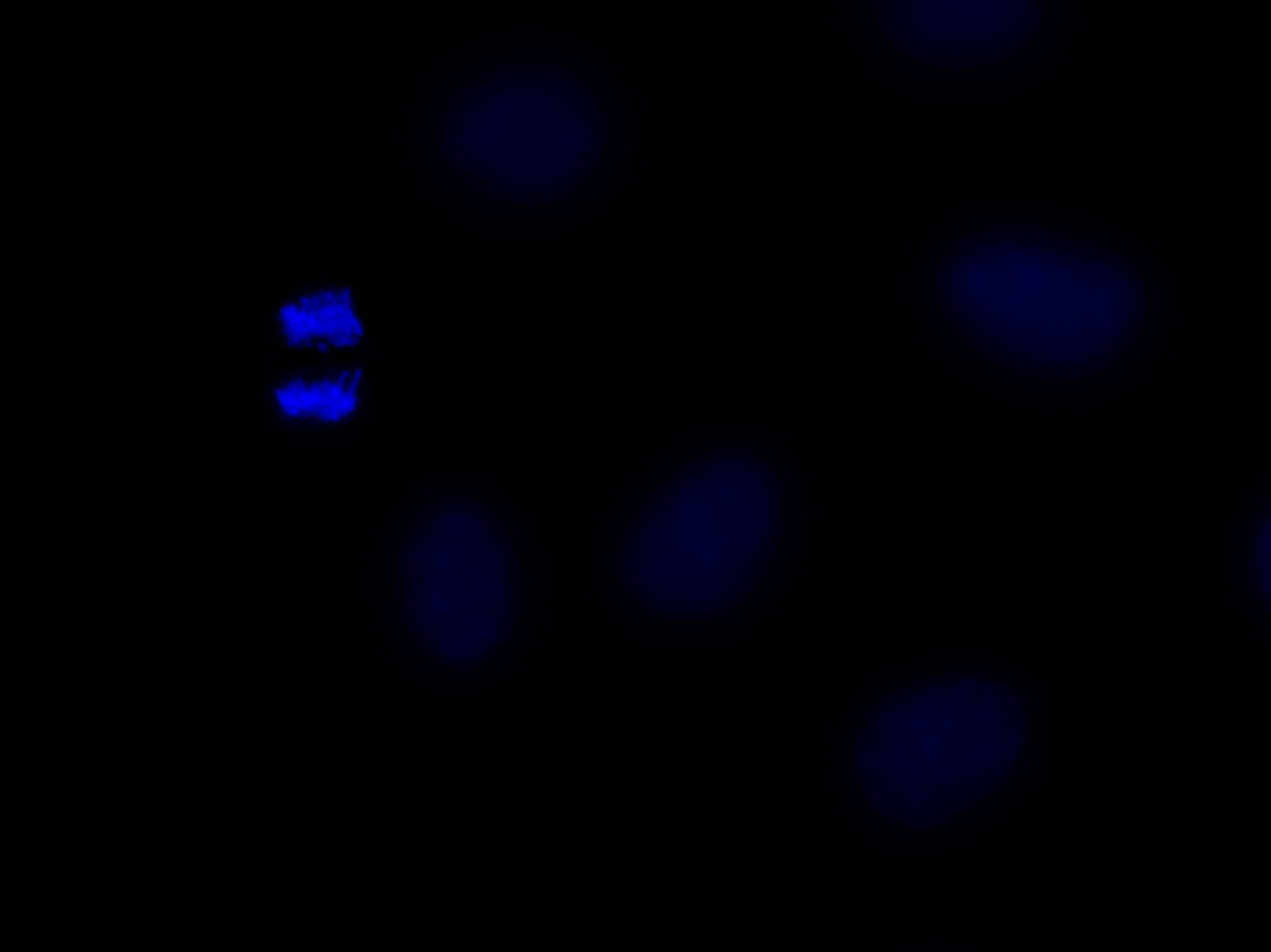

Supplement: Supplementary file 9 — Figure EV2 Source Data [file 44319_2026_833_MOESM9_ESM.zip › EV2A/EMBO_GFP-SCP4_Anaphase_06_dec-Orthogonal Projection-07/EMBO_GFP-SCP4_Anaphase_06_dec-Orthogonal Projection-07_c1.tif]

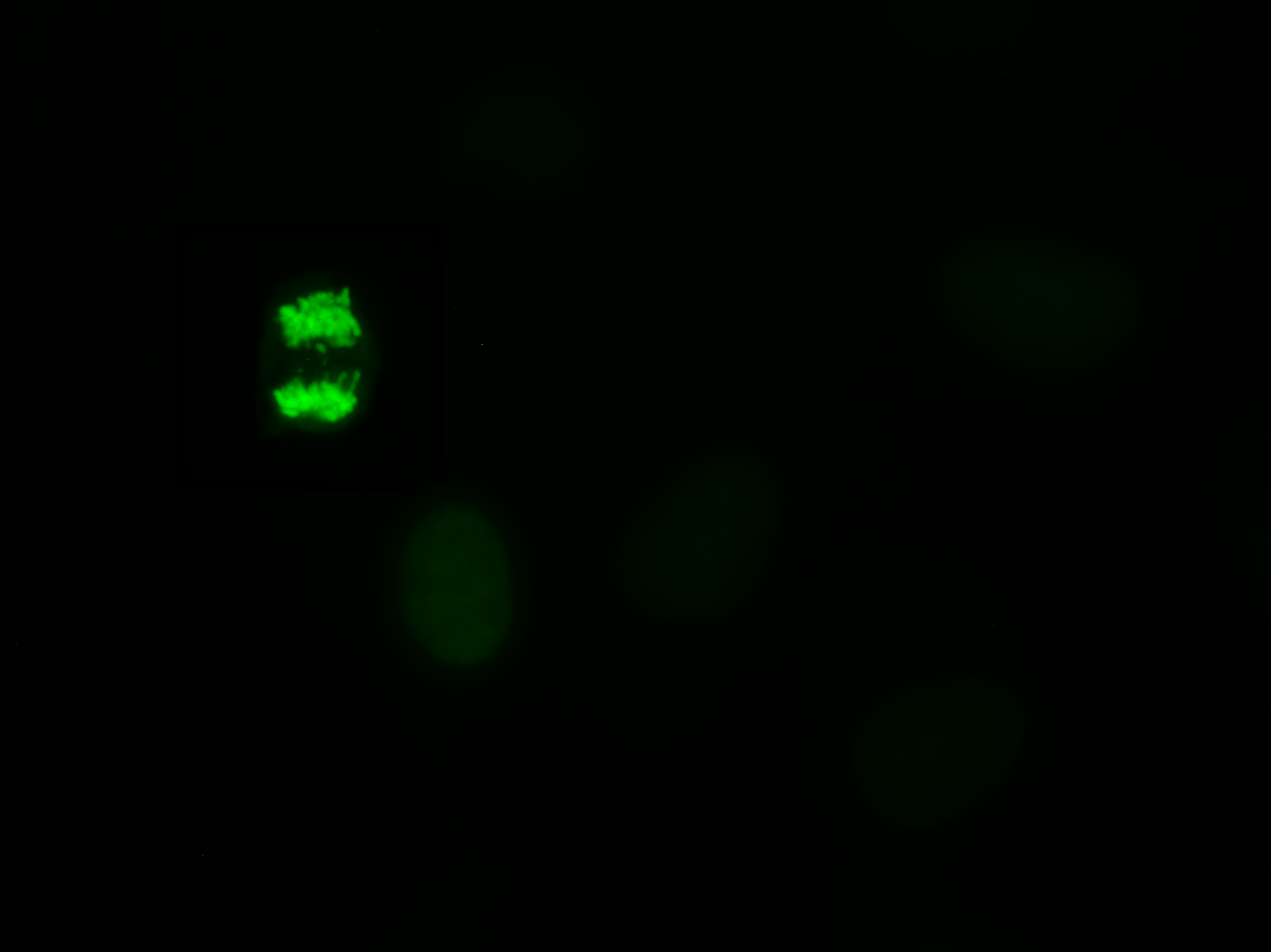

Supplement: Supplementary file 9 — Figure EV2 Source Data [file 44319_2026_833_MOESM9_ESM.zip › EV2A/EMBO_GFP-SCP4_Anaphase_06_dec-Orthogonal Projection-07/EMBO_GFP-SCP4_Anaphase_06_dec-Orthogonal Projection-07_c2.tif]

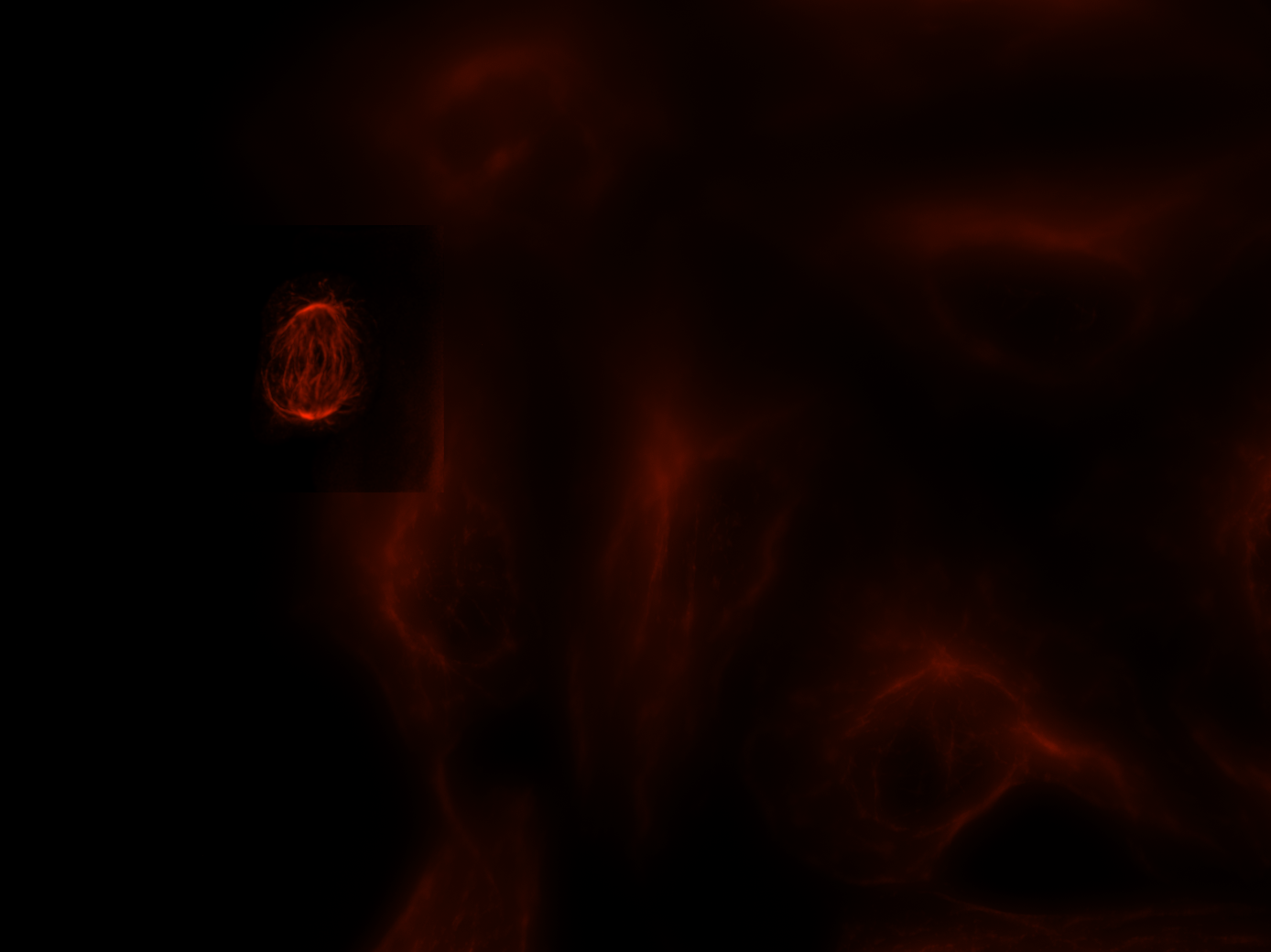

Supplement: Supplementary file 9 — Figure EV2 Source Data [file 44319_2026_833_MOESM9_ESM.zip › EV2A/EMBO_GFP-SCP4_Anaphase_06_dec-Orthogonal Projection-07/EMBO_GFP-SCP4_Anaphase_06_dec-Orthogonal Projection-07_c3.tif]

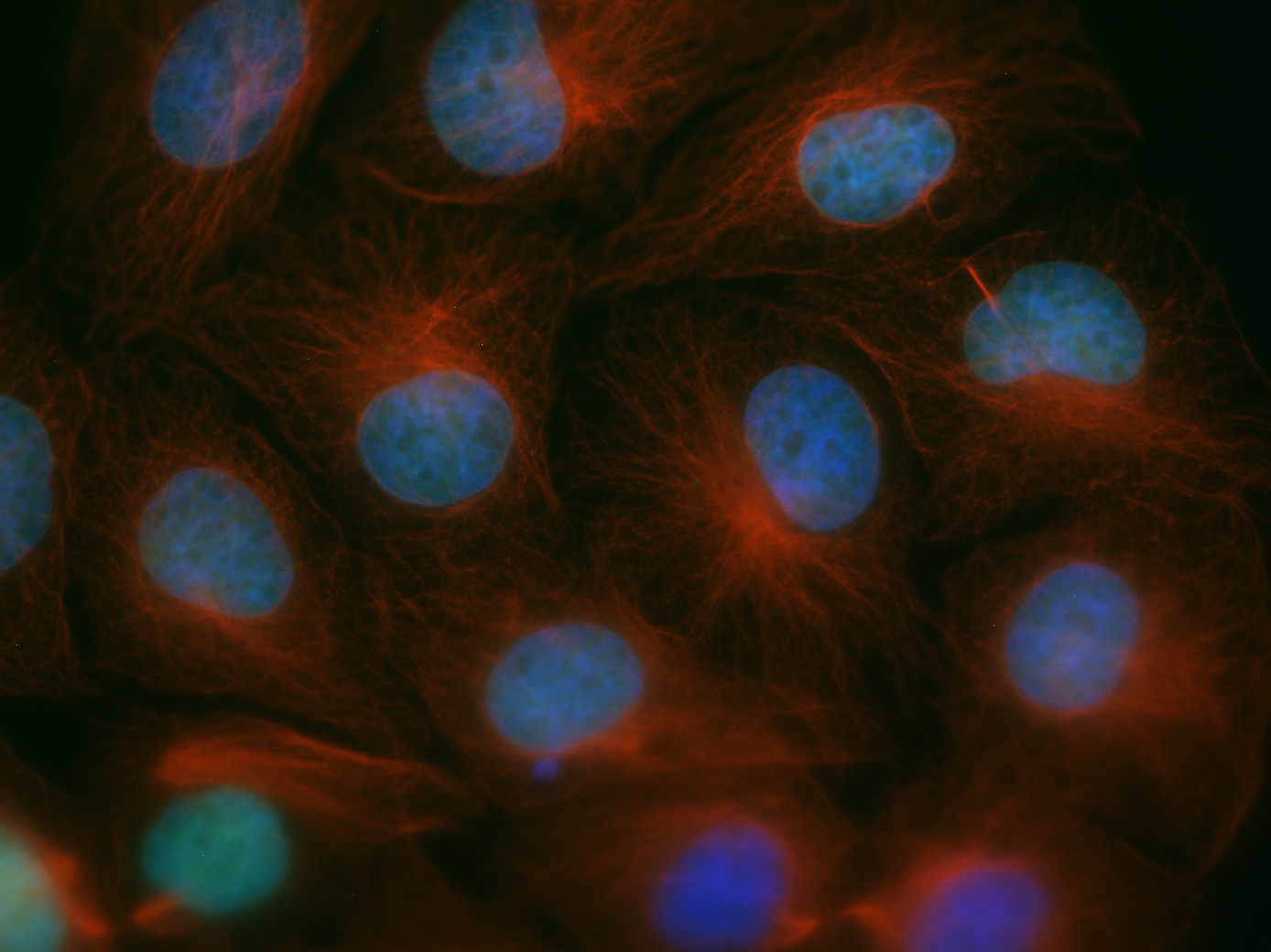

Supplement: Supplementary file 9 — Figure EV2 Source Data [file 44319_2026_833_MOESM9_ESM.zip › EV2A/EMBO_GFP-SCP4_Interphase_08-Orthogonal Projection-09/EMBO_GFP-SCP4_Interphase_08-Orthogonal Projection-09_c1-3.tif]

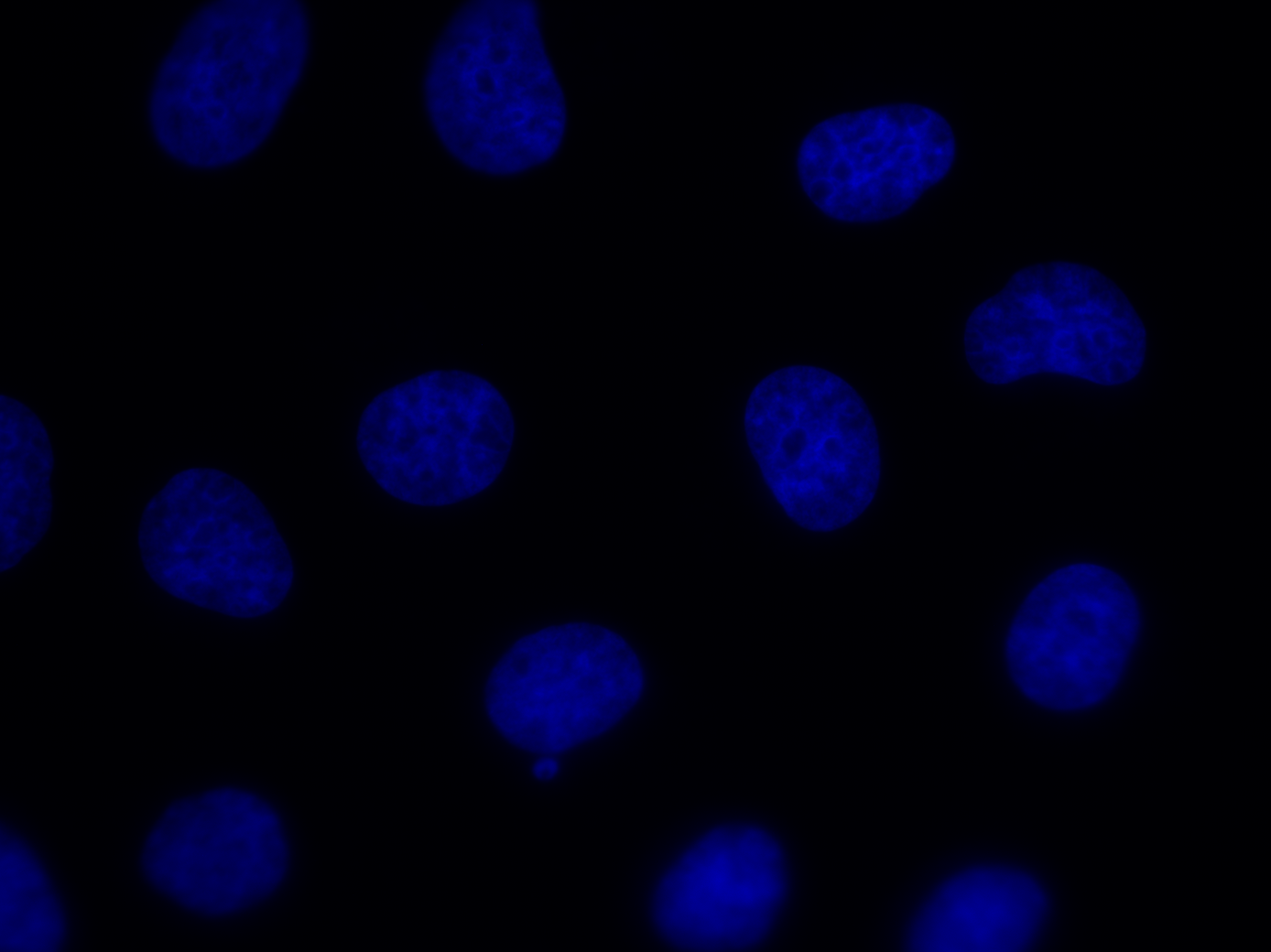

Supplement: Supplementary file 9 — Figure EV2 Source Data [file 44319_2026_833_MOESM9_ESM.zip › EV2A/EMBO_GFP-SCP4_Interphase_08-Orthogonal Projection-09/EMBO_GFP-SCP4_Interphase_08-Orthogonal Projection-09_c1.tif]

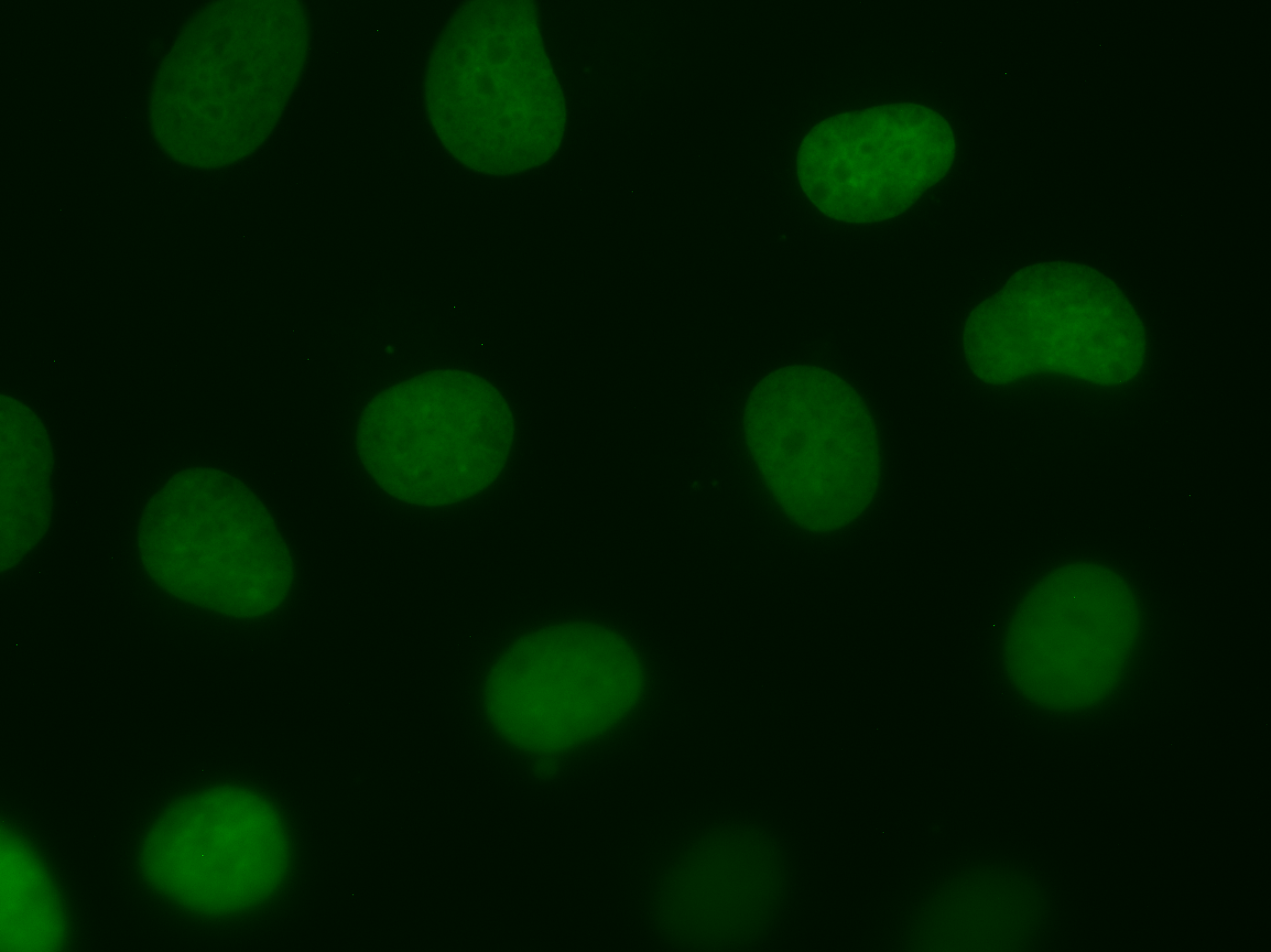

Supplement: Supplementary file 9 — Figure EV2 Source Data [file 44319_2026_833_MOESM9_ESM.zip › EV2A/EMBO_GFP-SCP4_Interphase_08-Orthogonal Projection-09/EMBO_GFP-SCP4_Interphase_08-Orthogonal Projection-09_c2.tif]

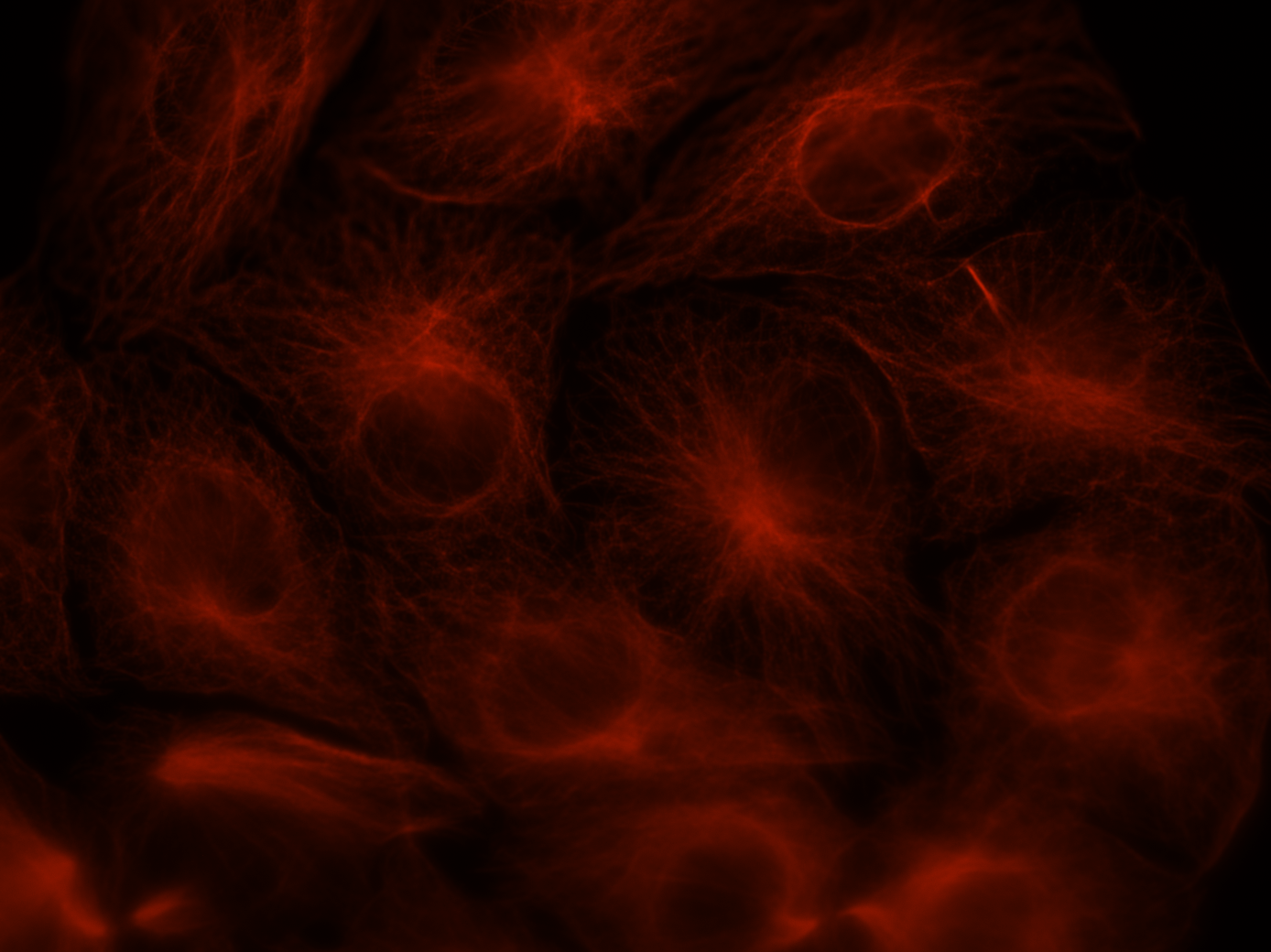

Supplement: Supplementary file 9 — Figure EV2 Source Data [file 44319_2026_833_MOESM9_ESM.zip › EV2A/EMBO_GFP-SCP4_Interphase_08-Orthogonal Projection-09/EMBO_GFP-SCP4_Interphase_08-Orthogonal Projection-09_c3.tif]

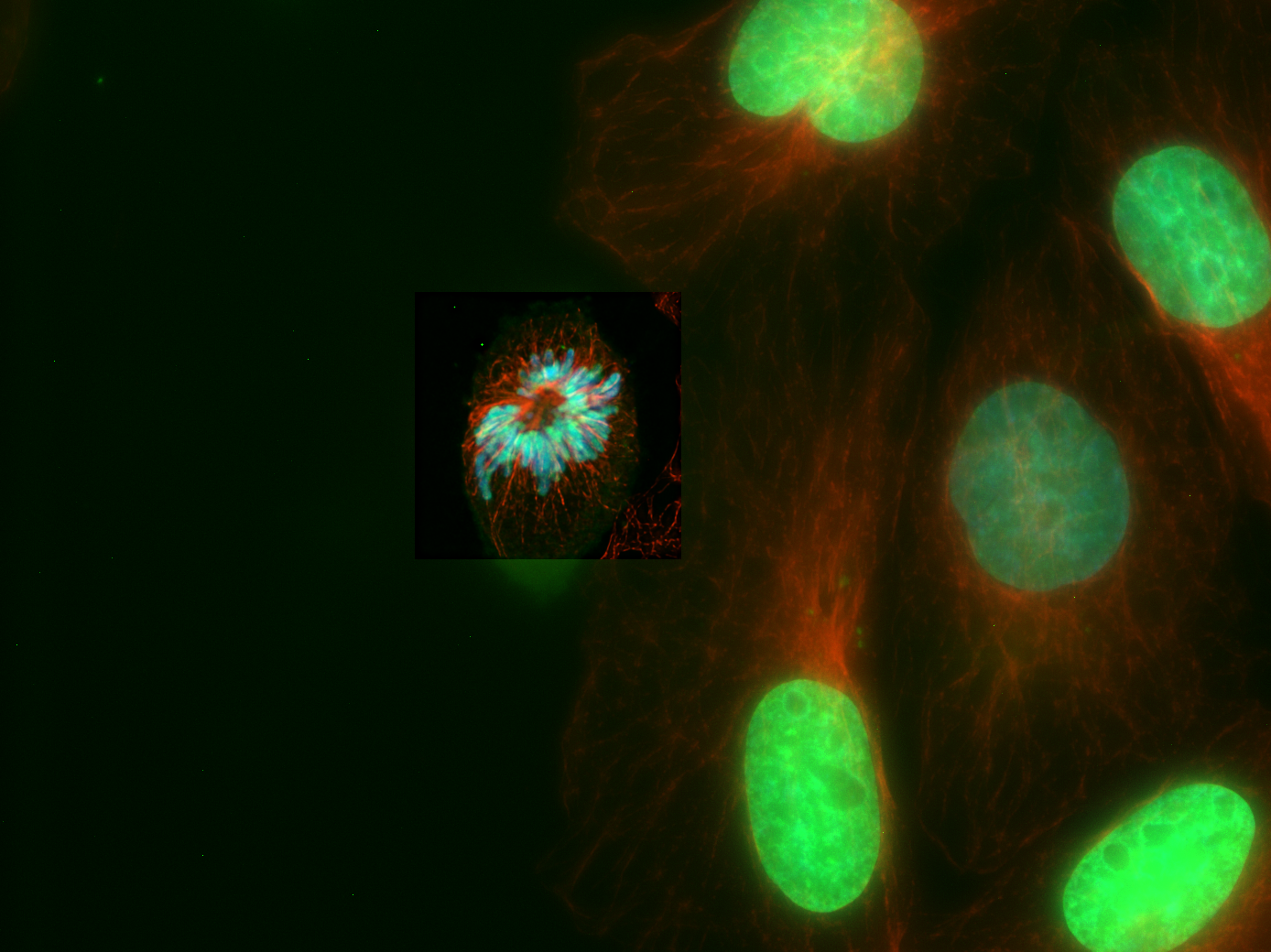

Supplement: Supplementary file 9 — Figure EV2 Source Data [file 44319_2026_833_MOESM9_ESM.zip › EV2A/EMBO_GFP-SCP4_Prometaphase_11_dec-Orthogonal Projection-10/EMBO_GFP-SCP4_Prometaphase_11_dec-Orthogonal Projection-10_c1-3.tif]

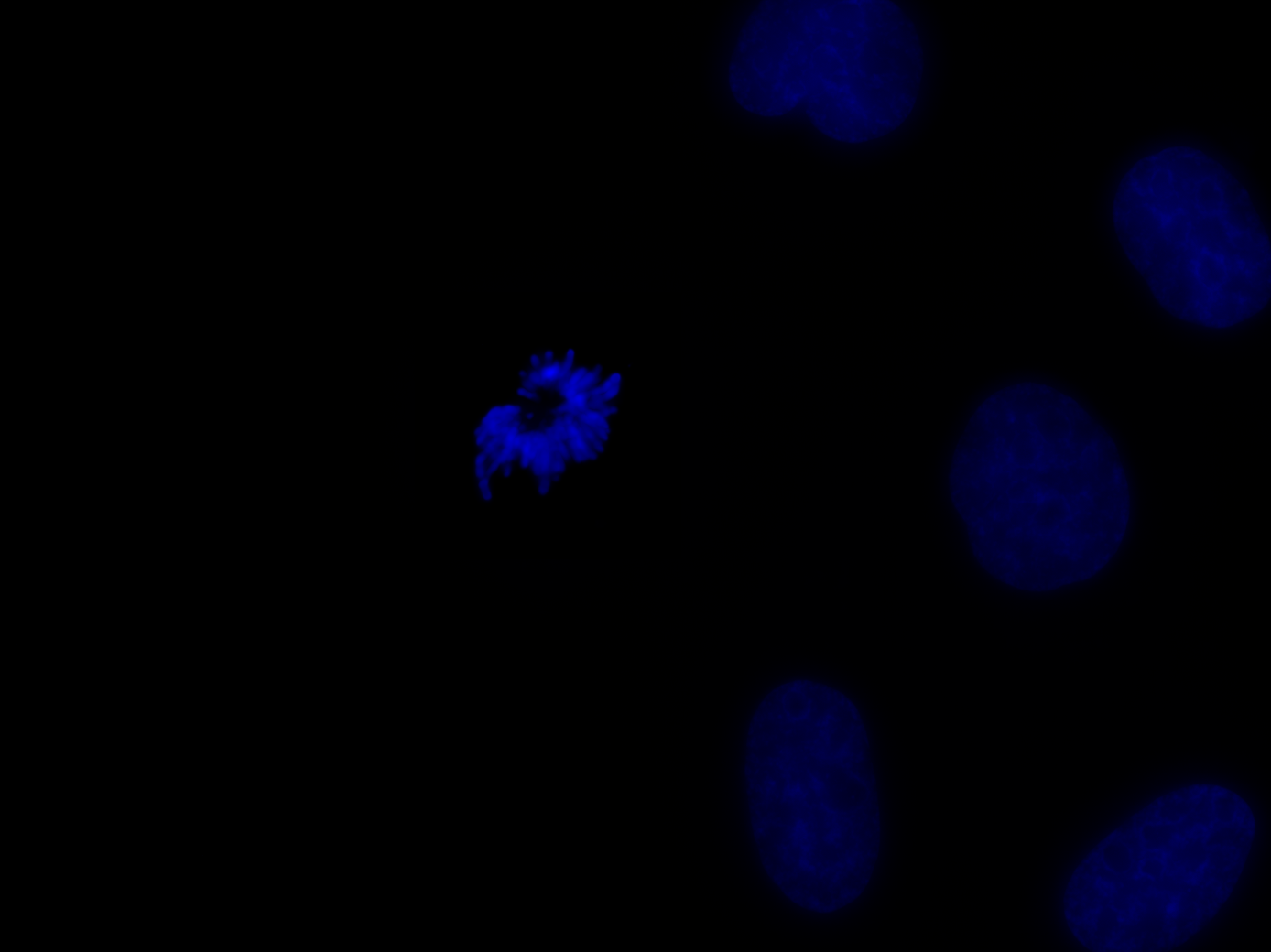

Supplement: Supplementary file 9 — Figure EV2 Source Data [file 44319_2026_833_MOESM9_ESM.zip › EV2A/EMBO_GFP-SCP4_Prometaphase_11_dec-Orthogonal Projection-10/EMBO_GFP-SCP4_Prometaphase_11_dec-Orthogonal Projection-10_c1.tif]

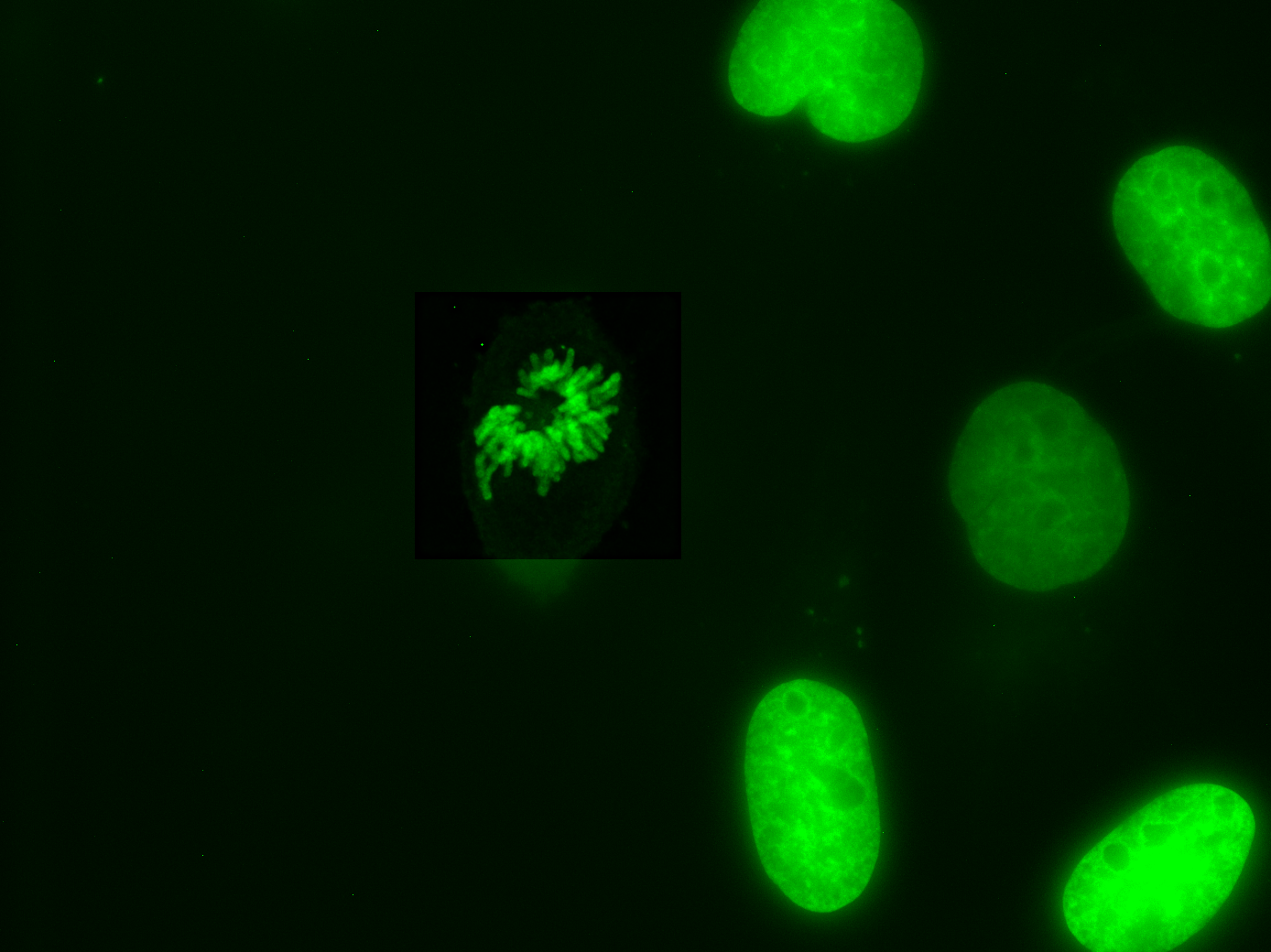

Supplement: Supplementary file 9 — Figure EV2 Source Data [file 44319_2026_833_MOESM9_ESM.zip › EV2A/EMBO_GFP-SCP4_Prometaphase_11_dec-Orthogonal Projection-10/EMBO_GFP-SCP4_Prometaphase_11_dec-Orthogonal Projection-10_c2.tif]

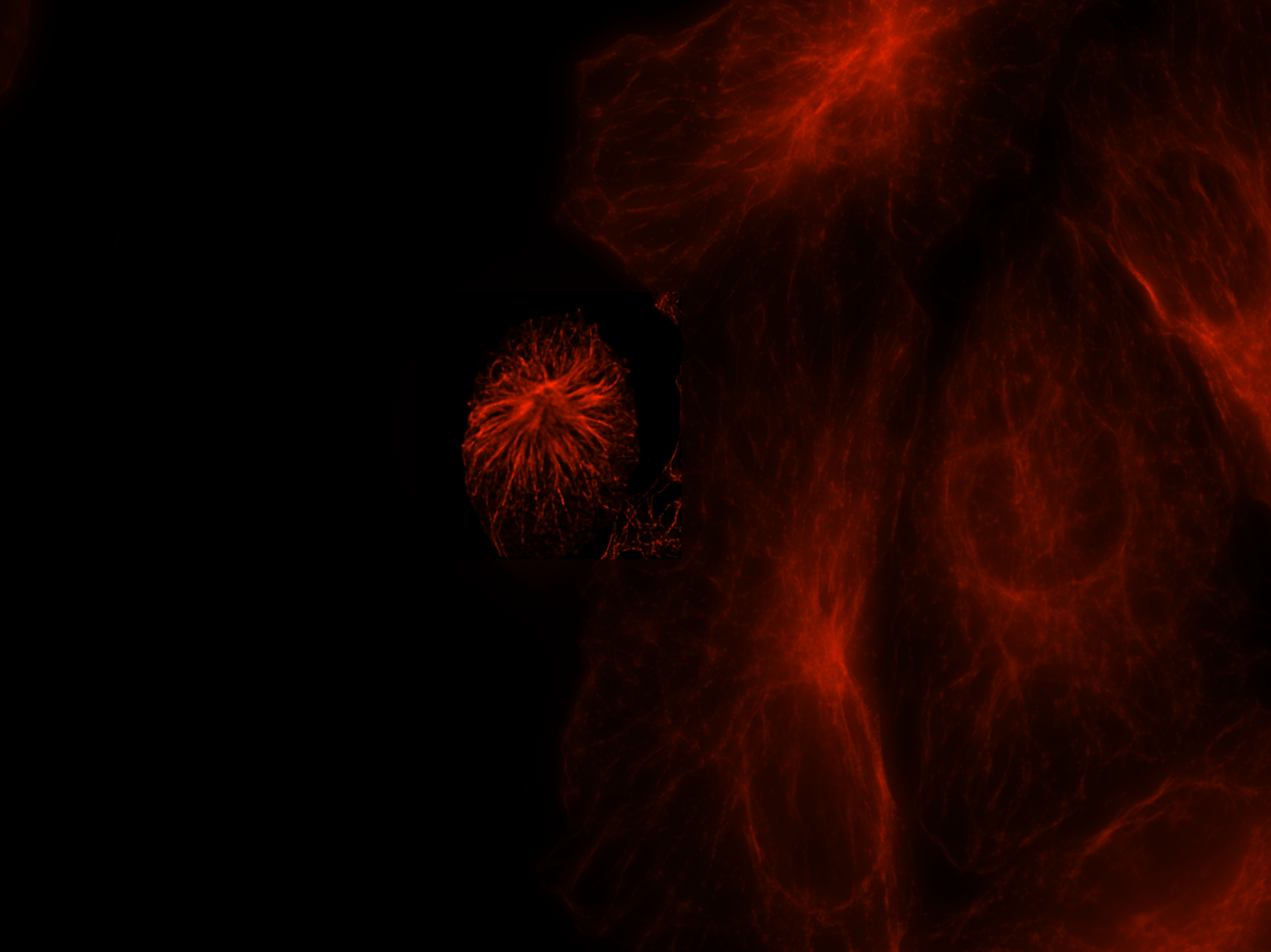

Supplement: Supplementary file 9 — Figure EV2 Source Data [file 44319_2026_833_MOESM9_ESM.zip › EV2A/EMBO_GFP-SCP4_Prometaphase_11_dec-Orthogonal Projection-10/EMBO_GFP-SCP4_Prometaphase_11_dec-Orthogonal Projection-10_c3.tif]

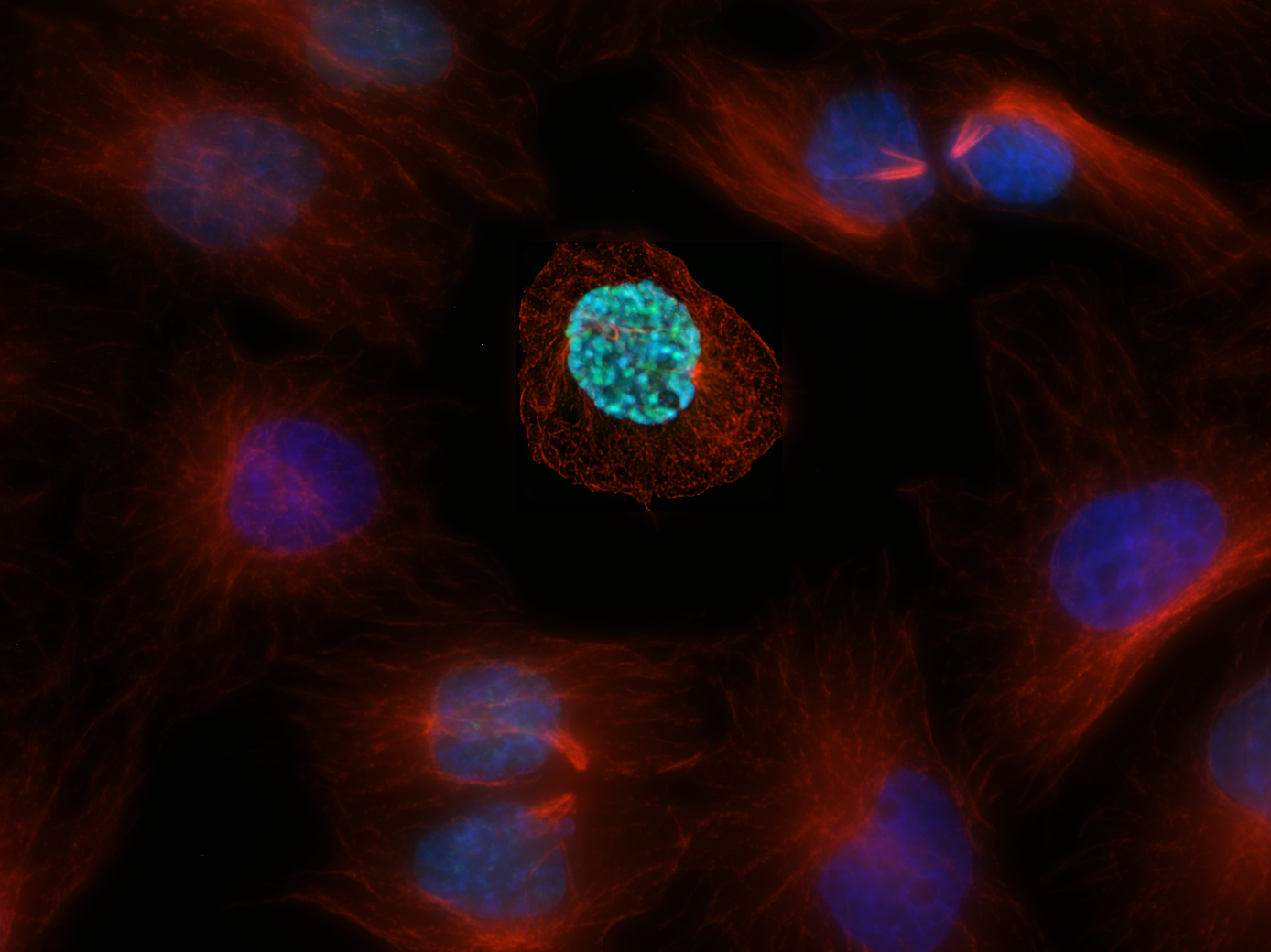

Supplement: Supplementary file 9 — Figure EV2 Source Data [file 44319_2026_833_MOESM9_ESM.zip › EV2A/EMBO_GFP-SCP4_Prophase_03_dec-Orthogonal Projection-11/EMBO_GFP-SCP4_Prophase_03_dec-Orthogonal Projection-11_c1-3.tif]

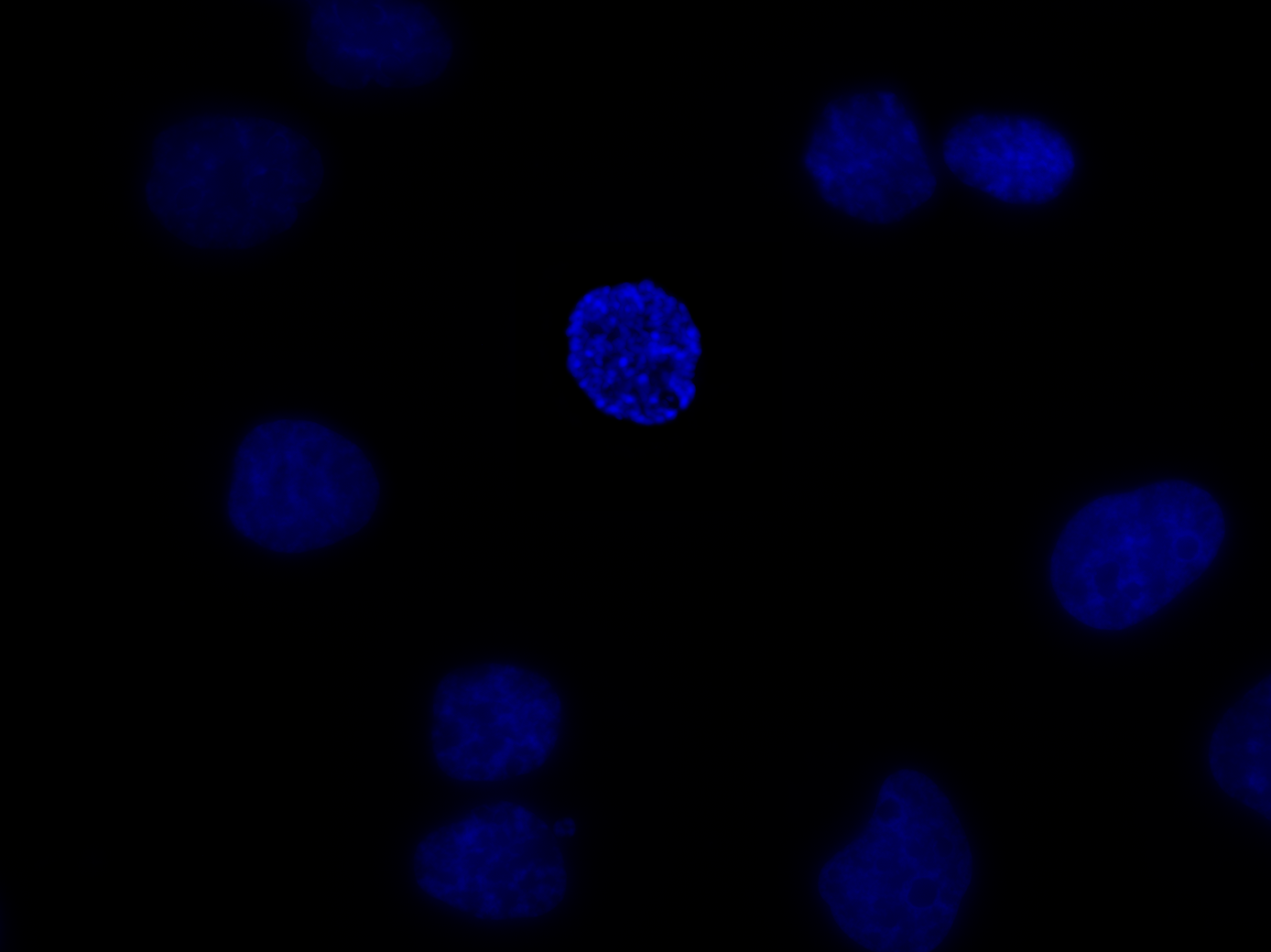

Supplement: Supplementary file 9 — Figure EV2 Source Data [file 44319_2026_833_MOESM9_ESM.zip › EV2A/EMBO_GFP-SCP4_Prophase_03_dec-Orthogonal Projection-11/EMBO_GFP-SCP4_Prophase_03_dec-Orthogonal Projection-11_c1.tif]

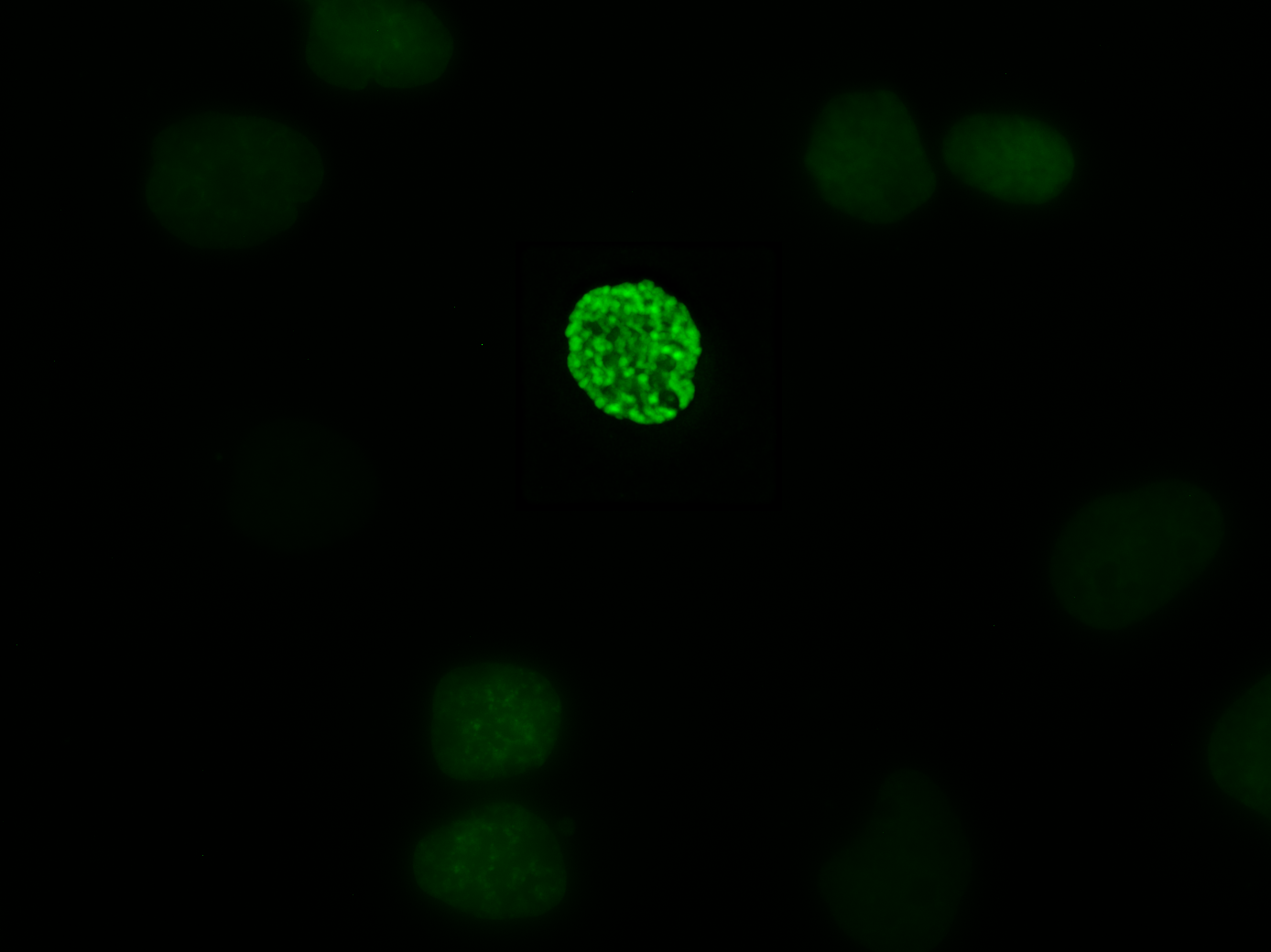

Supplement: Supplementary file 9 — Figure EV2 Source Data [file 44319_2026_833_MOESM9_ESM.zip › EV2A/EMBO_GFP-SCP4_Prophase_03_dec-Orthogonal Projection-11/EMBO_GFP-SCP4_Prophase_03_dec-Orthogonal Projection-11_c2.tif]

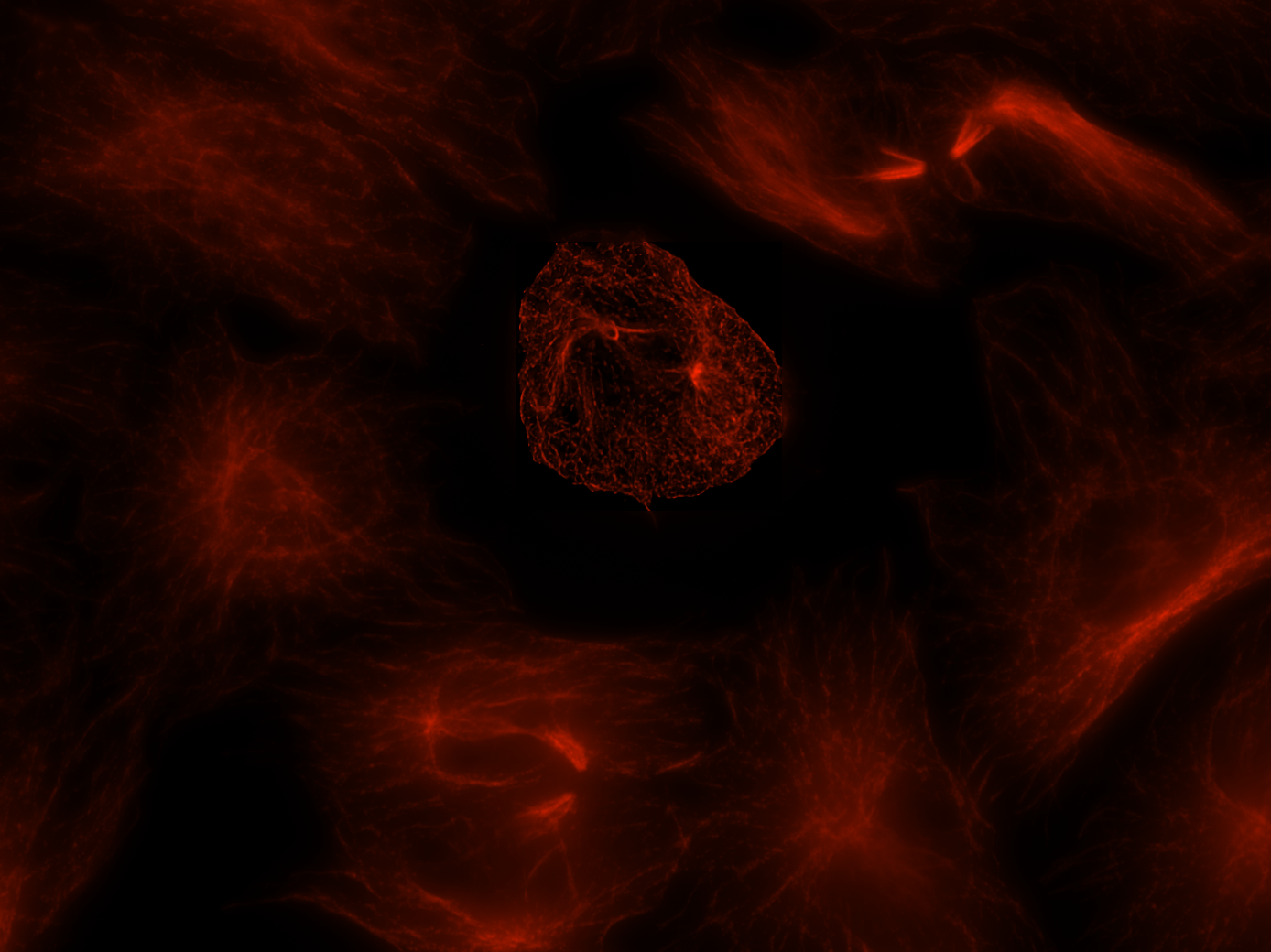

Supplement: Supplementary file 9 — Figure EV2 Source Data [file 44319_2026_833_MOESM9_ESM.zip › EV2A/EMBO_GFP-SCP4_Prophase_03_dec-Orthogonal Projection-11/EMBO_GFP-SCP4_Prophase_03_dec-Orthogonal Projection-11_c3.tif]

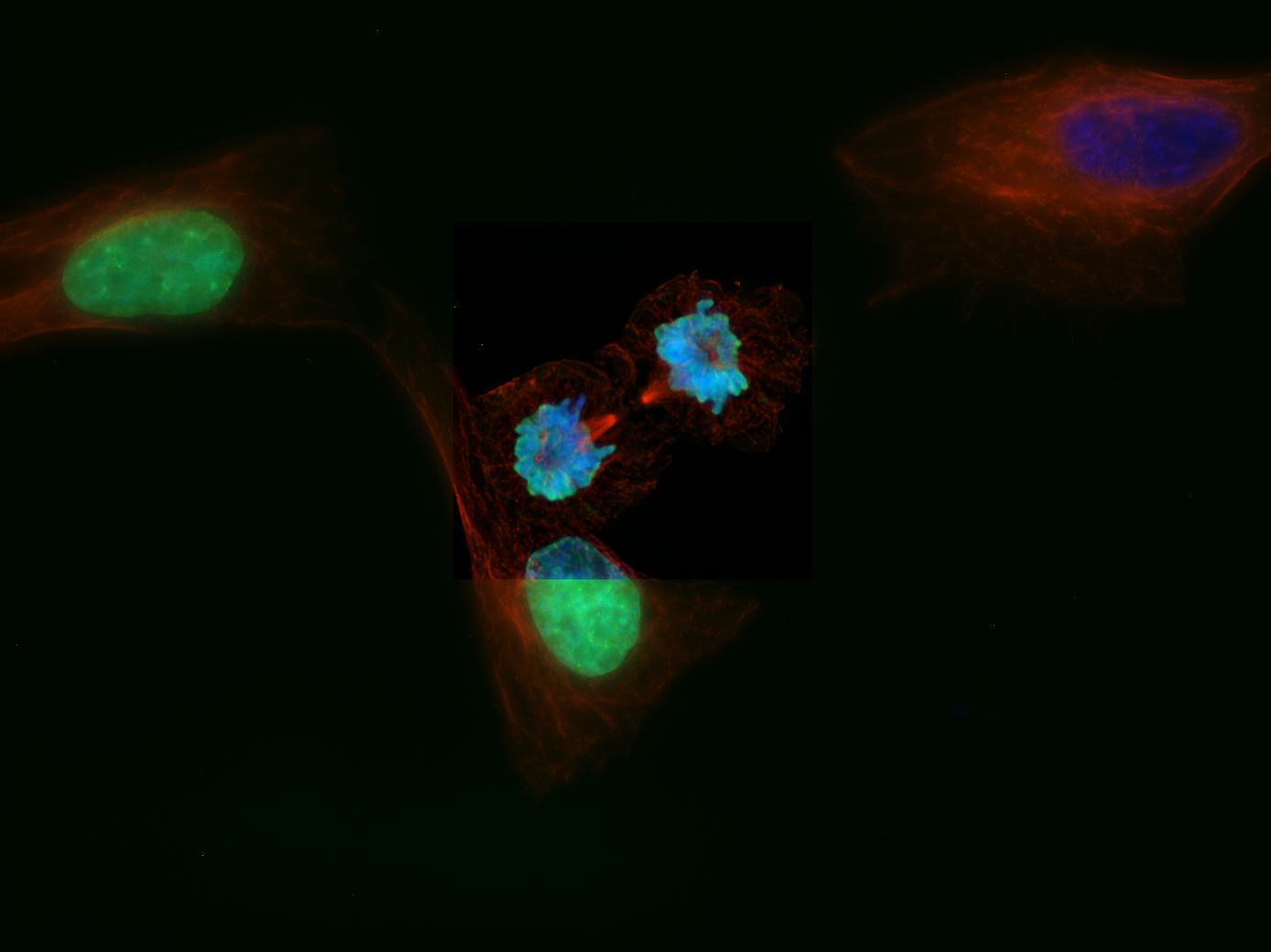

Supplement: Supplementary file 9 — Figure EV2 Source Data [file 44319_2026_833_MOESM9_ESM.zip › EV2A/EMBO_GFP-SCP4_Telophase_04_dec-Orthogonal Projection-06/EMBO_GFP-SCP4_Telophase_04_dec-Orthogonal Projection-06_c1-3.tif]

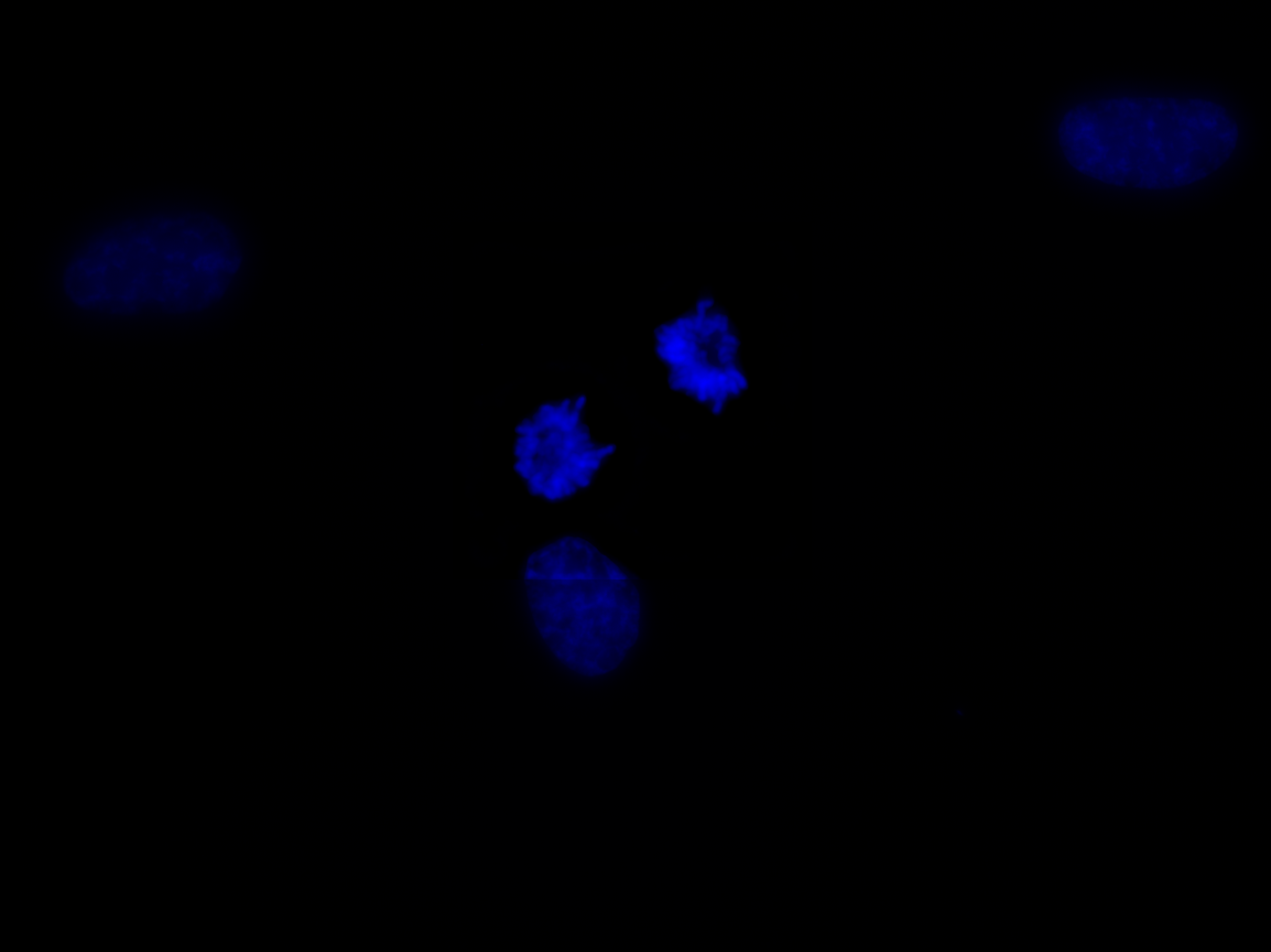

Supplement: Supplementary file 9 — Figure EV2 Source Data [file 44319_2026_833_MOESM9_ESM.zip › EV2A/EMBO_GFP-SCP4_Telophase_04_dec-Orthogonal Projection-06/EMBO_GFP-SCP4_Telophase_04_dec-Orthogonal Projection-06_c1.tif]

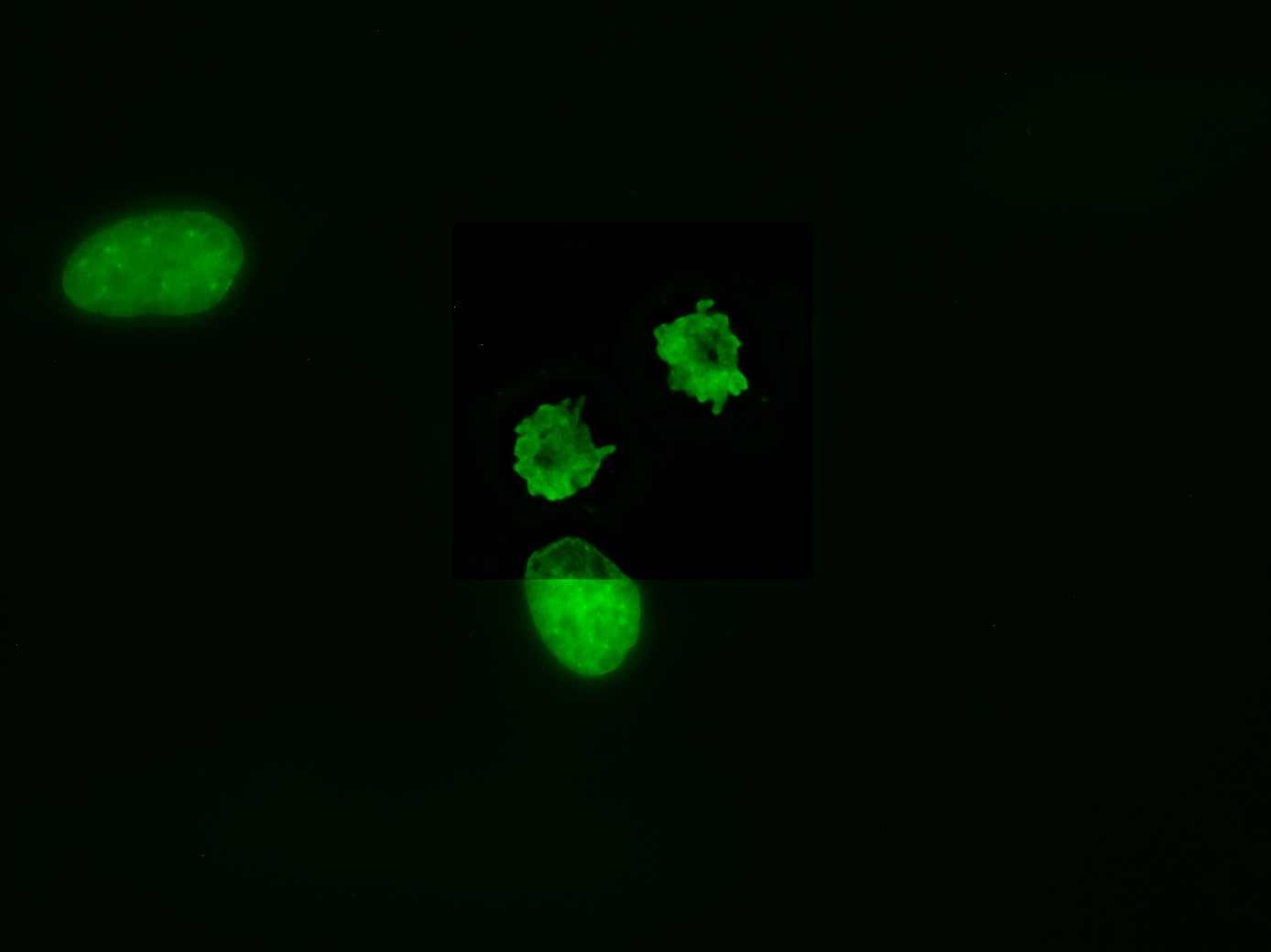

Supplement: Supplementary file 9 — Figure EV2 Source Data [file 44319_2026_833_MOESM9_ESM.zip › EV2A/EMBO_GFP-SCP4_Telophase_04_dec-Orthogonal Projection-06/EMBO_GFP-SCP4_Telophase_04_dec-Orthogonal Projection-06_c2.tif]

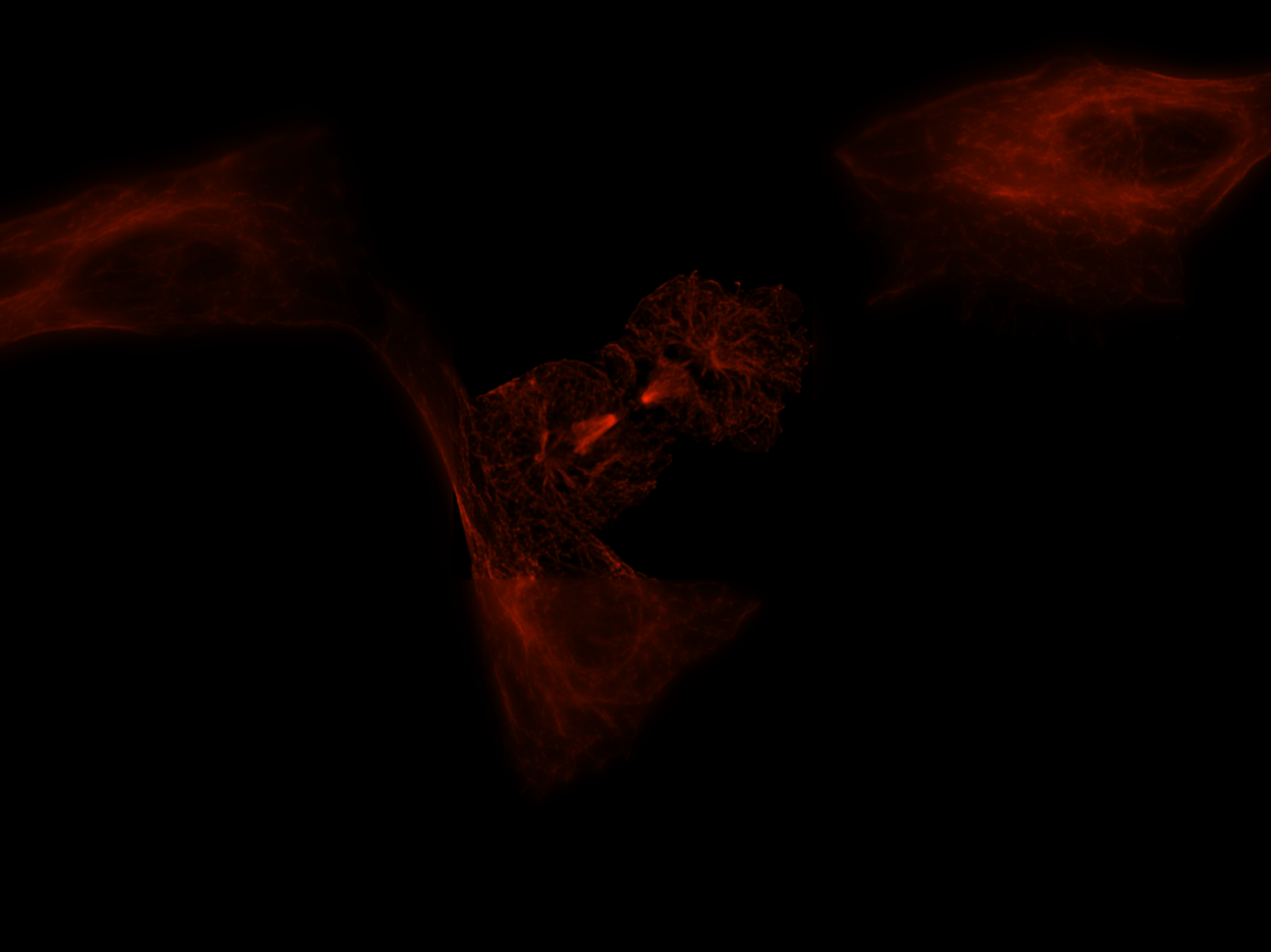

Supplement: Supplementary file 9 — Figure EV2 Source Data [file 44319_2026_833_MOESM9_ESM.zip › EV2A/EMBO_GFP-SCP4_Telophase_04_dec-Orthogonal Projection-06/EMBO_GFP-SCP4_Telophase_04_dec-Orthogonal Projection-06_c3.tif]

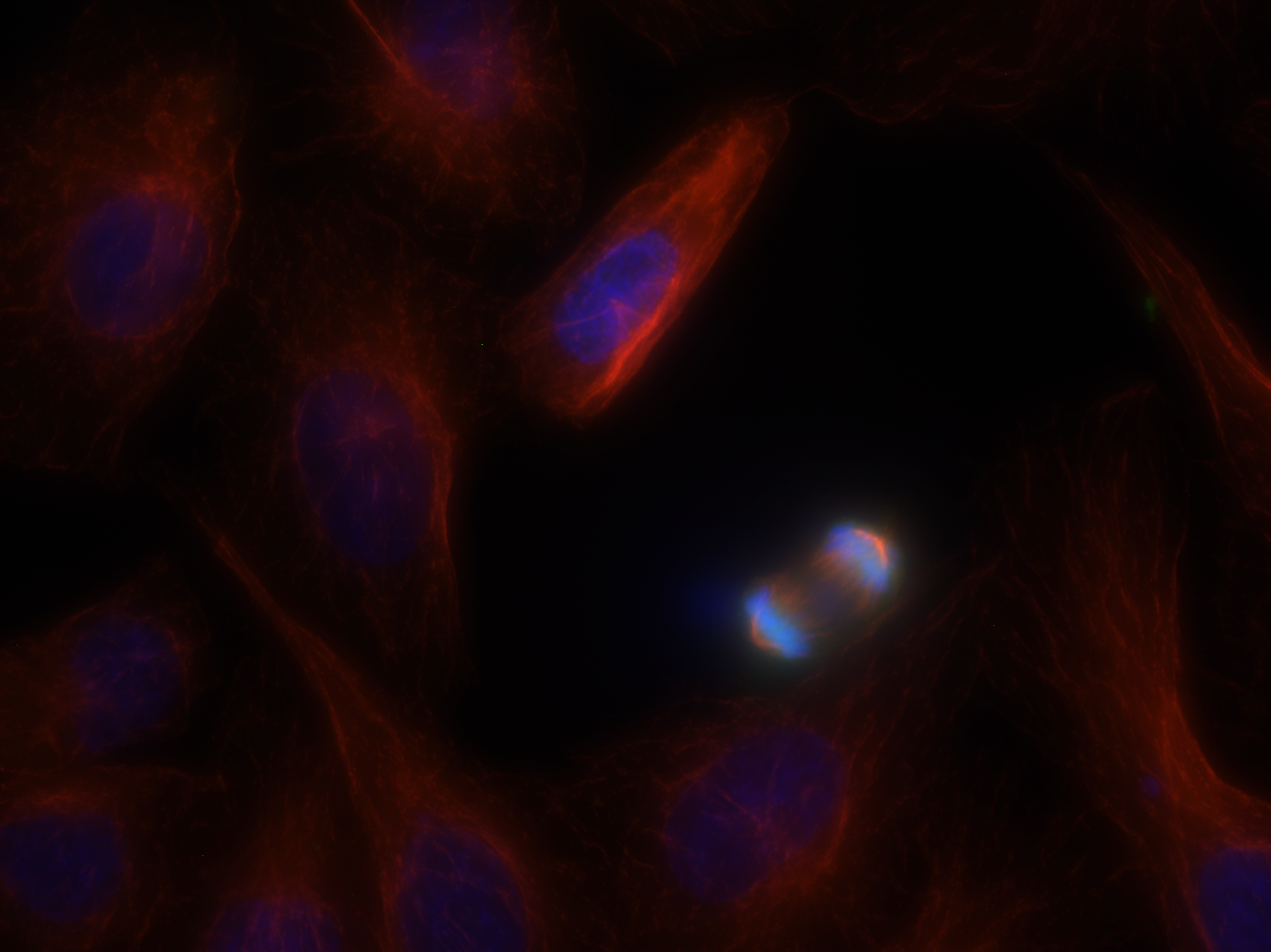

Supplement: Supplementary file 9 — Figure EV2 Source Data [file 44319_2026_833_MOESM9_ESM.zip › EV2B/Repo-Man/EMBO_GFP-Repo-Man_Anaphase_05-Orthogonal Projection-01/EMBO_GFP-Repo-Man_Anaphase_05-Orthogonal Projection-01_c1-3.tif]

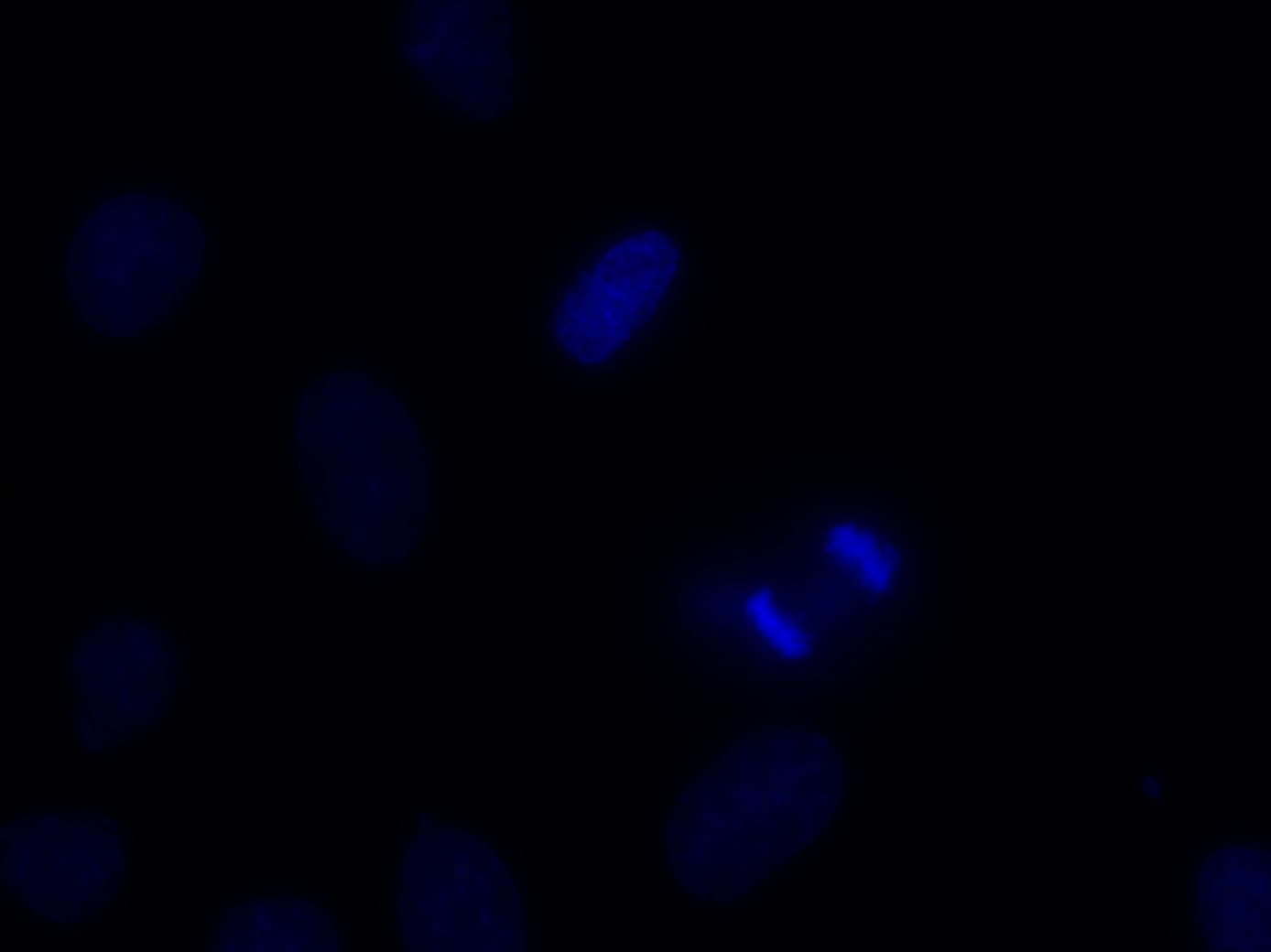

Supplement: Supplementary file 9 — Figure EV2 Source Data [file 44319_2026_833_MOESM9_ESM.zip › EV2B/Repo-Man/EMBO_GFP-Repo-Man_Anaphase_05-Orthogonal Projection-01/EMBO_GFP-Repo-Man_Anaphase_05-Orthogonal Projection-01_c1.tif]

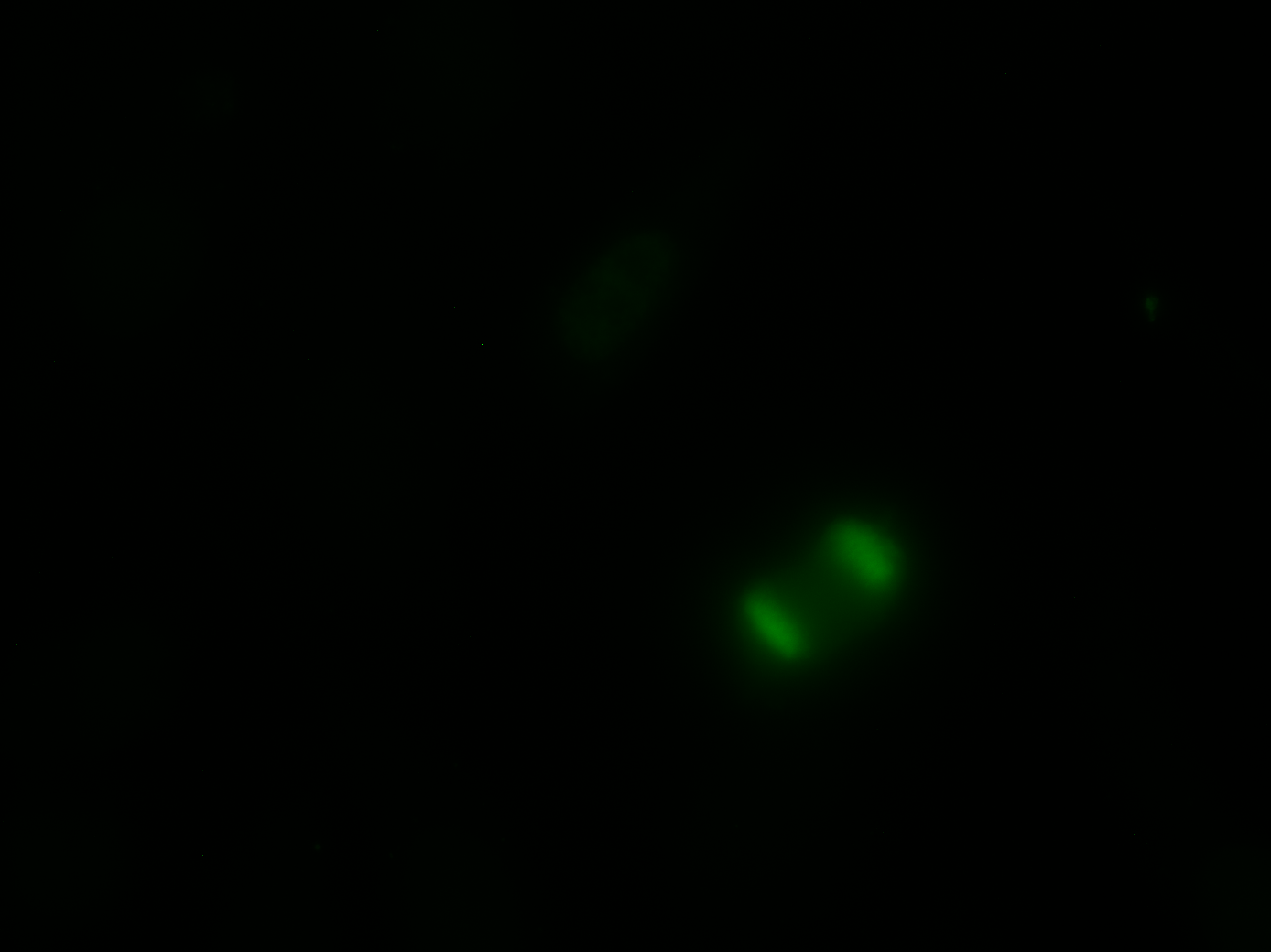

Supplement: Supplementary file 9 — Figure EV2 Source Data [file 44319_2026_833_MOESM9_ESM.zip › EV2B/Repo-Man/EMBO_GFP-Repo-Man_Anaphase_05-Orthogonal Projection-01/EMBO_GFP-Repo-Man_Anaphase_05-Orthogonal Projection-01_c2.tif]

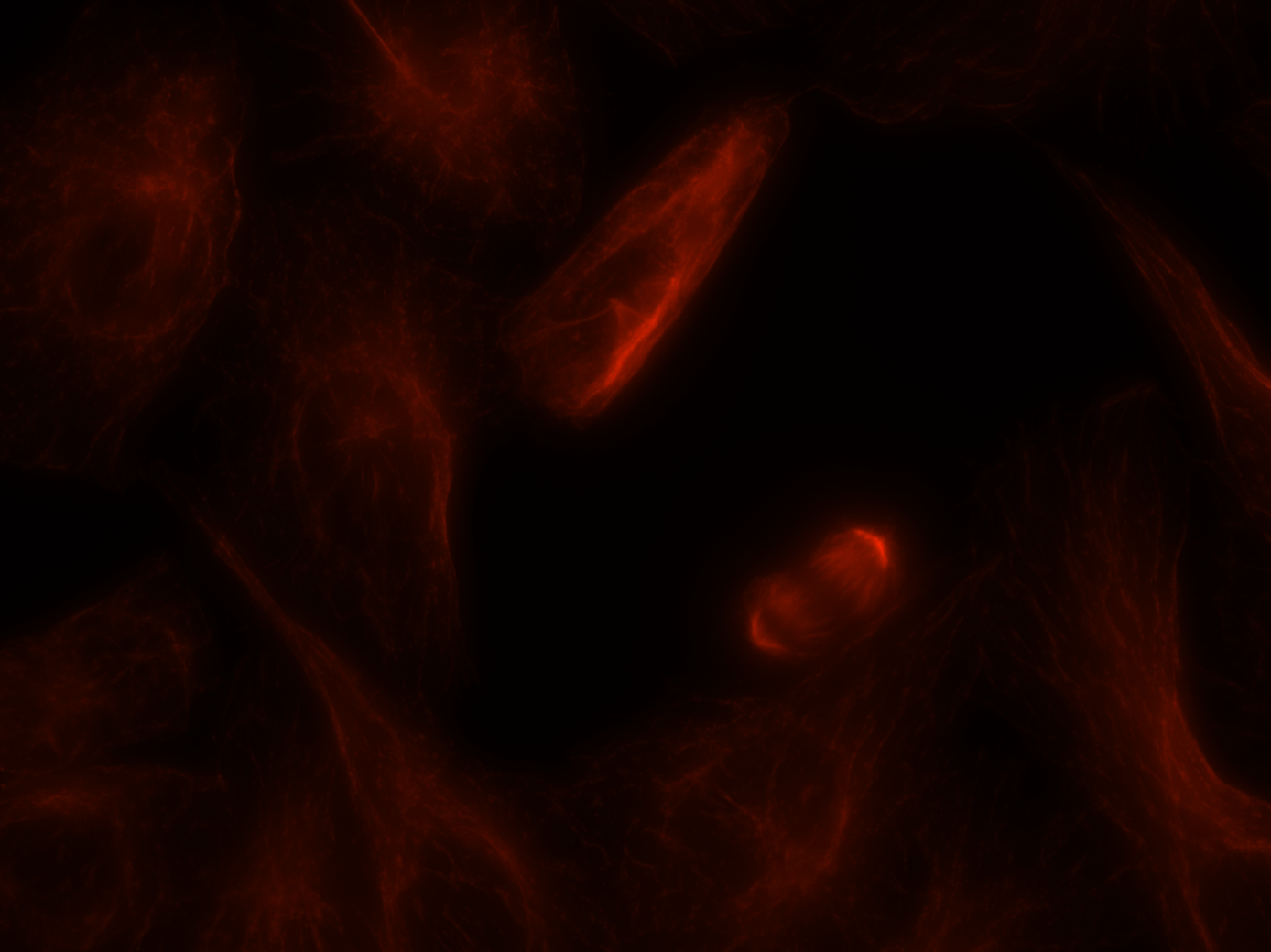

Supplement: Supplementary file 9 — Figure EV2 Source Data [file 44319_2026_833_MOESM9_ESM.zip › EV2B/Repo-Man/EMBO_GFP-Repo-Man_Anaphase_05-Orthogonal Projection-01/EMBO_GFP-Repo-Man_Anaphase_05-Orthogonal Projection-01_c3.tif]

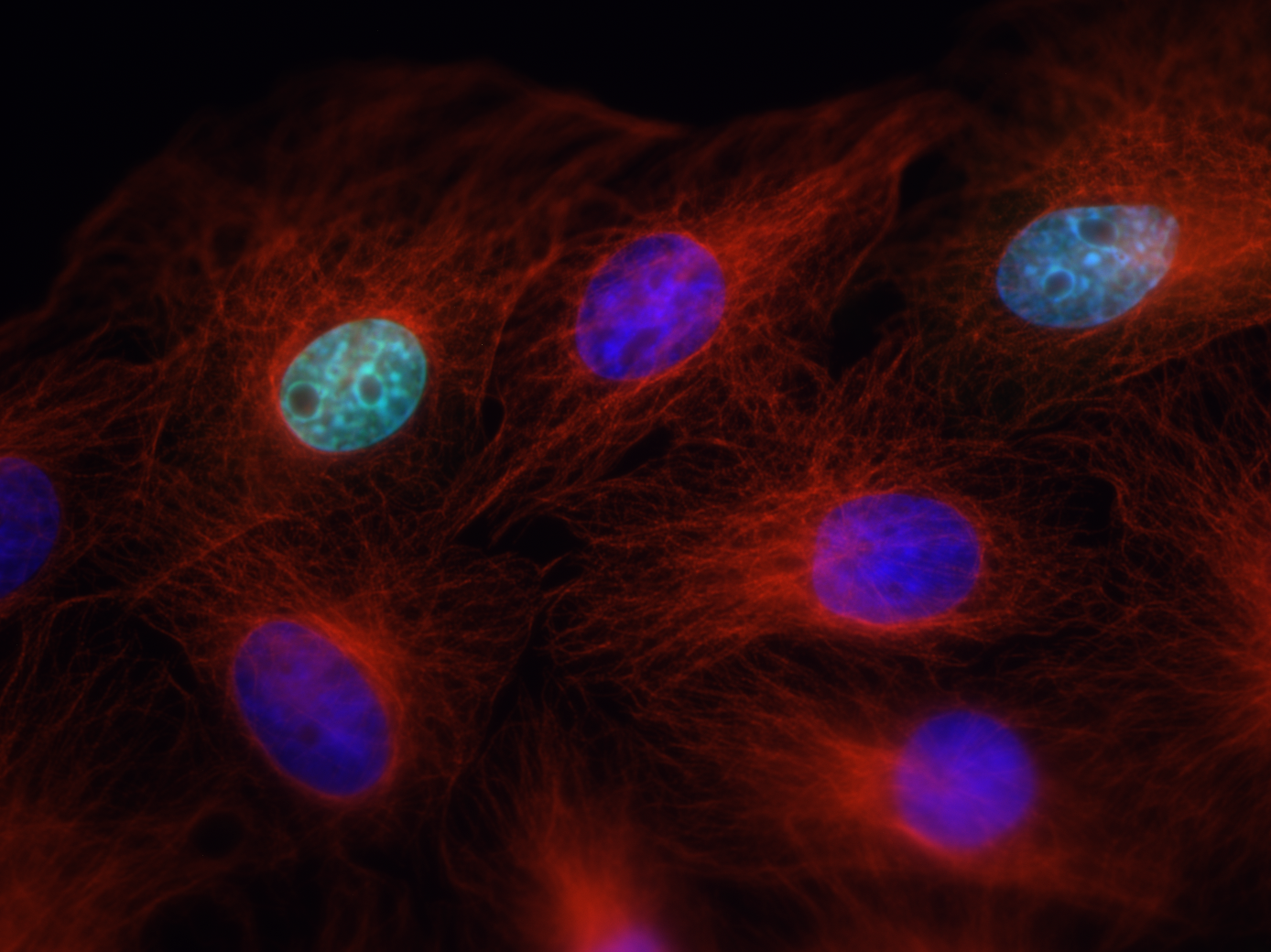

Supplement: Supplementary file 9 — Figure EV2 Source Data [file 44319_2026_833_MOESM9_ESM.zip › EV2B/Repo-Man/EMBO_GFP-Repo-Man_Interphase_03-Orthogonal Projection-02/EMBO_GFP-Repo-Man_Interphase_03-Orthogonal Projection-02_c1-3.tif]

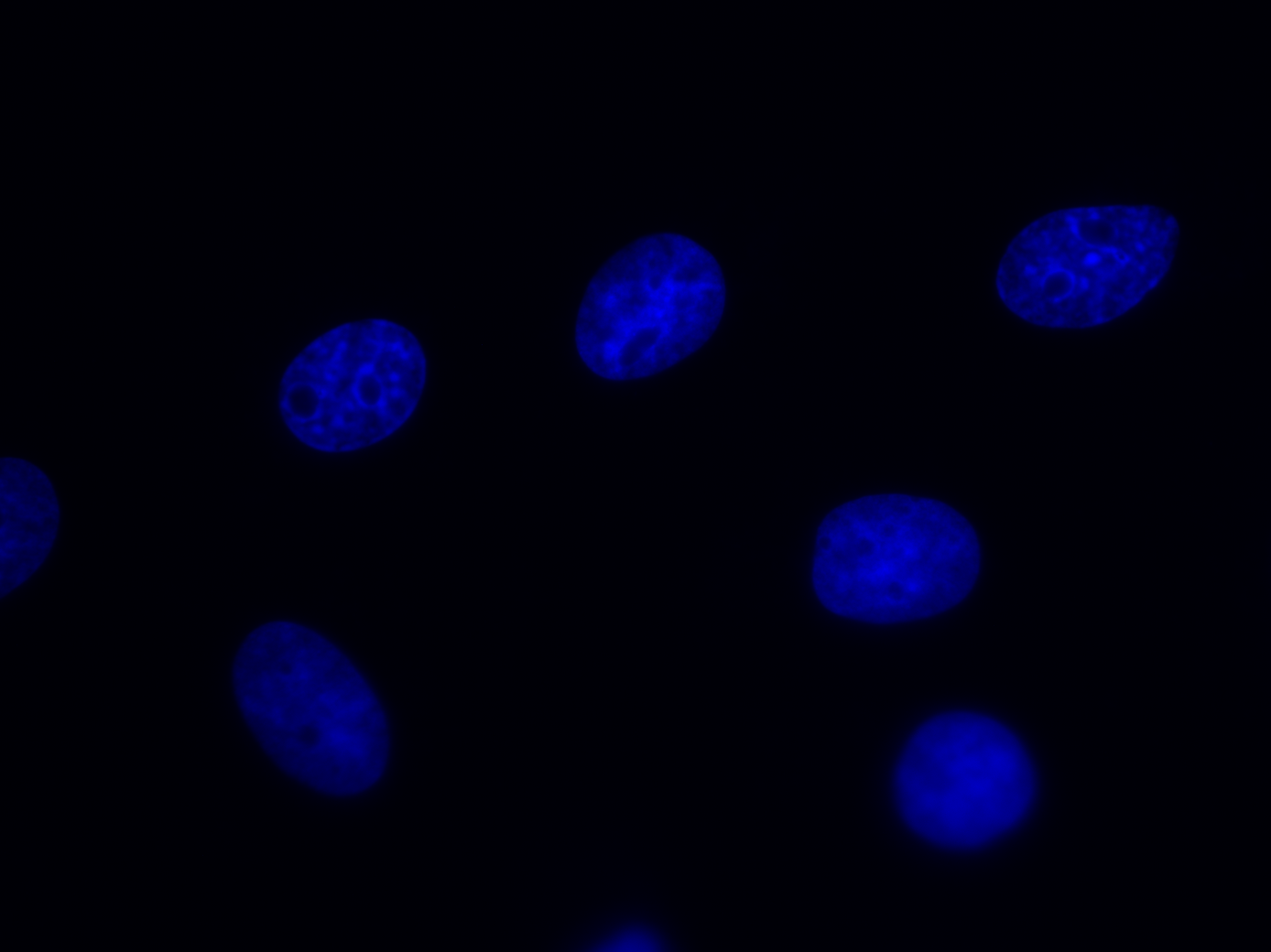

Supplement: Supplementary file 9 — Figure EV2 Source Data [file 44319_2026_833_MOESM9_ESM.zip › EV2B/Repo-Man/EMBO_GFP-Repo-Man_Interphase_03-Orthogonal Projection-02/EMBO_GFP-Repo-Man_Interphase_03-Orthogonal Projection-02_c1.tif]

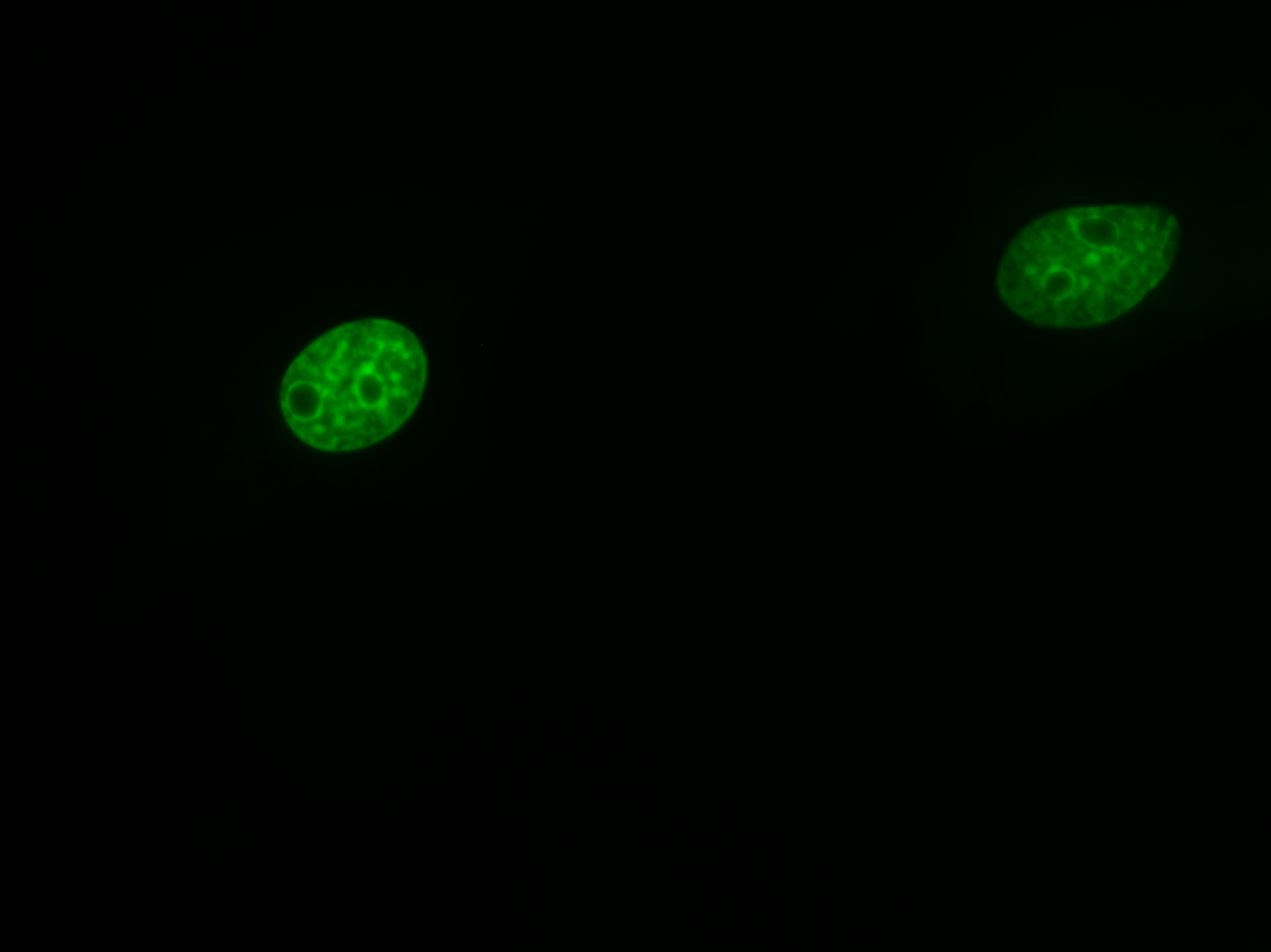

Supplement: Supplementary file 9 — Figure EV2 Source Data [file 44319_2026_833_MOESM9_ESM.zip › EV2B/Repo-Man/EMBO_GFP-Repo-Man_Interphase_03-Orthogonal Projection-02/EMBO_GFP-Repo-Man_Interphase_03-Orthogonal Projection-02_c2.tif]

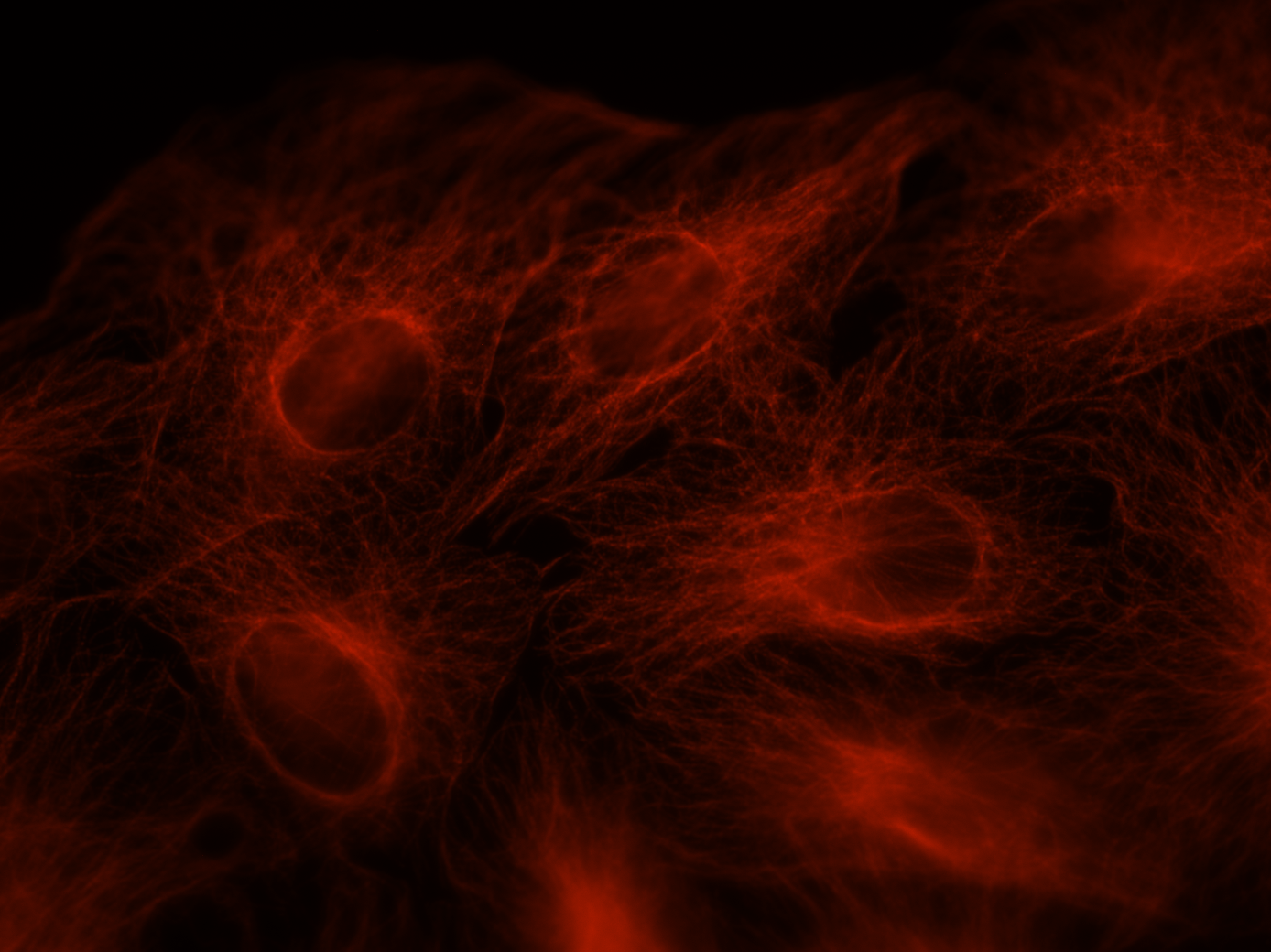

Supplement: Supplementary file 9 — Figure EV2 Source Data [file 44319_2026_833_MOESM9_ESM.zip › EV2B/Repo-Man/EMBO_GFP-Repo-Man_Interphase_03-Orthogonal Projection-02/EMBO_GFP-Repo-Man_Interphase_03-Orthogonal Projection-02_c3.tif]

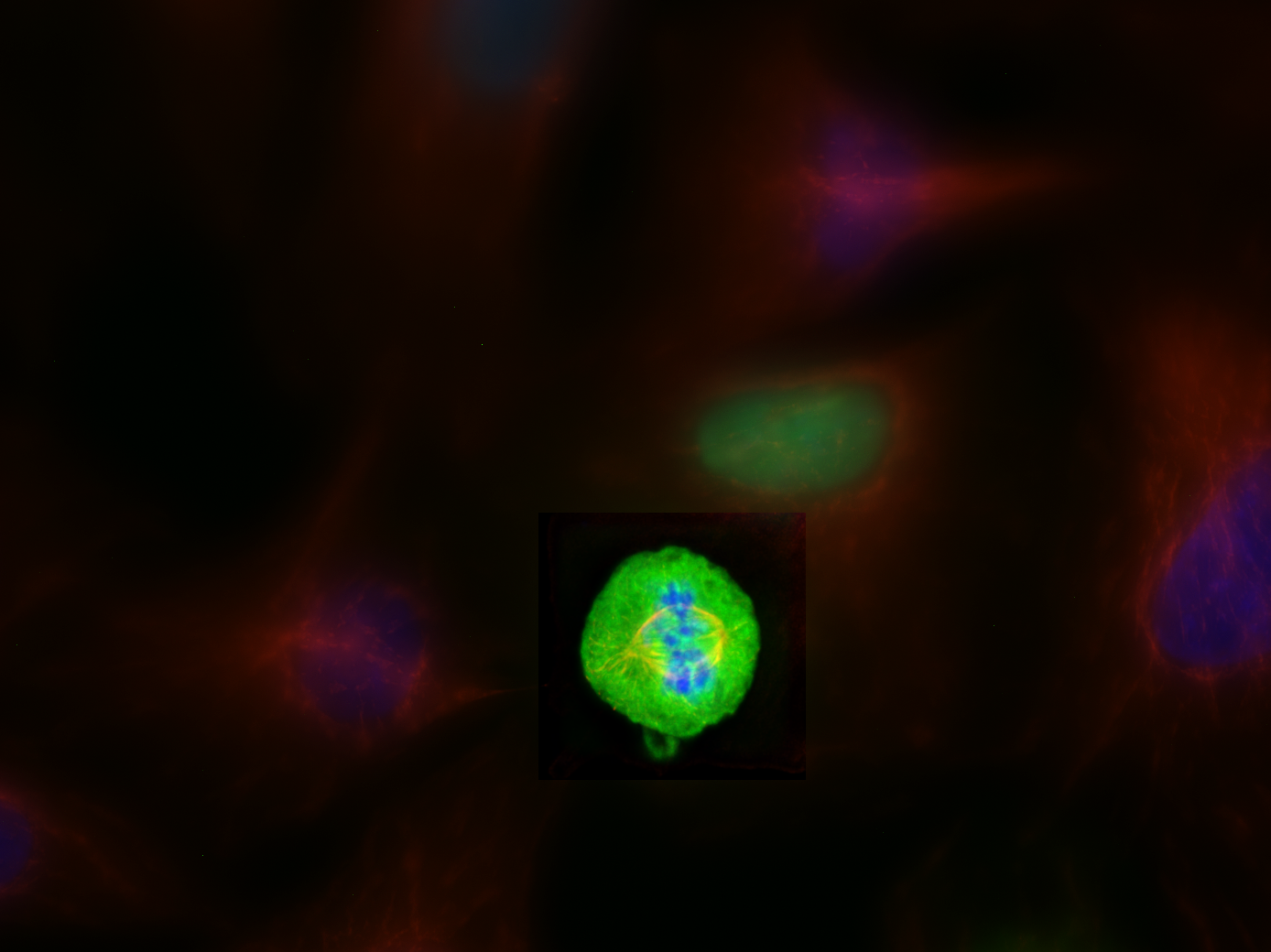

Supplement: Supplementary file 9 — Figure EV2 Source Data [file 44319_2026_833_MOESM9_ESM.zip › EV2B/Repo-Man/EMBO_GFP-Repo-Man_Metaphase_07_dec-Orthogonal Projection-03/EMBO_GFP-Repo-Man_Metaphase_07_dec-Orthogonal Projection-03_c1-3.tif]

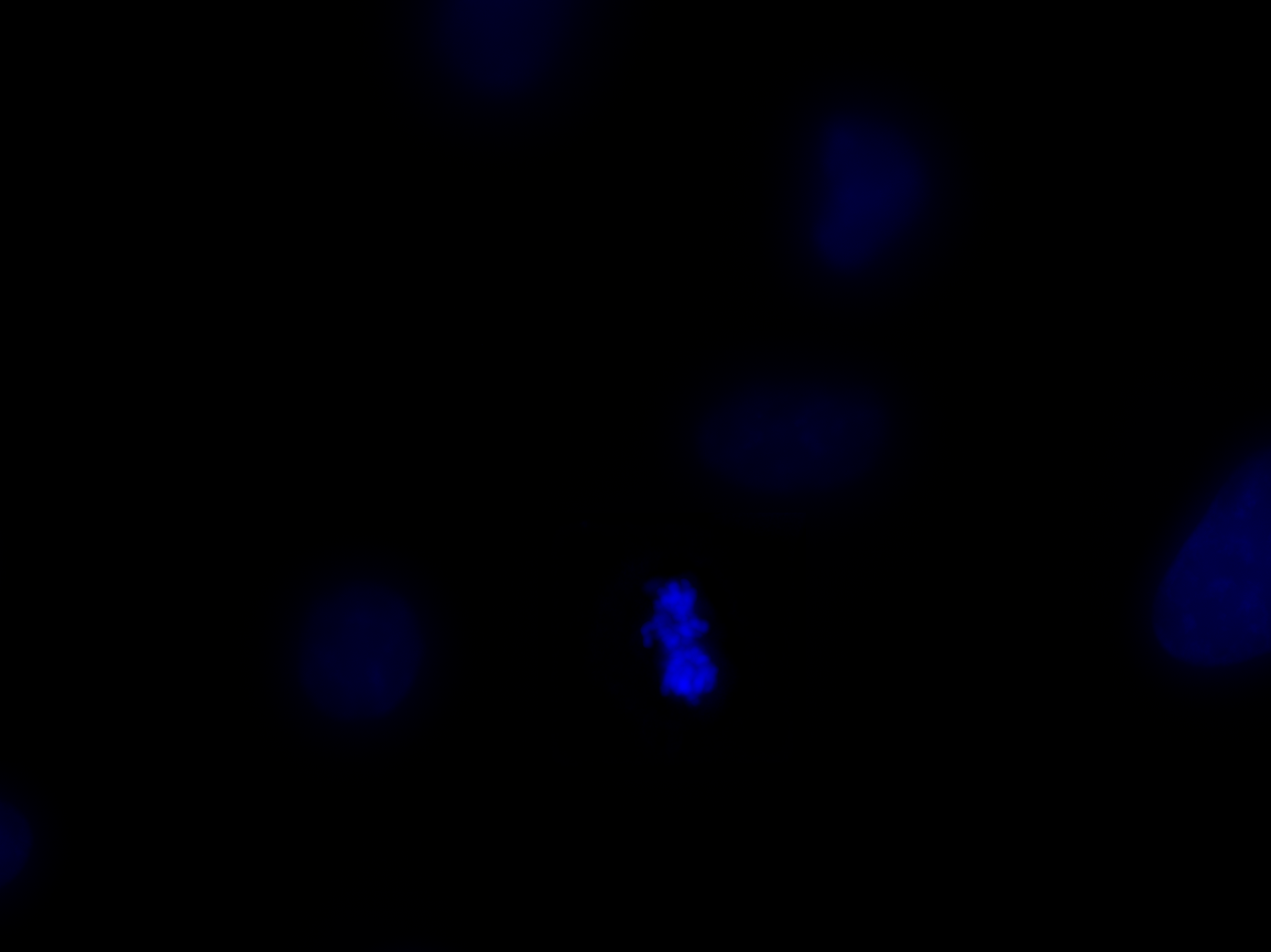

Supplement: Supplementary file 9 — Figure EV2 Source Data [file 44319_2026_833_MOESM9_ESM.zip › EV2B/Repo-Man/EMBO_GFP-Repo-Man_Metaphase_07_dec-Orthogonal Projection-03/EMBO_GFP-Repo-Man_Metaphase_07_dec-Orthogonal Projection-03_c1.tif]

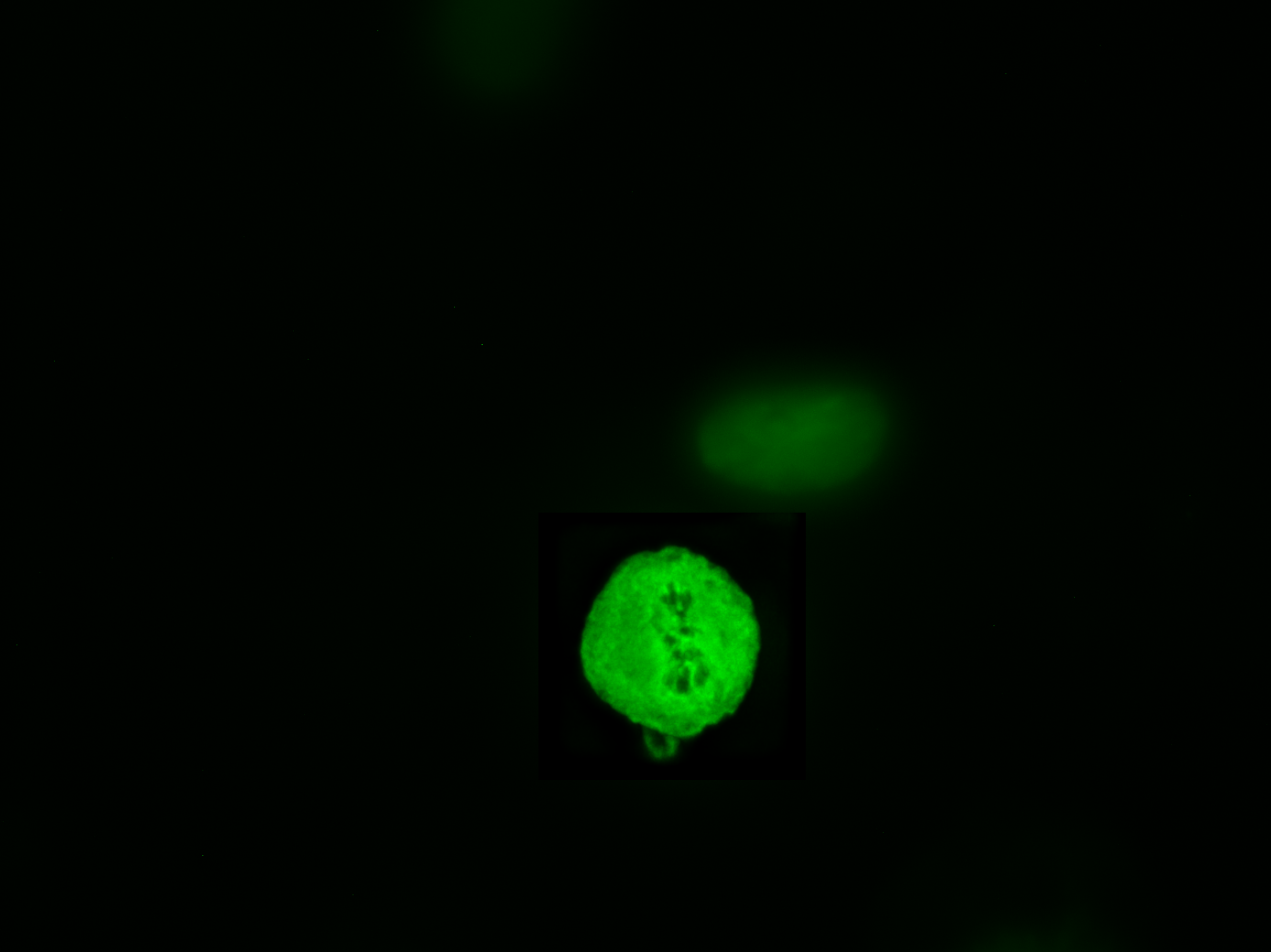

Supplement: Supplementary file 9 — Figure EV2 Source Data [file 44319_2026_833_MOESM9_ESM.zip › EV2B/Repo-Man/EMBO_GFP-Repo-Man_Metaphase_07_dec-Orthogonal Projection-03/EMBO_GFP-Repo-Man_Metaphase_07_dec-Orthogonal Projection-03_c2.tif]

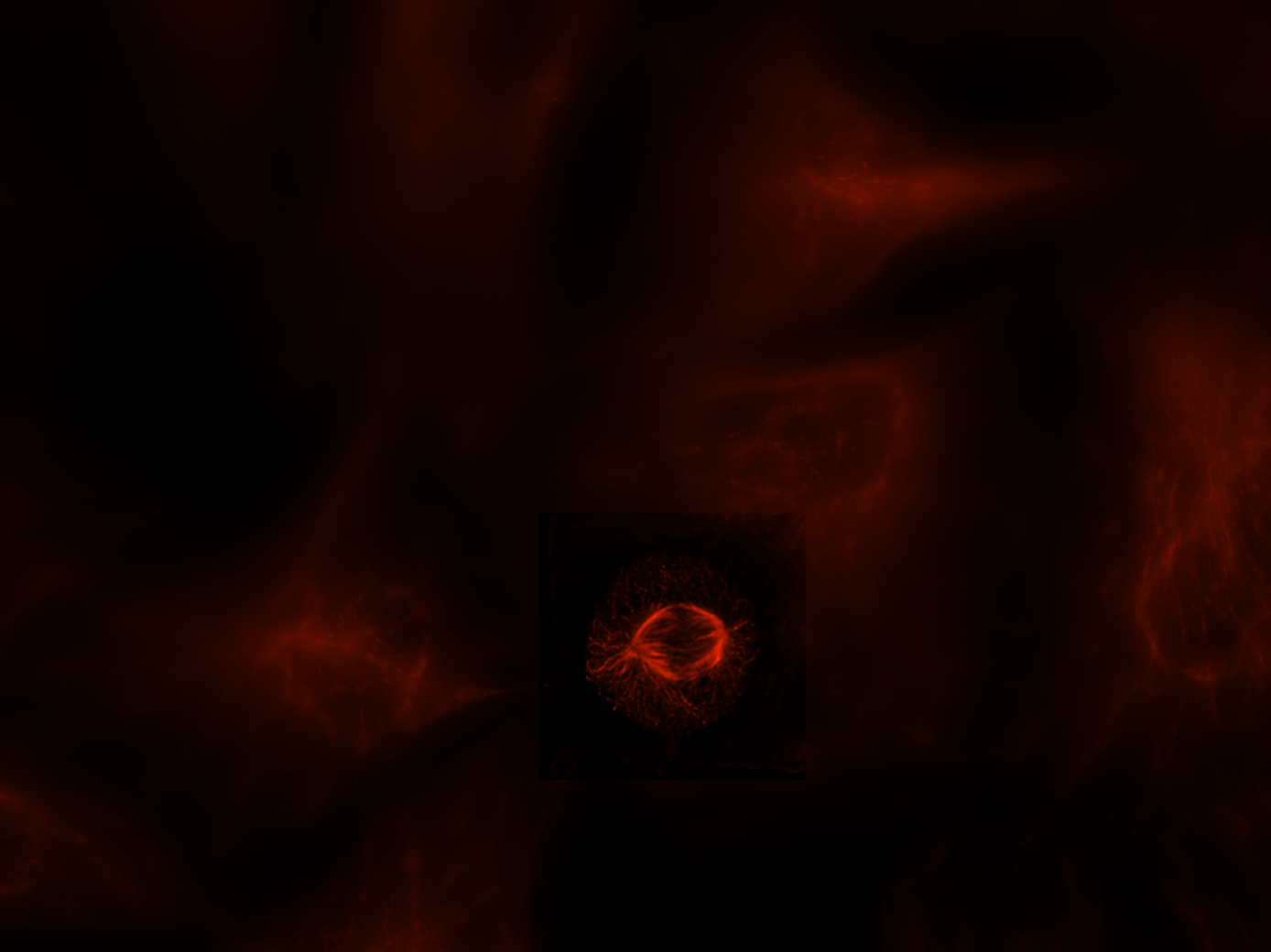

Supplement: Supplementary file 9 — Figure EV2 Source Data [file 44319_2026_833_MOESM9_ESM.zip › EV2B/Repo-Man/EMBO_GFP-Repo-Man_Metaphase_07_dec-Orthogonal Projection-03/EMBO_GFP-Repo-Man_Metaphase_07_dec-Orthogonal Projection-03_c3.tif]

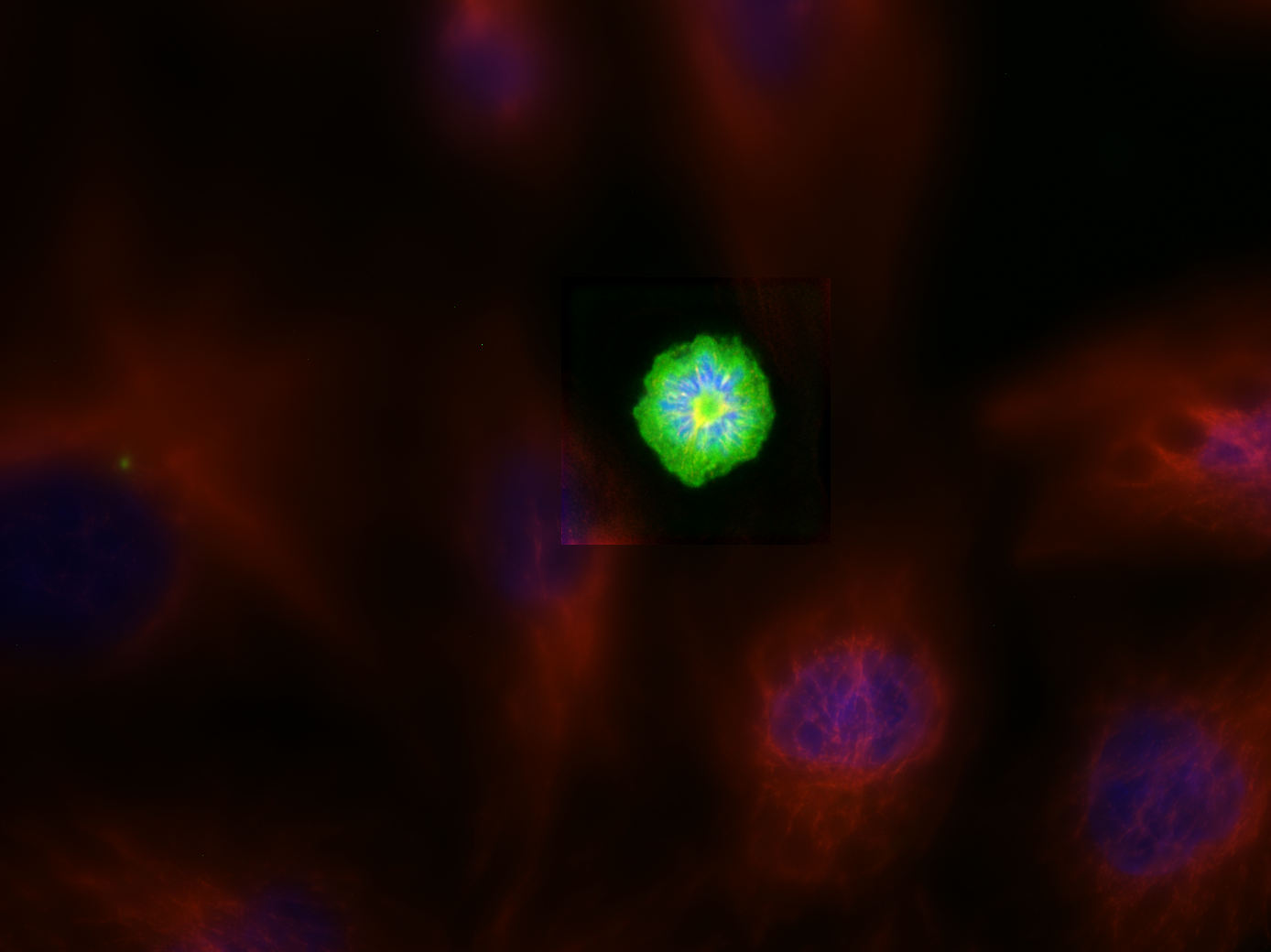

Supplement: Supplementary file 9 — Figure EV2 Source Data [file 44319_2026_833_MOESM9_ESM.zip › EV2B/Repo-Man/EMBO_GFP-Repo-Man_Prometaphase_03_dec-Orthogonal Projection-04/EMBO_GFP-Repo-Man_Prometaphase_03_dec-Orthogonal Projection-04_c1-3.tif]

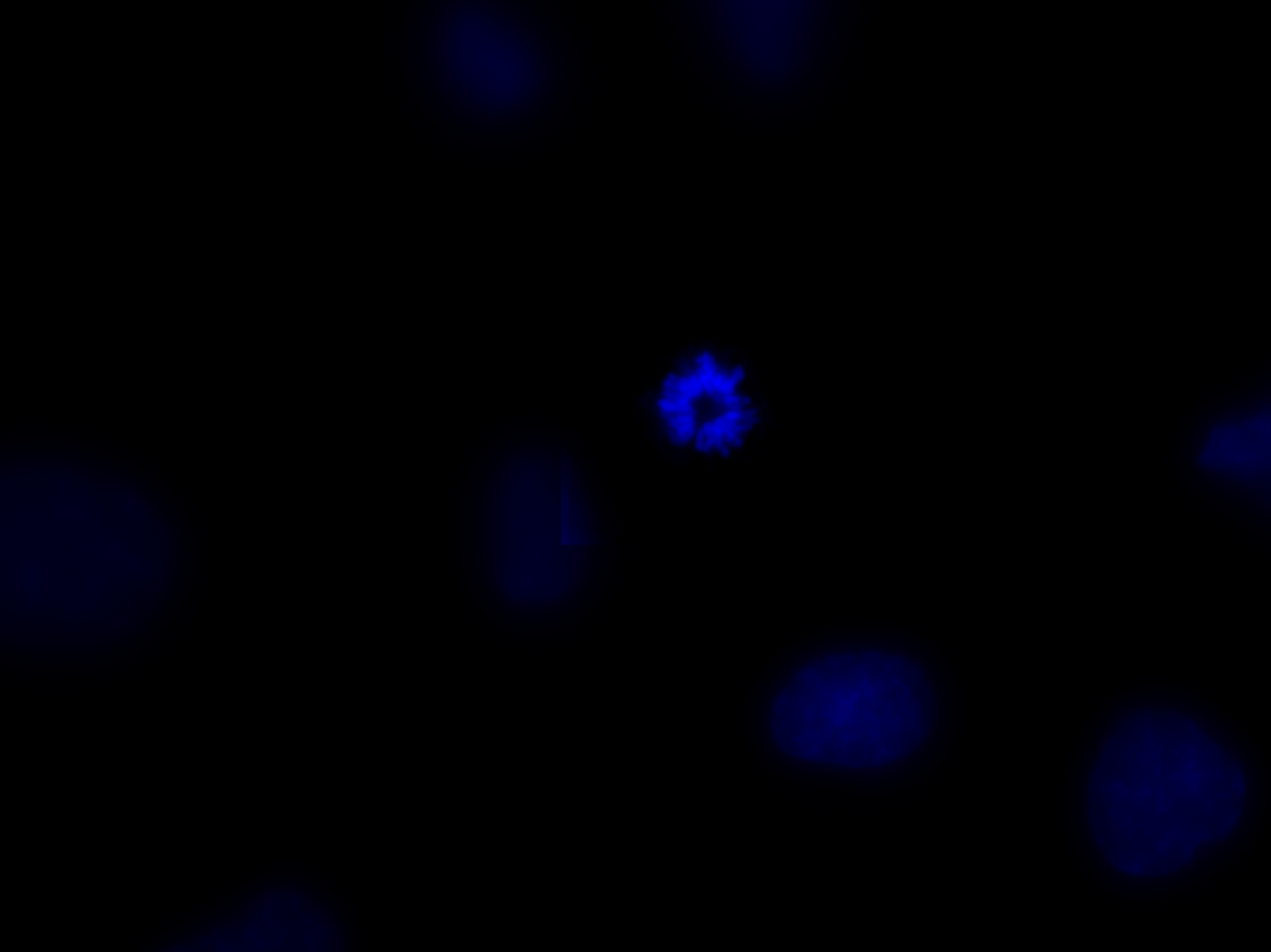

Supplement: Supplementary file 9 — Figure EV2 Source Data [file 44319_2026_833_MOESM9_ESM.zip › EV2B/Repo-Man/EMBO_GFP-Repo-Man_Prometaphase_03_dec-Orthogonal Projection-04/EMBO_GFP-Repo-Man_Prometaphase_03_dec-Orthogonal Projection-04_c1.tif]

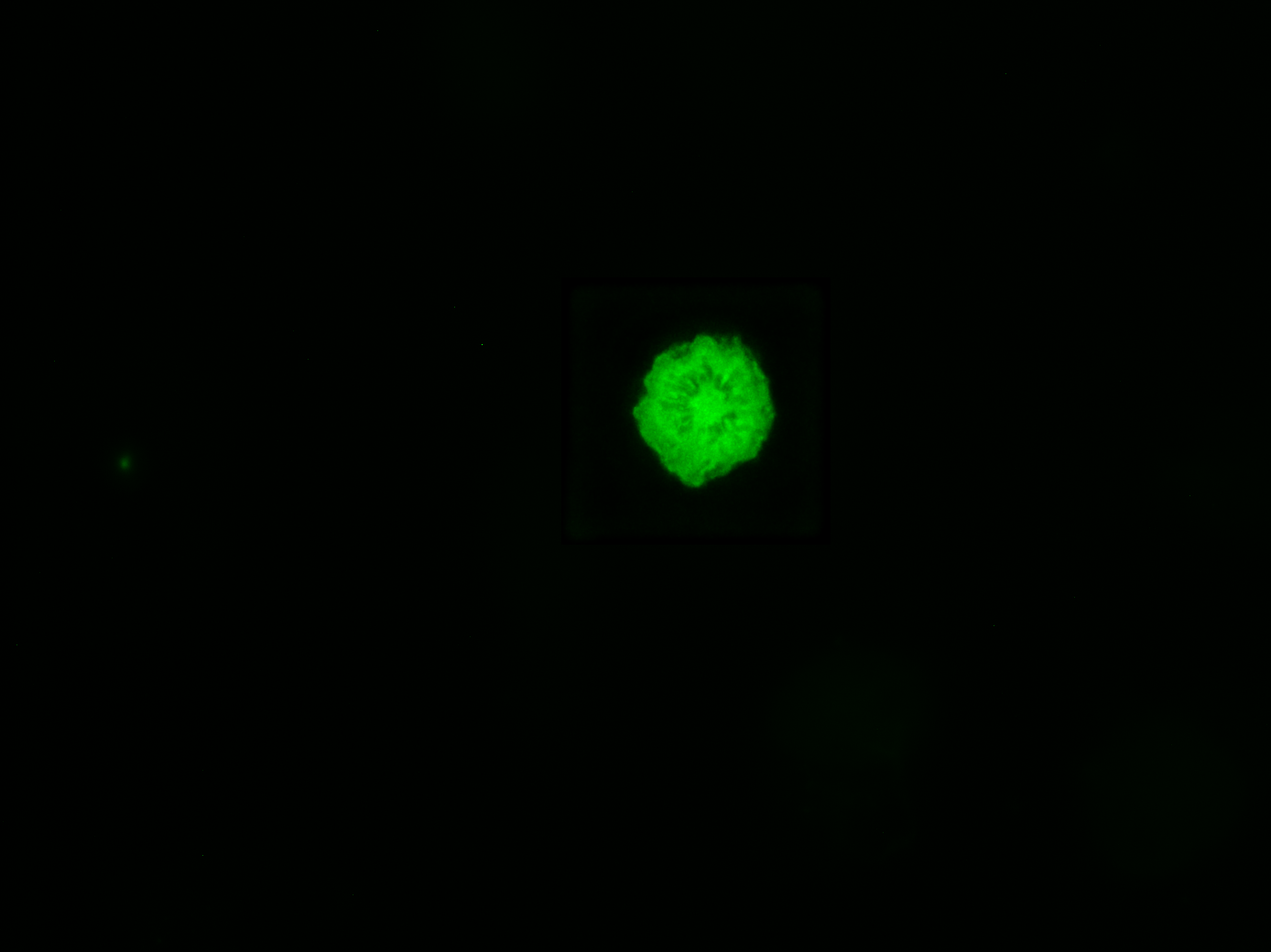

Supplement: Supplementary file 9 — Figure EV2 Source Data [file 44319_2026_833_MOESM9_ESM.zip › EV2B/Repo-Man/EMBO_GFP-Repo-Man_Prometaphase_03_dec-Orthogonal Projection-04/EMBO_GFP-Repo-Man_Prometaphase_03_dec-Orthogonal Projection-04_c2.tif]

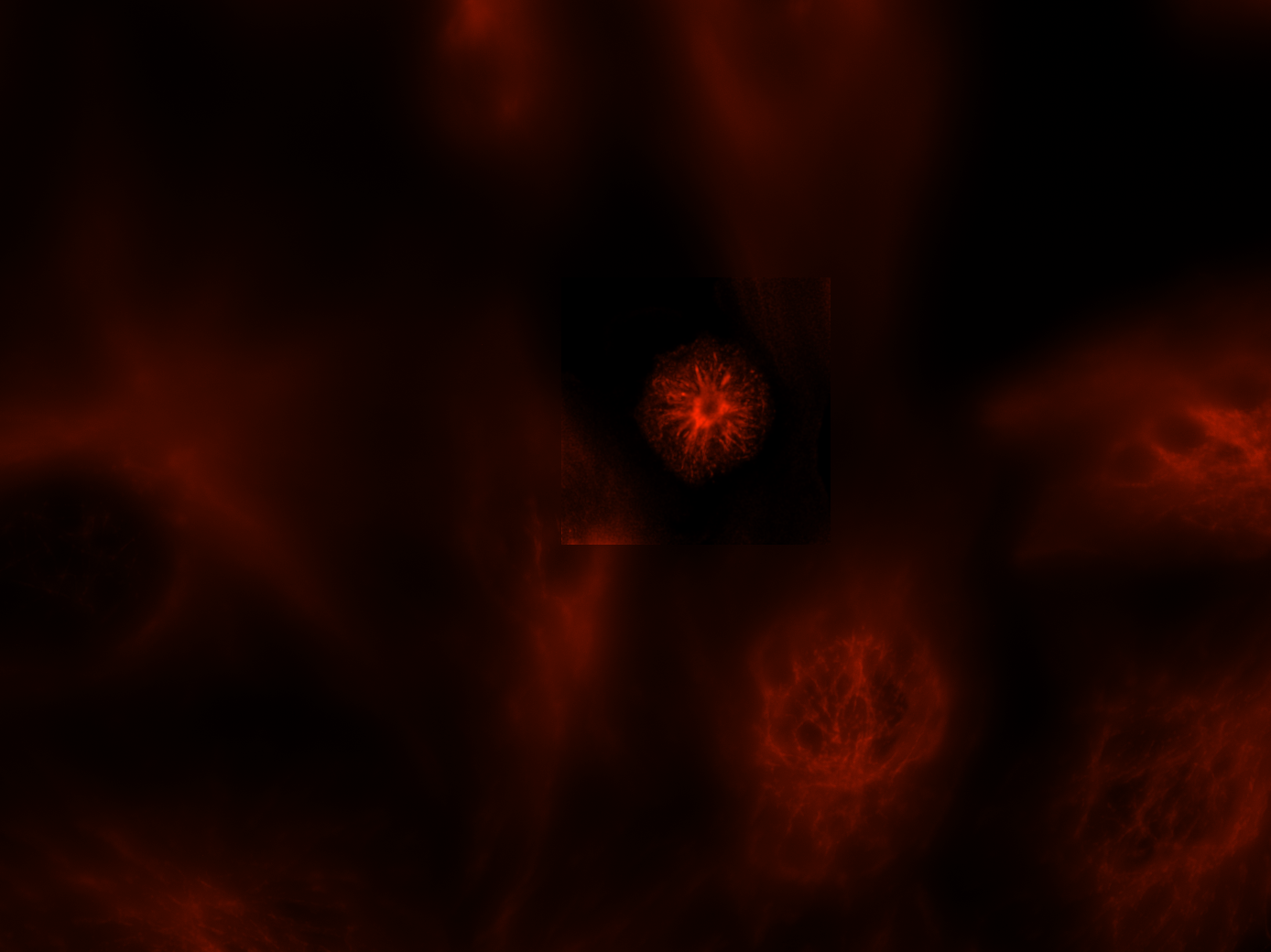

Supplement: Supplementary file 9 — Figure EV2 Source Data [file 44319_2026_833_MOESM9_ESM.zip › EV2B/Repo-Man/EMBO_GFP-Repo-Man_Prometaphase_03_dec-Orthogonal Projection-04/EMBO_GFP-Repo-Man_Prometaphase_03_dec-Orthogonal Projection-04_c3.tif]

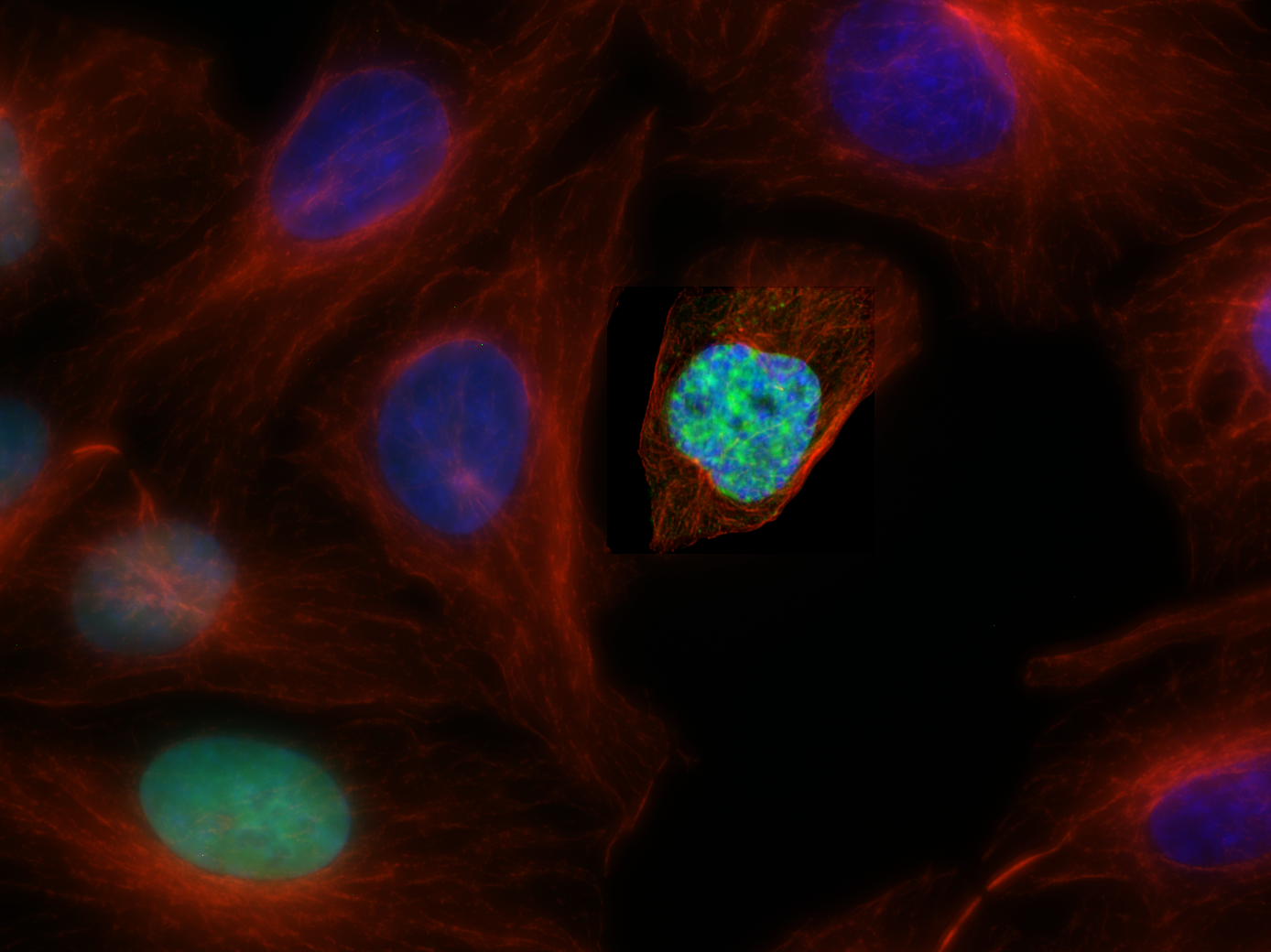

Supplement: Supplementary file 9 — Figure EV2 Source Data [file 44319_2026_833_MOESM9_ESM.zip › EV2B/Repo-Man/EMBO_GFP-Repo-Man_Prophase_04_dec-Orthogonal Projection-05/EMBO_GFP-Repo-Man_Prophase_04_dec-Orthogonal Projection-05_c1-3.tif]

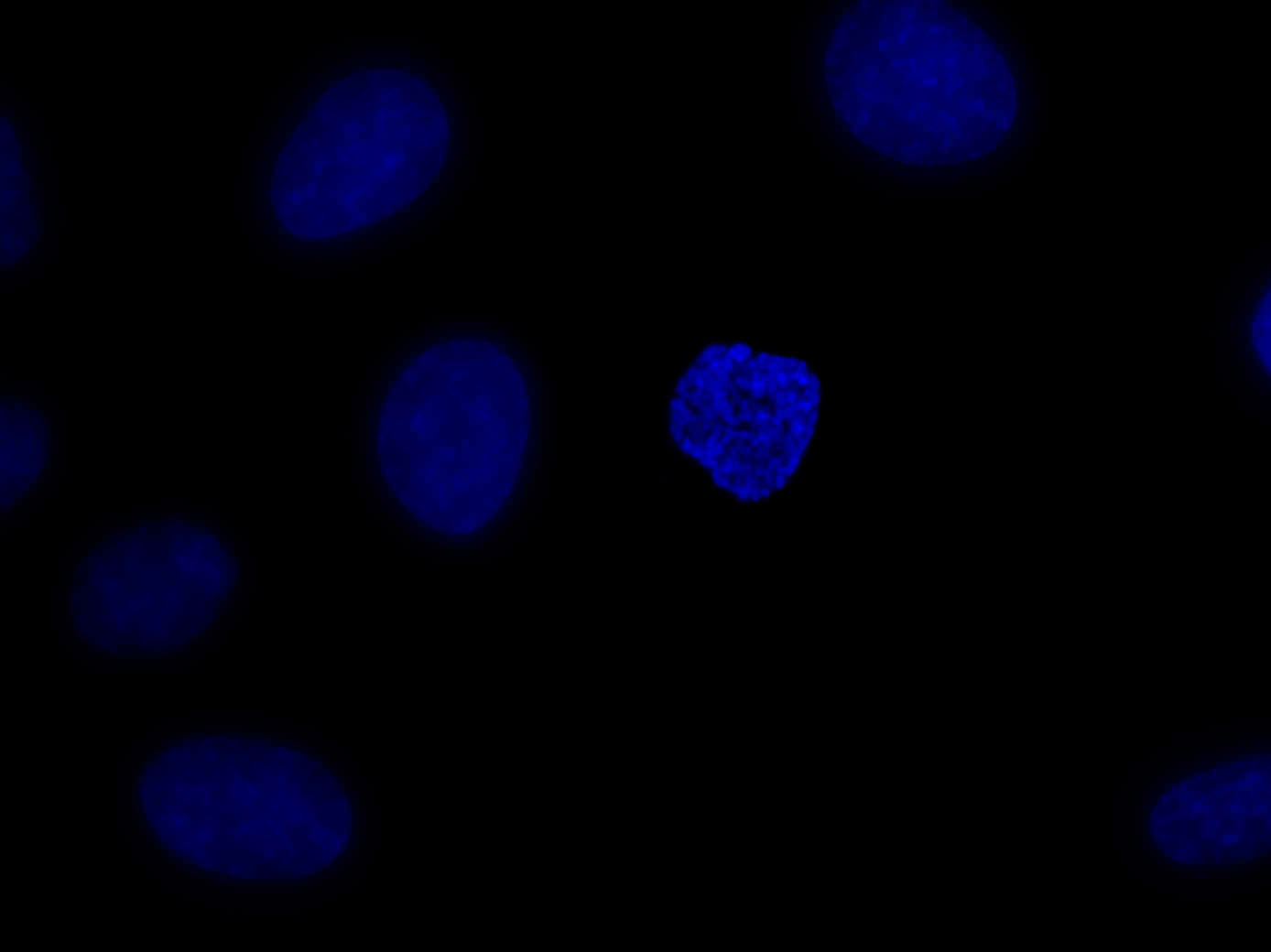

Supplement: Supplementary file 9 — Figure EV2 Source Data [file 44319_2026_833_MOESM9_ESM.zip › EV2B/Repo-Man/EMBO_GFP-Repo-Man_Prophase_04_dec-Orthogonal Projection-05/EMBO_GFP-Repo-Man_Prophase_04_dec-Orthogonal Projection-05_c1.tif]

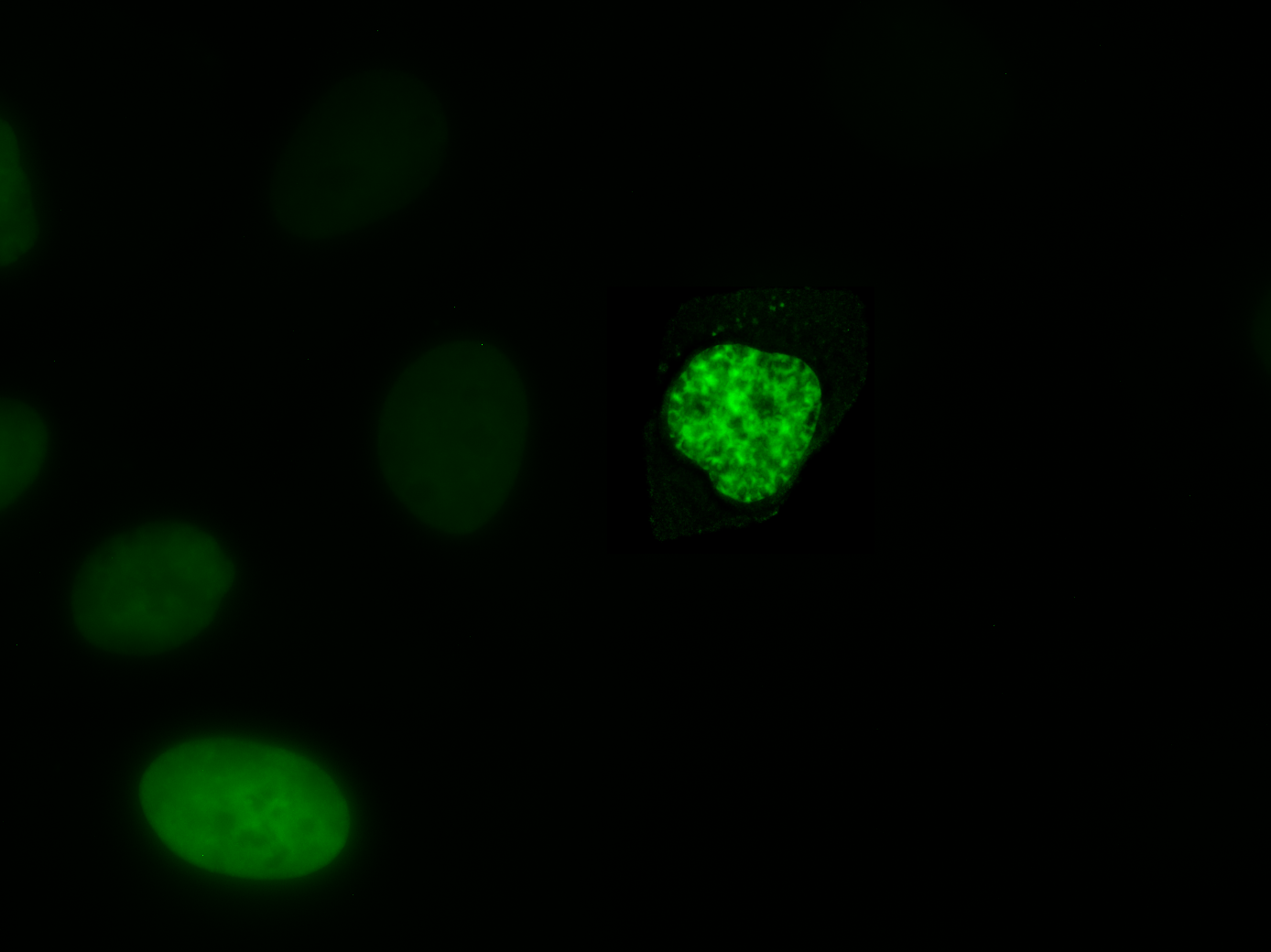

Supplement: Supplementary file 9 — Figure EV2 Source Data [file 44319_2026_833_MOESM9_ESM.zip › EV2B/Repo-Man/EMBO_GFP-Repo-Man_Prophase_04_dec-Orthogonal Projection-05/EMBO_GFP-Repo-Man_Prophase_04_dec-Orthogonal Projection-05_c2.tif]

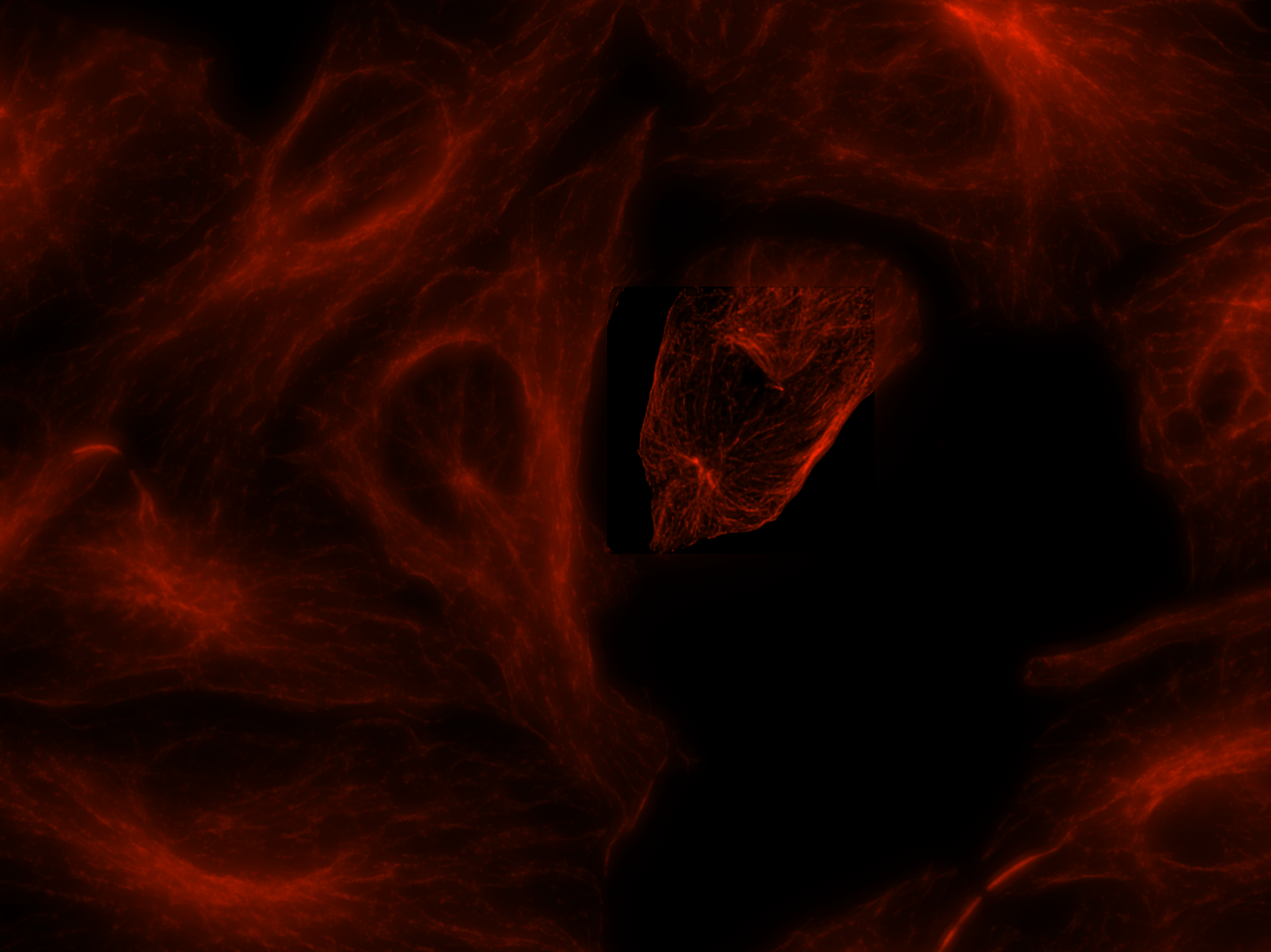

Supplement: Supplementary file 9 — Figure EV2 Source Data [file 44319_2026_833_MOESM9_ESM.zip › EV2B/Repo-Man/EMBO_GFP-Repo-Man_Prophase_04_dec-Orthogonal Projection-05/EMBO_GFP-Repo-Man_Prophase_04_dec-Orthogonal Projection-05_c3.tif]

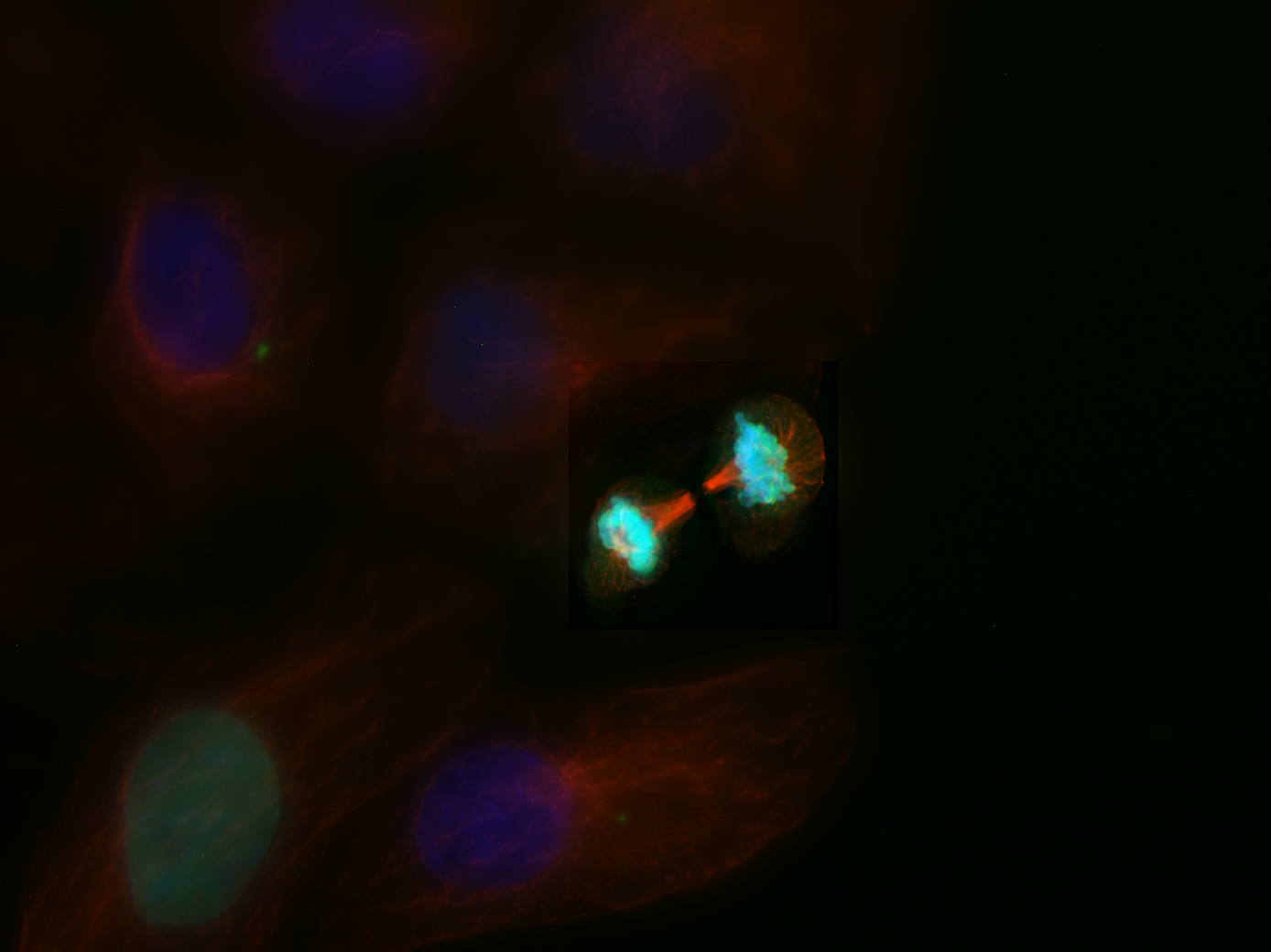

Supplement: Supplementary file 9 — Figure EV2 Source Data [file 44319_2026_833_MOESM9_ESM.zip › EV2B/Repo-Man/EMBO_GFP-Repo-Man_Telophase_03_dec-Orthogonal Projection-08/EMBO_GFP-Repo-Man_Telophase_03_dec-Orthogonal Projection-08_c1-3.tif]

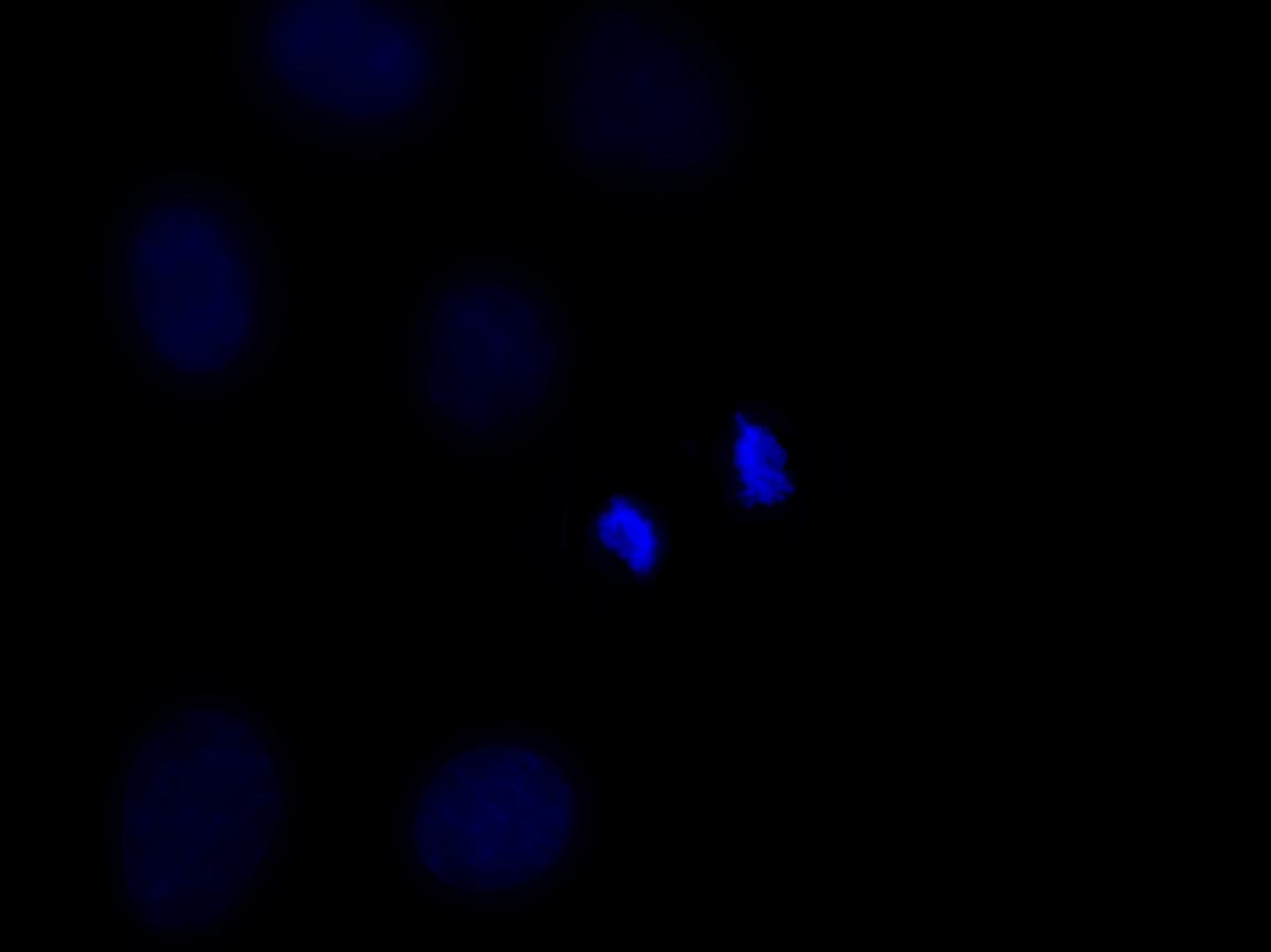

Supplement: Supplementary file 9 — Figure EV2 Source Data [file 44319_2026_833_MOESM9_ESM.zip › EV2B/Repo-Man/EMBO_GFP-Repo-Man_Telophase_03_dec-Orthogonal Projection-08/EMBO_GFP-Repo-Man_Telophase_03_dec-Orthogonal Projection-08_c1.tif]

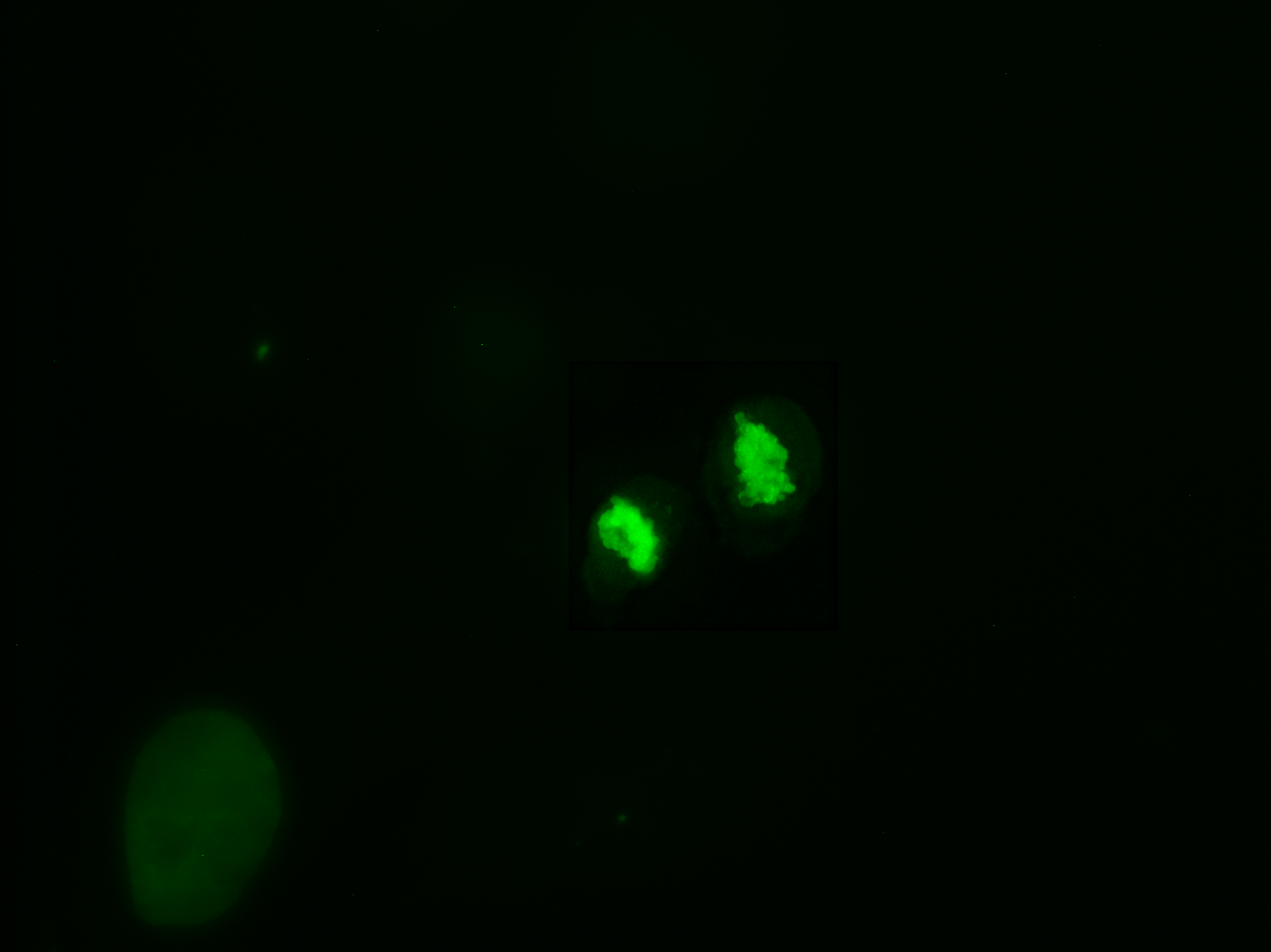

Supplement: Supplementary file 9 — Figure EV2 Source Data [file 44319_2026_833_MOESM9_ESM.zip › EV2B/Repo-Man/EMBO_GFP-Repo-Man_Telophase_03_dec-Orthogonal Projection-08/EMBO_GFP-Repo-Man_Telophase_03_dec-Orthogonal Projection-08_c2.tif]

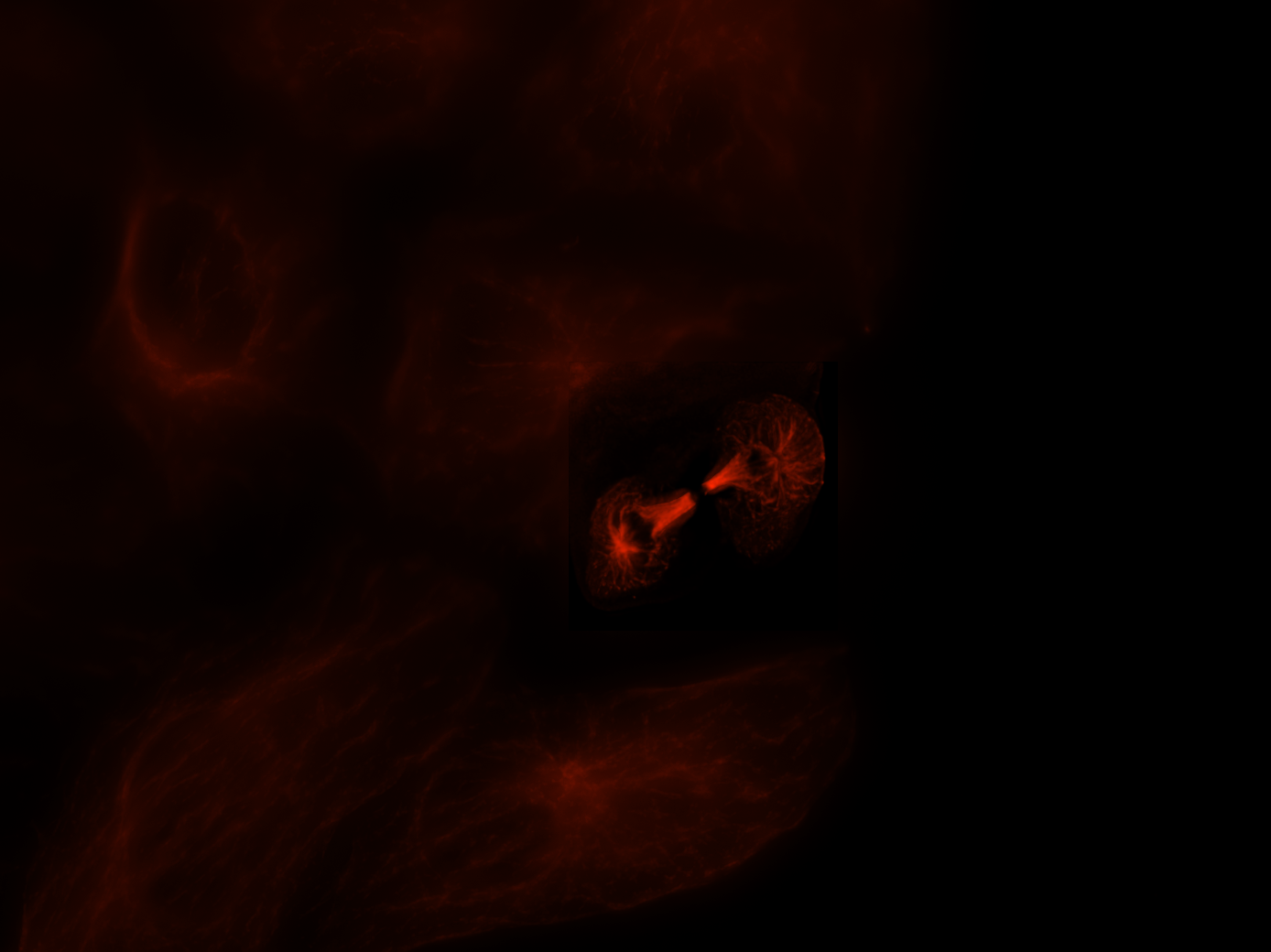

Supplement: Supplementary file 9 — Figure EV2 Source Data [file 44319_2026_833_MOESM9_ESM.zip › EV2B/Repo-Man/EMBO_GFP-Repo-Man_Telophase_03_dec-Orthogonal Projection-08/EMBO_GFP-Repo-Man_Telophase_03_dec-Orthogonal Projection-08_c3.tif]

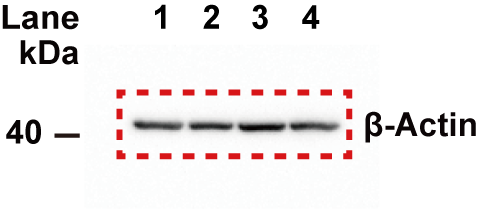

Supplement: Supplementary file 10 — Figure EV3 Source Data [file 44319_2026_833_MOESM10_ESM.zip › EV3A/Actin/Origin EV3A Actin.tif]

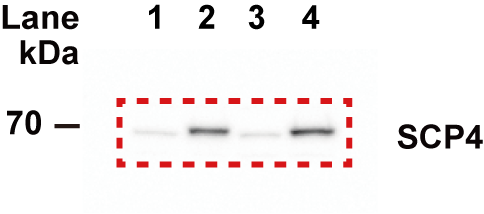

Supplement: Supplementary file 10 — Figure EV3 Source Data [file 44319_2026_833_MOESM10_ESM.zip › EV3A/SCP4/Origin EV3A SCP4.tif]

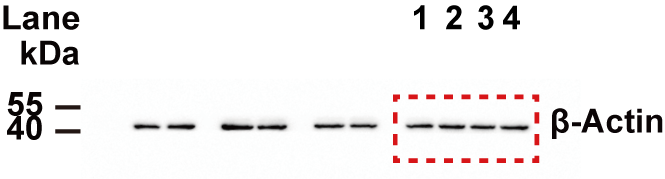

Supplement: Supplementary file 10 — Figure EV3 Source Data [file 44319_2026_833_MOESM10_ESM.zip › EV3B/Actin/Origin EV3B Actin.tif]

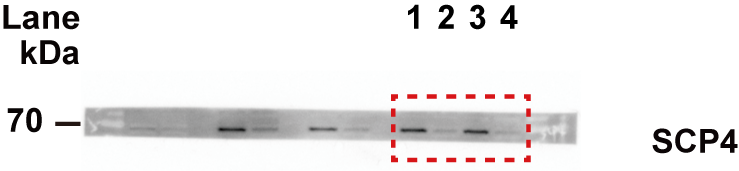

Supplement: Supplementary file 10 — Figure EV3 Source Data [file 44319_2026_833_MOESM10_ESM.zip › EV3B/SCP4/Origin EV3B SCP4.tif]

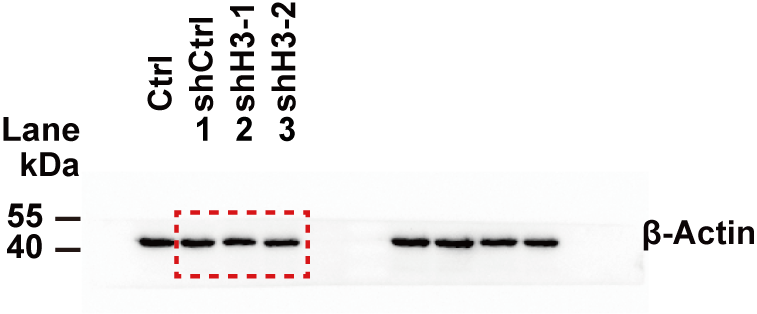

Supplement: Supplementary file 11 — Figure EV4 Source Data [file 44319_2026_833_MOESM11_ESM.zip › EV4A/Actin/Origin EV4A Actin.tif]

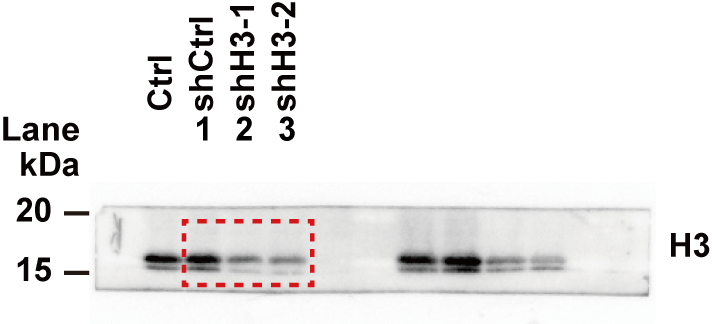

Supplement: Supplementary file 11 — Figure EV4 Source Data [file 44319_2026_833_MOESM11_ESM.zip › EV4A/H3/Origin EV4A H3.tif]

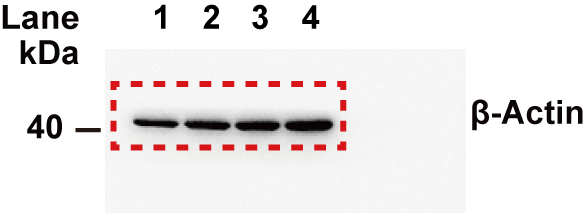

Supplement: Supplementary file 11 — Figure EV4 Source Data [file 44319_2026_833_MOESM11_ESM.zip › EV4B/Actin/Origin EV4B Actin.tif]

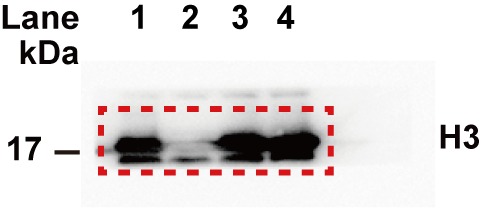

Supplement: Supplementary file 11 — Figure EV4 Source Data [file 44319_2026_833_MOESM11_ESM.zip › EV4B/H3/Origin EV4B H3.tif]

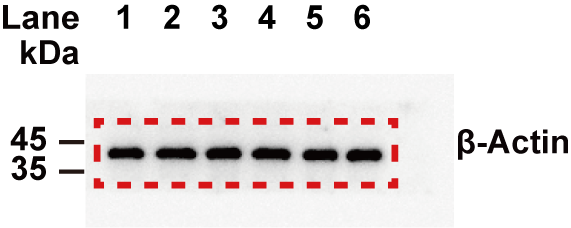

Supplement: Supplementary file 11 — Figure EV4 Source Data [file 44319_2026_833_MOESM11_ESM.zip › EV4C/Actin/Origin EV4C Actin.tif]

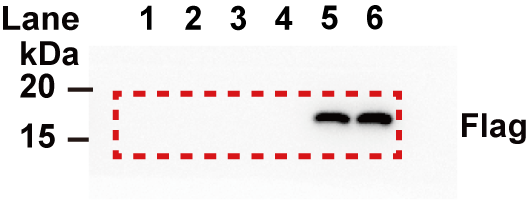

Supplement: Supplementary file 11 — Figure EV4 Source Data [file 44319_2026_833_MOESM11_ESM.zip › EV4C/FLAG/Origin EV4C Flag.tif]

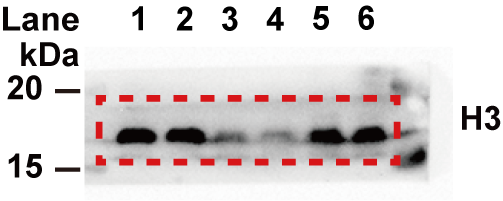

Supplement: Supplementary file 11 — Figure EV4 Source Data [file 44319_2026_833_MOESM11_ESM.zip › EV4C/H3/Origin EV4C H3.tif]

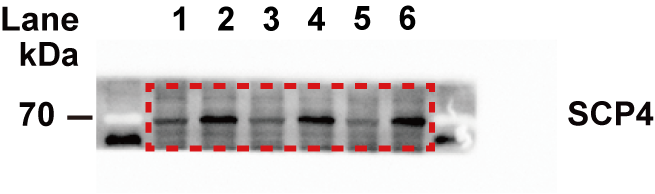

Supplement: Supplementary file 11 — Figure EV4 Source Data [file 44319_2026_833_MOESM11_ESM.zip › EV4C/SCP4/Origin EV4C SCP4.tif]

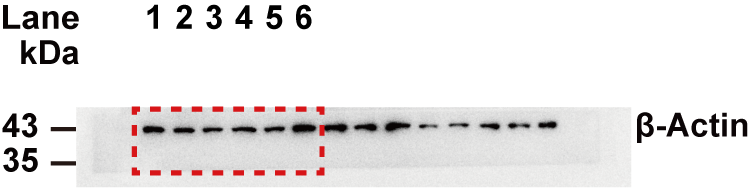

Supplement: Supplementary file 11 — Figure EV4 Source Data [file 44319_2026_833_MOESM11_ESM.zip › EV4D/Actin/Origin EV4DActin.tif]

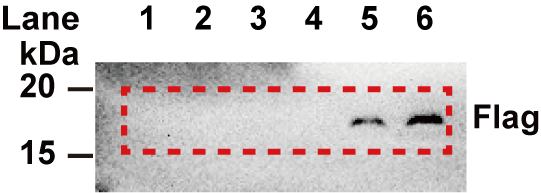

Supplement: Supplementary file 11 — Figure EV4 Source Data [file 44319_2026_833_MOESM11_ESM.zip › EV4D/FLAG/Origin EV4D Flag.tif]

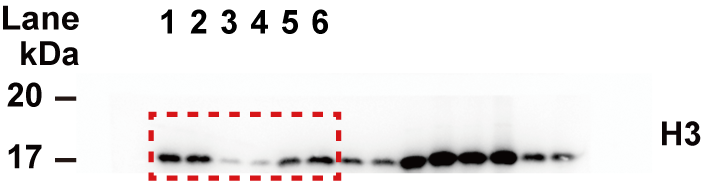

Supplement: Supplementary file 11 — Figure EV4 Source Data [file 44319_2026_833_MOESM11_ESM.zip › EV4D/H3/Origin EV4D H3.tif]

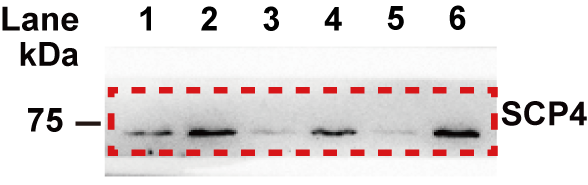

Supplement: Supplementary file 11 — Figure EV4 Source Data [file 44319_2026_833_MOESM11_ESM.zip › EV4D/SCP4/Origin EV4D SCP4.tif]

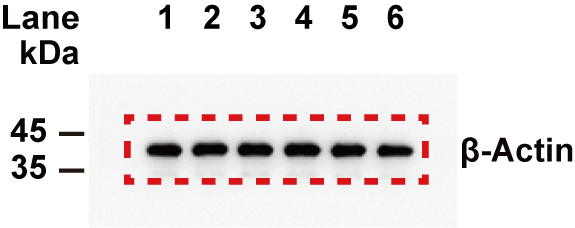

Supplement: Supplementary file 11 — Figure EV4 Source Data [file 44319_2026_833_MOESM11_ESM.zip › EV4E/Actin/Origin EV4E Actin.tif]

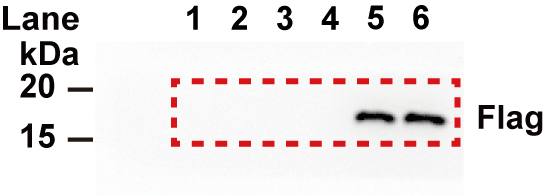

Supplement: Supplementary file 11 — Figure EV4 Source Data [file 44319_2026_833_MOESM11_ESM.zip › EV4E/FLAG/Origin EV4E Flag.tif]

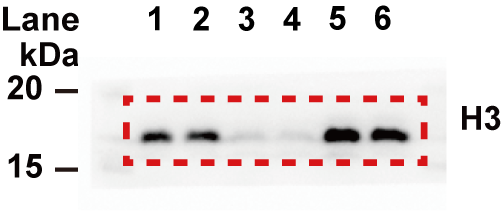

Supplement: Supplementary file 11 — Figure EV4 Source Data [file 44319_2026_833_MOESM11_ESM.zip › EV4E/H3/Origin EV4E H3.tif]

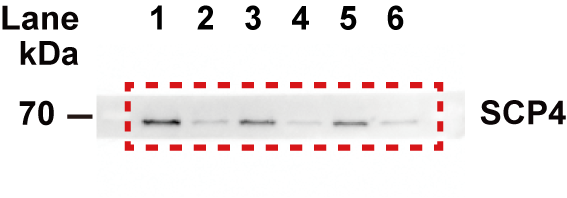

Supplement: Supplementary file 11 — Figure EV4 Source Data [file 44319_2026_833_MOESM11_ESM.zip › EV4E/SCP4/Origin EV4E SCP4.tif]

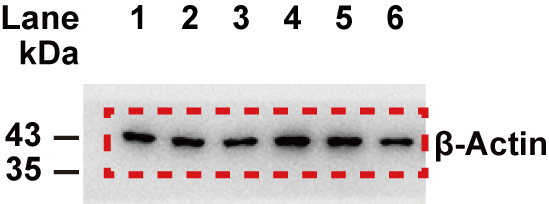

Supplement: Supplementary file 11 — Figure EV4 Source Data [file 44319_2026_833_MOESM11_ESM.zip › EV4F/Actin/Origin EV4F Actin.tif]

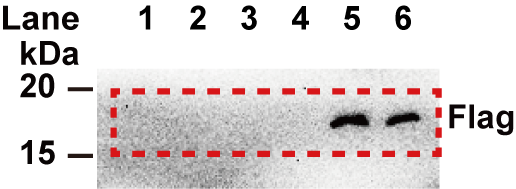

Supplement: Supplementary file 11 — Figure EV4 Source Data [file 44319_2026_833_MOESM11_ESM.zip › EV4F/FLAG/Origin EV4F Flag.tif]

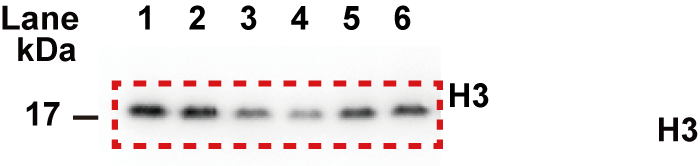

Supplement: Supplementary file 11 — Figure EV4 Source Data [file 44319_2026_833_MOESM11_ESM.zip › EV4F/H3/Origin EV4F H3.tif]

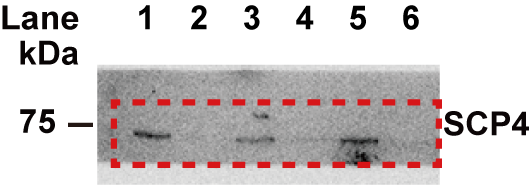

Supplement: Supplementary file 11 — Figure EV4 Source Data [file 44319_2026_833_MOESM11_ESM.zip › EV4F/SCP4/Origin EV4F SCP4.tif]
