# Supplementary material for: SCP4 dephosphorylates mitotic histone H3 to maintain chromosome stability
Source: EMBO Rep. 2026 Jun 19;27(14):3944–63. doi: 10.1038/s44319-026-00833-1 (PMC13400628; doi:10.1038/s44319-026-00833-1)
Supplement: Supplementary file 12 — Expanded View Figures [file 44319_2026_833_MOESM12_ESM.pdf]

Expanded View Figures

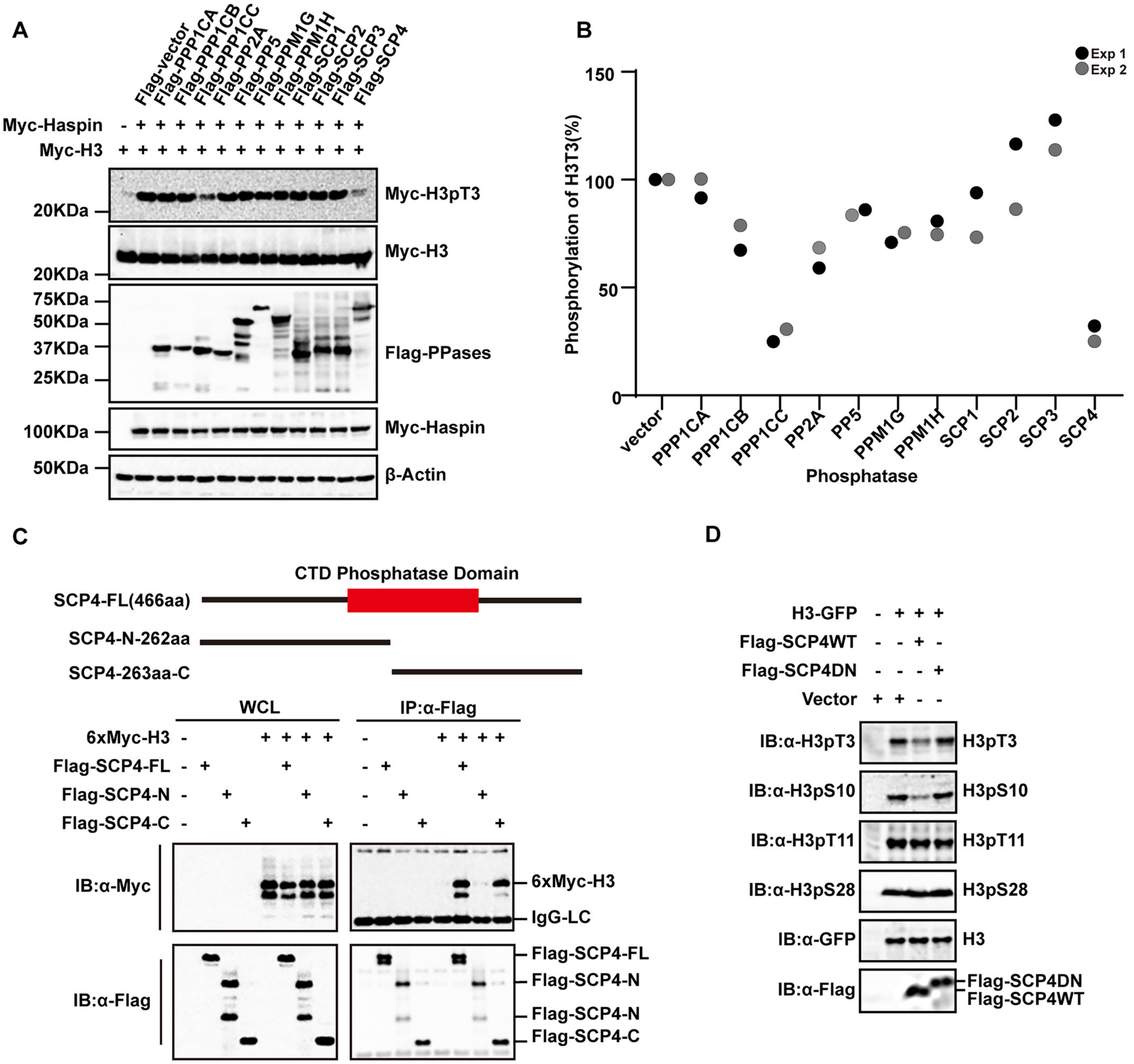

Figure EV1. SCP4 reduced the phosphorylation level of H3 at the Thr3 site.

(**A**) A representative panel of serine/threonine phosphatase library was screened to identify phosphatase(s) that reduce H3T3 phosphorylation, as assessed using phospho-T3- specific antibody. (**B**) Quantification of the ability of serine/threonine phosphatase family members to reduce H3T3 phosphorylation levels. The relative density ratio of H3pT3 to Myc-H3 levels was quantified using ImageJ (Version 1.54p). H3pT3 dephosphorylation results were obtained from two independent phosphatase screen assays ($n = 2$) and are presented as individual data points. (**C**) The C-terminal domain of SCP4 binds to H3. (**D**) SCP4 reduced the phosphorylation levels of T3 and S10, but not T11 and S28, in H3 in HEK293T cells. Source data are available online for this figure.

 © The Author(s)

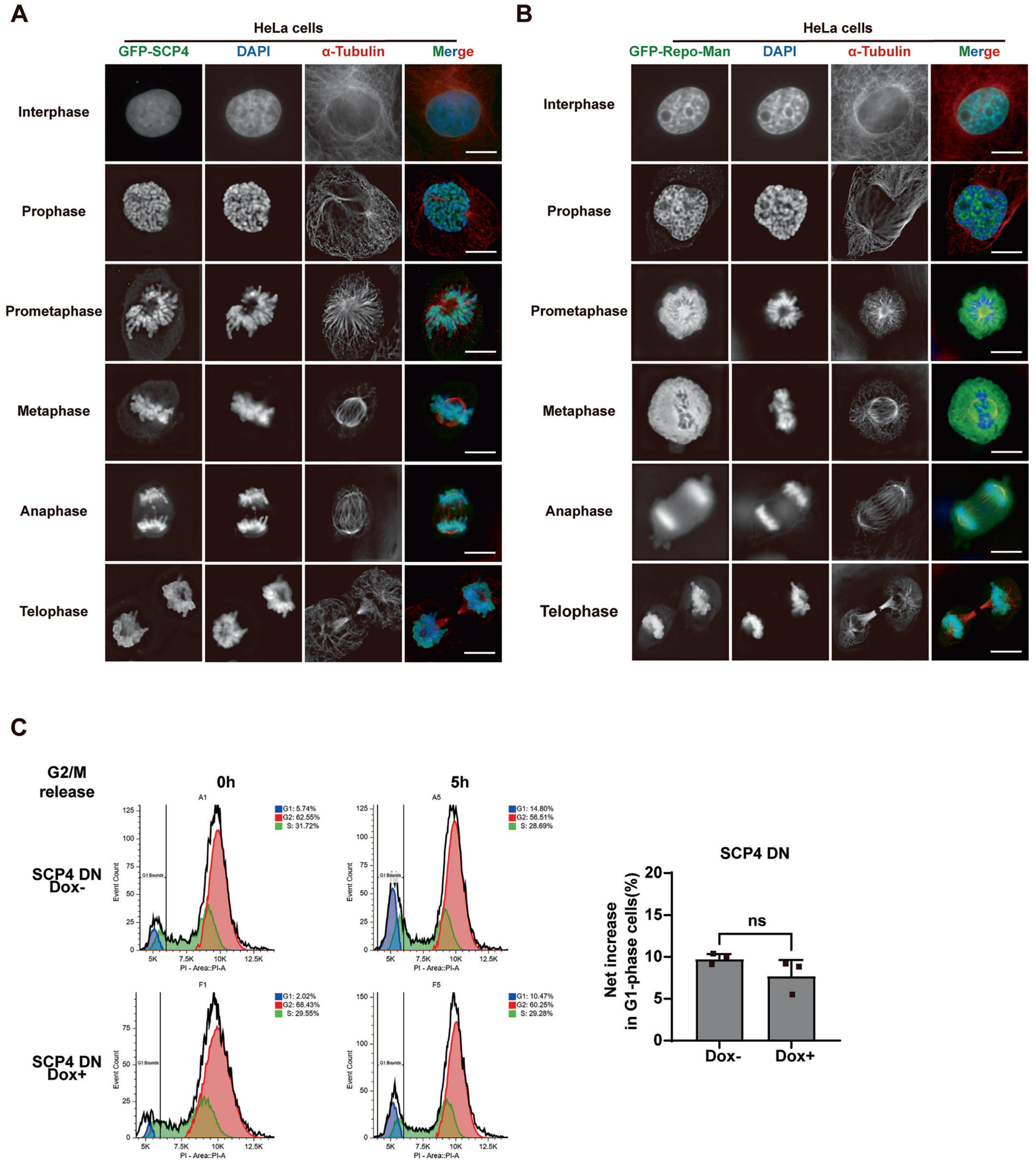

Figure EV2. Distinct chromosomal localization patterns of SCP4 and Repo-Man during mitosis.

(**A**, **B**) The chromosomal localization of GFP-SCP4 (**A**) and GFP-Repo-Man (**B**) in interphase, prophase, prometaphase, metaphase, anaphase, and telophase was determined by IF staining of HeLa cells. SCP4 remains associated with chromosomes throughout mitosis, while Repo-Man localizes to the nucleus in interphase, disperses from prophase to metaphase, and recruits to chromosomes at the onset of anaphase. Scale bars: 10 μm. (**C**) FACS analysis of cell cycle distribution showed that SCP4-DN expression had no significant effect on the progression from G2/M into G1 phase in HeLa cells after release from nocodazole-induced synchronization. The FACS data were analyzed using the Floreada.io software (version 3/30/2025) and presented as the mean percentage ± SD. The net increase in the percentage of G1 phase cells was calculated from three independent rounds, and statistical analysis was performed by using an unpaired, two-tailed Student's *t*-test. ns, not significant ($P = 0.1592$). Source data are available online for this figure.

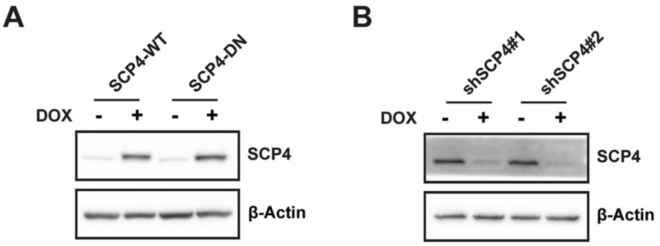

Figure EV3. Generation of HCT116 and HeLa stable cell lines with SCP4 overexpression or knockdown, as well as H3T3 mutant.

(**A**, **B**) Dox-inducible SCP4 overexpression (**A**) or knockdown by shSCP4 (**B**) was confirmed by WB. β-Actin: internal control for WB. Source data are available online for this figure.

 © The Author(s)

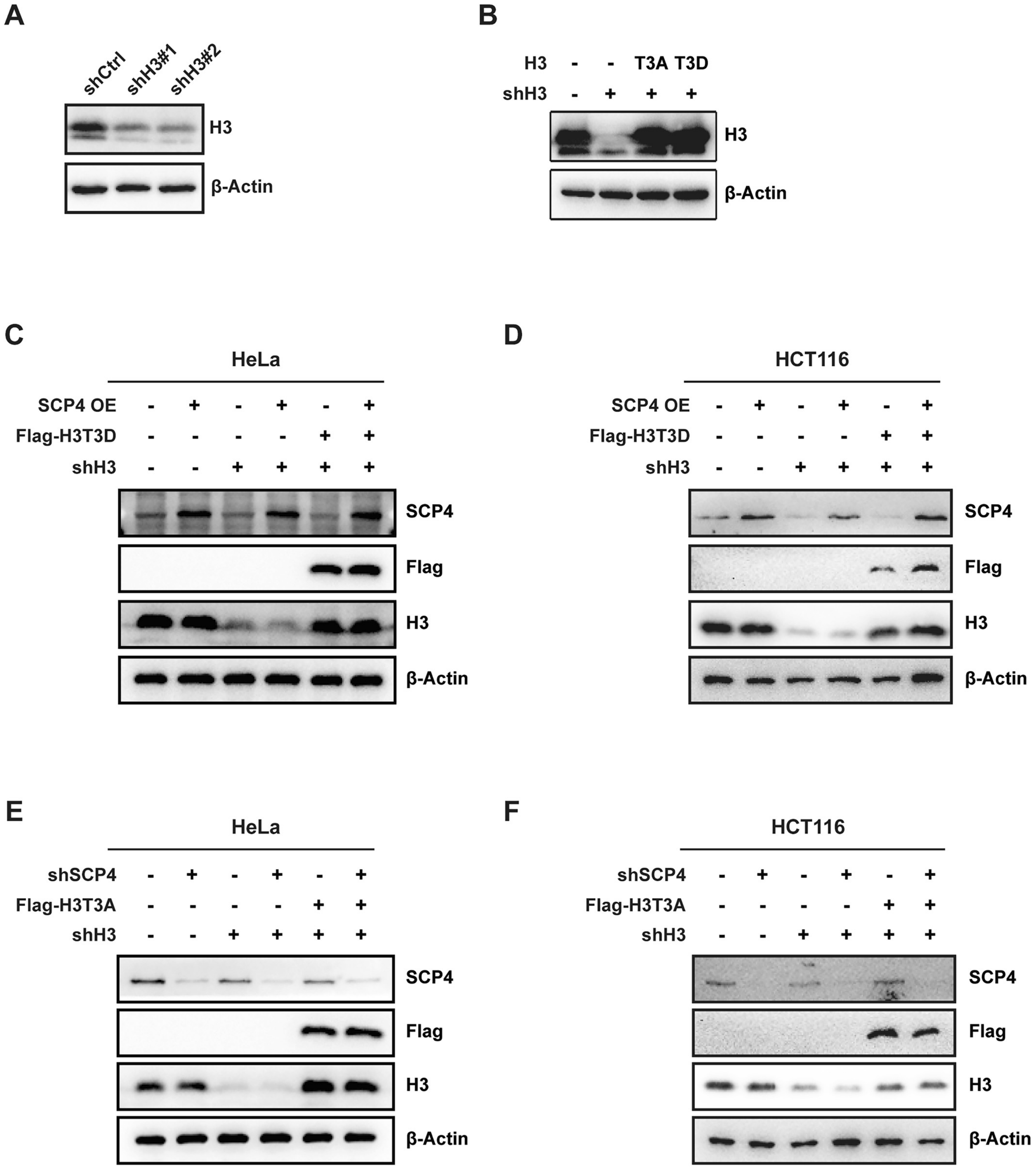

Figure EV4. Stable HeLa and HCT116 cell lines were generated to express either a phospho-mimetic H3 mutant (H3T3D or Thr3Asp) together with SCP4 or a non-phosphorylatable H3 mutant (H3T3A or Thr3Ala) together with shSCP4, while endogenous H3 was simultaneously knocked down using shH3.

(**A**) The knockdown efficiency of shH3#1 and shH3#2 was confirmed by WB in HeLa cells. (**B**) Expression of shH3-resistant H3T3D and H3T3A mutants was confirmed by WB in HeLa cells. (**C**, **D**) SCP4-overexpressing cells were established in H3T3D-expressing HeLa (**C**) and HCT116 cells (**D**). (**E**, **F**) SCP4-knockdown cells were established in H3T3A-expressing HeLa (**E**) and HCT116 cells (**F**). β-Actin: internal control for WB. Source data are available online for this figure.

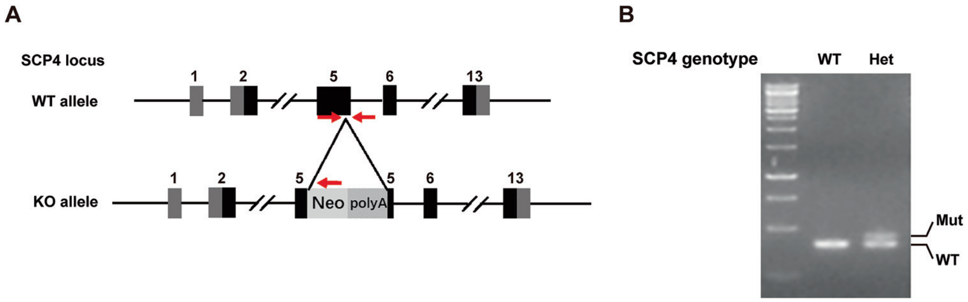

Figure EV5. Generation of Scp4 gene knockout mice.

(**A**) Diagram illustrating the strategy used to generate Scp4 knockout mice. Red arrows indicate the position of primers used for genotyping. (**B**) Genotyping of Scp4 knockout mice by PCR. Het heterozygous mouse, Mut Scp4 mutant allele.

 © The Author(s)
