# Supplementary material for: SCP4 dephosphorylates mitotic histone H3 to maintain chromosome stability
Source: EMBO Rep. 2026 Jun 19;27(14):3944–63. doi: 10.1038/s44319-026-00833-1 (PMC13400628; doi:10.1038/s44319-026-00833-1)
Supplement: Supplementary file 2 — Peer Review File [file 44319_2026_833_MOESM2_ESM.pdf]

EMBO reports

SCP4 dephosphorylates mitotic histone H3 to maintain chromosome stability

Yupiao Zheng, Zhengmao Zhang, Qi Wang, Jin Cao, Yang Li, Tingbo Liang, Xin-Hua Feng, and Xia Lin

Corresponding author(s): Xia Lin (xialinzheda@zju.edu.cn) , Xin-Hua Feng (fenglab@zju.edu.cn), Tingbo Liang (liangtingbo@zju.edu.cn)

Review Timeline:

Submission Date:	28th Aug 25
Editorial Decision:	6th Nov 25
Revision Received:	3rd Feb 26
Editorial Decision:	27th Mar 26
Revision Received:	15th Apr 26
Accepted:	29th May 26

Editor: Kurt Weir

Transaction Report:

(Note: With the exception of the correction of typographical or spelling errors that could be a source of ambiguity, letters and reports are not edited. Depending on transfer agreements, referee reports obtained elsewhere may or may not be included in this compilation. Referee reports are anonymous unless the Referee chooses to sign their reports.)

Dear Dr. Lin

Thank you for the submission of your research manuscript to our journal. We have now received the full set of referee reports that is copied below.

As you will see, the referees acknowledge that the findings are interesting and that the conclusions are overall supported by the data presented but they also raise a number of concerns and have suggestions how to further strengthen the data, including a greater characterization of the in vivo phenotype and additional experiments or controls to confirm the specificity of the interaction between SCP4 and H3T3.

Given these constructive comments, we would like to invite you to revise your manuscript with the understanding that the referee concerns (as detailed above and in their reports) must be fully addressed and their suggestions taken on board. Please address all referee concerns in a complete point-by-point response. Acceptance of the manuscript will depend on a positive outcome of a second round of review. It is EMBO Reports policy to allow a single round of revision only and acceptance or rejection of the manuscript will therefore depend on the completeness of your responses included in the next, final version of the manuscript.

We realize that it is difficult to revise to a specific deadline. In the interest of protecting the conceptual advance provided by the work, we recommend a revision within 3 months (February 6th). Please discuss the revision progress ahead of this time with the editor if you require more time to complete the revisions.

I am also happy to discuss the revision further via e-mail or a video call, if you wish.

You can either publish the study as a short report or as a full article. For short reports, the revised manuscript should not exceed 27,000 characters (including spaces but excluding materials & methods and references) and 5 main plus 5 expanded view figures. The results and discussion sections must further be combined, which will help to shorten the manuscript text by eliminating some redundancy that is inevitable when discussing the same experiments twice. For a normal article there are no length limitations, but it should have more than 5 main figures and the results and discussion sections must be separate. In both cases, the entire materials and methods must be included in the main manuscript file.

================
IMPORTANT NOTE:

We perform an initial quality control of all revised manuscripts before re-review. Your manuscript will FAIL this control and the handling will be delayed IN CASE the following APPLIES:

1) A data availability section providing access to data deposited in public databases is missing. If you have not deposited any data, please add a sentence to the data availability section that explains that.

2) Your manuscript contains statistics and error bars based on n=2. Please use scatter blots in these cases. No statistics should be calculated if n=2.

When submitting your revised manuscript, please carefully review the instructions that follow below. Failure to include requested items will delay the evaluation of your revision.

================

When submitting your revised manuscript, we will require:

1) a .docx formatted version of the manuscript text (including legends for main figures, EV figures and tables). Please make sure that the changes are highlighted to be clearly visible.

2) individual production quality figure files as .eps, .tif, .jpg (one file per figure).
Please download our Figure Preparation Guidelines (figure preparation pdf) from our Author Guidelines pages https://www.embopress.org/page/journal/14693178/authorguide for more info on how to prepare your figures.

3) a .docx formatted letter INCLUDING the reviewers' reports and your detailed point-by-point responses to their comments. As part of the EMBO Press transparent editorial process, the point-by-point response is part of the Review Process File (RPF), which will be published alongside your paper.

4) a complete author checklist, which you can download from our author guidelines (). Please insert information in the checklist

that is also reflected in the manuscript. The completed author checklist will also be part of the RPF.

5) Please note that all corresponding authors are required to supply an ORCID ID for their name upon submission of a revised manuscript (). Please find instructions on how to link your ORCID ID to your account in our manuscript tracking system in our Author guidelines
()

6) We replaced Supplementary Information with Expanded View (EV) Figures and Tables that are collapsible/expandable online. A maximum of 5 EV Figures can be typeset. EV Figures should be cited as 'Figure EV1, Figure EV2" etc... in the text and their respective legends should be included in the main text after the legends of regular figures.

- For the figures that you do NOT wish to display as Expanded View figures, they should be bundled together with their legends in a single PDF file called *Appendix*, which should start with a short Table of Content. Appendix figures should be referred to in the main text as: "Appendix Figure S1, Appendix Figure S2" etc. See detailed instructions regarding expanded view here:

- Additional Tables/Datasets should be labeled and referred to as Table EV1, Dataset EV1, etc. Legends have to be provided in a separate tab in case of .xls files. Alternatively, the legend can be supplied as a separate text file (README) and zipped together with the Table/Dataset file.

7) Please note that a Data Availability section at the end of Materials and Methods is now mandatory. In case you have no data that requires deposition in a public database, please state so instead of refereeing to the database.
See also < https://www.embopress.org/page/journal/14693178/authorguide#dataavailability>). Please note that the Data Availability Section is restricted to new primary data that are part of this study.

8) At EMBO Press we ask authors to provide source data for the main manuscript figures. You will receive a separate email with instructions for providing source data with your revised manuscript, including how to upload and organize the files.

Additional information on source data and instruction on how to label the files are available

9) The journal requires a statement specifying whether or not authors have competing interests (defined as all potential or actual interests that could be perceived to influence the presentation or interpretation of an article). In case of competing interests, this must be specified in your disclosure statement. Further information: https://www.embopress.org/competing-interests

10) Figure legends and data quantification:
The following points must be specified in each figure legend:

- the name of the statistical test used to generate error bars and P values,
- the EXACT p-values,
- the number (n) of independent experiments (please specify technical or biological replicates) underlying each data point,
- the nature of the bars and error bars (s.d., s.e.m.)

- If the data are obtained from n {less than or equal to} 5, show the individual data points in addition to the SD or SEM.
- If the data are obtained from n {less than or equal to} 2, use scatter blots showing the individual data points.

Discussion of statistical methodology can be reported in the materials and methods section, but figure legends should contain a basic description of n, P and the test applied.

See also the guidelines for figure legend preparation:
https://www.embopress.org/page/journal/14693178/authorguide#figureformat

- Please also include scale bars in all microscopy images and define their size in the legend, not the image.

11) Our journal encourages inclusion of *data citations in the reference list* to directly cite datasets that were re-used and obtained from public databases. Data citations in the article text are distinct from normal bibliographical citations and should directly link to the database records from which the data can be accessed. In the main text, data citations are formatted as follows: "Data ref: Smith et al, 2001" or "Data ref: NCBI Sequence Read Archive PRJNA342805, 2017". In the Reference list, data citations must be labeled with "[DATASET]". A data reference must provide the database name, accession number/identifiers and a resolvable link to the landing page from which the data can be accessed at the end of the reference. Further instructions are available at .

12) All Materials and Methods need to be described in the main text using our 'Structured Methods' format. According to this format, the Methods section includes a Reagents and Tools Table (listing key reagents, experimental models, software and

relevant equipment and including their sources and relevant identifiers) followed by a Methods and Protocols section describing the methods, ideally using a step-by-step protocol format. The aim is to facilitate adoption of the methodologies across labs. Please download and fill our Reagents and Tools Table template (.docx), which you can find in our author guidelines: https://www.embopress.org/page/journal/14693178/authorguide#structuredmethods.
When submitting your revised manuscript, please do not include the Reagents and Tools Table in the Methods section of the manuscript but upload it as a separate file choosing the file type "Reagent Table".
An example of a Method paper with Structured Methods can be found here: https://www.embopress.org/doi/10.15252/msb.20178071.

13) As part of the EMBO publication's Transparent Editorial Process, EMBO Reports publishes online a Review Process File to accompany accepted manuscripts. This File will be published in conjunction with your paper and will include the referee reports, your point-by-point response and all pertinent correspondence relating to the manuscript.

You are able to opt out of this by letting the editorial office know (emboreports@embo.org). If you do opt out, the Review Process File link will point to the following statement: "No Review Process File is available with this article, as the authors have chosen not to make the review process public in this case."

We would also welcome the submission of cover suggestions, or motifs to be used by our Graphics Illustrator in designing a cover.

I look forward to seeing a revised form of your manuscript when it is ready and please let me know if you have questions or comments regarding the revision.

Yours sincerely,

Kurt Weir
Editor
EMBO Reports

Referee #1:

Zheng Y, Zhang Z, Wang Q and Cao J report in this paper that SCP4 can act as a phosphatase for histone H3 Threonine 3 (T3) in vitro (biochemically) and in cells. SCP4 is a nuclear protein and interacts with histone H3 on chromatin. Interestingly active SCP4 is able to interact with presumably more affinity to histone H3 than its catalytically defective version (SCP4 dominant negative mutant). The paper also shows that aberrant expression of SCP4 (achieved by overexpression or shRNA-mediated knock-down) disturbs the recruitment of the CPC components survivin and Aurora B, leading to abnormal chromosome dynamics during the cell cycle causing aneuploidy. Further, it is shown (using phosphomimetic and non-phosphorylatable proteoforms of histone H3) that a dynamic balance of histone H3 (T3) phosphorylation is required for cell cycle progression and chromosomal stability. Finally, using a gene KO mice model of SCP4, the authors report mitotic abnormalities during the first cleavage at the 2-cell zygote stage.

The paper is well executed, well written and technically sound. My only concern is about the novelty of the study per se. The importance of this phosphorylation event on histone H3 is well documented. There have been other phosphatases already linked to this phospho-mark (such as PP1-Repo-Man) and other substrates for SCP4 have previously been described too. So, in my mind, even if this is a solid paper, it perhaps lacks enough novelty to deserve consideration for publication in EMBO Reports but this last decision is in the remit of the editor. Below some specific comments I can make:

Major comments:

(1) What are the effects of the overexpression of the DN version of SCP4 and the shRNAs for SCP4 in cells in terms of general cell cycle distribution by flow cytometry?

(2) Figure 1D shows very interesting data regarding the selectivity of SCP4 for histone H3 vis-à-vis other members of the SCP family. SCP1-3 are smaller proteins compared to SCP4, the shift in that blot looks quite big, I was expecting a 20 kDa shift or so, can the authors include MW markers in this figure. But more importantly can they work out a bit the molecular determinants present in SCP4 responsible for this selective control of histone H3?

(3) I am somehow surprised of the high percentages of abnormal cell cycle events quantified in the WT (not o/e SCP4) HeLa cells. There is also a bit of variability in the data shown (20% in Figure 4D but closer to 40% in 4B). I know these cells are

aberrant but maybe can other papers with similar levels found by the authors be cited to indicate that this is indeed the case.

(4) The data on the SCP4 KO mice is interesting but somehow preliminary. Would they say this is an essential gene for embryonic development? Can other markers perhaps be checked such as survivin and Aurora B that would tie with the rest of the paper? Are there other potential substrates of SCP4 that could be involved in the effects they see (some of these other potential substrates have been reported by the authors in previous publications)? Can maybe some (the key one(s)) be checked or at least discussed as other possible targets explaining the phenotype observed?

Minor comments:

(1) Figure 1A: can a marker for noco release can be included? This is perhaps important when MG and CQ are included that may affect cell cycle release.

(2) Figure 1F: please quantify the H3pT3 and H3 signals (ratio).

(3) Figure 2B: as above, please quantify. This is an interesting piece of data suggesting that active SCP4 interacts better with histone H3 compared to an inactive SCP4 version. Can the authors speculate or briefly discuss the potential reasons?

Referee #2:

Summary:

The mitosis-specific phosphorylation of histone H3 at threonine 3 (H3T3ph) is a key post-translational modification that functions as a signaling hub to recruit essential protein complexes, including the chromosomal passenger complex (CPC), thereby ensuring faithful chromosome segregation. Previous studies have established that the PP1/Repo-Man phosphatase complex dephosphorylates H3T3ph toward the end of mitosis. However, because Haspin kinase activity is not strictly limited to mitosis, it has been assumed that additional phosphatase(s) counteract Haspin activity to maintain H3T3ph homeostasis.
In this manuscript, Zheng, Zhang, Wang, Cao et al. identify SCP4 as a phosphatase that regulates H3T3ph levels in mitosis through a phosphatase library screen. Using both cell biological and biochemical approaches, they provide evidence to support their conclusion that SCP4 functions as a histone H3 phosphatase, as indicated in the title of the paper.

Their key findings are:
1. SCP4 is identified as a novel phosphatase for histone H3 at threonine 3 (H3T3) during mitosis.
2. SCP4 dephosphorylates H3T3 both in vitro and in vivo.
2. Altering SCP4 expression disrupts CPC localization, leading to chromosome instability.
3. SCP4 is important for early developmental viability.

Overall, the evidence supporting SCP4's ability to dephosphorylate H3T3 in vitro-and some of the in vivo data-are compelling. However, there remain concerns regarding (1) the extent to which SCP4 functions as an H3T3 phosphatase in vivo. (2) less investigation of the effect of SCP4 function on other histone H3 phosphorylation marks. (3) There is limited investigation of the relationship between SCP4 and the well-established Repo-Man/PP1 pathway for histone H3T3 dephosphorylation, an issue that has important implications for the mitotic chromosome field. These concerns should be thoroughly addressed before publication.

Major comments:

1. The authors identified SCP4 through a phosphatase library screen, and the abstract states that this screening was performed systematically. However, the manuscript does not provide a clear explanation of how the screen was conducted leading to the identification of SCP4, even in the Materials and Methods section. This information is critical for readers, and the authors should include either the original data or a summary table listing the candidates used in the screen along with the corresponding results.
2. In this manuscript, the authors test the phosphatase activity of SCP4 using H3T3ph as the primary readout. However, as noted above, they do not provide details on how phosphatase activity against histone H3 phosphorylation was screened. It remains possible that SCP4 also dephosphorylates other histone H3 phosphorylation sites, such as H3S10 or H3T11, as previously demonstrated for Repo-Man/PP1. The authors should demonstrate the specificity of SCP4 toward H3T3ph relative to other histone H3 phosphorylation marks, and clarify how SCP4 functions as a histone H3 phosphatase during mitosis. This point is particularly important for their proposed model in Figure 5, which states that "Aberrant H3T3 phosphorylation leads to mitotic defects and increased chromosomal instability".
3. On page 4, the authors state that "this phenotype distinguishes SCP4 from Repo-Man/PP1γ, which regulates H3T3 dephosphorylation late in mitosis." However, several of the cited studies report (for example, supplemental data in PMID: 21514157) that PP1 knockdown produces a similar retention of H3T3ph levels in mitosis, comparable to what is shown in Figure 1D of this manuscript. To strengthen their claim, it would be important to assess the effects of altering the Repo-Man/PP1

pathway directly, especially given the differences in cell type and experimental system compared with the referenced studies. Additionally, it is recommended that the authors provide a quantification of the Western blot signal over the time course, normalized to total H3. Ideally, this analysis should also include other histone H3 phosphorylation marks, as noted above.
4. In Figure 1E and 1F, the authors performed in vitro kinase assays using recombinant proteins, with Western blot as the primary readout. However, in Figure 1E, H3T3ph is detected even in the absence of Haspin kinase treatment. In Figure 1F, the reduction of H3T3ph signal upon SCP4-WT treatment is very subtle. To strengthen the conclusions, it is recommended that the authors employ alternative readouts, such as 32P-ATP incorporation or phosphoprotein-specific staining. In addition, including H3T3A and/or H3T3D mutants as negative controls would help demonstrate the specificity of SCP4 activity toward H3T3.

Minor comments:
1. It would be helpful to enlarge the fluorescence images to improve visualization, even in the digital format.
2. It is recommended to show representative fluorescence images for Figure 5A-C in either main or supplemental figures.
3. Please indicate not only the number of cells analyzed but also the number of independent experiments performed.
4. Related to the above point, please briefly describe the statistical method used in each figure legend. Summarizing the methods only in the Materials and Methods section does not make it clear which statistical test was applied to each figure.
5. Please include the antibody dilution ratios in the Materials and Methods section.
6. Please include the catalog numbers for reagents used in this study
7. Please include the uncropped Western Blot membrane raw data in supplemental figures
8. In Page#3, typo "Repo-man/PP1r" -> "Repo-Man/PP1γ"

Referee #3:

How do the released cells look like?

Transient Histone H3 phosphorylation by Haspin plays an important role in mitotic regulation. In this thorough study Zheng at al propose that SCP4 dephosphorylates H3. On the whole the experiments presented in the manuscript strongly support this hypothesis.
A few minor remarks

Fig 1. A
Unless mitotic shake off is used nocodazole arrest and release rarely leads to very synchronous G1 cell populations. It is interesting that MG132 treatment, which you would expect arresting cells in metaphase does not eliminate H3 dephosphorylation. Maybe SCP4 dephosphorylates H3 prior to this stage. This could be briefly discussed.

Fig 3.
The results of the IF are indeed very nice but in the way they are represented are anecdotal. It would be useful to add some quantitative data (counting the number of cells with the different patterns).

Manuscript EMBOR-2025-62636V2

We appreciate the reviewers for their constructive and insightful comments. We have carefully addressed all concerns by incorporating new data, additional figures and tables, and detailed descriptions of the statistical analyses. The revised manuscript has been updated accordingly, with all changes highlighted in blue in the main text, Materials and Methods section, and figure legends. Below is our point-by-point response to the reviewers' comments:

Reviewer #1

Major comments:

(1) *Zheng Y, Zhang Z, Wang Q and Cao J report in this paper that SCP4 can act as a phosphatase for histone H3 Threonine 3 (T3) in vitro (biochemically) and in cells. SCP4 is a nuclear protein and interacts with histone H3 on chromatin. Interestingly active SCP4 is able to interact with presumably more affinity to histone H3 than its catalytically defective version (SCP4 dominant negative mutant). The paper also shows that aberrant expression of SCP4 (achieved by overexpression or shRNA-mediated knock-down) disturbs the recruitment of the CPC components survivin and Aurora B, leading to abnormal chromosome dynamics during the cell cycle causing aneuploidy. Further, it is shown (using phosphomimetic and non-phosphorylatable proteoforms of histone H3) that a dynamic balance of histone H3 (T3) phosphorylation is required for cell cycle progression and chromosomal stability. Finally, using a gene KO mice model of SCP4, the authors report mitotic abnormalities during the first cleavage at the 2-cell zygote stage.*

The paper is well executed, well written and technically sound. My only concern is about the novelty of the study per se. The importance of this phosphorylation event on histone H3 is well documented. There have been other phosphatases already linked to this phospho-mark (such as PP1-Repo-Man) and other substrates for SCP4 have previously been described too. So, in my mind, even if this is a solid paper, it perhaps lacks enough novelty to deserve consideration for publication in EMBO Reports but this last decision is in the remit of the editor.

Response: Thanks to the reviewer for her/his praise of our solid study. However, she/he has expressed concerns about the novelty of the study. I would like to address the concern as follows:

First, SCP4 and Repo-Man/PP1γ regulate H3 phosphorylation through different mechanisms. During prometaphase and metaphase, Repo-Man localization, its interaction with PP1γ, and PP1γ enzymatic activity are tightly modulated by mitotic kinases and regulatory proteins. Repo-Man/PP1γ activity is suppressed during early mitosis through multiple mechanisms such as Cyclin B/CDK1-mediated phosphorylation, which either directly decreases the PP1γ activity (PMID: 22555598,16492807, 16998479, 21820363) or phosphorylation of Inhibitor-1/2 proteins (PMID: 14715909, 12697755) that inhibits PP1γ activity. Since the Repo-Man/PP1γ activity is suppressed during prometaphase/metaphase, identifying a direct chromosome-associated phosphatase responsible for H3 dephosphorylation at this

stage is important. In this study, we demonstrate that SCP4 directly associates with chromosomes and dephosphorylates H3 during prometaphase/metaphase, exhibiting a chromatin-binding pattern distinct from that of Repo-Man. These data are presented in the new figures (Figure EV2A and EV2B).

Second, the fact that other substrates for SCP4 have been previously described should not diminish the significance of studying this specific protein. Our study can provide insights into novel regulatory mechanisms or biological roles of SCP4 in mitosis that have not yet been uncovered. Our investigation of the SCP4-mediated H3T3 dephosphorylation is crucial for elucidating its role in mitosis. The importance of our study extends beyond mere novelty; it holds the potential to significantly advance our understanding of this phosphatase in fundamental biological processes and potential implications for health and disease. Our findings also support the notion that phosphatases are highly versatile and act on a broad spectrum of substrates. While the human genome encodes 539 protein kinases (PMID:28400531), only about 190 protein phosphatases have been identified, suggesting that phosphatases generally exhibit lower substrate specificity than kinases. Indeed, SCP4 (encoded by CTDSPL2 gene) is a multifunctional phosphatase capable of dephosphorylating FoxO1/3a and Smad proteins. Its ubiquitous and high-level expression across most cell and tissue types (Tau specificity score = 0.24; PMID: 25613900) further suggests that SCP4 is indispensable for maintaining cellular fitness.

(2) *What are the effects of the overexpression of the DN version of SCP4 and the shRNAs for SCP4 in cells in terms of general cell cycle distribution by flow cytometry?*

Response: To address the reviewer's question, we assessed the effect of SCP4-DN expression and shSCP4 on cell cycle distribution using FACS analysis, with the results presented in the new figures (Figure 3F and Figure EV2C) of the manuscript. Briefly, FACS analysis showed that Dox-induced expression of phosphatase-dead mutant SCP4-DN had no significant effect on the G1 content during cell cycle progression (Figure EV2C), whereas SCP4 knockdown significantly delays cell cycle progression from G2/M into G1 phase in HeLa cells (Figure 3F). In addition, mitotic index analysis showed that the depletion of SCP4 increases the mitotic index in HCT116 cells, indicating an elevated proportion of cells in M phase following SCP4 knockdown (Figure 3E).

(3) *Figure 1D shows very interesting data regarding the selectivity of SCP4 for histone H3 vis-à-vis other members of the SCP family. SCP1-3 are smaller proteins compared to SCP4, the shift in that blot looks quite big, I was expecting a 20 kDa shift or so, can the authors include MW markers in this figure. But more importantly can they work out a bit the molecular determinants present in SCP4 responsible for this selective control of histone H3?*

Response: We thank the reviewer for his/her comment. We believe the reviewer is referring to Figure 2D. SCP4 has a much larger molecular weight than SCP1-3 (PMID: 17157258). Full-length SCP1, SCP2, and SCP3 contain 261, 283, and 266 amino acids, respectively, and migrate at approximately 37 kDa on SDS-PAGE gels. In contrast, SCP4 comprises 466 amino acids and migrate at approximately 70 kDa. To determine the domain of SCP4 responsible for binding to H3, we performed co-IP and found that the C-terminal domain of SCP4 binds to H3. The data are presented in the new figure (Figure EV1C). Please note that the SCP4 N-terminal domain showed two bands: the lower molecular weight

band corresponds to an unmodified form, while our preliminary data suggested that the upper band corresponds to a highly modified form.

(4) *I am somehow surprised of the high percentages of abnormal cell cycle events quantified in the WT (not o/e SCP4) HeLa cells. There is also a bit of variability in the data shown (20% in Figure 4D but closer to 40% in 4B). I know these cells are aberrant but maybe can other papers with similar levels found by the authors be cited to indicate that this is indeed the case.*

Response: We apologize for the inaccurate labeling which caused misunderstanding. We have corrected the Y-axis labeling in Figure 4B and 4D (statistical analysis) to “percentage of abnormal mitotic cells (%)”. The labels “-” and “+” have been corrected to “Dox-” and “Dox+”, respectively. In the legend, we specify that “At least fifty missegregation cells per condition were analyzed and quantified (Figure 4B and 4D).”

The HeLa-CCL2 cell line used in the study was selected for the inducible overexpression of SCP4 or shSCP4 for knockdown (KD) under the control of the doxycycline (Dox)-inducible promoter. The cells presented in Figure 4A and 4C are Dox-inducible HeLa cells with different passage numbers due to the selection process. As previously reported, a quarter to one-third of mitotic cells in the HeLa-Kyoto cell line exhibit missegregation with lagging or bridged chromosomes (PMID: 26423723). Although different HeLa cell lines were used (and cell lines from different laboratories, even different passages, present heterogeneity in gene copy number variation (CNV)), both HeLa-CCL2 and HeLa-Kyoto cell lines exhibit similarly extensive genomic rearrangements throughout their entire genomes (PMID: 30778230). Specifically, a study of single chromosome analysis showed that the percentage of cells with chromosome missegregation was 13.7% for chromosome 8, 17.4% for chromosome 12, and 8% for both chromosome 6 and 12 in mitotic HeLa-CCL2 cells (PMID: 16222248). Therefore, we presume that if we count collectively all chromosome missegregation events together, HeLa-CCL2 cells have a chromosome missegregation rate similar to that of HeLa-Kyoto cells, which is consistent to our findings.

(5) *The data on the SCP4 KO mice is interesting but somehow preliminary. Would they say this is an essential gene for embryonic development? Can other markers perhaps be checked such as survivin and Aurora B that would tie with the rest of the paper? Are there other potential substrates of SCP4 that could be involved in the effects they see (some of these other potential substrates have been reported by the authors in previous publications)? Can maybe some (the key one(s)) be checked or at least discussed as other possible targets explaining the phenotype observed?*

Response: We appreciate the reviewer’s feedback. We agree that the SCP4 KO data for the two-cell stage are preliminary due to technical difficulties. Nevertheless, we believe that SCP4 is an essential protein for cell fitness given its ubiquitous expression, including germline cells (PMID: 25613900, PMID: 17327269). Our current study is the first to report that SCP4 is essential for the first cleavage at the two-cell zygote stage. Although technical limitations prevent us from genotyping single zygotes, the frequency of defective zygotes is consistent with the expected Mendelian ratios, suggesting the observed defects are indeed caused by SCP4 knockout.

In Figure 5E, we presented the chromatin bridge at the telophase of the first cleavage in the two-cell zygote. We selected P-H3S10 to highlight the chromatin bridge and α-tubulin to visualize the undivided zygote. At telophase, Aurora B and Survivin are no longer associated with chromosomes, as they have migrated from the centromeres to the midbody.

SCP4 is a phosphatase of FoxO1/3a and Smad1 proteins. However, as previously reported, FoxO1/3a and Smad1 are not involved in early zygote cleavage or mitosis regulation. FoxO1 knockout results in embryonic lethality at E11 due to abnormal vascular development (PMID: 24874427), while FoxO3 knockout mice are viable and progress to adulthood (PMID: 40087279). Homozygous loss of Smad1 causes embryonic lethality around embryonic day 14.5 (PMID: 36810619). The loss of these reported SCP4 substrates in mice does not induce an embryonic lethal phenotype as severe as that observed in SCP4 knockout mice.

Minor comments:

(1) Figure 1A*: can a marker for noco release can be included? This is perhaps important when MG and CQ are included that may affect cell cycle release.*

Response: We apologize for the misunderstanding caused by the original labeling. The label in Figure 1A has been changed to "Nocodazole release: - 1h" to be more concise.

(2) Figure 1F: *please quantify the H3pT3 and H3 signals (ratio).*

Response: The new revised Figure 1F replaces the original Figure 1F. We have now quantified the ratio of H3pT3 to total H3 levels in the revised figure.

(3) Figure 2B: *as above, please quantify. This is an interesting piece of data suggesting that active SCP4 interacts better with histone H3 compared to an inactive SCP4 version. Can the authors speculate or briefly discuss the potential reasons?*

Response: Thank the reviewer for bringing up this observation. We have added the quantification data to Figure 2B. Regarding the question about the different binding affinity between SCP4-WT and SCP4-DN to H3 in Figure 2B, this binding assay was performed using proteins produced from an *in vitro* translation system. In this system, the H3 was not phosphorylated, and GST-SCP4 was not post-translationally modified. The difference in binding affinity between SCP4 and H3 may be attributed to the substitution of the negatively charged aspartic acid (Asp, D) in SCP4-WT to the uncharged asparagine (Asn, N) in SCP4-DN.

Reviewer #2

Major comments:

(1) *The authors identified SCP4 through a phosphatase library screen, and the abstract states that this screening was performed systematically. However, the manuscript does not provide a clear explanation of how the screen was conducted leading to the identification of SCP4, even in the Materials and Methods section. This information is critical for readers, and the authors should include either the original data or a summary table listing the candidates used in the screen along with the corresponding results.*

(2) *In this manuscript, the authors test the phosphatase activity of SCP4 using H3T3ph as the primary readout. However, as noted above, they do not provide details on how phosphatase activity against histone H3 phosphorylation was screened. It remains possible that SCP4 also dephosphorylates other histone H3 phosphorylation sites, such as H3S10 or H3T11, as previously demonstrated for Repo-Man/PP1. The authors should demonstrate the specificity of SCP4 toward H3T3ph relative to other histone H3 phosphorylation marks, and clarify how SCP4 functions as a histone H3 phosphatase during mitosis. This point is particularly important for their proposed model in Figure 5, which states that "Aberrant H3T3 phosphorylation leads to mitotic defects and increased chromosomal instability".*

Response: We appreciate the reviewer's thoughtful comments and suggestions.

We have added part of the systematic screening data to the manuscript as a new figure (Figure EV1A, EV1B). As described, plasmids encoding members of the serine-threonine family phosphatase, including Protein Phosphatase 1, Protein Phosphatase 2, nuclear magnesium-dependent protein phosphatases, and C-terminal domain phosphatase family members, were co-transfected with H3 in HEK293T cells individually. The effect of each phosphatase on the phosphorylation level of H3T3 was determined by WB using an anti-pT3 specific antibody. We consistently observed that H3pT3 was dephosphorylated by PPP1CC (the catalytic subunit of PP1γ) and SCP4 when compared to the other phosphatases tested (Figure EV1A, EV1B).

We have also presented the data on the effect of SCP4 on other H3 phosphorylation sites in the new Figure EV1D. These results showed that SCP4 affects the phosphorylation of mitosis-related sites T3 and S10, but not T11 and S28.

(3) *On page 4, the authors state that "this phenotype distinguishes SCP4 from Repo-Man/PP1γ, which regulates H3T3 dephosphorylation late in mitosis." However, several of the cited studies report (for example, supplemental data in PMID: 21514157) that PP1 knockdown produces a similar retention of H3T3ph levels in mitosis, comparable to what is shown in Figure 1D of this manuscript. To strengthen their claim, it would be important to assess the effects of altering the Repo-Man/PP1γpathway directly, especially given the differences in cell type and experimental system compared with the referenced studies. Additionally, it is recommended that the authors provide a quantification of the Western blot*

signal over the time course, normalized to total H3. Ideally, this analysis should also include other histone H3 phosphorylation marks, as noted above.

Response: We have added the quantification data to Figure 1D. Although PP1 has some overlapping function with SCP4 during mitosis, PP1 is reported as a strong phosphatase that primarily dephosphorylates H3pT3 and H3pT11 (described in the main figure of PMID: 21514157), and also targets H3pS10 and H3pS28 (reported in the supplemental data in PMID: 21514157), suggesting that PP1 has a broader substrate specificity on H3, whereas SCP4 does not target H3pT11 and H3pS28. This difference reflects a functional and mechanistic distinction between the two phosphatases.

Furthermore, by examining the chromosomal localization of SCP4 and Repo-Man during mitosis using immunofluorescent staining, we observed distinct localization patterns for the two proteins. These data were presented in the manuscript as a new figure (Figure EV2A and EV2B). Briefly, SCP4 remains associated with chromosomes throughout all mitotic phases (Figure EV2A). In contrast, Repo-Man initially localizes in the nucleus in interphase, disperses from prophase to metaphase, and then abruptly recruits to chromosomes at the onset of anaphase (Figure EV2B, consistent with previous reports: PMID: 16492807, PMID: 16998479, PMID: 21820363). This dynamic localization of Repo-Man coordinates with Cyclin B/CDK1 activity, which phosphorylates Repo-Man at multiple sites to regulate its localization.

(4) *In Figure 1E and 1F, the authors performed in vitro kinase assays using recombinant proteins, with Western blot as the primary readout. However, in Figure 1E, H3T3ph is detected even in the absence of Haspin kinase treatment. In Figure 1F, the reduction of H3T3ph signal upon SCP4-WT treatment is very subtle. To strengthen the conclusions, it is recommended that the authors employ alternative readouts, such as 32P-ATP incorporation or phosphoprotein-specific staining. In addition, including H3T3A and/or H3T3D mutants as negative controls would help demonstrate the specificity of SCP4 activity toward H3T3.*

Response: We have quantified the ratio of H3pT3 to total H3 levels, and replaced the original Figures 1E and 1F with new ones derived from new data. In the original Figures 1E and 1F, the H3pT3 signal observed in the absence of Haspin kinase represents a nonspecific background signal. The low efficiency of H3T3 phosphorylation by Haspin was likely due to the GST tag at the N-terminus of H3, which may sterically block the Haspin-substrate interaction. In this revision, we replaced the GST tag at the C-terminus of H3 (H3-GST). This construct showed markedly increased Haspin-mediated phosphorylation efficiency and a more pronounced SCP4-dependent dephosphorylation effect (new Figure 1E and 1F).

Minor comments:

(1) *It would be helpful to enlarge the fluorescence images to improve visualization, even in the digital format.*

Response: We have enlarged the fluorescent images in Figure 3A-C, 4A, 4C, 4E, 4F, and 5A-E to improve clarity.

***(2)** It is recommended to show representative fluorescence images for Figure 5A-C in either main or supplemental figures.*

Response: We have added representative fluorescence images to the manuscript as new panels in Figure 4 (Figure 4E, 4F) and Figure 5 (Figure 5C, 5D).

***(3)** Please indicate not only the number of cells analyzed but also the number of independent experiments performed.*

Response: We have added the information and data on the number of independent experiments performed (N=3) to the manuscript as a new table Table EV1.

***(4)** Related to the above point, please briefly describe the statistical method used in each figure legend. Summarizing the methods only in the Materials and Methods section does not make it clear which statistical test was applied to each figure.*

Response: The following information has been added to corresponding figure legend: For bar graphs in Figure 4B and 4D, data are presented as the mean percentage ± SD from three independent experiments (n = 3). Statistical significance was determined by unpaired two-tailed Student's *t*-test; For bar graphs in Figure 4E and 4F, data are presented as the mean percentage ± SD from three independent experiments (n = 3). Statistical significance was determined by unpaired two-tailed Student's *t*-test; In Figure 5A and 5B, data are presented as the mean percentage ± SD from three independent experiments (n = 3). Statistical significance was determined by unpaired two-tailed Student's *t*-test; In Figure 5C and 5D, data are presented as the mean percentage ± SD from three independent experiments (n = 3). Statistical significance was determined by unpaired two-tailed Student's *t*-test. $*P < 0.05$; $**P < 0.01$; $***P < 0.001$; ns, not significant.

***(5)** Please include the antibody dilution ratios in the Materials and Methods section.*

Response: The dilution ratios for all antibodies used in the manuscript have been added to the Materials and Methods section as a Table of Reagents.

***(6)** Please include the catalog numbers for reagents used in this study.*

Response: The catalog numbers for all reagents used in the manuscript have been added to the Table of Reagents.

***(7)** Please include the uncropped Western Blot membrane raw data in supplemental figures.*

Response: We have provided uncropped WB membrane raw data.

***(8)** In Page#3, typo "Repo-man/PP1r" -> "Repo-Man/PP1γ"*

Response: We appreciate the reviewer's careful review. The error has been corrected.

Reviewer #3

Major comments：

How do the released cells look like? *Transient Histone H3 phosphorylation by Haspin plays an important role in mitotic regulation. In this thorough study Zheng at al propose that SCP4 dephosphorylates H3. On the whole the experiments presented in the manuscript strongly support this hypothesis.*

Response: The nocodazole-synchronized cells were round-shaped. We collected the prometaphase cells via mitotic shake-off and seeded them onto poly-L-lysine-coated plates. Subsequently, the cell culture medium was changed to media with or without MG132/CQ. The cells that arrested at metaphase cells remain round, but those cultured in the absence of MG132/CQ begin flattening and spreading.

Minor comments:

(1) *Fig 1A: Unless mitotic shake off is used nocodazole arrest and release rarely leads to very synchronous G1 cell populations. It is interesting that MG132 treatment, which you would expect arresting cells in metaphase does not eliminate H3 dephosphorylation. Maybe SCP4 dephosphorylates H3 prior to this stage. This could be briefly discussed.*

Response: We synchronized cells at prometaphase with nocodazole and subsequently treated them with MG132/CQ to maintain a high Cyclin B/CDK1 level by inhibiting Cyclin B degradation, thus arresting the cells at metaphase. Previous findings (PMID: 16492807, PMID: 16998479, PMID: 21820363) indicated that high level Cyclin B/CDK1 activity inhibits Repo-Man/PP1γ activity. As shown in Figure EV2B and reported previously (PMID: 16492807, PMID: 16998479, PMID: 21820363), Repo-Man disperses from prometaphase to metaphase but abruptly localizes to the chromosomes at anaphase onset. However, the presence of MG132/CQ does not prevent the decrease in the H3pT3 level, suggesting that another chromosome-binding phosphatase is involved in dephosphorylating H3pT3 under conditions of high Cyclin B/CDK1 activity.

(2) *Fig 3. The results of the IF are indeed very nice but in the way they are represented are anecdotal. It would be useful to add some quantitative data (counting the number of cells with the different patterns).*

Response: Figure 3A-3C presents the immunostaining results for Survivin and H3pT3 level on mitotic cytospin of prometaphase chromosomes after nocodazole-induced synchronization. We have described the results in Figure 3 in more detail in the revised manuscript, including the quantification results. A total of 50 cells were counted for the analysis.

1st Revision - Editorial Decision 27th Mar 2026

Dear Dr. Lin

Thank you for the submission of your revised manuscript to EMBO reports. We have now received the full set of referee reports that is copied below.
As you can see, the referees find that the study is significantly improved during revision and recommend publication with only minor revisions. Before I can accept the manuscript, I need you to address their comments and some points below:

- As a standard procedure, we edit the title and abstract of manuscripts to make them more accessible to a general readership. Please find the suggested versions below my signature.

- Please include the quantification of the image data in the source data files.

- We requested the ORCID ID number be linked to the account in our online system for Xin-Hua Feng and Tingbo Liang, as this is obligatory for all co-corresponding authors.

- As we are switching from a free-text author contribution statement towards a more formal statement based on Contributor Role Taxonomy (CRediT) terms, please remove the present Author Contribution section and instead specify each author's contribution(s) directly in the Author Information page of our submission system during upload of the final manuscript. See https://casrai.org/credit/ for more information.

- Please remove the Authors' email address section. We only need the emails of the corresponding authors that are on the title page.

- Please rename the 'Materials and Methods' section to 'Methods'.

- Please move the study approval from the Acknowledgements section to the Methods section.

- Please include the husbandry conditions for the mice used in the study in the Methods section and fill those sections in the Author Checklist.

- Please include information on blinding, even if no blinding was performed.

- Please rename the Conflict of Interest section into "Disclosure and Competing Interests Statement", in accordance with our updated Guide to Authors (https://www.embopress.org/competing-interests)

- Please move the Disclosure and Competing Interests Statement after the Acknowledgements.

- Please adjust the format of the reference list and of the in-text citations according to EMBO Journal format. In particular, references should be listed in alphabetical order and use et al after 10 author names. Please refer to our Guide to Authors for additional information on EMBO J reference format.

- Please rename the Supplemental Figure Legends to Expanded View Figure Legends.

- Please upload Table EV1 in excel format rather than image format. Remove the legend from the manuscript and provide it in the table file.

- Please note that EMBO press papers are accompanied online by:
A) a short (2 sentences) summary of the findings and their significance,
B) 2-5 short bullet points highlighting the key results, and
C) a synopsis image in .jpg or .png format that is exactly 550 pixels wide and 300-600 pixels high (the height is variable). Please note that the text needs to be legible at the final size. Please upload this information along with your revised manuscript (the text for A and B should be provided in one separate Word file uploaded as Cover Art/Synopsis Image).

- Please remove the Reagent and Tools Table from the manuscript file and upload it as an individual file. For more information, please check https://www.embopress.org/page/journal/14602075/authorguide#structuredmethods and download the template for Reagent Table (attached for your convenience)

- Please note that we need figures with n <5 to have individual data points represented. In particular, consider figures 1F, 2B, 3E, 3F, 4B, 4D, 4E, 4F, 5A, 5B, 5C, 5D, EV1B, EV2C.

Our production/data editors have asked you to clarify several points in the figure legends (see below). Please incorporate these changes in the manuscript and return the revised file with tracked changes with your final manuscript submission.

- Please note that the exact p values are not provided in the legends of figures 3E, F; 4B, D, E, F.
- Please note that the box plots need to be defined in terms of minima, maxima, centre, bounds of box and whiskers, and percentile in the legends of figures 3A, C
- Please note that information related to n is missing in the legends of figures 3A, EV1 B
- Please note that the error bars are not defined in the legends of figures 3F, EV1 B, EV2 C

Kurt Weir
Editor
EMBO Reports

"Mitosis is tightly regulated at multiple levels to ensure chromosome stability. The transient phosphorylation of histone H3 at Threonine 3 (H3T3) during cell division is critical for proper chromosome condensation and the accurate segregation of sister chromatids. While Haspin has been identified as the kinase responsible for H3T3 phosphorylation during mitosis, the phosphatases that counteract this modification to maintain balanced phosphorylation levels remain under investigation. In this study, we systematically screened phosphatases encoded in the human genome and identified the nuclear phosphatase SCP4 as an H3T3 phosphatase. SCP4 modulates H3T3 phosphorylation levels and influences the chromosomal recruitment of chromosomal passenger complex (CPC) during mitosis. Aberrant SCP4 expression leads to defective chromosome separation during metaphase and chromosome lagging in anaphase, resulting in aneuploidy. Notably, in SCP4 knockout mice, zygotes exhibit mitotic defects during the first cleavage at the two-cell stage, highlighting SCP4's essential role in ensuring faithful cell division. In summary, we identify SCP4 as a novel phosphatase regulating H3T3 phosphorylation and chromosome dynamics during mitosis, providing new insights into mechanisms safeguarding genomic stability."

Referee #1:

In this manuscript, the authors report extensive findings around the phosphatase SCP4 that is described to dephosphorylate threonine 3 in histone H3, counteracting the effects of Haspin during the cell cycle. The importance of the reversibility of this phosphorylation mark on histone H3 is highlighted in very detailed and high-quality cell cycle experiments.

The authors have also satisfactorily addressed all the points I have raised in my previous review of the manuscript.

I only have two very minor points to mention that I think they can be addressed by the authors without an extra round of revisions with the referees:

(1) Figures 1F and 2B: bar graphs do not have error bars. If this does not apply to these figures, maybe the authors should mention it (e.g., a representative figure of these experiments is shown).

(2) The authors use a catalytically inactive mutant of SCP4 but this mutant when overexpressed does not seem to have effects in the cell cycle. I wonder why it is not acting as a potential dominant negative, is it because it does not bind to histone H3 as the WT SCP4, as it is shown in Figure 2B? I am not too concerned about this in terms of eventual publication of this article but I still feel this slight point could be discussed in the text, if there is space, of course.

Referee #2:

Although I have a small minor comment, the authors have adequately addressed the concerns raised by the reviewers. In its current form after the minor revision, I support the publication of this manuscript in EMBO Reports.
Minor comment: Some quantified data lack information on the number of samples and/or the number of experimental replicates. For example, this information is missing for Figures 1D, 3A, and 3C. Please include these details.

Referee #3:

My two minor requests in the previous revision seem to have been properly addressed.

EMBOR-2025-62636V2 Decision Letter

Dear Dr. Lin

Thank you for the submission of your revised manuscript to EMBO reports. We have now received the full set of referee reports that is copied below.
As you can see, the referees find that the study is significantly improved during revision and recommend publication with only minor revisions. Before I can accept the manuscript, I need you to address their comments and some points below:

Response: We appreciate the editor and the reviewers for their constructive comments throughout the review process, which have been instrumental in improving the quality of this manuscript. We have addressed all comments by revising the manuscript accordingly and incorporating additional information into the main text and figure legends as appropriate. Below, we provide a detailed, point-by-point response to each comment:

1. As a standard procedure, we edit the title and abstract of manuscripts to make them more accessible to a general readership. Please find the suggested versions below my signature.

Response: We appreciate your professional editing. The Abstract has been revised accordingly.

2. Please include the quantification of the image data in the source data files.

Response: All quantification data have been updated and included in the Source Data File for the following panels: Figures 1D, 1F, 2B, 3A, 3C, 3E-F, 4B, 4D-F, 5A-D, EV1B, and EV2C.

3. We requested the ORCID ID number be linked to the account in our online system for Xin-Hua Feng and Tingbo Liang, as this is obligatory for all co-corresponding authors.

Response: The ORCID ID for Xin-Hua Feng is 0000-0002-4418-0811 and is 0000-0003-3262-2587 for Tingbo Liang. These IDs have been linked to the account as requested.

4. As we are switching from a free-text author contribution statement towards a more formal statement based on Contributor Role Taxonomy (CRediT) terms, specify each author's contribution(s) directly in the Author Information page of our submission system during upload of the final manuscript.
See https://casrai.org/credit/ for more information.

Response: We have specified each author's contributions directly in the Author Information section of the submission system, in accordance with the journal's instructions.

5. Please remove the Authors' email address section. We only need the emails of the corresponding authors that are on the title page.

Response: This has been corrected.

6. Please rename the 'Materials and Methods' section to 'Methods'.

Response: This has been corrected.

7. Please move the study approval from the Acknowledgements section to the Methods section.

Response: This has been corrected.

8. Please include the husbandry conditions for the mice used in the study in the Methods section and fill those sections in the Author Checklist.

Response: Husbandry conditions have been filled in the Author Checklist and also added to the manuscript as follows:

Husbandry Care for Mice: Mice were housed in groups of 2 - 4 per cage under specific pathogen-free (SPF) conditions in a temperature-controlled room maintained at 22 ± 2 °C with a relative humidity of 50–60% and a 12:12 h light/dark cycle. They had ad libitum access to standard rodent chow and filtered tap water. All animals were acclimatized to the facility for at least one week prior to the start of experiments. Cage changes and general husbandry procedures were performed weekly. All procedures were conducted in accordance with institutional animal care guidelines and approved by the local ethics committee.

9. Please include information on blinding, even if no blinding was performed.

Response: Blinding information has been filled in the Author Checklist and also added to the manuscript as follows:

Blinding: While group assignment of mice was based on genotype, all subsequent phenotypic assessments and data analyses were performed by investigators blinded to the mice's genetic status. Group codes were revealed only after all data had been acquired and analyzed.

10. Please rename the Conflict of Interest section into "Disclosure and Competing Interests Statement", in accordance with our updated Guide to Authors (https://www.embopress.org/competing-interests)

Response: This has been corrected.

11. Please move the Disclosure and Competing Interests Statement after the Acknowledgements.

Response: This has been corrected.

12. Please adjust the format of the reference list and of the in-text citations according to EMBO Journal format. In particular, references should be listed in alphabetical order and use et al after 10 author names. Please refer to our Guide to Authors for additional information on EMBO J reference format.

Response: The reference list and in-text citations have been formatted according to the journal's instructions.

13. Please rename the Supplemental Figure Legends to Expanded View Figure Legends.

Response: This has been corrected.

14. Please upload Table EV1 in excel format rather than image format. Remove the legend from the manuscript and provide it in the table file.

Response: We have uploaded Table EV1 in Excel format (.xlsx). The table legend has been removed from the manuscript and is now included directly in the Table EV1 Excel file.

15. Please note that EMBO press papers are accompanied online by:
A) a short (2 sentences) summary of the findings and their significance,
B) 2-5 short bullet points highlighting the key results,
C) a synopsis image in .jpg or .png format that is exactly 550 pixels wide and 300-600 pixels high (the height is variable). Please note that the text needs to be legible at the final size. Please upload this information along with your revised manuscript (the text for A and B should be provided in one separate Word file uploaded as Cover Art/Synopsis Image).

Response: The required information has been provided as follows:

A) Summary of The Findings and Their Significance:

We identify SCP4, a chromatin-associated phosphatase, as a novel and critical regulator of mitotic fidelity and genomic stability. This finding expands our understanding of the molecular mechanisms by which cells prevent mitotic errors that contribute to tumorigenesis and developmental abnormalities.

B) Key Results:

- SCP4 is a specific mitotic phosphatase for the dephosphorylation of histone H3 threonine 3 (H3T3).
- SCP4 controls the precise recruitment of the chromosomal passenger complex (CPC) to mitotic chromosomes.
- Dysregulated SCP4 activity leads to chromosome missegregation and aneuploidy.
- *Scp4* knockout causes catastrophic mitotic defects during zygotic cleavage at the two-cell stage.

C) A synopsis image has been provided in jpg format as instructed (550 pixels wide and 300-600 pixels high). However, as this is a raster image (jpg) rather than vector graphics, some text may appear blurry when enlarged to the final production size. We have provided the best possible version given the current figure resolution.

16. Please remove the Reagent and Tools Table from the manuscript file and upload it as an individual file. For more information, please check https://www.embopress.org/page/journal/14602075/authorguide#structuredmethods and download the template for Reagent Table (attached for your convenience)

Response: This has been corrected.

17. Please note that we need figures with n <5 to have individual data points represented. In particular, consider figures 1F, 2B, 3E, 3F, 4B, 4D, 4E, 4F, 5A, 5B, 5C, 5D, EV1B, EV2C.

Response: We have revised the figures as instructed. For Figures with n <5 (1F, 2B, 3F, 4B, 4D-F, 5A-D, EV1B, and EV2C), individual data points are presented. For Figure 3E, data are presented as individual data points overlaid on a box-and-whisker plot. The Min, Max, median, first quartile

(Q1/bottom box), third quartile (Q3/top box), and whiskers are provided in the accompanying table within the Source Data File. Experimental details are also described in the legend of Figure 3E.

18. Our production/data editors have asked you to clarify several points in the figure legends (see below). Please incorporate these changes in the manuscript and return the revised file with tracked changes with your final manuscript submission.

Response: Requested changes have been made in the revised manuscript, with all revisions tracked.

- Please note that the exact p values are not provided in the legends of figures 3E, F; 4B, D, E, F.

Response: Exact p-values have been provided in the legends for Figures 3E-F, 4B, and 4D-F.

- Please note that the box plots need to be defined in terms of minima, maxima, centre, bounds of box and whiskers, and percentile in the legends of figures 3A, C.

Response: The detailed numerical values for the box plots (Min/Q1/median/Q3/Max) have been included in the Source Data File for Figures 3A and 3C.

- Please note that information related to n is missing in the legends of figures 3A, EV1B.

Response: Information on the number of experimental repeats has been included in the figure legends for Figure 3A (n=3) and for Figure EV1B (n=2).

- Please note that the error bars are not defined in the legends of figures 3F, EV1B, EV2C

Response: The error bars are defined as mean ± SD in the legends of Figures 3F and EV2C. The bar graph in Figure EV1B has been replaced with an individual data point plot, as the screen assay was performed twice (n=2).

Kurt Weir
Editor
EMBO Reports

"Mitosis is tightly regulated at multiple levels to ensure chromosome stability. The transient phosphorylation of histone H3 at Threonine 3 (H3T3) during cell division is critical for proper chromosome condensation and the accurate segregation of sister chromatids. While Haspin has been identified as the kinase responsible for H3T3 phosphorylation during mitosis, the phosphatases that counteract this modification to maintain balanced phosphorylation levels remain under investigation. In this study, we systematically screened phosphatases encoded in the human genome and identified the nuclear phosphatase SCP4 as an H3T3 phosphatase. SCP4 modulates H3T3 phosphorylation levels and influences the chromosomal recruitment of chromosomal passenger complex (CPC) during mitosis. Aberrant SCP4 expression leads to defective chromosome separation during metaphase and chromosome lagging in anaphase, resulting in aneuploidy. Notably, in SCP4 knockout mice, zygotes exhibit mitotic defects during the first cleavage at the two-cell stage, highlighting SCP4's essential role in ensuring faithful cell division. In summary, we identify SCP4 as a novel phosphatase regulating H3T3 phosphorylation and chromosome dynamics during mitosis, providing new insights into mechanisms

safeguarding genomic stability."

Referee #1:

In this manuscript, the authors report extensive findings around the phosphatase SCP4 that is described to dephosphorylate threonine 3 in histone H3, counteracting the effects of Haspin during the cell cycle. The importance of the reversibility of this phosphorylation mark on histone H3 is highlighted in very detailed and high-quality cell cycle experiments.

The authors have also satisfactorily addressed all the points I have raised in my previous review of the manuscript.

I only have two very minor points to mention that I think they can be addressed by the authors without an extra round of revisions with the referees:

(1) Figures 1F and 2B: bar graphs do not have error bars. If this does not apply to these figures, maybe the authors should mention it (e.g., a representative figure of these experiments is shown).

Response: In the original submission, the bar graphs displayed results from only the first of the three independent experiments. In the revised manuscript, all three replicates are now presented as individual data points, with a representative western blotting result also shown.

(2) The authors use a catalytically inactive mutant of SCP4 but this mutant when overexpressed does not seem to have effects in the cell cycle. I wonder why it is not acting as a potential dominant negative, is it because it does not bind to histone H3 as the WT SCP4, as it is shown in Figure 2B? I am not too concerned about this in terms of eventual publication of this article but I still feel this slight point could be discussed in the text, if there is space, of course.

Response: We agree with the reviewer's assessment. We speculate that the catalytically inactive SCP4-DN mutant of SCP4 has low binding affinity to histone H3 because the negatively charged aspartic acid (Asp, D) is replaced by the uncharged asparagine (Asn, N). The physiological level of wild-type SCP4 is sufficient to overcome any potential dominant-negative effect of the mutant.

Referee #2:

Although I have a small minor comment, the authors have adequately addressed the concerns raised by the reviewers. In its current form after the minor revision, I support the publication of this manuscript in EMBO Reports.

Minor comment: Some quantified data lack information on the number of samples and/or the number of experimental replicates. For example, this information is missing for Figures 1D, 3A, and 3C. Please include these details.

Response: We have revised the legends of Figures 1D, 3A, and 3C to include information on the number of experimental repeats. In the revised manuscript, the line graph in Figure 1D presents results from the first of the three independent experiments, and all raw data have been provided in the Source Data File. For the box plots in Figures 3A and 3C, quantification of IF staining was performed across three independent transfections (n=3), with results from the first experiment shown as representative

data. Detailed statistical information, including the Min, Max, median, first percentile (Q1/bottom box), third percentile (Q3/top box), and whiskers, is provided in the Source Data File.

Referee #3:

My two minor requests in the previous revision seem to have been properly addressed.

Response: Thank you.

2nd Revision - Editorial Decision 29th May 2026

Xia Lin
The First Affiliated Hospital, Zhejiang University School of Medicine
Department of Hepatobiliary and Pancreatic Surgery and Zhejiang Provincial Key Laboratory of Pancreatic Disease
Hangzhou 310003
China

Dear Dr. Lin,

I am pleased to inform you that your manuscript has been accepted for publication in EMBO reports. Your manuscript will be processed for publication by EMBO Press. It will be copy edited and you will receive page proofs prior to publication. Please note that you will be contacted by Springer Nature Author Services to complete licensing and payment information.

You may qualify for financial assistance for your publication charges - either via a Springer Nature fully open access agreement or an EMBO initiative. Check your eligibility: https://link.springer.com/journal/44319/how-to-publish-with-us

Should you be planning a Press Release on your article, please notify the production team via this form in order to coordinate publication and release dates: https://forms.cloud.microsoft/e/eH4A8JVP6g

If you have any questions, please do not hesitate to contact the Editorial Office. Thank you for your contribution to EMBO Reports.

Yours sincerely,

Kurt Weir
Editor
EMBO Reports

>>> Please note that it is EMBO Reports policy for the transcript of the editorial process (containing referee reports and your response letter) to be published as an online supplement to each paper. If you do NOT want this, you will need to inform the Editorial Office via email immediately. More information is available here: https://link.springer.com/partners/embo-press/editorial-policies#Peer%20review

EMBO Press Author Checklist

Corresponding Author Name: Xia Lin, Xin-Hua Feng, Tingbo Liang
Journal Submitted to: EMBO Reports
Manuscript Number: EMBOR-2025-62636V3

USEFUL LINKS FOR COMPLETING THIS FORM
The EMBO Journal - Author Guidelines
EMBO Reports - Author Guidelines
Molecular Systems Biology - Author Guidelines
EMBO Molecular Medicine - Author Guidelines

Reporting Checklist for Life Science Articles (updated January
This checklist is adapted from Materials Design Analysis Reporting (MDAR) Checklist for Authors. MDAR establishes a minimum set of requirements in transparent reporting in the life sciences (see Statement of Task: 10.31222/osf.io/9sm4x). Please follow the journal's guidelines in preparing your
Please note that a copy of this checklist will be published alongside your article.

Abridged guidelines for figures
1. Data
The data shown in figures should satisfy the following conditions:
- the data were obtained and processed according to the field's best practice and are presented to reflect the results of the experiments in an accurate and unbiased manner.
- ideally, figure panels should include only measurements that are directly comparable to each other and obtained with the same assay.
- plots include clearly labeled error bars for independent experiments and sample sizes. Unless justified, error bars should not be shown for technical
- if n<5, the individual data points from each experiment should be plotted. Any statistical test employed should be justified.
- Source Data should be included to report the data underlying figures according to the guidelines set out in the authorship guidelines on Data

2. Captions
Each figure caption should contain the following information, for each panel where they are relevant:
- a specification of the experimental system investigated (eg cell line, species name).
- the assay(s) and method(s) used to carry out the reported observations and measurements.
- an explicit mention of the biological and chemical entity(ies) that are being measured.
- an explicit mention of the biological and chemical entity(ies) that are altered/varied/perturbed in a controlled manner.
- the exact sample size (n) for each experimental group/condition, given as a number, not a range;
- a description of the sample collection allowing the reader to understand whether the samples represent technical or biological replicates (including how many animals, litters, cultures, etc.).
- a statement of how many times the experiment shown was independently replicated in the laboratory.
- definitions of statistical methods and measures:
  - common tests, such as t-test (please specify whether paired vs. unpaired), simple χ2 tests, Wilcoxon and Mann-Whitney tests, can be unambiguously identified by name only, but more complex techniques should be described in the methods section;
  - are tests one-sided or two-sided?
  - are there adjustments for multiple comparisons?
  - exact statistical test results, e.g., P values = x but not P values < x;
  - definition of 'center values' as median or average;
  - definition of error bars as s.d. or s.e.m.

Please complete ALL of the questions below.
Select "Not Applicable" only when the requested information is not relevant for your study.

Materials

Newly Created Materials	Information included in the manuscript?	In which section is the information available? (Reagents and Tools Table, Materials and Methods, Figures, Data Availability Section)
New materials and reagents need to be available; do any restrictions apply?	Not Applicable	

Antibodies	Information included in the manuscript?	In which section is the information available? (Reagents and Tools Table, Materials and Methods, Figures, Data Availability Section)
For **antibodies** provide the following information:<br>- Commercial antibodies: RRID (if possible) or supplier name, catalogue number and or/clone number<br>- Non-commercial: RRID or citation	Yes	Reagents and Tools Table

DNA and RNA sequences	Information included in the manuscript?	In which section is the information available? (Reagents and Tools Table, Materials and Methods, Figures, Data Availability Section)
**Short novel DNA or RNA including primers, probes**: provide the sequences.	Yes	Reagents and Tools Table, Methods

Cell materials	Information included in the manuscript?	In which section is the information available? (Reagents and Tools Table, Materials and Methods, Figures, Data Availability Section)
**Cell lines:** Provide species information, strain. Provide accession number in repository **OR** supplier name, catalog number, clone number, and/**OR** RRID.	Yes	Reagents and Tools Table
**Primary cultures:** Provide species, strain, sex of origin, genetic modification status.	Not Applicable	
Report if the cell lines were recently **authenticated** (e.g., by STR profiling) and tested for mycoplasma contamination.	Not Applicable	

Experimental animals	Information included in the manuscript?	In which section is the information available? (Reagents and Tools Table, Materials and Methods, Figures, Data Availability Section)
**Laboratory animals or Model organisms:** Provide species, strain, sex, age, genetic modification status. Provide accession number in repository **OR** supplier name, catalog number, clone number, **OR** RRID.	Not Applicable	
**Animal observed in or captured from the field:** Provide species, sex, and age where possible.	Not Applicable	
Please detail **housing and husbandry conditions**.	Yes	Methods

Plants and microbes	Information included in the manuscript?	In which section is the information available? (Reagents and Tools Table, Materials and Methods, Figures, Data Availability Section)
**Plants:** provide species and strain, ecotype and cultivar where relevant, unique accession number if available, and source (including location for collected wild specimens).	Not Applicable	
**Microbes:** provide species and strain, unique accession number if available, and source.	Not Applicable	

Human research participants	Information included in the manuscript?	In which section is the information available? (Reagents and Tools Table, Materials and Methods, Figures, Data Availability Section)
If collected and within the bounds of privacy constraints report on age, sex and gender or ethnicity for all study participants.	Not Applicable	

Core facilities	Information included in the manuscript?	In which section is the information available? (Reagents and Tools Table, Materials and Methods, Figures, Data Availability Section)
If your work benefited from core facilities, was their service mentioned in the acknowledgments section?	Not Applicable	

Design

Study protocol	Information included in the manuscript?	In which section is the information available? (Reagents and Tools Table, Materials and Methods, Figures, Data Availability Section)
If study protocol has been **pre-registered, provide DOI in the manuscript**. For clinical trials, provide the trial registration number **OR** cite DOI.	Not Applicable	
Report the **clinical trial registration number** (at ClinicalTrials.gov or equivalent), where applicable.	Not Applicable	

Laboratory protocol	Information included in the manuscript?	In which section is the information available? (Reagents and Tools Table, Materials and Methods, Figures, Data Availability Section)
Provide DOI OR other citation details if **external detailed step-by-step protocols** are available.	Not Applicable	

Experimental study design and statistics	Information included in the manuscript?	In which section is the information available? (Reagents and Tools Table, Materials and Methods, Figures, Data Availability Section)
Include a statement about **sample size** estimate even if no statistical methods were used.	Yes	Figure legends
Were any steps taken to minimize the effects of subjective bias when allocating animals/samples to treatment (e.g. **randomization procedure**)? If yes, have they been described?	Not Applicable	
Include a statement about **blinding** even if no blinding was done.	Yes	Methods
Describe **inclusion/exclusion criteria** if samples or animals were excluded from the analysis. Were the criteria pre-established?<br><br>If sample or data points were omitted from analysis, report if this was due to attrition or intentional exclusion and provide justification.	Not Applicable	
For every figure, are **statistical tests** justified as appropriate? Do the data meet the assumptions of the tests (e.g., normal distribution)? Describe any methods used to assess it. Is there an estimate of variation within each group of data? Is the variance similar between the groups that are being statistically compared?	Not Applicable	

Sample definition and in-laboratory replication	Information included in the manuscript?	In which section is the information available? (Reagents and Tools Table, Materials and Methods, Figures, Data Availability Section)
In the figure legends: state number of times the experiment was **replicated** in laboratory.	Yes	Figure legends
In the figure legends: define whether data describe **technical or biological replicates**.	Yes	Figure legends

Ethics

Ethics	Information included in the manuscript?	In which section is the information available? (Reagents and Tools Table, Materials and Methods, Figures, Data Availability Section)
Studies involving **human participants**: State details of **authority granting ethics approval** (IRB or equivalent committee(s), provide reference number for approval.	Not Applicable	
Studies involving **human participants**: Include a statement confirming that **informed consent** was obtained from all subjects and that the experiments conformed to the principles set out in the WMA Declaration of Helsinki and the Department of Health and Human Services Belmont Report.	Not Applicable	
Studies involving **human participants:** For publication of **patient photos**, include a statement confirming that consent to publish was obtained.	Not Applicable	
Studies involving experimental **animals**: State details of **authority granting ethics approval** (IRB or equivalent committee(s), provide reference number for approval. Include a statement of compliance with ethical regulations.	Yes	Methods
Studies involving **specimen and field samples:** State if relevant **permits** obtained, provide details of authority approving study; if none were required, explain why.	Not Applicable	

Dual Use Research of Concern (DURC)	Information included in the manuscript?	In which section is the information available? (Reagents and Tools Table, Materials and Methods, Figures, Data Availability Section)
Could your study fall under dual use research restrictions? Please check biosecurity documents and list of **select agents and toxins** (CDC): https://www.selectagents.gov/sat/list.htm	Not Applicable	
If you used a select agent, is the security level of the lab appropriate and reported in the manuscript?	Not Applicable	
If a study is subject to dual use research of concern regulations, is the name of the **authority granting approval and reference number** for the regulatory approval provided in the manuscript?	Not Applicable	

Reporting

The MDAR framework recommends adoption of discipline-specific guidelines, established and endorsed through community initiatives. Journals have their own policy about requiring specific guidelines and recommendations to complement MDAR.

Adherence to community standards	Information included in the manuscript?	In which section is the information available? (Reagents and Tools Table, Materials and Methods, Figures, Data Availability Section)
State if relevant guidelines or checklists (e.g., **ICMJE, MIBBI, ARRIVE, PRISMA**) have been followed or provided.	Not Applicable	
For **tumor marker prognostic studies,** we recommend that you follow the **REMARK** reporting guidelines (see link list at top right). See author guidelines, under 'Reporting Guidelines'. Please confirm you have followed these guidelines.	Not Applicable	
For **phase II and III randomized controlled trials**, please refer to the **CONSORT** flow diagram (see link list at top right) and submit the CONSORT checklist (see link list at top right) with your submission. See author guidelines, under 'Reporting Guidelines'. Please confirm you have submitted this list.	Not Applicable	

Data Availability

Data availability	Information included in the manuscript?	In which section is the information available? (Reagents and Tools Table, Materials and Methods, Figures, Data Availability Section)
Have **primary datasets** been deposited according to the journal's guidelines (see 'Data Deposition' section) and the respective accession numbers provided in the Data Availability Section?	Not Applicable	
Were **human clinical and genomic datasets** deposited in a public access-controlled repository in accordance to ethical obligations to the patients and to the applicable consent agreement?	Not Applicable	
Are **computational models** that are central and integral to a study available without restrictions in a machine-readable form? Were the relevant accession numbers or links provided?	Not Applicable	
If publicly available data were reused, provide the respective **data citations in the reference list**.	Not Applicable	
